# Barriers and facilitators to hepatitis B birth dose vaccination: Perspectives from healthcare providers and pregnant women accessing antenatal care in Nigeria

**DOI:** 10.1371/journal.pgph.0001332

**Published:** 2023-06-08

**Authors:** Catherine Freeland, Florence Kanu, Yahaya Mohammed, Ugochukwu Uzoechina Nwokoro, Hardeep Sandhu, Hadley Ikwe, Belinda Uba, Adeyelu Asekun, Charles Akataobi, Adefisoye Adewole, Rhoda Fadahunsi, Margeret Wisdom, Okeke Lilian Akudo, Gideon Ugbenyo, Edwin Simple, Ndadilnasiya Waziri, James Jacob Vasumu, Abubakar Umar Bahuli, Suleiman Saidu Bashir, Abdullahi Isa, George Onyemachi Ugwu, Emmanuel Ikechukwu Obi, Haj Binta, Bassey Okposen Bassey, Faisal Shuaib, Omotayo Bolu, Rania A. Tohme

**Affiliations:** 1 Hepatitis B Foundation, Doylestown, Pennsylvania, United States of America; 2 Global Immunization Division, United States, Centers for Disease Control and Prevention, Atlanta, Georgia, United States of America; 3 Department of Medical Microbiology and Parasitology, College of Health Sciences, Usmanu Danfodiyo University, Sokoto, Nigeria; 4 African Field Epidemiology Network, Abuja, Nigeria; 5 Department of Community Medicine, University of Nigeria Teaching Hospital, Enugu, Nigeria; 6 Centers for Disease Control and Prevention Nigeria, Abuja, Nigeria; 7 National Emergency Routine Immunization Coordination Centre, Abuja, Nigeria; 8 National Stop Transmission of Poliomyelitis, Abuja, Nigeria; 9 Adamawa State Primary Health Care Development Agency, Yola, Adamawa State, Nigeria; 10 Department of Obstetrics and Gynaecology, College of Medicine, University of Nsukka, Nsukka, Nigeria; 11 Enugu State Primary Health Care Development Agency, Enugu, Nigeria; 12 Department of Community Medicine, Faculty of Medical Sciences, College of Medicine, University of Nigeria/ University of Nigeria Teaching Hospital, Enugu, Nigeria; 13 National Primary Health Care Development Agency, Abuja, Nigeria; University of Cape Town, SOUTH AFRICA

## Abstract

Nigeria is estimated to have the largest number of children worldwide, living with chronic hepatitis B virus (HBV) infection, the leading cause of liver cancer. Up to 90% of children infected at birth develop chronic HBV infection. A birth dose of the hepatitis B vaccine (HepB-BD) followed by at least two additional vaccine doses is recommended for prevention. This study assessed barriers and facilitators of HepB-BD administration and uptake, using structured interviews with healthcare providers and pregnant women in Adamawa and Enugu States, Nigeria. The Consolidated Framework for Implementation Sciences Research (CFIR) guided data collection and analysis. We interviewed 87 key informants (40 healthcare providers and 47 pregnant women) and created a codebook for data analysis. Codes were developed by reviewing the literature and reading a subsample of queries line-by-line. The overarching themes identified as barriers among healthcare providers were: the lack of hepatitis B knowledge, limited availability of HepB-BD to vaccination days only, misconceptions about HepB-BD vaccination, challenges in health facility staffing capacity, costs associated with vaccine transportation, and concerns related to vaccine wastage. Facilitators of timely HepB-BD vaccination included: vaccine availability, storage, and hospital births occurring during immunization days. Overarching themes identified as barriers among pregnant women were lack of hepatitis B knowledge, limited understanding of HepB-BD importance, and limited access to vaccines for births occurring outside of a health facility. Facilitators were high vaccine acceptance and willingness for their infants to receive HepB-BD if recommended by providers. Findings indicate the need for enhanced HepB-BD vaccination training for HCWs, educating pregnant women on HBV and the importance of timely HepB-BD, updating policies to enable HepB-BD administration within 24 hours of birth, expanding HepB-BD availability in public and private hospital maternity wards for all facility births, and outreach activities to reach home births.

## Introduction

Chronic hepatitis B virus (HBV) infection is a major cause of liver cancer, with 296 million individuals affected worldwide [1]. HBV is transmitted through direct contact with infected blood and bodily fluids, unprotected sex with an infected individual, use of contaminated medical or injection equipment, and vertical transmission from an infected mother to her newborn during childbirth [2, 3]. Children infected with HBV at birth have a greater than 90% chance of developing chronic HBV infection and substantially increase new infections globally [4, 5].

The World Health Organization (WHO) has committed to the elimination of mother-to-child-transmission (MTCT) of HBV with an impact target of ≤ 0.1% hepatitis B surface antigen (HBsAg) prevalence in children under five by 2030 and programmatic targets of ≥ 90% coverage with timely hepatitis B vaccine birth dose (HepB-BD) and three doses of hepatitis B vaccine (HepB3) [6]. To prevent HBV infection, WHO recommends at minimum, all countries include three doses of hepatitis B vaccine (HepB) within routine immunization schedules, with the first dose given to infants within 24 hours of birth [7]. All countries in the WHO African Region have introduced the HepB infant series (included as part of the diphtheria-pertussis-tetanus-*Haemophilus influenzae* type b-hepatitis B pentavalent combination vaccination) given at six, ten, and 14 weeks of age. However, as of 2021 only 14 (28%) of 47 WHO AFRO member countries have introduced HepB-BD [8]. Without timely HepB-BD, children remain at risk of HBV infection during the first six weeks of life.

Despite the availability of hepatitis B vaccine, cost, knowledge, and access barriers limit the vaccine’s broad use within low- and middle-income countries, particularly in Africa where HBV is endemic [9]. These barriers include the lack of clear policy emphasizing the importance of timely HepB-BD, the lack of disease and burden awareness, supply chain challenges, limited evidence on HBV seroprevalence and rates of MTCT, vaccine costs and distribution, limited cold chain storage, the lack of trained community health workers, and a high proportion of births taking place outside of health facilities [10–13].

Globally, Nigeria carries one of the highest burdens of hepatitis B with a prevalence of 12.2% and has the largest number of children infected with chronic HBV worldwide [8, 14]. The hepatitis B vaccination schedule in Nigeria allows for the birth dose to be given within 24 hours and up to two weeks of birth, followed by three doses of the pentavalent vaccine. All newborns delivered within public health facilities in Nigeria are eligible to receive free routine immunizations, including the birth dose; however, newborns delivered at private facilities or by traditional birth attendants (TBAs) may not get timely HepB-BD. This study aimed to assess barriers and facilitators of HepB-BD administration and uptake in Nigeria, using qualitative interviews with pregnant women, community health volunteers (CHVs), and healthcare workers (HCWs) involved in labor and delivery and routine immunization services in Adamawa and Enugu states.

## Materials and methods

### Study population

This study was conducted in a Northeastern state (Adamawa) and Southeastern state (Enugu) in Nigeria; HCWs, CHVs, and pregnant women attending antenatal care services at primary care facilities in Adamawa and Enugu states, Nigeria, were eligible to participate. Study staff approached HCWs, CHVs, and pregnant women in August and September 2021 working or receiving care at the selected health facilities. Participation in this study was voluntary and not compensated. Only the location of the interview and worker title (e.g., HCW or CHV) were collected from participants; no other identifiable information was obtained. We paid attention to equal representation from each state and achieved a total sample of 87 individuals from Adamawa and Enugu (32 HCWs, 8 CHVs, and 47 pregnant women).

States selected represented both the Northern and Southern regions of Nigeria because each region has unique cultural, religious, linguistic, and population-level characteristics that impact health services. Epidemiological data reflect regional differences in HBV prevalence, which tends to be higher in Northern Nigeria because it is more rural [15]. For example, urban women (84%) were more likely than rural women (56%) to receive antenatal care from a skilled provider, and antenatal care disparities also exist among women based on socioeconomic characteristics (education and wealth) [16]. Within Nigeria, 39% of women delivered their last live birth in a health facility, and of these women, 26% delivered in a public health facility, while 13% delivered in a private health facility. Newborns delivered within a health facility were more likely to receive a postnatal health check within two days of birth than those delivered elsewhere [16]. In Enugu State, 78–95% of live births were delivered in a health facility compared to 26–42% in Adamawa State. Based on 2018 Demographic and Health Survey, total HepB-BD coverage (classified in Nigeria as a birth dose administered within 24 hours of birth or after 24 hours up to two weeks of age) was 87% in Enugu and 45% in Adamawa [16].

### Ethics statement

The study protocol was reviewed by the U.S. Centers for Disease Control and Prevention (CDC) under human research protection procedures and was approved by the institutional ethics committee of the state ministries of health in Adamawa [ADHREC29/07/2021/061] and Enugu [MH/MSD/REC21/184] States. We obtained verbal informed consent before all discussions to ensure each participant was informed of the risks, benefits, and purpose of their participation. We did not collect identifiable nor sensitive information from participants.

### Study materials

Each study population (HCWs, CHVs, and pregnant women) had a unique but complementary interview guide for discussion (S1 Text). The Consolidated Framework for Implementation Research (CFIR) model [17] was used in addition to a literature review to identify constructs of importance for each qualitative interview guide. The CFIR includes 39 constructs across five domains that are considered influential in implementation outcomes, and it provides a structure to systematically assess the context in which implementation occurs [17–19]. The CFIR model guided data collection and analysis for this study. The first domain of the CFIR is related to the characteristics of the intervention being implemented in a particular organization. The second and third CFIR domains are the inner and outer settings. Changes in the outer setting can influence implementation, often mediated through changes in the inner setting. Generally, the outer setting includes the economic, political, cultural, and social contexts through which the implementation process will occur [20]. The fourth CFIR domain is the individuals involved in the intervention and the implementation process (i.e., HCWs, CHVs, and pregnant women). These individuals are carriers of cultural, organizational, professional, and individual mindsets, norms, interests, and affiliations [20]. The fifth domain is the implementation process [20, 21]. The interview guide for HCWs and CHVs involved in labor and delivery and routine immunization included questions on challenges, needs, and the importance of HepB-BD administration. HCWs were also asked about their roles and responsibilities in vaccine administration, barriers, and facilitators to timely administration of HepB-BD. The interview guide for pregnant women included questions on HBV and HepB-BD knowledge, attitude, perceptions, and experiences related to HepB-BD administration.

### Data collection

Interviews took place within the HCWs respective primary care government run facilities at the selected study sites and the designation of each HCW was noted. Interviews were audio recorded and transcribed verbatim. More than half of the interviews (29; 62%) were conducted in English, while 18 (38%) interviews with pregnant women were conducted in the local language (Hausa or Igbo). The use of a translator required back translation to ensure translation accuracy. Participants did not review transcripts after interviews were transcribed and translated.

### Data analysis

We analyzed the data, using qualitative techniques by creating a codebook for the organization of data; the codes were developed after reviewing the literature (*a priori*) and through the line-by-line reading of a subsample of queries (Table 1) [22]. We gave a specific definition for each code to ensure coding accuracy and improve inter-coder reliability (ICR) [23]. We used NVivo 12 software (QRS International, Doncaster, Australia) for data coding and analysis. Two researchers independently double-coded all data to ensure coding accuracy, and we assessed ICR repeatedly, using the kappa coefficient to identify coding discrepancies. A kappa score of .80 or greater indicated an acceptable coding agreement. The analysis team met throughout the coding process to discuss and resolve differences in coding. After coding was complete, it was reviewed and organized into thematic categories.

**Table 1:** The theme identified, description, volume reported in each transcript (file), and reference volume within transcripts.

Name	Description	Files	References
Access to birth dose	Use when the participant describes how easy or difficult it might be to get the hepatitis B birth dose.	49	112
Barriers	Use when the participant describes any challenges associated with the hepatitis B infection and hepatitis B immunization. Code according to the sub-code definitions	15	16
Barrier—Cost	Use when the participant is referring to challenges accessing immunization due to cost	12	17
Barrier—Reporting, Monitoring	Use when the participant is discussing challenges associated with monitoring or reporting hepatitis B/Hep B-BD	2	2
Barrier—Access to immunization	Use when participant is reporting challenges in accessing immunization	17	26
Cultural Challenges	Use when participant describes challenges with immunization because of community cultural norms and perceptions about immunization	8	13
Delivery Location	Use when the participant describes challenges to accessing vaccine because of location (rural areas, etc.)	3	7
Follow-up visits	Use when the participant is reporting challenges in follow-up visits for immunization	1	1
Resources	Use when the participant reports challenges to immunization because of resource limitations (stipends, incentives, materials, etc.)	11	13
Short staffing	Use when the participant reports staffing-related challenges or staff motivation to conduct immunization services	10	18
Time challenges	Use when the participant reports challenges related to the recommendation of administering HepB-BD within 24 hours	5	6
Transportation	Use when the participant describes transportation-related to get immunization	11	21
Vaccine wastage	Use when the participant discusses concerns about vaccination wastage	7	8
Birth Dose Perceptions	Use when the participant describes the current perceptions of HepB-BD	49	186
Challenges with HBV	Use when the participant talks about hepatitis B- specific challenges, including diagnostics, screening, treatment, management, and infrastructure related to hepatitis B management and care	37	73
Concerns about HepB-BD	Use when the participant describes any concerns about the safety or administration of HepB-BD	12	16
Current HBV Policies	Use when the participant describes the current policies or programs regarding HepB-BD in their clinic	41	173
HBV Needs	Use when the participant describes specific needs related to hepatitis B in their community, including having a hepatitis B infection	34	101
HepB-BD Understanding	Use when the participant describes what they know about hepatitis B vaccine birth dose or how they feel about the hepatitis B birth dose	47	185
Lack of Knowledge—HBV	Use when the participant describes lack of hepatitis B knowledge	32	56
Learning Preferences	Use when individual is describing learning preferences for hepatitis B information	26	57
Misconceptions about HBV	Use when the participant describes a misconception regarding hepatitis b virus (transmission, treatment, cure, etc.)	10	13
Recommendations	Use when the participant describes recommendations or suggestions for sensitization, educational, or awareness communication, campaigns, or messaging needs related to hepatitis B infection or HepB-BD.	51	300
Successful Programs	Use when the participant discusses current successful programs within their health facility or past programs that had a positive impact based on their perspective	32	68

## Results

In total, we completed 87 key informant interviews in Adamawa and Enugu. A total of 40 Healthcare workers (HCWs) comprising 7 Doctors, 3 community health extension workers, 7 routine immunization focal persons, 7 officers in charge (HCW in charge of the facility), 8 midwives, and 8 CHVs were interviewed, with equal numbers from each state. The majority of officers in charge, community health extension workers, community volunteers, and midwives were female (73%) and all doctors interviewed were male. All HCWs interviewed were between the ages of 23 to 57 years with an average age of 36 years. The remaining 47 informants were pregnant women attending antenatal care at select health facilities in each state. Findings demonstrate barriers and facilitators to timely HepB-BD and were thematically defined, using quotes describing the overarching themes organized by healthcare providers and pregnant women. The codebook used to organize thematic findings with the corresponding frequencies (See Table 1).

### Healthcare worker and community volunteer findings

#### Healthcare worker and community health volunteer findings

The analysis of our qualitative interviews with HCWs and CHVs revealed that the overarching themes related to barriers were the lack of HBV-specific knowledge in the community, limited staffing within the health facility, unavailability of a specific budget for vaccine transportation to the health facility, limited availability of HepB-BD outside of immunization days, the lack of outreach to provide timely HepB-BD to children born outside of health facilities or outside of fixed immunization days, and misconceptions on HepB-BD timeliness and contraindications. Facilitators were the lack of vaccine stock-outs as a result of solar-powered refrigerators, the community’s trust in HCWs, and awareness among HCWs about the need for expanded sensitization and knowledge on HBV and HepB-BD. Constructs identified from findings were outlined by the CFIR (Table 2).

**Table 2:** Consolidated Framework for Implementation Science Research (CFIR) identified barriers and facilitators from interviews by healthcare workers and pregnant women related to hepatitis B and HepB-BD.

CFIR Domain	Theme	Facilitator	Barrier
Healthcare Workers (including nurses, doctors, and community health volunteers)			
Inner Setting/Characteristics of Individuals	Knowledge	Knowledge of hepatitis B and its impact on the liver was reported The need for more awareness and sensitization on hepatitis B through HCW training was acknowledged	Specific knowledge about hepatitis B (e.g., symptoms, transmission) was commonly incorrect. reported.Misconceptions about transmission existed widely.
Inner Setting	Diagnosis and treatment of HBV infection	No identified facilitators	Costly medication and treatment The need for specialized care or referrals for HBV infection was a burden to accessing care and management Low community-level diagnosis Limited infrastructure and resources for diagnosis and treatment of HBV infection
Characteristics of individuals	Challenges with HBV Awareness in the community	HCWs identified the need for more sensitization, knowledge, and awareness on the hepatitis B disease at all levels.	Common misconceptions associated with HBV within the community (witchcraft, low awareness/knowledge)
Inner setting	HepB-BD availability and accessibility	Cold chain equipment was reported to be strong (solar refrigeration was reported in each state widely) HepB-BD availability at the health facility It was reported that, in many health facilities, HCWs were managing immunization regularly (checking cold chain equipment, storage, and appropriately tracking vaccine stock)	Rural areas have limited access to health facilities (challenges with the cost of transportation) Traditional birth attendants (TBAs) and home births are commonly used and they do not have access to HepB-BD In some facilities, scheduled immunization days limit the broad accessibility of the vaccine
			Cost of obtaining vaccination (e.g., transportation, time, and distance to the vaccination storage facility) Understaffing of healthcare facilities Vaccine stockouts
Characteristics of individuals	HepB-BD understanding	HCWs agreed that HepB-BD was important HCWs acknowledged the need to focus on the timeliness of HepB-BD	Misconceptions about vaccination wastage described among HCWs Misconceptions about the timeliness of HepB-BD (2 weeks instead of within 24 hours of birth or as soon as possible after birth) Misconceptions about the vaccination contraindications among HCWs
**Pregnant Women**			
Outer setting	Hepatitis B knowledge	No identified facilitators	Low knowledge and awareness related to hepatitis B overall Reported misconceptions about the transmission of hepatitis B
Outer setting/Inner setting	Access to and demand for HepB-BD	Most felt the vaccine would be available to them Most felt that they would ensure their child would get the HepB-BD if their HCW recommended it	Access to people delivering at home with traditional birth attendants or within the private sector (outside government-run health facilities).
Inner setting	HepB-BD challenges	HepB-BD vaccination available and in stock	Cost of obtaining vaccination (e.g., transportation, time, and distance to the vaccination facility) Understaffing of healthcare facilities

#### Hepatitis B knowledge among healthcare providers

The range of knowledge about HBV was highly variable among the HCWs interviewed. Doctors knew significantly more about HBV than other health professionals interviewed (e.g., midwives, nurses); however, doctors were less familiar with routine immunization logistics for hepatitis B, specifically who administered timely immunization and where immunization occurred. Most HCWs knew hepatitis B was a “*virus that normally affects the liver*. *Once you receive the vaccine*, *it will produce antibody against the virus and protect the child from contracting that virus*.” However, misconceptions emerged during interviews with several healthcare providers regarding the transmission of HBV. One nurse described, “*they usually like to hold the baby*, *and some of them might be infected with Hepatitis B without having any symptoms*. *Hence*, *when they hold the baby*, *they may infect it*.”

Similarly, a CV shared, “*if the person that have the disease touch the baby or even the baby inhales it from the carrier*, *the baby may contract the disease*, *so you have to protect the baby before going to the village*.” A nurse midwife in Adamawa shared misconceptions related to the transmission of hepatitis B as “*people can also get it from sweats from a patient link in a vehicle*.” A health facility nurse-in-charge in Enugu described, “*Hep B is a viral infection*. *Mosquito can transfer it from person to person*.” Other misconceptions emerged regarding hepatitis B being curable. One doctor shared, “*hepatitis B is not a death sentence*, *and it is curable*, *and equipment for the test should be made available in the health facilitie*s.” Overwhelmingly, health professionals agreed that there is a strong need for hepatitis B education at the community level. The majority of HCWs and CHVs interviewed acknowledged that the most significant barrier to HBV prevention and control is the lack of knowledge associated with the disease.

#### Challenges with hepatitis B diagnosis and treatment

Health professionals noted the following challenges associated with the lack of knowledge on hepatitis B: a) costly medication and treatment were unaffordable for many people, b) isolation due to the illness, c) referrals causing people to drop out of care, and d) overwhelming general lack of resources and hospitals that accommodate people living with HBV. The majority of health professionals interviewed noted the lack of knowledge in the community regarding the diagnosis of HBV, “*most people having hepatitis B*, *they are not aware that they have it*.” One chief medical officer noted treatment being a significant burden for HBV, describing, “*Yes*, *treatment of hepatitis B infection is not as available*, *because the medication for it is not as usually accessible*, *[it’s] very costly… some prefer carrying their infection and wait for the consequences*.” Similarly, a doctor shared, “*The challenge we have here is that if we test a person and we found out that he has hepatitis*, *we usually advise or refer*. *So*, *the challenge here is that some of them*, *usually don’t go for their drugs*. *And after giving their drugs*, *the fact that the treatment take[s] longer time*. *Because of poverty*, *some of them*, *they will decide to drop the treatment halfway*. *And as a result*, *some will lead to dead*.” Related to challenges accessing care for hepatitis B, a doctor described challenges with referrals, “*Whenever we send them over to go to the state capital to go and get adequate care most of them turn it down due to distance*, *financial constraint*.”A nurse noted symptoms of hepatitis B impacting those living with the disease, “*Hepatitis B is a killer disease*. *Whenever someone is affected*, *the person will be frequently ill*, *sometime[s] they present with oedema*, *abdominal swelling*, *and sometime[s] they will be irritable*.” The lack of resources and infrastructure to manage those living with hepatitis B was further described as “*Then you do the fibroid scan which I doubt if there is any place in Adamawa state that has the fibroid scanning machine*. *Maybe Adamawa German hospital*. *So*, *it is actually very cumbersome*.”

#### Challenges with hepatitis B awareness in the community

Health professionals spoke of the need for increased awareness and sensitization regarding HBV within communities, the need for infrastructure and resources to manage people living with HBV, and the need to spread the word about the importance of immunization. A unique finding identified the lack of a word for hepatitis B within local languages in Adamawa and Enugu, adding to HBV being described by symptoms and causing confusion within the community. One described, “*The need is to create awareness at their settlement*, *at their villages*, *the mode of transmission*, *then the basic symptoms and if they have those symptoms*, *they should present to the hospital as early as possible instead of using local medication*, *herbs*.” Similarly, another doctor spoke about the lack of awareness of their diagnosis among those living with hepatitis B and described, “*I have seen a quite a lot of patients with hep-B and to my surprise*, *most of them are not even aware they have the infection*. *So*, *I think there is lack of awareness*.” A chief medical officer in Enugu described, “*you know the infection is something they don’t know they have*, *sometimes they might be thinking they’re charmed*, *sometimes they might be thinking they are charmed by witchcraft in the society*.*”* A nurse discussed the importance of prevention, “*We also need to educate people about the importance of the vaccine and how to live in a health[y] way to avoid getting infected*. *It is good to start since birth*, *so that we get optimal protection*.”

#### Hepatitis B birth dose availability and access

All health professionals mentioned that the hepatitis B vaccine was easily available and maintaining the cold chain worked well; many noted that solar refrigerators were very helpful. One physician from Enugu mentioned, “*It’s always available*, *we have solar fridge here*. *So*, *the vaccine is always there in the fridge*, *and somebody is always there*.” Similarly, another mentioned, “*because the solar is here*, *at the beginning of the month*, *we do bring all the vaccines we need for the month*. *So most often we do not run out of stock*.” While most HCWs noted that stockouts of the hepatitis B vaccine have not happened in some time thanks to the solar refrigerators, a few did mention a few occurrences. One described, “*The challenge is when the vaccine is out of stock*. *That is the only challenge*.”

Challenges associated with access to HepB-BD for HCW were the costs of transportation and health facility staffing. Similarly, a nurse responsible for immunization services shared, “*the only thing*, *we transport*. *They don’t give us money for collection of vaccine or anything*. *Challenges is that maybe their facility is far from the health local government where they do get the vaccine*.” HCWs in both Adamawa and Enugu also shared that staffing is a significant challenge, a nurse midwife in Enugu explained, “*we don’t have enough workers here*, *we are understaffed*.” Another nurse echoed “*we do not have enough staff so that we will be going to outreach to far place from this health center and tell them the importance of that Hep B*.”

#### Challenges with timely HepB-BD vaccination

The main challenges affecting timely HepB-BD vaccination (within 24 hours of birth) were births outside of public health facilities, misconceptions among HCWs about the timeliness of HepB-BD administration, and the lack of vaccine administration outside of regularly scheduled immunization days. A medical officer in Enugu shared the challenges associated with home births: "*Some areas in the urban slums people who patronize the birth attendants*, *after delivery they don’t get the Hepatitis B [birth dose] because they don’t have it there*. *So*, *the attendant nurses who have the knowledge will always refer them to [health] centres where they can receive [it] but sometimes*, *those women don’t access the vaccine*.”

A medical doctor from Enugu emphasized the importance of TBAs and shared, “*some [pregnant women] end up in the private sector*, *some end up in the TBAs area*, *some don’t even go*, *they delivery at home*. *So for those ones that do not have access to government establishment*, *most don’t hear it*.” A nurse responsible for immunization in Adamawa described, *“the challenges is like the way I said it before if the women did not deliver at the hospital or they are too far from the hospital and during raining season that time and then they doesn’t know about it*.*”* A CHV in Adamawa shared, “*In our Clinic as of now*, *we are giving hepatitis B within 14 days but if the woman delivered at antenatal health care at immunization date*, *we give the injection at birth*.” Similarly, a nurse mentioned the role of immunization days and stated, “*if you don’t deliver at the hospital on that day*, *please bring in your child during the week for the immunization day and even if she delivers at the hospital and is not the day of immunization*, *we tell her to come back*.” Other HCWs echoed similar sentiments relating to the roles of immunization days. Similarly, a doctor from Adamawa shared, “*women who come around to deliver on Wednesdays*, *Tuesdays*, *Fridays*, *Saturdays and the rest of them*? *So usually what you expect that these ones are not*, *they may not really get within the first 24 hours*.*”* Another nurse midwife in Adamawa stated that *“Women are coming for the vaccine though most come late at 2 weeks*, *but we emphasize for women to have it at the clinic after birth*.”

#### Hepatitis B birth dose misconceptions

HCWs agreed that HepB-BD vaccination was important. All of them described that hepatitis B vaccine administration within two weeks of birth is critical, while some noted the importance of administrating HepB-BD within 24 hours of birth. These findings demonstrate a need to expand knowledge about the importance of HepB-BD timeliness. HCWs agreed that there is some knowledge of hepatitis B at the community level, but overall knowledge is limited. Most described the need for improved education, particularly to encourage health facility deliveries rather than home births so HepB-BD can be administered on time. A HCW mentioned, “*We keep encouraging [because] prevention is far better than cure so it is better prevented than waiting for when it comes down with the infection at that time the cost will be high*.” A nurse mentioned confusion around birth dose and described, “*It is important*, *like [the oral polio vaccine] zero dose*, *it has no function*, *but after that zero dose and Hepatitis B one*, *then*, *the immunity will now activate and work very well*.” Another mentioned avoiding immunization of HepB-BD if there are contraindications, a common misconception about HepB-BD, and shared, “*If we notice the baby has no jaundice we give immediately*.” Another misconception about HepB-BD administration was described by a community volunteer who shared, “*in order to avoid vaccine wastage we normally ask patients to come back on that immunization days… I want them to come together so that they will receive it like at a larger number*.”

### Healthcare workers’ recommendations to improve hepatitis B birth dose coverage

We asked healthcare providers to provide recommendations on strategies to improve timely HepB-BD uptake in their facilities. Many suggested logistical strategies, such as ensuring HepB-BD is available within maternity wards. One HCW stated, “*What I will suggest is that hepatitis B vaccine should be available in all the health facilities*, *that is*, *even in the labour room so that as the child is being delivered*, *you give the hepatitis B*, *in that way they will not be missing the birth dose*, *even the private hospital*.” Another individual stated, “*The awareness should be there*, *create more awareness*, *that’s number one*. *Then number two*, *empower the health workers on the need for them to reach out to their clients*. *For instance*, *series of training*, *training resource persons on the need for this*.” Others suggested broader outreach, “*Outreaches*, *that’s always done in churches and schools*, *maybe girls’ schools*, *boys’ schools*, *and then in the marketplaces then awareness*, *then you increase the rate at which we advertise in the radio*.”

Another individual similarly stated, “*educating people by way of maybe*, *you can come and do some dramas for us or training the community*, *educating the community*. *so that they will know or even if you have posters*.” One nurse noted the importance of including TBAs in the education about HepB-BD and shared, “*A lot of pregnant women go for antenatal but not all of them*. *Secondly*, *not all of them go to government centers where those things are supposed to be available*, *some end up in the private sector*, *some end up in the Traditional Birth Attendants area*, *some*, *they deliver at home*. *So*, *what I have come to say the organization need to extend their hands not to restrict the training or awareness to those working in the government establishment*, *if it can be extended to non-governmental areas like private clinics*, *TBAs*, *give them their training*.” Another suggested financial resources would be valuable by stating, “*please employ workers and give us transport*.” Another suggested going to homes to encourage delivering in a health facility and emphasize the importance of coming to the facility for HepB-BD administration.

### Findings from pregnant women participants

We identified the lack of knowledge about HBV, limited understanding of the importance of timely HepB-BD, and access to the vaccine as overarching themes for barriers among pregnant women. The main facilitator to getting a HepB-BD was the high acceptability of HepB-BD if it is offered by HCWs. Women also suggested that HCWs conduct outreach activities to improve HepB-BD uptake and increase HBV awareness within the community, incorporate education about HepB-BD during antenatal care, and provide education about HBV and HepB-BD in the media. Below are some examples of each overarching theme from the interviews and by using the CFIR as outlined in Table 2.

#### Hepatitis B knowledge among pregnant women

Many women reported not knowing much about HBV. However, some had heard of it but remembered a few details about the virus. An individual shared, “*From the little I know is that they say that it’s a deadly disease and it’s contacted by through sexual intercourse and also breastfeeding of baby*.” A few women did know that hepatitis B “*is a disease of the liver*,” and others shared that there is “*an immunization given to kids*. *It’s for health purpose*.” Others also had misconceptions about HBV and one shared, “*I don’t know much*, *I know it’s a disease of newborn babies that affect them when they are born*,” while another stated, “*the little knowledge I know about it is that it is a disease that normally treated in pregnant woman during about 3 to 6 months of pregnancy to avoid some sicknesses after childbirth*.” Others mentioned they had heard of HBV but described, *“it is not in my lineage*” or shared, “*the little I know about it*, *is a disease that kills but for now there is no remedy for it*.” Overall, there was limited knowledge about HBV and misconceptions about disease transmission existed within the community.

#### Hepatitis B birth dose understanding and acceptance

When asked about the HepB-BD, many women mentioned they did not know much about it other than the fact that it is given to children. All women interviewed shared that they would ensure their child would get HepB-BD if their HCWs recommended it. Overall, the women expressed significant trust in their HCWs. One individual shared, “*I just told the midwife that she should attend to me that the way that my baby will be okay*, *that will not have issue on my baby*, *that anything that you know as you are a mother before me that you can take care of my child*, *as in I give everything to you*.” Interestingly even though women did not know much about HBV, they would still take it “*to protect my baby against hepatitis B*.” When asked, all women interviewed felt the birth dose was essential and said they would request it from their HCWs after delivery.

#### Access to hepatitis B birth dose

Women described the importance of messaging to increase awareness and access to HepB-BD, especially in rural areas or those delivering at home. A woman described the importance of reaching all women, “*some people if the[y] give birth at home they don’t use to bring their child for vaccination*, *so the suggestion I will make here is that if it will be possible it is good for health workers like you to go into the community make awareness tell them the danger that is involved if your child doesn’t have the vaccine*.*”* Similarly, another woman expressed the importance of rural outreach. She shared, “*Some people we come but when you have time to come to village square and make the village come out*, *that some people are coming to address you people on hepatitis B*. *If it’s the village square everybody will come and hear what you are saying*.”

#### Recommendations by pregnant women

The pregnant women interviewed overwhelmingly suggested that providing education during antenatal care through health talks could be most effective in improving HepB-BD awareness among women. Many women described how antenatal care was a helpful tool in education and described receiving information on giving birth, baby positioning, signs the body is ready to deliver, and immunizations. A woman said, “*Antenatal day*, *so that’s the best way I suggest that you should come and lecture them about it*.” Others suggested using mass media as a strategy to reach a broader audience. Another said, “*I suggest that the government should try to give all information on hepatitis B on radio and television*, *even on*, *maybe in churches and schools*, *we announce to them about it*.” Others suggested incentives to increase women’s attendance in educational events. A prospective mother shared, “*Some will give bag*, *some will give tissue*, *and some will give soap*. *If you give anything that will*, *even those that will collect bag*. *Whenever they hear that they will get them something*, *[they] will want to come out*.”

Another woman suggested having print materials at the time of education and added that visuals would help with learning, *“that one is included because during that time of education all this flyers now they use to give us example show us all this flyer and also pictures*.*”* A couple of women emphasized the importance of social media and mass media messaging. A woman explained, *“I advise that you put it on social media… like on Facebook*, *and then Instagram*. *You should make sure they always advertise … all these a radio station*, *television station for them for people*, *because*, *not everybody that go online or have phone to browse*, *to go on Facebook*, *and most people even in these rural area*, *they’d have television*, *and when they start seeing this hepatitis B vaccine*.*”* Others also suggested expanding awareness at various places where people go, stating that *“Yes*, *they should create awareness at the markets*, *mosques*, *and churches*.*”*

## Discussion

This qualitative study among HCWs, CHVs, and pregnant women in two Nigerian states demonstrated that healthcare providers in Adamawa and Enugu experienced barriers regarding the accessibility of HepB-BD, including transportation costs for accessing vaccination, staffing shortages within health facilities, and births taking place outside public health facilities (e.g., at home, with TBAs, or in private facilities). In addition, gaps in knowledge among HCWs regarding HBV transmission, misconceptions about the timeliness of HepB-BD, and concerns about vaccination wastage. Facilitators included the cold chain availability in most cases, low stockout of the vaccine, the acknowledgment of the importance of HepB-BD among providers, and high acceptability of HepB-BD among pregnant women. Healthcare providers and pregnant women noted a significant need for enhanced training, sensitization, and awareness on HBV and the importance of timely HepB-BD. While we expected to see significant differences in barriers and facilitators to HepB-BD administration between the two states, findings identified similarities among both states rather than differences.

Pregnant women interviewed were receptive to all services offered and recommended by healthcare providers. Acceptance of HepB-BD if offered by HCWs indicate that vaccine acceptability is high among pregnant women but vaccine access is limited due to the restriction of HepB-BD vaccination to immunization days in most public facilities, the lack of HepB-BD at private health facilities, and the lack of outreach sessions to vaccinate children born at home. Findings also suggest low vaccine stocks due to solar refrigerators. With these findings, HCWs involved in labor and delivery and routine immunization should work collaboratively to ensure HepB-BD is administered on time. HepB-BD should be accessible within delivery wards and beyond regularly scheduled immunization days, not only in public facilities but also in private facilities and among TBAs.

Successful strategies in improving timely HepB-BD vaccination among health facility births include integration of HepB-BD vaccination with essential newborn care, assigning the responsibility of HepB-BD vaccination to the person who delivers the baby, and storing HepB-BD near the delivery ward to reach children at all times and not only during immunization days [10, 24]. In addition, reaching births in private health facilities is important to ensure equity and prevent further infections. Government policies should be updated to provide HepB-BD to private health facilities in exchange for timely reporting of all doses administered at health facilities [10, 24]. While ensuring timely HepB-BD vaccination of children born at home might be challenging, the following documented strategies could be helpful: a) educating caregivers about the importance of timely HepB-BD vaccination so they bring their newborn as soon as possible after birth, integrating HepB-BD administration during postnatal care during outreach sessions, and increasing the capacity of community health workers to administer vaccines including HepB-BD [10, 24]. Birth notification, using village health volunteers was piloted in Lao PDR and resulted in more health facility deliveries and improved HepB-BD coverage [25]. Similarly, linking CHVs to HCWs to track home births led to significant improvements in timely HepB-BD coverage among home births in Kiribati [26].

Pregnant women and healthcare providers interviewed were not widely aware of HBV, and several misconceptions about its transmission, symptoms, and consequences emerged. This finding is consistent with other studies within the African region, highlighting the low knowledge and awareness related to HBV [27–29], and expands upon findings from previous work within Enugu [30, 31]. A unique finding identified the lack of a word for hepatitis B within local languages in Enugu and Adamawa. This has also been reported in other regions of Uganda and is key in addressing misconceptions and enhancing education [29]. Often, HBV was described by symptoms rather than by name. Research in other settings has also found this to be true in other countries as a contributor to disease confusion [32]. Additionally, healthcare providers had misconceptions about when to administer HepB-BD, a misunderstanding that may stem from the Nigeria immunization guidelines, allowing the birth dose to be given up to two weeks of age. Additional misconceptions were related to contraindications to HepB-BD administration and concerns about vaccine wastage. These misconceptions and concerns should be addressed during immunization training for HCWs, as well as updating policies and guidance on HepB-BD administration in Nigeria to follow the recommendations of the WHO Strategic Advisory Group of Experts (SAGE) [7]. Infants should receive the HepB-BD within 24 hours of birth or as soon as possible after birth because delaying vaccination increases the risk of HBV infection by over 8-fold [7]. Additionally, there are no true contraindications to HepB-BD, and trainings for HCWs should emphasize the importance of the vaccine being administered to all infants, including preterm infants [24]. In addition, training and policies should clearly specify that an open multi-dose vial of monovalent hepatitis B vaccine can be used for up to four weeks if the vaccine has not expired, has been kept at the appropriate temperature, the vaccine vial monitor has not reached the discard point, the aseptic technique was used to withdraw all vaccine doses, and the vial was not submerged in water [24].

Healthcare providers and pregnant women expressed the need for education and awareness on HBV, emphasizing the importance of timely HepB-BD. Healthcare providers and pregnant women discussed the significance of providing education throughout the community, both within antenatal care through health talks and beyond through multi-media platforms, such as radio, social media, and community religious leaders. Future efforts should educate and train healthcare providers on hepatitis B disease and the importance of administering timely HepB-BD. Emphasis should be placed on addressing any misconceptions about transmission and contraindications associated with vaccine administration. Several pregnant women recommended incorporating education on hepatitis B and the importance of HepB-BD vaccination during antenatal care visits. Literature has demonstrated that patients with more antenatal care visits have lower maternal, fetal, and neonatal morbidity and mortality than those with fewer visits [33]. Additionally, CHVs, and local leaders can help spread awareness on the importance of timely HepB-BD at the community level and encourage antenatal care attendance to ensure the messaging is widely shared. Recent studies have emphasized the importance of the preconception period as an important opportunity to improve understanding and promote maternal and child health outcomes, particularly in low- and middle-income countries where antenatal care is limited [33]. Education of HCWs, CHVs, and pregnant women led to significant improvement of timely HepB-BD among children born at home in Kiribati [26]. Healthcare providers mentioned challenges related to access to and availability of screening, treatment, and management of HBV. Costs associated with treatment and transportation to referral sites are significant burdens on individuals living with HBV, so much so that forgoing treatment might be preferred or necessary due to these constraints. HCW education and access to affordable HBV diagnostics and treatment should be improved.

While this study provides valuable insight into HepB-BD barriers, facilitators, knowledge, and practices related to hepatitis B virus infection and management, limitations should be noted. While qualitative data collection methods provide rich, in-depth, experiential information, data are limited in external validity (generalizability) and cannot be representative of all experiences related to the HBV knowledge, administration, and uptake of HepB-BD within Nigeria. Additionally, some bias may be present in the selection of respondents as some respondents may have been different than those who did not volunteer to participate. However, the number of respondents approached to participate was large in order to capture a more broad and representative cross section of the population and diversify responses for better generalizability. Additionally, saturation of data was reached for both healthcare workers and pregnant women in this study to mitigate this limitation.

Nigeria has one of the highest burdens of hepatitis B virus infections globally and the highest burden among children under five years of age [9]. To reach the elimination goals for viral hepatitis, it is crucial to improve timely HepB-BD coverage by expanding access to HepB-BD beyond regularly scheduled immunization days, ensuring all births at public and private health facilities receive timely HepB-BD, and conducting outreach to reach children born outside of a health facility [24, 34]. Increasing awareness and education on hepatitis B among HCWs and the community are crucial steps towards improving hepatitis B knowledge, increasing timely HepB-BD coverage, and achieving the elimination of HBV. Furthermore, it is necessary to improve access to treatment, diagnostics, management by reducing cost burdens on individuals living with HBV.

## Supporting information

S1 Text Health care worker and community health volunteer key informant interview guide. (DOCX)

S2 Text Interview guide for pregnant women. (DOCX)

S1 Data Data files, key informant transcripts. (PDF)
