# Supplementary material for: Barriers and facilitators to hepatitis B birth dose vaccination: Perspectives from healthcare providers and pregnant women accessing antenatal care in Nigeria
Source: PLOS Glob Public Health. 2023 Jun 8;3(6):e0001332. doi: 10.1371/journal.pgph.0001332 (PMC10249797; doi:10.1371/journal.pgph.0001332)
Supplement: S1 Text — (DOCX) [file pgph.0001332.s001.docx]

**S1 Text. Health Care Worker and Community Health Volunteer Key Informant Interview Guide**

Thank you for your interest and time for this interview.  The interview is voluntary, and you can stop or choose not to answer any questions at any time throughout the interview.  There are no right or wrong answers; we just want to get your honest thoughts and opinions. This interview is being recorded for our accuracy because we do not want to miss any of your comments.  No information will be able to be traced back to you. We will keep all material private and will not share with anyone.  This interview will take approximately 30 minutes to complete. Do you have any questions about this interview? Would you like to participate in this interview- please respond yes or no.

**State:** [ ] Enugu [ ] Adamawa

1. What are the biggest challenges for people living with hepatitis B in your community?
2. What are the biggest needs to address hepatitis B in your community?
3. Is there a strong need for the hepatitis B birth dose?

- Why or why not?
- Do others see a need for the hepatitis B birth dose?
- Can you tell me about the current programs or practices for the hepatitis B birth dose in your clinic?

1. Is the hepatitis B birth dose easy to get within 24 hours of birth? Why or why not?
   - - Who can administer the hepatitis B birth dose?
     - What are challenges/barriers to getting the hep B birth dose vaccine?
2. Have you gotten information from participants regarding their experiences with the hepatitis B birth dose?
   - 1. What is the community understanding of the hepatitis B birth dose?
     2. Are people interested in getting the hepatitis B birth dose?
     3. Can you describe what kind of specific information/messaging you have heard?
3. Do you have any ideas or suggestions for improving hepatitis B birth dose vaccination rates?
   - 1. At the community level, health care system level, provider level?
     2. Are there specific strategies for education or awareness that might be most effective?
4. What types of health interventions have been the most successful in your community and why have they been so successful?
5. Is there anything else you think we should know about hepatitis B, sharing information about hepatitis B, or the hepatitis B birth dose?
