# Supplementary material for: Barriers and facilitators to hepatitis B birth dose vaccination: Perspectives from healthcare providers and pregnant women accessing antenatal care in Nigeria
Source: PLOS Glob Public Health. 2023 Jun 8;3(6):e0001332. doi: 10.1371/journal.pgph.0001332 (PMC10249797; doi:10.1371/journal.pgph.0001332)
Supplement: S2 Text — (DOCX) [file pgph.0001332.s002.docx]

**S2 Text. Interview Guide for Pregnant Women**

Thank you for your interest and time for this interview.  The interview is voluntary, and you can stop or choose not to answer any questions at any time throughout the interview.  There are no right or wrong answers; we just want to get your honest thoughts and opinions. This interview is being recorded for our accuracy because we do not want to miss any of your comments.  No information will be able to be traced back to you. We will keep all material private and will not share with anyone.  This interview will take approximately 30 minutes to complete. Do you have any questions about this interview?

**State:** [ ] Enugu [ ] Adamawa

1. What do you know about hepatitis B?
2. What do you know about the hepatitis B birth dose?
   - How do you feel about the hepatitis B birth dose?
   - Have you ever asked for the hepatitis B birth dose before for other children?
3. Do you plan to get the hepatitis B birth dose for your baby?
   - Why or why not?
   - Do you have any feelings of concern? Stress? Excitement? Why?
4. Do you think the hepatitis B birth dose will work to prevent hepatitis B?
   - Why or why not?
5. Do you think you could easily get the hepatitis B birth dose vaccine if you asked for it?
   - Will you ask for it?
   - Why do you say that?
   - Is there anything that might come in the way or challenges you might face related to accessing the birth dose?
6. Do you feel the hepatitis B birth dose is important for you to get for your child? Why or why not?
7. In general, do you think pregnant women in your region get the hepatitis B birth dose for their babies?
   - What gives you that level of confidence (or lack of confidence)? – not sure confidence is the right word based on the parent question to this probe
8. Do you have any suggestions for us if we want to educate people in your community about the hepatitis B birth dose?

- Would you prefer to learn about hepatitis B? Through stories, videos, flyers, other methods?
- How do you prefer to learn about health issues?

1. Is there anything else you think we should know about hepatitis B, sharing information about hepatitis B, or the hepatitis B birth dose?
