# Supplementary material for: Barriers and facilitators to hepatitis B birth dose vaccination: Perspectives from healthcare providers and pregnant women accessing antenatal care in Nigeria
Source: PLOS Glob Public Health. 2023 Jun 8;3(6):e0001332. doi: 10.1371/journal.pgph.0001332 (PMC10249797; doi:10.1371/journal.pgph.0001332)
Supplement: S1 Data — (PDF) [file pgph.0001332.s003.pdf]

Note taking template for Pregnant women KII guide State 2, LGA 2, Facility 1

Demographic information of interviewee

Age:28

Questions

1. What do you know about hepatitis B?
   The way that I understand my own, you know that because I'm not speaking igbo, like when they teach us in the class, like the antenatal day, I do ask people, so my own view is that when they say that that that ehhhh…. *sighs* (Coordinator: Hepatitis B) B is that, is about your health, as in how you take care of yourself for you not to have the disease, you have to keep yourself safe and neat and environment, that's what I know about it (Coordinator: about Hepatitis B) yes

2. What do you know about the hepatitis B birth dose?

   No

   (Coordinator: you've never heard about Hepatitis B Birth dose vaccine)
   I have heard it but I couldn't remember it again
   (Coordinator: okay, good. Now, the pregnancy is this the first one?)
   No, the second one
   (Coordinator: So, when you had the previous pregnancy, did you hear anything about when you come for immunization you gave birth to that child, did you hear anything about Hepatitis B birth dose vaccine)
   Yes
   (Coordinator: What did you hear about it)
   Uhmmmmm, what I know about that is about the health of the baby and the mothers, that's what I know about it.

2a. How do you feel about the hepatitis B birth dose?

Pauses (Coordinator: like when you gave birth to that your first child, you said you heard about the vaccine and what it means is for the health of the child and the mother) Yes (Coordinator: Did you vaccinate that your child) Of course (Coordinator: which health facility did you vaccinate the child, was it here?) No, in Port Harcourt, it's still health centre. I attend my antenatal here, then I went to Port Harcourt the same health centre to put to bed in Port Harcourt. (Coordinator: Okay, what do you feel about the hepatitis B birth dose?) It's okay because when you do that, the child will look healthy you'll not have any communication(complication) of your baby again.

2b. Have you ever asked for the hepatitis B birth dose before for other children?

What I notice, I just told the nurse, the midwife that she should attend to me that the way that my baby will be okay, that will not have issue on my baby, that anything that you know that is a, as you are a mother before me that you can take care of my child, as in I give everything to you, is only God that can control and I want you to take care of this my baby for me, that the one that you know that is the best, do it and he say Yes.

3.Do you plan to get the hepatitis B birth dose for your baby?

pauses (Coordinator: what I mean, will you allow the health workers to vaccinate your child, this baby with hepatitis B vaccine when the child is born) Of course

3a. Why or why not?

Because I want my baby should as I said it earlier because I want my baby should look healthy and be okay. How am I going to worry when the doctors and nurses attend to me in the way that I want it and my baby will be okay, I'll not have any worry, the only thing I'll have a joy.

3b. Do you have any feelings of concern, stress, excitement? Why?

Yes. It's just be like when you want, when you know that you are feeling headache already, as in the headache was just coming and you need something that can kill that it immediately, you can rush to that place immediately for it not to get the disease. That's my own understanding.

4. Do you think the hepatitis B birth dose will work to prevent hepatitis B? (Why or why not?)

Yes.

5. Do you think you could easily get the hepatitis B birth dose vaccine if you asked for it?

If its available in the Hospital, if not available, they will tell that its not available to them, can come another day or they'll directed me to another health centre which I can get it. I can get it in health centre, in any health centre.

5a. Will you ask for it?

I told you that what I did normally, when I come to hospital when I put to bed, I'll tell the nurse should take control, that whatever that you know that is good for my baby – do it.

5b. Why do you say that?

Because, I am the kind of person that things bothering me quick, when I put something in my mind, I will not sleep over it. So, when I want to do something, I know that I want to do it let that one clear on my side, I don't want issue that I'll be running up and down, while maybe the nurse was told me that do this ma that I say No that I don't want, them know more than me. That's why I always give them go ahead, do whatever that you know that it will be good for my baby, I don't doubt doctor or nurse.

5c. Is there anything that might come in the way or challenges you might face related to accessing the birth dose?

Eh maybe the nurse told me that I cannot give my baby, which is I cannot doubt. Anything they say, I'll do it, I'll not doubt them.

6. Do you feel the hepatitis B birth dose is important for you to get for your child? (Why or why not?)

Yes of course. Cos its necessary for my baby.

7. In general, do you think pregnant women in your region get the hepatitis B birth dose for their babies?

Yes, because is only if its available, the nurse will ask them to take it. That's my own belief.

7a. What gives you that level of confidence (or lack of confidence)?

I'm a mother, they too they're a mother, we cannot feel the same thing. Everybody is a different people that they can feel the way they feel, but I feel that anything that is good, I think that another person can support it because what I notice is that if something is not good, they'll not introduce us to us to that it.

8. Do you have any suggestions for us if we want to educate people in your community about the hepatitis B birth dose?

Yes, of course, because you know we human beings we're all different, when you want to introduce something to somebody, you have to explain it to your own level of understanding because they may scared like how am I going to take this, that I am as in when I put to bed the first one, the second one, the third one, I did not give my baby, why are you saying that I should give my baby in this fourth baby now. There's a way that ah madam, you know then, by then we upgrade in our system, many things happening. There's a way that you can encourage them, they can be in the party to. But there are some people they are very stubborn, when you tell them they should do this, they'll never do that. Is what they will outsider. Me whatever they do to me I do come to the hospital and confirm before, can I take this, can I do this, they'll say okay go ahead this one is good for you, this one is not good for you. I don't doubt with doctors or nurse.

8a. Would you prefer to learn about hepatitis B? (Through stories, videos, flyers, other methods)

Okay, like the way that I think I have known you today, I have seen you, I have seen your face, that if I have any, that I want to know more about it, I can come to my doctor or go to my nurse to ask much depth and maybe like facebook part, all those things that I can be able to ask. If it's a real facebook account that I can able to call, if there's any number or anywhere that I can login and check a website, I can do that.

8b. How do you prefer to learn about health issues?

Uhmm, you know, you know there's a way they, some like, it just be like, some like when you want to go to a work, there's a training they'll say that today we want to teach on some health problems or health issues, they'll say that by so so time they will want to announce from so so time today that maybe next week we want to have a seminar that we can teach about health. Some people that have interest, they'll come, some people will not come. So, because if you didn't come close to such you will not know, but when you come close to such you will know much better and it is very .....that you know what they are doing. Eh that's why I say that, like a seminar or training? Is it not the same? Okay seminar or training. Like a hall, like you organize like a meeting that you can teach people around you, that people that are ready to learn from you.

9. Is there anything else you think we should know about hepatitis B, sharing information about hepatitis B, or the hepatitis B birth dose?

Ehhhhhhh, nothing much, nothing much but just that, a lot of people doesn't know about it, if there's anyway like maybe like flier or to do like is it promo, how am I going to said it, for them

to know much better. You know some people the way that they live like we don't care, some people doesn't care, so if there's a way that they will know about it much better, we'll try to bring it up, and they will be still teaching us when we come to antenatal at least let people that doesn't know about it let them know much better, so that its very important.

Note taking template for Health care worker KII guide State 2, LGA 2, Facility 1

Demographic information of interviewee

Age: 50 Years

Sex: Female

Designation: Routine Immunization Focal Person

Questions

1. What are the biggest challenges for people living with hepatitis B in your community?

I think the biggest challenges is how to live with the hepatitis b itself

They need health education, they need support, they need their drug too ehe, that is the challenge they have

2. What are the biggest needs to address hepatitis B in your community?

The biggest need is vaccination, immunization, we need to vaccinate them

3a. Is there a strong need for the hepatitis B birth dose? (Why or why not?)

Yes, there is a strong need

Eh because eh at birth, the children are tender, we suppose to immunize them, and some of them, they may even be exposed child. The mother may even have it and you will not know that the mother is hepatitis positive so is very important we immunize them at birth dose

3b. Do others see a need for the hepatitis B birth dose?

They may see it, I may not speak for them nah. Yes, when we give them health education on the importance of the immunization, they may see the need for it.

4.Can you tell me about the current programs or practices for the hepatitis B birth dose in your clinic?

In my clinic, we do give them hepatitis b vaccine at birth, that is within 24 hours. But if the other people that are coming because those that we delivered here we make sure we give but others that come from outside we do give them up to two weeks, the birth dose.

4a. Is the hepatitis B birth dose easy to get within 24 hours of birth? (Why or why not?)

Yes,

Here in our Health Facility, it is easy to get because we do have it in our solar all the time and apart from that, the cold room for the LGA is within this facility so we can easily get it and give.

4b. Who can administer the hepatitis B birth dose?

I think almost every trained person here can administer it.

Probed for designations: the RI focal person used to give, our officer-in-charge used to give, the midwife used to give, then any trained health worker that is around after the delivery of a baby give the hepatitis b vaccine, any trained person around use to give.

4c. What are challenges/barriers to getting the hep B birth dose vaccine?

Well, the challenges, at times if you run out of stock, the cold chain people may tell us that they don't have that we should wait if they go to State they will bring so at times it used to be like that so whenever they bring it, we give.

Probed for more challenges:

For now, I don't know any other one o.

The period stock-out is occasionally, it doesn't last.

5. Have you gotten information from participants regarding their experiences with the hepatitis B birth dose?

No

My experience is that some mothers may not know the need of taking the vaccine at birth, that is, those that come from outside, they may not know the need but those that deliver here, we do explain to them and give the vaccine but some that come from outside, some people may even stay up to three weeks before coming for BCG, so they don't know the need and we have to health educate them. Some of them don't know the need at birth.

5a. What is the community understanding of the hepatitis B birth dose?

You know all these things depend on awareness, everything depends on awareness. If they are not aware of it, they may not know anything about it, but if they are aware of it then, we can now talk about their understanding.

Their understanding is that they know that it is to prevent the disease which is hepatitis b. They know that the vaccine is to prevent it, I can say that they know that.

5b. Are people interested in getting the hepatitis B birth dose?

Yes, they do come

5c. Can you describe what kind of specific information/messaging you have heard?

I don't know

Probed:

No

6. Do you have any ideas or suggestions for improving hepatitis B birth dose vaccination rates?

Uhmm, yes, what I will suggest is that hepatitis b vaccine should be available in all the health facilities, that is, even in the labour room so that as the child is being delivered, you give the the hepatitis b, in that way they will not be missing the birth dose, even the private hospitals, we supposed to have it available.

6ai. At the community level

Yes, at community level, we need awareness, we need awareness, that is, health educate them and tell them the importance of the vaccine.

6aii. Health care system level

At the ministry, at the agency, what I will suggest is that number 1, there is no staff o, we don't have staff doing……, all these people you are seeing there, they are volunteers and students. We don't have staff, we need staff that will be carrying out all these activities. There is no staff at all, as big as this place is, we have only, we are not up to 7 trained staff here, about 6, others are volunteers. So we need staff that will be carrying out all these activities. That is the major problem that we are having.

6aiii. Provider level

Eh we need eh continuous training

6b. Are there specific strategies for education or awareness that might be most effective?

Yes, you can go into the community, do it as eh announcement, we have town criers, we have eh town announcers with eh this eh megaphone, you can tell them too through that medium.

Probed for more:

I don't know oo.

7. Can you tell me about your experience with the maternal tetanus vaccination?

My experience about that one is that eh, that maternal tetanus dose, in this community, the awareness, that I the live one, that 5 doses, the awareness in this area is very low, is very very low. So we need sensitization and eh, eh community awareness. What they are used to in this area although we use to give them health education about the TT live eh this thing, but what they are used to generally here is once they are pregnant, they take TT1, TT2, that's all. That's what they are used to. The awareness of TT1, TT2, TT3, TT4, TT5 is very very low in this community.

7a. What barriers or challenges have you experienced related to the maternal tetanus vaccination?

No, except this one I told you now that they are not aware of taking it 5 times.

7b. What successes have you experienced, what is working?

Eh, I can say that I myself, since I have been here, I have never witnessed any neonatal eh tetanus eh this thing, I have never witnessed any mother that delivered having tetanus, that's number one, then the vaccines are always available, so I think there is reduction in people that is having the……,I have never witnessed any so that is number one success which means the vaccine is… they are working.

8. What types of health interventions have been the most successful in your community and why have they been so successful?

What are the health interventions? Like eh immunization, antenatal and eh family planning, the most successful. Many of them are successful, I don't know the one I will say is the most successful now, (laughter). Okay let me start from my own point, immunization, yes because all the vaccines are readily available, once they come here they will be immunized and go, so I think it is a success. Ehe! because all these eh child killer diseases is reducing, there is growth reduction in IT, most of them I have never witnessed, I used to hear o with ear. So there are success.

9. Is there anything else you think we should know about hepatitis B, sharing information about hepatitis B, or the hepatitis B birth dose?

No

State 2, LGA 2, Facility 2

Moderator: my name is [name], my colleagues name is [name] we are from AFENET(African Field Epidemiology Network), we are in this health facility [facility name] in [LGA name] to conduct Hepatitis B birth dose assessment and to conduct a Key informant interview for the Officer in Charge of the health facility now we want to start with the KII with you the officer in charge [facility name] in [LGA name] this interview will last for about 30-45 minutes and the questions we ask you are for you to respond in ways that you understand they are not exams questions so feel free anyone you don't understand, may I know so that we could possibly ask it in a way you could understand thank you we don't want you to mention your name while we are discussing, just tell us whatever you feel about the question we are going to ask you. Do you permit us to start the interview?

OIC: Go ahead

Moderator: thank you

OIC: (coughs)

Moderator: Sorry please so may we know your age ma,

OIC: it's forty five

Moderator: what is your designation here

OIC:OIC

Moderator: you are the officer in charge of [facility name] in [LGA name]

OIC: yes

Moderator: thank you ma

OIC: (coughs)

Moderator: sorry ma

Moderator: the first question we want to ask is, What are the biggest challenges for people living with hepatitis B in your community?

OIC: the challenges ?

Moderator: of people living with Hepatitis B

OIC: (coughs) the challenges are that emmm, there is no challenge because we are giving enough vaccines to protect children from death so as a result of that we don't have any challenges

Moderator: okay now what you say is okay but what do you think are the possible challenges of people who already have Hepatitis B face in the community

OIC: (coughs) whenever I eat groundnut it scratches my throat

Moderator: sorry, so people that already have this disease. You know about Hepatitis B

OIC: yes, yes

Moderator: so what are the possible challenges you feel they can have?

OIC: one of the challenges is emmmm when they have facility like this, then it will be good for the facility to have a competent hands then when we get such people either through lab or through appearance then the challenges is that how we refer the people to a higher level for management.

Moderator: so how do they appear you talked about eh that it can either be by during test or

OIC: like pregnant mothers through pregnant mothers you can get it through pregnant mothers

Moderators: you say you can identify them through the way they look, is their anyhow they look that you feel maybe a challenge to them

OIC: the challenge to them is that emm emm sme of them may not have the money to emmm treat themselves, they may not have the money to treat themselves and mmmm even if you tell them to go outside they will be no transportation(clears throat) and availability of the drugs will be there in case if they are referred back when they are referred back for us to continue with what they did, we may not have emmmm drugs for any other thing like that

Moderator: okay, that’s okay, that’s okay. What are the biggest needs to address hepatitis B in your community?

OIC: what is what…?

Moderator: what are the biggest needs, the needs to address hepatitis B in your community

OIC: emmmm its emmmm I think mobilization, we mobilize people and tell them what is, (clears throat) what is existing so when those people, when you mobilize them they can even have the access because some of them you don’t mobilize them they cannot know that heath facility can identify their problem, so mobilization is mostly dead and health workers to mobilize

Moderator: Is there a strong need for the hepatitis B birth dose vaccination, do you think there is a strong need

OIC: Yes,

Moderator: Why?

OIC: because if you check them during birth as we were told during training emmm that emmm from day 1 from birth to fourteen days we give hepatitis B vaccine zero dose so when you get the there at six weeks, ten weeks and fourteen weeks you get them, I don't think they will have it in future time.

Moderator: Do others see a need for the hepatitis B birth dose? Do you think other people see a need for hepatitis B birth dose vaccination

OIC: Yes because of the health education that is going around so people now started knowing that while people that are (ehh) have it, maybe adults or those people that doesn't have the immunization at birth that is have complete dose of immunization some of them have it and when because of mobilization we tell that why these people have is because they were not covered so those people that are covered do not have challenges, those people that will have challenges are those people that did not cover hepatitis B either during birth maybe they are ignorance of that health education help to eliminate those things.

Moderator: Can you tell me about the current programs or practices for the hepatitis B birth dose in your clinic?

OIC: what?

Moderator: Can you tell me about the current programs or practices the things you do, for the hepatitis B birth dose vaccine in your health facility.

OIC: as I said earlier immediately a child is born we now cover that person, that child with hep B Zero.

Moderator: that is what you do in this health facility

OIC: off course and even those people that born somewhere because of the mobilization even they born anywhere or TBA some like that because they know that we are giving vaccines here, the health education is going round they do bring before 2 weeks of birth.

Moderator: Is the hepatitis B birth dose easy to get within 24 hours of birth in your health facility?

OIC: of course

Moderator: why

OIC: we have solar

Moderator: you have solar? okay

OIC we have solar so it's always available here so immediately a child is born you now give it

Moderator: Who can administer the hepatitis B birth dose in this your facility? Who and who can administer it?

Answer: the OIC is there, the focal person RI CHEW is there she is the one that is on training now.

Moderator: What are challenges/barriers to getting the hep B birth dose vaccine? Are there barriers or challenges in getting it in this your health facility?

OIC: the only thing is that transportation, its transportation the solar is already there, then, although we don't have light, because of the energy power, the sun, the energy power we don't have any problem about the storage, only the problem we have is to get it and store. Like Abakpa is our Zonal area for collection

Moderator: that is transporting the vaccine?

OIC: transportation is our major problem and sending the data is also, transportation

Moderator: transportation for bringing the vaccines, the antigens and also transportation for sending the data to the LGA

OIC: yes to the LIO

Moderator: can you expand more on this transportation problem, can you tell us more

OIC: on this transportation

Moderator: hmmmm

OIC: hmmmmm

Moderator: why is it a challenge

OIC: it's a challenge because hmmmmmm if you are not paid and you don't have your own money to go and collect it. It becomes a challenge, because no money to transport yourself, because you I don't have the money and government is not giving me the money, like emmm what do I call it overtime or overhead

Moderator: Organize logistics for bringing the vaccines and taking your data

OIC: yes

Moderator: so it's a challenge

OIC: big challenge

Moderator: Have you gotten information from participants, those women who are coming for ANC, who are coming for immunization, have you gotten information from them regarding their experiences with the hepatitis B birth dose? Have you gotten information during the course of

your interaction with them as they come ANC as the come for immunization as they come child birth have you gotten information from them regarding their experience with the hepatitis B birth dose? Are there any information that you have gotten from them, their views, their beliefs, their perceptions, their reactions, their acceptance or rejection anything you want to tell us?

OIC: no they accept it because many of them when you give them health education and tell them instances, some of them will say ewooo, is this what that killed their brother or killed their son or killed of their children they have seen it somewhere and the believe its maybe witches and wizards and they now go for emm native drugs so is it that this common thing that have treatment that killed many people so those people that come accept it but my view is that some of them do tell their experiences elsewhere not here.

Moderator: What is the community understanding of hepatitis B birth dose?

OIC: community what?

Moderator: community understanding this Ugboezeji now, how do they understand this hepatitis B birth dose vaccination, what can you share with us of their understanding of it

OIC: if a child is covered immediately after birth and during the course of routine immunization there will be low death in children and it will not, maybe even if the child grows up and be in the midst of people that have it, the person will not contract it as those people that do not have it.

Moderator: Are people interested in getting the hepatitis B birth dose in your understanding in this Ugboezeji?

OIC: yes

Moderator: you think people are interested ?

OIC: yes much

Moderator: why do you say so?

OIC: because they have seen the good part of it their children.

Moderator: can you describe what kind of specific information you have got concerning Hepatitis B birth dose vaccination what I mean is can you describe for us some specific information that you have got or some messaging ways of maybe communicating you about Hepatitis B birth dose are they some you want to share with us some ways you have got information concerning it?

OIC: not yet

Moderator: Okay I mean all this information that you have been sharing with us the knowledge you have about it can you give us some specific means through which you got this knowledge

OIC: okay from interaction

Moderator: okay interaction with who

OIC: my clients or the community when we go for outreach then from emmmm….

Moderator: I mean the things you know about Hepatitis B also how did you get to know them Hepatitis B birth dose are there some specific means or ways that you were able to get to know about this things that you are telling us because you have shared a lot of knowledge about it. So may we know the ways you got have this knowledge you are sharing.

OIC: through training, its through training, social media

Moderator: okay training social media interaction with client when you go for outreaches when they come to health facilities, that's okay lets move forward to the next question. Have you do you have any ideas or suggestions that you would want to give to us for improving hepatitis B birth dose vaccination rates are there some suggestions you want to give to us on how Hepatitis B birth dose vaccination rates can be improved so that we look at it and see how it will help?

OIC: hmmmmm

Moderator: are there some things you feel when they are done it will help in increasing Hepatitis B birth dose vaccination rate this are the things you want us to do this this this…?

OIC: what we want you people to do is to give us health workers when we have the health workers we will have remote areas to cover so we would go to those hard to reach area and the people will know more about this, so what we need is mostly is health workers to go inside the interior communities and mobilize

Moderator: and mobilize people?

OIC: and mobilize people only two persons cannot do the whole job

Moderator: you are just only two in this health facility?

OIC: we are only two, the OIC and then RI

Moderator: Okay are there any other things you feel we should do?

OIC: transportation

Moderator: okay so what do you want us to do about it?

OIC: please employ workers and give us transport, jingles those people that can make people to create awareness even vehicle is there, keke if you give we would us mic and go round and tell people

Moderator: so at community level, I want you to break them down you have spoken generally, what do you suggest we can do at community level to increase the hepatitis B birth dose vaccination rate?

Moderator: community level at Ugboezeji level

OIC: outreach and mobilization

Moderator: what about at the healthcare system level like ministry of health, agency, at facility level what are the things you feel we can do? What do you feel we should advice, recommend to the ministry of health to the agency, to the health facility, what are those things you think we should recommend that if those things are done, they will help to improve the hepatitis B birth dose vaccination rate

OIC: hmmmm I still talk on manpower

Moderator: okay The ministry of health the agency should employ more people

OIC: tribble yes then and availability of vaccine although they are trying

Moderator: they are trying but they should do more

OIC: yes they should do more, we don’t lack expect in future but has of now they are supplying us enough

Moderator: what about at the health care provider level at the routine immunization officer the OIC the other health workers not just restricted to ugboezeji, in ugboezeji and other health workers who are the ones giving the services, what do you suggest can be dine at that level that could help to improve the hepatitis B birth dose vaccination rate?

OIC: still health workers provide health workers because what am suffering now other people are suffering even if you don’t go there, we all put hands together and tell ourselves this our problem and that transportation as well we must talk of transportation

Moderator: is there any other thing that can be done at the level if the health care worker that could improve the services of wanting to improve the hepatitis B birth dose vaccination?

OIC: then some of the health facility do not have a suitable place for giving the vaccine like that small place don’t accommodate mothers had it been that you come tomorrow you won’t have a place to work in unless you then we don’t have benches and you can see our table

Moderator: because tomorrow is your immunization days

OIC: we have registers but things like these tables chairs and at times on immunization days I will stand up they will take all my chairs, the seat am sitting and even the one my will be sitting while

I am consulting I will just stand up and the patient will stand up while I give the seats to nursing mothers because they can easily fall down, so benches tables other tools, so they have to expand our place of immunization

Moderator: Are there specific strategies for education or awareness that might be most effective, creating awareness at the community level, at health facility level? Are there some specific strategies for educating them and creating awareness that you feel that might be most effective?

OIC: yes

Moderator; can you share with us please

OIC: posters, posters is there and giving them little little things, cause we believe when mothers come and after educating them, like now I saw this type of thing, face mask you give them they value it so if they can provide something that after being fully immunized, like I could remember 2015 or back when fully immunized you give a mother a net. Thank you for completing the immunization and it also helps to give information to other people after the immunization they will give you net free, government should know something they will be giving pregnant mothers so when they register, this is for you and your newborn baby i.e. the in-utero to sleep, and after when they deliver you give them maybe that one has torn or spoilt, you give them, then after the completion of immunization you give them again in one pregnancy they can get three nets then. At that time the immunization was encouraged and people were coming very well. They need incentives.

Moderator: thank you, Can you tell me about your experience with the maternal tetanus vaccination?

OIC: my experience

Moderator: yes your experience about maternal tetanus now they call it Td

OIC: yes Td

Moderator: when women come for ANC tell us you experience

OIC: all of it?

Moderator: anyone you want to share with us.

OIC: the experience I am having here is that when a mother is registered at 16 weeks we give the first Td after 4weeks we give the second Td and tell the mother and give the mother the date and after 6 months of the 2nd then the mother will come 3rd Td we now tell the mother the new date the forth then we give date the fifth one its now complete Td for the mother and that will help the mother even if the mother goes anywhere where health center is far away or doesn’t know

any health center maybe the person packed in that place and the labor started the person can go anywhere and deliver and they person will not get what Tetanus. Then those people that do not have it my experience is that it if they deliver in a dirty environment and even if they deliver in a clean environment, You know that women this thing is always porous so there is chances of that person contracting it., then we will now educate them please its better for you to have the 5 doses in case you don't know where you will get yourselves in the next pregnancy.

Moderator: so do they comply to have the 5 doses in Ugboezeji

OIC: the educated people are complying, some educated people are complying expect they pack out and go another place, we tell them to continue

Moderator: What barriers or challenges have you experienced related to the maternal tetanus vaccination? Are there some challenges or barriers you have experienced in your health facility concerning the maternal tetanus vaccination

OIC: the only thing is that, the challenge is that some of them do say that after the immunization injecting them they feel pains and we tell that person that it shows that it is potent

Moderator: What successes have you experienced, what is working? What makes you joyful about this vaccination we have been discussing about, are there some success you have recorded or experience what is working for you can you share with us

OIC: things that are working for me is that when they give birth there is no problem even the, the baby will be free and the mother will be free till the baby starts 6 weeks so it's giving me joy.

Moderator: What types of health interventions have been the most successful in your community and why have they been so successful? The health interventions the health strategies that have been successful in this community ugboezeji, and why have they been that successful.

OIC: because they follow our instructions

Moderator: I want you to get the question first of all are there some methods or health intervention that have been successful in ugbezeji are you getting it. Are there some I don't know how else to put it, the strategies the health interventions, are there Are some things you have done to improve the health of the community that have been successful, why have they been successful.

OIC: Td or general

Moderator: general are there some things you have done at the level of the health facilities for the community that you feel were successful and why do you think they were successful, like you told us that some health intervention during the course of our discussion so well want you to share with us are their some intervention that you have conducted or you have done in this

community that were successful that why do you think they were successful We want to learn also

OIC: although when I was posted here 2 years ago, here was not moving at all, so what I did was to conduct free treatment for them to know that government hand of a thing in the community and after doing that people started utilizing this place and those they needed they will all get it here, the ones I can do. I don't know whether you have gotten what....

Moderator: yes, yes

Moderator: that's okay this the last question we want to ask Is there anything else you think we should know about hepatitis B, sharing information about hepatitis B, or the hepatitis B birth dose? Is there anything we didn't talk about that you would want to bring into this discussion let us know feel free to do that

Answer: materials to do the work like cotton wool is not there, they have not provided any cotton

Moderator: okay materials to do the work should also be supplied things like cotton wool

OIC: gloves although they supplied us something like that to us

Moderator: You mean sanitizers

OIC: yes

Moderator: thank you so much for your time we appreciate the responses you have given and we say a very big thank you

State 2, LGA 2, Facility 2

Interviewer: my name is [name] and my collogues name is [name]. we are from the African Field Epidemiology Network. We have come to this health facility [facility name] in [LGA name] to conduct a key informant interview for a pregnant woman, so feel free calm down and respond to the questions we are going to ask you

Respondent: No problem

I: thank you, before we start the questions may we know your age? How old are you

Age: I am 22 years old

I: 23 years

R: 22 years old

I: 22yrs old

R: yes sir

I: What do you know about hepatitis B disease?

R: the little knowledge I know about it is that. it is a disease that normally treated in pregnant woman during about 3 to 6 months of pregnancy to avoid some sicknesses after childbirth

I: What do you know about the hepatitis B birth dose vaccination?

R: I don't have the idea of that one

Interviewer: ok, you don't have idea of it?

Respondent: yes

Interviewer: Have you ever asked for the hepatitis B birth dose before for any other children?

Respondent: I have not

I: Do you plan to get the hepatitis B birth dose vaccine for your baby, this baby that you are carrying now when you give birth to the baby?

Respondent: when I gave birth to the baby if I get the actual information about it I will conduct it on my baby

3a. Why would you want to do it for the baby when you give birth to the baby?

Respondent: to avoid some sicknesses

Interviewer: are there examples of sickness you would want to avoid?

Respondent: like yellow fever, eemmm this running stomach of a thing

I: Do you have any feelings of concern, stress, excitement about this hepatitis B birth dose vaccination?

Respondent: No I don't have

Interviewer: Why don't you have?

Respondent: Hmm I don't have, I don't have a reason for that

Interviewer: Do you think the hepatitis B birth dose vaccination will work to prevent hepatitis B?

Respondent: Yes I think so

Interviewer: why?

Respondent: Because they say that prevention is better than cure

Interviewer: Do you think you could easily get the hepatitis B birth dose vaccine if you asked for it?

Respondent: Yes I think so

Interviewer: why do you think so

Respondent: I don't know the reason

Interviewer: But you feel that if you give birth and come to this health center and ask for it you will get it

Respondent: yes

Interviewer: Will you ask for it when you give birth to this baby?

Respondent: Yes I will

Interviewer: Why do you say that?

Respondent: If I ask the nurses and doctors here they will

Interviewer: What I mean is that you say you will ask for it, why.....

Respondent: Because I told you before that I want to prevent some sickness after my childbirth

Interviewer: Is there anything that might be a challenges that will face you related to accessing the birth dose, is there anything that will be challenge that will make you not access or get this vaccine that we are talking about when you give birth to this baby

Respondent: Something like money. If I ask for it and the money they are asking for is too much, that is the only thing that will stop me or that will be a challenge to that.

Interviewer: Do you think that hepatitis B birth dose is important for you to get for your child?

Respondent: It is important,

Interviewer: why is it

R: in order to prevent some sicknesses

Interviewer: In general, do you think pregnant women in your region Ugbuezeji get the hepatitis B birth dose vaccines for their babies?

Respondent: Yes I think so

Interviewer: why do you think so

R: Because the nurses used to teach us about this hepatitis B and how to avoid it after giving birth to a child

Interviewer: What gives you that level of confidence (or lack of confidence)?

R: Nothing gives me o but in my mind I believe it is good to follow the nurse's order than to do off-head

Interviewer: Do you have any suggestions for us if we want to educate people in your community about the hepatitis B birth dose vaccine?

Respondent: I don't understand

Interviewer: the next question I want to ask is that do you have any suggestions for us if we want to educate people in your community in Ugbuezeji about the birth dose of hepatitis B vaccination, is there any suggestion that you can give us on the way we can do it so that it will be good

Respondent: The only way I know is that number one thing is that you people need to gather some people talk about this hepatitis B. and after talking about it the money involved in the drugs, you people will make it to be cheaper n affordable that is the only way the people will love to participate in it.

Interviewer: Would you prefer to learn about hepatitis B Through stories, videos, flyers, other methods, how do you refer to learn about Hepatitis B

Respondent: I prefer practical aspect of it

Interviewer: like?

Respondent: Like when you come to this health center after teaching us, after teaching the people you people will practice it. As in give immunization and when people see that your drugs are working, they'll start following the order.

Interviewer: in general how do you prefer to learn about health issues, if we want to tell you or teach you about health issues in general how do you prefer to learn it

Respondent: You people will bring the equipment for it so that after teaching us u people will practice it. As in give drugs and we will go home to take it.

Interviewer: are there other ways that you would understand it better, like I have got this ne you have answered now. Is there another way that when they teach you about health issues that is normal easy for you to understand.

Interviewer: Is there anything else you think we should know about hepatitis B, sharing information about hepatitis B, or the hepatitis B birth dose vaccination that we may not mentioned during our discussion is there anything you want us to know, tell us

Respondent: I don't have any other one.

Interviewer: are you sure?

Respondent: I'm sure

Interviewer: you know we have asked a lot of questions is there something you feel is important that you may not have mentioned and also want to tell us about feel free

Respondent: They only uh uh the only thing is to go to the hospitals and let people know about it before childbirth.

Interviewer: okay so we should let pregnant women when they are coming for ANC

Respondent: That's the only suggestion

Respondent: Yes, yes, we educate them on it.

Interviewer: that's okay thank you for your time.

Respondent: You are welcome.

Interviewer: we appreciate

Note taking template for Health care worker KII guide State 2, LGA 2, Facility 3

Demographic information of interviewee

Age: 35

Sex: Male

Designation: Chief Medical Officer

Questions

1. What are the biggest challenges for people living with hepatitis B in your community?

You know the infection is something they don't actually know they have, sometimes they might be thinking they're charmed, sometimes they might be thinking they are charmed by witchcraft in the society, but its only when the doctor has a very good skill that he can send the person to the good investigation, maybe Hepatitis B Surface antigen assay, that is when you can now spot the individual with a positive case. So, on that note; one, ignorance, they are not aware, many are not even aware they have it, then for the people that have it, they still doubt if it is true because they are always influenced by the negative aspect of the practice.

2. What are the biggest needs to address hepatitis B in your community?

Number one is Group Awareness and Treatment

3a. Is there a strong need for the hepatitis B birth dose? (Why or why not?)

Yes, the reason is that if a person is given a birth dose, it will build up enough resistance for the individual towards catching that virus even if he's exposed to the society.

3b. Do others see a need for the hepatitis B birth dose?

Yes, for people that are not so resistant to immunization. You know some families are against immunization due to their faith or maybe its their practice, but for people who acknowledge that immunization is key to good health, they don't have any issue with that.

4.Can you tell me about the current programs or practices for the hepatitis B birth dose in your clinic?

For instance, during antenatal, we do some routine investigations and during those investigations Hepatitis B is one of the cardinal tests. For instance, we do Hepatitis B, we do the VDRL, we do the other relevant investigations but Hepatitis B is a must. So when you screen a woman during pregnancy and discover that she is positive, you will commence her on treatment and then counsel her through that pregnancy and her medications and the need for birth dose.

4a. Is the hepatitis B birth dose easy to get within 24 hours of birth? (Why or why not?)

Yes, it is easy because due to the help of these ehhhh satelite activities, one like SOML activities, they are making it possible to ehhh install freezers for storage of hepatitis B vaccine in all the facilities offering immunization.

4b. Who can administer the hepatitis B birth dose?

The nurse, nurse/midwife

4c. What are challenges/barriers to getting the hep B birth dose vaccine?

For us in Enugu, we don't have much challenges, or any challenge at all because it is always a must that we have that. So, in any functional facility offering immunization, Hepatitis B birth dose is always in stock waiting for any pregnant woman to deliver and its always given within 24hours of birth.

5. Have you gotten information from participants regarding their experiences with the hepatitis B birth dose?

Yes. For instance, some women who deliver at ehhhhh birth attendants, birth clinics maybe in the,you know here is covering some areas in the urban slums. Some people who patronize the the birth attendants, after delivery they don't get the Hepatitis B because they don't have it there. So, some of the attendant nurses who have the knowledge will always refer them to centres where they can receive but sometimes, those women don't access the vaccine. So, as a result, you have some people that will miss that birth dose ehhhh antigen.

5a. What is the community understanding of the hepatitis B birth dose?

Well, recently, the understanding is very good. More especially this time Hepatitis B is always advertised everywhere in radios, in jigles, you know within the ministry of health they carry out campaigns, series of campaigns about Hepatitis B, so the awareness is always on air, and people who attend antenatal, Hepatitis B is a key issue we discuss.

5b. Are people interested in getting the hepatitis B birth dose?

Yes and No. Yes, for the enlightened ones, No for the illiterates.

5c. Can you describe what kind of specific information/messaging you have heard?

Well, during the last Hepatitis B day, ehhhh if you just you're your radio you will hear jingles, need for Hepatitis B ehhh for pregnant mothers to attend antenatal, screen for Hepatitis B and when they deliver the necessity for them to get their birth dose, it's always on air. Then secondly, during antenatal we still like sing it to their ears "please make sur you receive your birth dose for Hepatitis B

6. Do you have any ideas or suggestions for improving hepatitis B birth dose vaccination rates?

Yes. For instance, we need to increase the awareness, like the SMAT survey is going on, the awareness is not too high in the society because they only understand that the only viral infection, we have is HIV and apart from HIV, Covid. Its only now that they are understanding that Hepatitis is even worse than HIV. So, the awareness should be there, create more awareness, that's number one. Then number two, empower the health workers on the need for them to reach out to their clients. For instance, series of training, training resource persons on the need for this.

6ai. At the community level

Okay, at community level, yes, we need to like carry out outreaches, you know immunization has a way of getting to outreach centres apart from the services you get from the hospital, you carry out outreaches, that's always done in churches and schools, maybe girls' schools, boys'

schools, and then in the market places then awareness, then you increase the rate at which we advertise in the radio, yes.

6aii. Health care system level

In the ministry of health, they are doing a wonderful work. I think they have a resource person in charge of Hepatitis B at the Ministry of health. Then for the Agency they should still have, like a synergy, they're working in synergy, they work hand in hand simultaneously like what the agency doesn't have, the ministry will augment, but the ideal thing is that the agency should have a resource person also trained on that, trained on Hepatitis B awareness.

Then, at the health facility level, like here in our own condition we have a resource person who is in charge of that health facility, and that resource person is doing a dual job like a Hepatitis B resource fellow and the immunization resource fellow, because it's always there that you can catch them young, maybe the women then we give them good awareness and the doctors should as well be be conversant with what is happening in the society about the scourge of ehhh Hepatitis B.

6aiii. Provider level

Yes, we can empower them, make sure that the test kits are there then if the test kits are there, then the medication, because if we don't supply them with medications, it's always very dangerous because if you ask them to go into the market and source for antiretroviral which is the key drug, it's always very difficult. So, if there's a way the organization can sponsor the provision of those drugs for Hepatitis B positive patients.

For vaccination, vaccination is very strong here. Its not something that is new, it is there ehhhmmm and functioning. Its only if we have like outreaches, new settlements and we're introducing the settlement for an outreach that we can now empower those going for the outreaches. That is empowering those going for those outreaches, because you know we have so many ehh settlements growing up around this Iji Nike because it's a new settlement and new sites are growing up and we're extending our services to those new sites. Then, you empower the health workers going to to reach out to those people.

6b. Are there specific strategies for education or awareness that might be most effective?

Yes, by use of churches and then schools. We have a good counsellor at the health facility setting and this counsellor can have an outreach, you know cave out time to you know visit the

nearby schools and churches once in a while to educate them on the need for testing for Hepatitis B and its medications.

7. Can you tell me about your experience with the maternal tetanus vaccination?

Yea, you know maternal tetanus, in this part of the country, the women don't receive live vaccine, so what they believe is that whenever a woman conceives, she'll receive the first dose then maybe a month after the second dose. So sometimes, if the woman did not attend a good centre for her antenatal, she may lose those two doses but eventually you see that a woman keeps on receiving those two two doses at each ehhh pregnancy. For instance, if she has just 3 pregnancies its just two times three, six shots, that is sporadically, it's not something that they do routinely. So, I believe that the ideal thing is that health education for our young girls in schools to be receiving normal tetanus eehhhh full vaccine.

7a. What barriers or challenges have you experienced related to the maternal tetanus vaccination?

Uhhhmmm, number one, for the illiterates, they deliver at poor centres, what do I mean by health ehh care services in the villages, that is they don't receive the the vaccine. Secondly, if you screen a woman and she's positive and you instruct her on how to get the drug for the vaccine *sighs* the drug for the disease, she may abscond and eventually land in the birth attendants care and by so doing she would still not receive any medication and the still breastfeed the the child, the baby, and at the end of the day the baby will end up receiving, getting Hepatitis B from the mother due to ignorance.

7b. What successes have you experienced, what is working?

Ehhhmmm, for instance, in the recent past I've discovered a family with almost all the siblings positive from Hepatitis B and the same Hepatitis B killed the parents. So, we as a result of that occurrence mounted a survey like it's on our schedule that any patient tha come sto the clinic will be screened. Any patient that comes, the way we're screening for HIV, we're at the same time screening for Hepatitis B. So, if we get any positive patient, we guide the patient, after counselling, we help the person in purchasing the drug and then make sure that the person is on the medication till the person recovers.

8. What types of health interventions have been the most successful in your community and why have they been so successful? Uhmmmm, you know health intervention is just like give and take. In our society, you know we have so many poor people there. They believe in offering free health services, so if we have an outreach, we do all these tests free of charge. Then for people that are positive, we counsel them and then commence them on our medications, maybe help them in providing the drugs though they will pay. Cos if you allow them to go and buy it, due to the fact that some of those medications are antiretroviral, they may not end up buying. They will just go and resort to herbs, herbal intervention, that kind of it.

9. Is there anything else you think we should know about hepatitis B, sharing information about hepatitis B, or the hepatitis B birth dose?

Well, the only thing you should know is that Hepatitis per say, though its treatable but you still need to be like, be consistent. If it is possible you will help us, as in help in providing for the test kits, and if there's any means of getting the drugs, because for us here ehh the CCCRN helped us in providing the test kit for HIV and their medications. So, if Hepatitis B can run concurrently with, like that of Hepatitis B *sighs* like that of HIV, it would be better so that if you detect somebody, you give the person drugs free.

For birth dose, I don't think, we're having much challenges now because the awareness is everywhere. Even people living with the positive thing, their babies are not even getting the virus because they are on medication. If we do, their panel test, you'll see that even though they're still positive, so they can't transmit it to their babies.

Yes, you'll empower us to strengthen our health education unit so that we can reach out to people still in there that have not heard anything about Hepatitis B

Note taking template for Pregnant women KII guide State 2, LGA 2, Facility 3

Demographic information of interviewee

Age: 27years

Questions

1. What do you know about hepatitis B?

From the little one I know is that they say that it's a deadly disease and it's contacted by through sexual intercourse and also breastfeeding of baby.

2. What do you know about the hepatitis B birth dose?

Birth dose vaccination. Ehhhh, what I understand there is that that vaccination they used to give them, help them to prevent the Hepatitis B, so that it will not cause harm to our children.

2a. How do you feel about the hepatitis B birth dose?

Well, the birth dose. I feel that whenever my kids take it, they are protected against the diseases.

2b. Have you ever asked for the hepatitis B birth dose before for other children?

Uhhhhmmmmm, No oh, it's ehhhh this my second son they inject it because that one, that Hepatitis B is not out from 2017 that I gave birth to my first daughter so I knew about it from 2019 that I gave birth to my second son and it was given to him even the one they call meningitis

3.Do you plan to get the hepatitis B birth dose for your baby?

Yeah, I'll give it to him because I heard that its protected them from that disease.

3a. Why or why not?

Why I want to give it is that to protect him or she against that disease so that He or she will not contact it.

3b. Do you have any feelings of concern, stress, excitement? Why?

No oh, I don't have because I have tested myself and I don't say that I am contacted to it, so I feel free that my child will not contact it.

4. Do you think the hepatitis B birth dose will work to prevent hepatitis B? (Why or why not?)

Uhmmm, I think that it will help because like when we gave birth, they advised us to protect eh our children this immunization of a thing they say that it protects from those diseases so that our children will not contact it. So, I strongly believe that by taking the injection, the vaccine, it will help them not to contact.

5. Do you think you could easily get the hepatitis B birth dose vaccine if you asked for it?

Hmh, I think, because it is now common in every hospital.

5a. Will you ask for it?

Yes of course, but there is a stages of that, its not the first dose that you'll protect your baby.

5b. Why do you say that?

The reason I have said it before, to protect them against the disease.

5c. Is there anything that might come in the way or challenges you might face related to accessing the birth dose?

sighs nothing

6. Do you feel the hepatitis B birth dose is important for you to get for your child? (Why or why not?)

Hmmmm, the same question and the same answer I suppose. Why is that to prevent them from getting the disease. I feel that it is important as they have taught us.

7. In general, do you think pregnant women in your region get the hepatitis B birth dose for their babies?

Heaa, I won't say yes because I'm not like them. Everybody has his own understanding, you understand? From my interaction with them today because it's today that I saw them, so, yea even the ones in the community, my friends yea, the ones I know I believe that they'll get it for their babies.

7a. What gives you that level of confidence (or lack of confidence)?

Yea, *laughs* as they said, because they're my friends and I knew them, we chat, we discussed, I believe that its important for them to give it to their baby when they give birth.

8. Do you have any suggestions for us if we want to educate people in your community about the hepatitis B birth dose?

Uhhhh, its good sha because not everybody. Okay, well the little one I know is that like these Wednesdays they used to Have this their thing. No this, I forgot, Antenatal day, so that's the best way I suggest that you should come and lecture them about it because there's no process for you guys to go and teach about.

8a. Would you prefer to learn about hepatitis B? (Through stories, videos, flyers, other methods)

Hmmmm, I prefer teaching, video is okay too. I don't like stories because with stories, its not what you say that another person will say. I prefer the professionals to teach me about.

8b. How do you prefer to learn about health issues?

Ehhhh, how I prefer is that like now I'm here in the hospital, I prefer that if I want to learn anything about health I'll go to the hospital, consult my doctor or the nurses.

9. Is there anything else you think we should know about hepatitis B, sharing information about hepatitis B, or the hepatitis B birth dose?

sighs I don't think, its like you have said it all.

Note taking template for Health care worker KII guide State 2, LGA 2, Facility 7

Demographic information of interviewee

Age: 28years

Sex: Female

Designation: Community Volunteer Health worker

Questions

1. What are the biggest challenges for people living with hepatitis B in your community?

We don't normally have eh this thing, we don't normally have ehhh as in have eh Hepatitis B patients here, so…

2. What are the biggest needs to address hepatitis B in your community?

The thing is that, you people should provide us with equipment like something that we use to be conducting as in to test them, you understand, yes. As in the strip for the we use and do the lab to know whether they….

3a. Is there a strong need for the hepatitis B birth dose? (Why or why not?)

Yes, you know some people will have eh Hepatitis, that Hepatitis but they will not know that they have it, but if you test them, the result will show whether they have it or not.

For prevention

3b. Do others see a need for the hepatitis B birth dose?

Yes.

4.Can you tell me about the current programs or practices for the hepatitis B birth dose in your clinic?

Repeat the question again. They used to come and take the injection as in the immunization. They give them as in the first ehhmmm after birth, so they give them the injection

4a. Is the hepatitis B birth dose easy to get within 24 hours of birth? (Why or why not?)

Yes, because we have eh this thing that we are storing vaccines so eeh every time of any day the vaccine is with us here.

4b. Who can administer the hepatitis B birth dose?

The health workers as in I mean the nurses that is working here.

4c. What are challenges/barriers to getting the hep B birth dose vaccine?

There is no any challenge

5. Have you gotten information from participants regarding their experiences with the hepatitis B birth dose?

No,Yes. Some people came, you know the hepatitis B vaccine ehen they say that there is a time it will be and the child will not take again because the other this thing, the other immunization that have it. So, the say that after birth if they did not get the Hepatitis B vaccine within like one month to two months that they are no more going to take it that its only BCG that they give them then the rest will be given them in that Pentavarous vaccine

5a. What is the community understanding of the hepatitis B birth dose?

What is there? Okay, they see it as a good thing, yes. For giving their children Hepatitis B vaccine.

5b. Are people interested in getting the hepatitis B birth dose?

Yes.

5c. Can you describe what kind of specific information/messaging you have heard?

Messages, nothing sha, they said that it's good, that its good, nothing like that.

6. Do you have any ideas or suggestions for improving hepatitis B birth dose vaccination rates?

Yes,

6ai. At the community level

okay by announcing as in going from inside villages and streets to announce it that everybody that gave birth should come and take Hepatitis B vaccine that its very very good for the health of the children

6aii. Health care system level

To make sure that they are providing the vaccine, that the vaccine is ready. That each time that parents bring their children come that there will be vaccine to give to them.

6aiii. Provider level

That the health worker be punctual to work, each time, they'll have to be at the right place, like today in the health centre. So, that if the people come, they'll be able to meet somebody, a nurse that will attend to them.

6b. Are there specific strategies for education or awareness that might be most effective?

You mobilize it, to mobilizing it. To mobilize, going using this eh thing to mobilize, going to inside villages, communities, streets, announcing there to come and take Hepatitis vaccine.

7. Can you tell me about your experience with the maternal tetanus vaccination?

It is good to take your tetanus vaccine

7a. What barriers or challenges have you experienced related to the maternal tetanus vaccination?

Some people did not used to come to antenatal till they get to nine months. That day they will come to deliver is the day they will come to register for antenatal by that time you'll find out that they are in labor. So, eventually, they will not take that Tetanus vaccine up to that time.

7b. What successes have you experienced, what is working?

Yes, things are working well. All I know is that that ehh tetanus vaccine is good and Hepatitis B vaccine is good so everybody that give birth should come and take eh that eh Hepatitis vaccine and those the pregnant women for that tetenus. Because I have seen somebody that is giving birth and in that process she start having convulsion, that I learnt that its because they did not take that eh tetanus vaccine. So, that is it, its good to take that drug.

8. What types of health interventions have been the most successful in your community and why have they been so successful?

Eh, they are doing good antenatal days and immunization days, they used to teach some the eh the importance that you give your child ehh immunization like that Hepatitis, BCG, hepatitis B vaccine and others. So, they're doing good by teaching.

9. Is there anything else you think we should know about hepatitis B, sharing information about hepatitis B, or the hepatitis B birth dose?

You have said everything all.

State 2, LGA 2, Facility 7

Interviewer: We want to conduct a Key informant interview for pregnant women in [facility name] in [LGA name], Please may we know your age? What is your age, how old are you

Respondent: 31 years old

I: 31 years old okay, What do you know about hepatitis B, the disease called Hepatitis B what do you know about it?

R: actually I don't know much, I know it's a disease of new born babies that affect them when they are born

I: What do you know about the hepatitis B birth dose vaccination?

R: no, I don't have an idea

I: no idea, How do you feel about the hepatitis B birth dose?

R: how I feel

I: yes about it

R: I feel it is a dangerous disease that need to be taken care of.

I: Have you ever asked for the hepatitis B birth dose vaccination before for other children, this hepatitis B vaccination have you ever asked for it for the other children you have given birth to, have you ever asked for it?

Answer: yes,

I: where did you ask for it

R: at the hospital

I: which hospital?

R: Chukwu asoka hospital

I: Do you plan to get the hepatitis B birth dose for your baby this baby that you are carrying now do you plan to get the hepatitis B birth dose vaccination for this baby when you give birth to him or her?

R: yes

I: Why do you say that why do you plan

R: well actually I plan because prevention is better than cure, I want to prevent it before it affect my baby as a mother that is the primary thing I have to do for him or her

I: Do you have any feelings of concern or stress or excitement about the hepatitis B birth dose vaccination? Do you have any concern or any stress?

R: my concern is that as government has started doing the work of it, they should take it more serious to avoid spreading of the disease.

I: Do you think the hepatitis B birth dose vaccination will work to prevent hepatitis B disease? Do you think that vaccination will work to prevent the disease?

R: I think it will work.

I: why do you think so

R: why I think so for the fact of making effort to prevent it, so government will not just bring what that cannot stop it. So What they bring I think it's for the betterment.

I: Do you think you could easily get the hepatitis B birth dose vaccine if you asked for it? Do you think you can easily get it if you ask for it

R: I don't think so

I: if you come to the hospital to ask for it, when you give birth to this your baby do you think that they can easily provide it for you? Assuming you give birth to this baby and you come to this hospital and say you want Hepatitis B birth dose vaccine for this your baby let them give it to your baby, do you think that can easily be done for you by the health workers?

I: do you understand the question

R: yes I understand but

I: my question is assuming you give birth to this baby.....

R: and they do not give him or her injection immediately....

I: nmmmm nmmmm the question is do you think that is if you give birth to this baby that you can easily get that baby to be vaccinated?

R: yes

I: why do you say it?

R: well I say yes because I think the vaccine has been in the hospital and when I ask of it they will give me

I: Will you ask for that vaccine when you give birth to this baby

R: yes

I: Why do you say that that you will ask for it when you give birth to this baby? What is your reason?

R: number one reason of asking for it has been the right of that my baby to be injected and second one is that I have been informed of the vaccine so if I did not ask for it then am denying that my child

I: Is there anything that might be a challenges you might have or face related to accessing this hepatitis B birth dose vaccine? Is there anything that might be a challenge?

R: for me to get it

I: yes, is there anything that may come in the way or challenges you might face related to accessing the birth dose vaccination, is there anything that can prevent you from getting it, when you give birth to this child is there anything that can prevent you both at your level, the level of the hospital, is there anything that can prevent you at the level of the community is there anything that can be of a challenge?

R: the only thing that can be of challenge is scarcity of it, if they say it is not here now that is the only thing that can a challenge

I: Do you feel the hepatitis B birth dose vaccine is important for you to get for your child?

R: Yes

I: why?

R: It is important for his or her health for his or her health living.

I: In general, do you think pregnant women in your locality region in Nchatancha get the hepatitis B birth dose for their babies? Generally do you think that women in Nchatacha community will bring their babies for hepatitis B birth dose vaccine do you think so?

R: I don't think that all do.

I: you don't think that all do? Okay why do you say so, what gives you that confidence to say that?

R: why I say so is that you that some pregnant women they take some things unserious. So I am not thinking that everybody will like to come and receive it as

I: but some

R: some ma8. Do maybe 50%

I: do you have any suggestions for us if we want to educate people in Nchatacha, pregnant women in Nchatancha about the hepatitis B birth dose, do you have any suggestion on how we

can educate them so that they will understand it is there any suggestion you would like to give us on the method we can use?

R: the only suggestion I have is that calling and educating them on it on how important it is. How safe it will make their baby to be.

I: how would you want us to do that education? which method?

R: calling them

I: okay calling them

R: you put information across so that all them will come all the pregnant women will come.

I: how Would you prefer to learn about hepatitis B, if somebody wants to give options on how you would prefer to learn about this hepatitis B? how will you prefer it which method to learn?

R: is there any other method?

I: like through stories, videos, flyers so which method works best for you that will make you understand it if you want to learn about hepatitis B

R: reading

I: How do you prefer to learn about health issues generally?

Answer: reading as well.

I: reading as well okay. Is there anything else you think we should know about hepatitis B, sharing information about hepatitis B, or the hepatitis B birth dose that you would like to share with us that we may not have talked about during our discussion with you?

R: No

I: no?

R: no

I:s thank you so much for your time

State 2, LGA 2, Facility 8

INTERVIEW TRANSCRIPT

Interviewer: Good afternoon ma. We want to conduct key informant interview for the midwives in [facility name] in [LGA name]

Respondent: Yes

I: Please may we know your age? How old are you?

R: I'm fifty

Moderator: Fifty. Okay. What is your designation.

R: I'm Deputy director community health

I: And you're the midwife that delivers children here?

R: Yes

I: What are the biggest challenges for people living with Hepatitis B in this community. What are the biggest challenges, the things you think are the biggest challenges for the people that have Hepatitis B disease in this community?

R: you know We don't treat them here, we normally send them for-to the bigger health facility like eh Annunciation or UNTH because when they go for test and they know that they have Hepatitis B, we normally tell them that hepatitis B is a sexually transmitted disease. That their partners can get it. That their partner supposed to go for test. So after the test, if the partner is negative, we now tell them that they will go and have hepatitis B--that the partner will go and have Hepatitis B injection and we don't give that one for adults. We give that one of children, newborn here. They will now go to Annunciation, they will go there, they will give them.

I: So, are there some challenges you feel they face?

R: Some of them doesn't go after telling the person everything. Some say they don't have money to have the injection--the partner that is negative. I will tell the person that you can get it from your husband if it's the woman that is negative. Some will say they don't have money to continue for the injection. Some do go and have the vaccine there.

I: Okay. What are the biggest needs to address Hepatitis B in your community? What are the biggest needs to address--what are the things that are needed to address this Hepatitis B disease in your community?

R: If you can come and eh give them lecture about it. Some of them doesn't even know about Hepatitis B. Some know it through us health workers when we give antenatal mothers health talk about Hepatitis B or during the immunization. But I know most of them that do not come for antenatal or immunization, some may have the disease and they don't know about it. But if you people can come and give them training about it, meet the community leaders or the women, they will know about it.

I: Is there a strong need for Hepatitis B birth dose vaccination in your community? Is there a strong need?

R: Yes

I: Why?

R: To protect the newborn from having that disease.

I: Do others see the need for Hepatitis B birth dose in this community? Do you think other people, other women see the need?

R: They need it. they see it but some of them doesn't deliver here. Some deliver to TBAs and when they deliver them, it is only those people that maybe came for antenatal here, that will come back for the hepatitis B immediately. But those people that have not heard about it will stay till on the immunization day, they will come. And I normally tell them that after two weeks, we cannot give you again because that hepatitis is included in pentavalent vaccine.

I: Can you tell us about the current programs or practices for Hepatitis B birth dose in your clinic? What are the things you do, the programs, the practices you do about this hepatitis B birth dose in your health facility?

R: This is, when somebody put to bed, before 24hours we give the child hepatitis B

I: Is that what you practice here?

R: Yes! And on immunization day, we give immunization every Wednesday, we normally give them.

I: Is the Hepatitis B birth dose easy to get within 24hours of birth?

R: Yes!

I: Why?

R: It's always available, we have solar fridge here. so the vaccine is always there in the fridge and somebody is always there.

I: Who are the people that can administer Hepatitis B birth dose in this your health facility?

R: I am the and my immunization focal person.

I: What are challenges or barriers to getting Hepatitis B birth dose vaccine? What are the challenges or barriers you have?

R: We don't have any barrier. We normally go to LIO and they normally give us the cold chain they normally give us. The only thing, we transport yourself. They don't give us money for collection of vaccine or anything. They only ask you not to take any 5 naira from any woman. And like this, em, during dry season, solar fridge can stop working, we normally call somebody. Pay the person, collect ladder and clean the panel. Because if you don't clean it, the vaccine inside the fridge will just damage.

I: Have you gotten any information from clients, pregnant women or people who are coming to your health facility regarding their experience with your Hepatitis B birth dose vaccination? have you gotten any information from them that you want to share to us? What do they say about hepatis B? these women that come for vaccination, these women give birth in your health facility?

R: They don't say anything. The vaccine is just okay for the children. they don't have any adverse event following that immunization.

I: What is the community understanding of the Hepatitis B birth dose? This community where we are, Onu-Ogba community, what is their understanding of hepatitis B birth dose in your own assessment, what do you think is their understanding of this Hepatitis B birth dose?

R: Their understanding is that, they know that, we educate them that this Hepatitis B is infectious if the child did not have the vaccine as soon as possible. so that is what I understand.

I: Do you think people are interested in getting Hepatitis B birth dose in this community?

R: Yes.

I: Okay. Can you describe what kind of specific information or messaging you have heard concerning this Hepatitis B birth dose? Can you describe the specific information or messaging YOU have heard?

R: The message I have heard about is that, it increases child's immunity and it protects the child not having the disease, maybe from the mother or from any other person.

I: Do you have any ideas or suggestions for improving Hepatitis B birth dose vaccination rate that you want to share with us? The ideas or suggestions you want to…

R: It's only educating people by way of maybe, you can come and do some dramas for us or training the community, educating the community. so that they will know Or even if you have posters, we can even put it

I: So at community level…

R: Yes

I: What do you say about community level, what can be done?

R: Educating them.

I: Okay. What about at the level of the health care system? Like the, at the level of the ministry of health, the agency, the health facility, what do you think can be done to increase the rate?

R: Just what we need is, we need more workers. We're understaffed. One person will do this, do this, if you're there in the delivery room maybe on immunization day you'll be on the delivery room, you'll be working for immunization, you'll be treating patients. We don't have enough workers at Enugu East here.

I: What about at the level of the health care worker? At the level of the healthcare provider, you people, what do you think can be done at your level to help increase Hepatitis B birth dose vaccination rate in this community?

R: If you people can help us in transporting it. It can help us. Like when I start job at Ezielo, that time, we have MPI bus, that bus normally go to the LGA collect vaccine. On the day, if you want to, the day we are going to give immunization the bus will just carry our drug; just like somebody that is selling bread. You come out with your Goi'style you put your vaccine. You show the person the requisition, they'll give you. After, at the end of the day, the person will come for the data. But here, we don't have such thing. We normally go on our own. For a whole day you'll be at Abakpa for collection. Because you have so many things to write there. You leave the facility open, if there is somebody that delivers, if I'm not there to give that 24hours…So if you can help us and let them know that we are understaffed, though we have been telling them. If they can employ people, if will help us in this system. So that when somebody delivers, immediately, after some hours, you will give the child that vaccine.

I: Okay. Are there some specific strategies for education or awareness that might be the most effective?

R: Even that drama is normally educative and simple to understand because when you do drama somebody may be laughing but maybe tomorrow, the person can think out something from that thing.

I: Okay. Can you tell us about your experience with maternal tetanus vaccination?

R: Maternal tetanus vaccination, this Td Tetanus Diphtheria* We normally give women at end of one month and give after one month. But there is another one called LIFER. We normally give that one five times but not in one pregnancy. That five times may be by three years the woman will cover it. We'll issue the woman with a card, that LIFER means if you finish that five times you will not have that immunization again for life. But that two times is what most of them are having. We give them at the end of four months and after one month we give another one.

I: What are barriers or challenges you have experienced related to maternal tetanus vaccination?

R: Some of them do not start their antenatal clinic on time. Some will come even by seven months, some will come by nine months. So when you give that person that vaccine, you know that the baby have that vaccine through--from the mother. If the woman did not take that vaccine on time, the child will not have that vaccine and the woman will deliver. That is, some of them, I've had experience, a woman umbilical cord, a child have umbilical cord that cannot heal. They said that, one doctor was saying that it's just because of the woman did not have Tetanus Diphtheria injection on time.

I: What successes have you experienced? What is working for you here? Can we know the successes that you've experienced, what works that you want to let us know?

R: What I normally tell women about that Tetanus Diphtheria is that they supposed to have it on time so that their baby will have it and it will work for their baby. Because that vaccine is for the baby and for the mother. If the mother does not have it on time, the baby will not have it.

I: So do you experience success in those…

R: Yes! Yes.

I: Okay, how? Can we know how?

R: Just about that umbilical cord, when we have that baby, we use this Chlorhexidine gel and the whole thing will fall out as at when due and the baby will be okay.

I: What types of health interventions have been the most successful in your community? And why have they been so successful? The health interventions that have been successful in this community and why do you think they have been so successful?

R: Well, health interventions, by the time, before I came to this clinic, this clinic was not functioning. No, even immunization, we were giving three persons in one month. So, but now, we know there is changes.

I: So why, why, how do you….

R: I normally stay from morning till night every day. If I came to work, I will not go until last. And when I'm going I know another person is there. So we normally stay that 24 ho…I give them that 24hours service. It helps people to be coming and going.

I: Is there anything else you think we should know about Hepatitis B, sharing information about Hepatitis B or the Hepatitis B birth dose vaccination that we may not have mentioned…

R: No

I: ...In the course of this discussion, you want us to know, feel free to share with us.

R: No oh, you people know almost everything about Hepatitis B

I: No, what I mean is there anything we didn’t touch?

R: No

I: ...Bring up?

R: No.

I: Thank you so much for your time. Thank you for your responses, we really appreciate. God bless you ma.

R: Thank you.

State 2, LGA 2, Facility 8

Interviewer: Good Morning all, [LGA name] team we want to conduct key informant interview for a pregnant woman at [facility name] in [LGA name], so may we know your age. How old are you?

Respondent: I am 27, 27 years old

I: 27 years old, Ok. What do you know about hepatitis B?

R: all I know if that Hepatitis B is a disease of the liver, that's the main thing I know about Hepatitis B.

I: do you know about Hepatitis B birth dose vaccination?

R: Yes, Hepatitis is giving before 2 weeks, from birth to 2 weeks with BCG and OPV

I: OK

R: and is giving 0.5mil

I: how do you about Hepatitis B birth dose vaccination? How do you feel about it.

R: Is good because it protects our children against hepatitis.

I: have you ever asked about Hepatitis B birth dose before for other children outside this one you are pregnant for now.

R: those ones I have giving birth to before?

I: yes the other children you have given birth to before, have you ever asked about Hepatitis B birth dose vaccination before.

R: after the first dose

I: No, when you gave birth, do you ask for the vaccine to be given to your children

R: Yes the OIC in charge told me about it, so no need of asking so I just give my children the vaccine.

I: do you have plan to get Hepatitis B birth dose vaccination for this baby you carrying for now.

R: yes of course

I: why?

R: because I know is very important in other to protect my baby against Hepatitis B.

I: Do you have feeling or concerns or any stress or excitement concerning this Hepatitis B birth dose vaccination? Do you have feeling or concerns or any stress or excitement about it?

R: well I am happy about the Hepatitis B birth dose vaccination because is good.

I: why? Ok, because is good

R: yes. it helps our children.

I: yes. it helps our children? alright, do you think Hepatitis B birth dose vaccine will work toprevent Hepatitis B disease?

R: yes of course

I: why?

R: the reason because that first dose it will your baby the immunity he needs in other to prevent him or her from getting that disease Hepatitis B.

I: OK, do you think that you can easily get Hepatitis B birth dose vaccine if you ask for it? Can yu get it easily?

R: YES, because is already at the facility.

I: so will you ask for it when you have this baby?

R: yes I will ask for it because I know is very important for my baby to take it.

I: why you will ask for it is because you know it is very important?

R: yes

I: and it is good to take it

R: yes off course

I: is there anything that might come in the way or challenges you might face related to assessing the Hepatitis B birth dose vaccine, is there any challenge you might face if you want to give it to your child?

R: No, because the first one I tried I didn't have any challenge, so I don't think there is any challenges.

I: do the feel that the Hepatitis B birth dose is Important for you to get for your child?

R: yes

I: why do feel is important?

R: it prevents my child from getting that disease Hepatitis B,

I: ok

R: because I know that my first baby the one I gave to her will prevent her. so is good.

I: in general do you think that pregnant women in your area, your region, in this community get Hepatitis B birth dose for their babies? In general do you think that they do that?

R: yes, because during this at natal visit the OIC and other health workers they use to teach us about those vaccines not only the Hepatitis B vaccine and other vaccines.

I: what gives you this level of confidence that you just expressed?

R: what gives me the level of confidence that…..

I: what gives you the confidence that in general that people in this area get the vaccine ?

R: the health workers in this facility are doing very well, they are working well, and all the vaccines they give to us is working, that's why I have the confidence.

I: ok, do you have any suggestions for us if we want to educate people in your community about Hepatitis B birth dose vaccination, do you have any suggestion you want to give us if we want to educate them or ways we can go about it?

R: for me o, if you want educate them no problem but the health workers are trying their best teaching us what we need to know so I don't think you need to educate us because all those vaccine they are teaching us the need for it.

I: Ok the health workers are also teaching you people?

R: yes

I: I am also asking is there any other suggestion you want to give as a pregnant mother, are there other things you may want to suggest that if we do this one it may also be good oh as a way it will educate pregnant women in this community, Onu-Ogba

R: no, for me I I don't think so, No

I: how would you prefer to learn about Hepatitis B, how would you prefer to learn it if somebody wants to teach you about Hepatitis B how would you prefer to learn it?

R: if some wants to teach me about Hepatitis B

I: Yes if someone wants to teach you, which strategy do you really understand when somebody teaches you with, which method works for you when somebody wants to teach about Hepatitis B disease:

R: hmmmm, is like if someone wants to teach me about Hepatitis B

I: yes, which one do you understand, which method would help you to understand it very well?

R: well, during that at natal care of before the at natal they used to health educate us and I think that one is more important so I think that one is better because during that time they will ask you question if

you have any problem and if you have any problem then you will throw your question and she will now treat it.

I: ok, what about through stories, videos, flyers or other methods, do you?

R: that one is included because during that time of education all this flyers now they use to give us example show us all this flyer and also pictures.

I: ok, how do you prefer to learn about things about this issues generally, how do you prefer to learn it about things about health?

R: how I prefer to learn about the health generally:

I: yes,

R: hmmmmm, through videos, through community mobilization, all this, through flyers like this now and mmm, what do they call it? Hmmmmm hmmmmm Trainings, go to trainings about that particular disease or assuming they wanted to introduce a new vaccine you go to training about that one, I think that one is good.

I: is there anything you think we should know about Hepatitis B sharing information about Hepatitis B birth dose vaccine we did not mention, is there anything you want to talk to us about that w did not mention in this discussion that you feel want to contribute.

R: No

I: thank you so much for your time

R: you are welcome

I: we really appreciate, God bless you

R: thank you and you too.

State 1, LGA 2, Facility 3

Introduction

Good morning, Sir, I am [name], and in my team is [name], we are from CDC-AFENET on a baseline assessment of Hepatitis B Birth Dose.

As part of the assessment, we are going to conduct an interview with you, and there are few questions we will ask you, but I will start by asking how old are you?

I am 38years old.

What is your designation?

I am a Community Health Worker, JCHEW.

In this community, what are the biggest challenges with people living with Hep B?

We experience challenges like transporting to know their status regarding Hep B and the drugs and is rampant.

Is that all? Yes

what are the biggest needs to address hepatitis B in your community?

To include community and religious leaders to advocacy. To advocate to leaders about hepatitis B vaccine effects, and to overcome all these problems.

Is there a strong need for hepatitis B birth dose?

Yes, because Hep B dose at birth is helping us as you can see these young men, you can get much hepatitis b in them as in adult people. So when it is given at birth it is preventive more.

Do others see the need for Hep B birth dose?

Yes, Ahhh…. others see a need about hepatitis birth dose as very important.

Can you tell me about the current programmes and practices for hepatitis B birth dose in this clinic?

Yes, the current programme is routine immunizations. at birth, we give them the Hep B BD at the clinic. Do you give them at fixed sessions or outreach? Yes, we can do it at fixed session or outreach depending on the birth of the child if the baby is still within 14days,

Is the vaccine easy to get within 24hours of birth?

Yes, it is easy,

Why did you say that?

because the LGA is supplying the vaccines and we have the cold chain to maintain the temperature to get the child vaccinated

Who can administer the He B BD?

The Health Worker who is responsible for immunization. Is that all? Sometimes the manager can do it and the assistant if available.

what do you think are the challenges/barriers of getting the Hep B birth dose?

The challenge is when the vaccine is out of stock. That is the only challenge.

From the people that have been given the hepatitis b birth dose, have you gotten any information from participants regarding their experiences regarding hepatitis B birth dose?

No Sir.

what is the community understanding of the Hep B birth dose?

Some people know it when they come, they caregivers, we educate them about it. So, we let them know the importance of Hep B BD.

Are interested in getting it?

Yes, people visiting us or when we go out for outreaches, they are very interested about it.

Ok, can you describe what type of specific information or message you have heard from them?

Yes, some people describe the hepatitis b vaccine as preventable when they get the information. Some when given to their children see it as having no problems. They are asking for more doses if available.

Do you have any ideas or suggestions for improving Hep B BD vaccination rates?

Maybe we get the adult vaccine. Some people ask for adult dose.

At least let the window be at least two months or 30 days instead of 1 days. Transport is also a challenge and we are getting defaulterswho are not able to come within the 14 days.

How do you think we can overcome the transport challenge?

To increase outreache services.

Community level

Effective outreach to every settlement

What of at Health care system level?

To mobilize the community and the health workers to know about all the hepatitis doses.

What of the provider level?

The provider level has to be very active and committed to the services rendering.

Are there any specific strategies for education or awareness that might be effective?

Yes, creating awareness and training of health care workers. Training odf health workers about hepatitis. Training to further their education

Can you tell tells us much about Hep B BD, can you me your experience with maternal tetanus vaccination?

Yes, maternal tetanus vaccination, is improving and geting healthy mothers, childbearing mothers to prevent them from tetanus before giving birth.

What your experience related to maternal tetanus vaccination?

Mothers are not getting the vaccine consistently up to five, they get one or two and defaults, this is my experience.

What successes have you experienced about maternal tetanus vaccine?

When we get the mothers, there is reduction of maternal to child transmission of tetanus

What type of health interventions have been most successful in your community, and why have they been successful?

Programmes like the Seasonal Malaria Prevention, Immunization, have been successful. Is that's all? Yes sir,

Is there anything else you think we should know about Hep B, sharing information about it or the Hep b BD?

The community has high infected persons especially adults. Sharing information is also important. hepatitis B vaccine is also important.

Munzo ne daga AFENET Abuja, Munzune so muke mugani musamman abinda kin fihimta gameda kin fahimta game da wannan ciwo wanda ake ce ciwon hanta wato Hepatitis

Amma kafin mu fara mene sunanki?

[name]

Kamar shekarunki nawa ne yanzu

Thirty-three years.

Kinada Sanaa

Ae inada Sanaa

Kinyi makaranta zuwa wani matsayi

Banyi makaranta ba.

Yaranki nawane?

Yara na bakwai dana cikinnan takwas in Allah ya fitar a raye.

Allah ya rayasu.

Amin

Kina zuwa anti nartal kullum kullum

Ae ina zuwa susai.

Ina son dai ki dan fahimtar dani abinda kin gane kadan game da wannan cewta to.

Menene kin gane game da wannan cewtan?

Hepatitis tana tana sa mutum jiki yana reka mutuwa daga baya sai kaji dok abinda ka taba Kaman abun maiko, daya shafi man geda zaka ji sheki yana kumbure,

zaka ga Kaman an baka gubane kuma ba guba ba, idan ka ci zaki zaiyi maka idan ka ci gyada zaiyemaka, inka sha kunun gyada zakaji cike yan aune, to, dazaran, kana zaune kawai zaka ga jinkinkannan Kaman an bubbugakane, zaka ce murane kuma ba muraba in anbaka shawara kaje asibiti in ka ke, zaka ce kaikam bakada wanan cewta a ciki kuma alhali kana tareda ci a cikinka, idanka je asibiti a je a tararda shi akwai a jikinka kuma, in anbaka duka ka keyaye, in ka keyaye zaka zo kaga gaskiya, abinda da baka ci dinnan to inka ci yana maka coursing wannan damuwa yanzu kam anhanaka, in kabi duka kana son lafiyanka, in baka ci ba zaka ga jikinka ya dawo normal kuma in kana kiwon sa zaka ji babu wani abu ajikinka, kuma kanabin dukan magani, in magani yakare ka kuma wajen likita a je a karamaka magani kana kara samun lafiyanka

Kin san yadda ake kamuwa da wannan ciwo na hepatitis? Ta wani fannine ake daukan cewtan?

To, daga kadan cikin mafi abinda na sani kadan, abubuwan da yake reka cousing wannan abubuwan, Yawan cin man gyada, da abubuwan gyada haka dinnan, baa yawan cin toka, baa yawan cin manja, to, yana iya ya kawu wannan to

Su wayene kuke ganin wanna hepatitis yake kamawa? Sai manyane ko yara ko kuma waye kamar su waye ne suke kamuwa da cewtan?

Mafi yawan mutane da suka balaga, a wurin saduwa, daga gumin mutum, haduwan gumin mace da na meji, idan wannan yana da ci wannan baida ci dole zai iya saduwa da ce ya dauka.

Yara fa?

Ae

Suna samu ne ko ba sa samu kwata kwata?

Yara suna samuwa amma dai mafi yawa a samuwa manya ne.

Ta in yara suke samuwa?

Ae, yara Kaman wand iyayen ci, ko Kaman namiji yana da ce, wannan kuma yaro ne an haife shi, inya dauke I dan gumin shi ya sauko a jikin shi in ya hadu da shi, to zai iya ya sadu da shi ya samu.

Shin kinada masaniya ko akwai hanya da zaa be a tsare yaranmu musamman kar so samu wannan ciwon? Alalmisali ko mama tana dauke da nauyen wannan cewta na Hepatitis B ko kuma ma baba yana da ce. Akwai tsarin da zamuyi mu tabbatar yaranmu kankana jariray da sauransu basu kamu wannan Hepatitis B ba?

Ae. Akwai regakafin ce na musamman wanda likitocin mu suke gayamana akan in gwada mutum bayida ce kuma idan yana da yara a gida yakamata a je ana yimusu rigakafi, da kai mahaifin da bakada ce a je aye regakafi yadda zaa samu a tsareshi.

To yaya keke ji gameda wannan regakafin nan a yara a fahimtanki wanda yana kare yara din?

Ae a gaskiya akwai muhimmanci sosai ayi. Ba matsala in yara sunyi da manya ko wadanda basu da ce, akwai muhimmanci sosai a wurin.

Kina tsammani yana da kariya?

Ae akwai kariya.

A nan wurin anayi ce?

Ae anayi.

Ko matan anguwan ko wadanda suke da yara kana suna zuwa suyi?

Ae suna zuwa

Ko basaso so dauka wannan rigafi na hepatitis B wanda ake bayarwa da zaran an haifi yaro?

Anayi, sonayi, in angayamusu sonayi sosai kuma suna karban shawarwarin likitoci sosai, basu ke.

Ke da kanki ba musamman kin taba nemi wannan maganin hepatitis wato rigakafin hepatitis don a bama yaranki?

Ae

Idan kika haifi yaro yanzu kin sauka lafiya zaki bukaci wannan magani a bawa yarunki?

Ae

Menene keke ganin zai taimaki yaronki don ya kare ce da ce?

Don cewtan nan ku da wanine ya daukeshi, ba wanda ko a cikin gidana ku a jikinaba ko wanine yake taredashi ko mun sadu dace a wani wuri ko a wurin buki ko ka guminshi ya sauka ya shafeshi ba zai kamuba.

Kinada marmari yanzu in kin haifu a bama yaronki wannan magani ko kuma kina dari darin haka watakila saboda wadansu dalilan da bansaniba?

Bawani dari dari, sai muna rukon Allah ya saukeni lafiya kuma yaro yafida da lafiyanci in kawu ci wurin likita yayimice rigakafin wannan.

Kinada tabbacin wannan zai iya tsare yaran kar su samu da wanana ciwon da kin gayamini kina da tabbacin haka

Sosai inada tabbacin haka. Basau dayaba ba sau biyu ba yarana sunfi biyar wanda nayi musu a jarirai.

Kinada sukin samun wannan rigakafi na hepatitis din?

Ae

A ina kike samu?

A cikin wannan asibitin ma akwai

Kuna samu ko da safi ko da yamma ko da wani lokaci?

Da sanyin safiya saboda baayinci da rana.

Koda yaucine kuke samuwa ko litini ko talata, ko lahadi ma in kunzo kuna samuwa? Ko akwai ranakun da in kunzu bakwa samu?

Ranan lahadi mafiyawa maaikatanmu, kamar incharge din mu, in munzu bazamu sametaba tana church, sai mu dakata kawai sai ranan lahadi yawuce.

Kamar lallai zake bukace ce kuma zaki tambaya a baki?

Ae, sosai ba a dari dari wurin bamu

Kina ga ba wata ko damuwa ko wani abu da zai hanaki kawu yaronki ayimice, ba wani matsala da zai hanaki?

Aa ba wani matsala, mijina ma yana bani goyon baya dari bisa dari.

Ko kina tasmani Kenan zan iya cewa wannan allura yana da kew don ki bama yaranki?

Sosai.

Kuma kullum inkin samu dama zaki bama yaranki?

Sosai.

To, matan dasuke tattare dake a anguwanku, Kaman yadda keke ji hakane suma suke ji? Kuma suna fihimta da amencewa cewa wannan hepatitis B din nan, yadda in kin kawu yaranki in anyi musu soma in son kawu yaransu zai kare so, ko kana ga akwai dan tunanin so daban.

Ae, da yake ina karantar dasu, ina gayamusu a fannin addini Allah ma ya yarda mutum ya tsare kance, bare ma bature ya kawa zamani mai sauki, sabuda haka

muyi maza maza abin da Allah yace, mutum ya tasare rance da lafiyanci, likitoci ma sun taimakemu da kawuwa, saboda hake ba kudin machin zamu biyaba ba kudin motaba kuda da kudin mota da mutum yabare cewta ya kwantarda ce a yita sharere, ba gwara yafitu yanada lafiyanci ba, to haka in na sanardasu sai so amasa da wuri, kamar yadda nema nayi haka suma suke yi

Wato kina da kwarin gwoiwa Kenan mata da suke da juna-biyu da wadanda suke da yara kanana wadada suke anguwani dukka za suzo so amchi wannan rigakafi na hepatitis B anan?

Ae sosai

Ko kina da wani abinda zaki gayamana wanda zai iya inganta samun wannan allura Hepatitis B musamman a cikin anguwanku ko ta hanyar fahimtar wa ko hanyar a dan bayani ko kuma wani taimako, akwai abind idan munyi kinaga zai kara ma mata kwarin goiwa sukawu yaransu ayi musu daga can anguwanku?

Ae.

Kamar sume menene zai iya kawo cin gaban samon wannan maganin musamman?

Ae, yanzu haka idan anyi idan Allah yasa na kuma gida da yamma bayan magariba an yi isha in suka zo karatu zan gayamusu anyimana albishir da babban murya an turu baki daga Abuja in kara karantarda ko yauche ko bi shawari na a kullum.

Me keke ga zai karamiki kwarin goiwa ki ce gaba da gayamusu ki fihimtar dasu ko ta hanyar wasu mutane da suke cikin gari ko kuma ta hanyar radio ko fosta?

A kwai dayawa.

Kamar sumenene hanyan da matan da suke zuwa gurin ki zaso ji da wuri?

Yanzu zan je in samu shugaban masallaci, zan gayamice to ladan in an gama sallah, ko maza idan kuka taro a masallaci, wata mace zata ji Magana a bakin na miji, in mijinta yazo da ce zata tabbatar cewa a se malamanmu tagayama a se magananma da gaskene. Don Allah ka tashe da karfin goiwa ka fadakarmin da wandanan a masallaci, kara wata taje asibiti majinta ya tayarmata don taje ba tare da duka ba, neman lafiya kam ba sai an neme duka ba, ku ka dawu baka samu matanka a gidaba, in an she taje asibiti ba wata Magana. Zan gayawa limamai, da fastoci so taimaka da wannan a fadakarawa jamaa ga abunda yake tafiya, a dinga zuwa an karban shawarwarin likitoci da magabata, kara ashe su suka fad basun raayibane,

Wato limaman anguwa zasu taimaka acikin wannan tafiya?

Ae.

Akwai wani abu da kuma zai taimakemu gameda wannan ko awani wurin taruwan jamaa?

Ae ko a gindin biciya ko a wajen dara, ko a wurin mai anguwa daku malamai zasu taimaka wajen isarda wannan sako da maigari da suran fadawansa musamman wajen sanarda mutanen dasu ke zuwa wajen so daga wasu wurare

Nagode sosai.

Nagode sosai malama.

State 1, LGA 2, Facility 5

To

Sannunki da zuwa

Shekarunki nawa?

Talatin

Me keka game da **ciwon** shawara ko ciwon Hanta?

Ciwon shawara idon mutum yana kore, fitsarinsa ma yana kore,

Sheni abinda kika sani akanshi?

Ae shine kawai abinda na sani.

Meye zaki iya cewa a gameda rigakafin wannan **ciwon** da ake baiwa yara dazaran an Haifesu?

Kai wannan kam bansaniba.

Babu wani abu da zaki iya fada gameda wannan rigakafin?

Na yara in an haifesu?

Yana kare musu cewtako ne

Muna maganene aka **ciwon** hanta din shi kansa ragakafinsa mekaka sani akai?

Na yara ko na manya?

Na yara da zaran an haifesu?

Shi kam ban saniba.

Kin taba tambaya a yiwa yaranki wannan rigakafin?

Na gida ko kuwa?

Wanda akeyiwa yara dai da zaran an haifisu. Daga ranan da aka haifesu akwai regakafin da ake musu guda uku akwai na **ciwon** hanta a ciki, kin taba tambaya a yiwa yaranki?

Ae a Sali ni ba nanne garinmu ba rani muka zo, amma anayimusu shan kano ne garenmu, amma ana yimusu

Wannan ciki da keke dashi in kin haifu kina da niyan a yima yaronki wannan allura?

I dan na hafeshi ko kuwa?

Ae,

To ba sai na haifeshi zaa yi marba?

Ae, ai tambayace in an haifeshi zake iya bare ayemasa.

Aaa zan barimana tunda na rabu dace lafiya sai na hana ayimas allura?

Me yasa zaki bari ayimasa?

Sabeda cewtakon mana

Kawai ba wani dalili?

Hmmmmmmm

Kinada wani tunani ko tsoro akan wannan allura?

Naji tsoro kar ayimas ne ko kuwa?

Ae, ko kuma wani abu zai iya samunsa in anyimasa?

Aa aa, banida wani tunani.

Meyasa kika fadi haka?

To baanayibane ba ba abinda yake samun yara, nikuma sai ince kar ayimasa?

Kina gain cewa shi wannan allura da akewa yara yana kare su da shi **ciwon** hanta?

Ae yana karewa kam.

Meye dalilinki na fadin haka

Sabida naga anayima yara

Sabuda anyimusu shi yasa keke ganin yana karewa?

Aeee

Kina ganin zaki iya samun wannan allura ci vaccine din idan kin haihu idan kin tamabaya?

Idan na tambaya anan asibitin?

Aee. In kin nema zaki samu?

Wanne Kenan?

Shi alluran rigakafin?

In dama anayi anan?

Ae anayi.

Idan kin nema anan akwai abinda zai hanaki samuwa?

Babu abinda zai hana samu, in dai anayi.

Zaki tambaya ayimiki? A yima yaron?

Idan na haifu?

Eee ina ana yi ma yaran

Aee

To zaayi masa,

A kawi wani matsala dazai hanaki samun wannan rigakafin a ganinki?

Ba matsalan dazata hanani

Kuma kina ganin wannan allurar rigakafin tanada amfani ga yara?

Tanada amfani sosai ma kuwa.

A jumlace kina ganin mata masu cike a nan karkarar suna samun wannan allurar rigakafi?

Ina ganin suna samu.

Meye yabaki wannan tabbaci?

Ina ganin anayima yara rigakafi anan garin

Shene tabbacinki?

Shine tabbacina naga rigakafin yana da amfani a wajejen yara.

Kinada wata shawara tayadda zaa kara ilmantar da mutane a wannan yankin a kan wannan alluran rigakafi? A kwai wata shawara dazaki bayar?

Shawari wace eri?

Tayadda zaa bunkasa yima mutane wannan alluran rigakafi?

Gaskiya bani da ita.

Kinada bukatar kisan menene hepatitis din? Meye wannan ciwon? Tawace hanya kekeso kesan ko a ilmantarda ke akan wannan **ciwon**?

Ciwon hantan ne?

Aee.

To a ilmantar dani

Ta wace hanya? Ta hanyan labaria ko gidajen redio, ko fosta, ko aa aa ta wace hanya keke gani?

Hanya mafi sauki dake jin abubawan dasuke faruwa?

Ta waya da akeji ko ta redio

Kina ganin haka shi yafi? Tayaya keke so, idan misali akazo zaa koyarda ke akan abin daya shafi wannan, ta wace hanya keke gainin yafi sauki zaki iya ganewa da wuri?

Nikam tunda waya tafadi ban sanja wata ba.

To tahanyan labaru fa ?un

Batun mekenan?

Ta hanyar dazaa sanardake abubuwan dake tafiya akan wannan **ciwon**?

A kwai wani abu da zake iya gayamana gameda **ciwon** shawara ko alluran rigakafinsa

Aaa aaa,

Babu?

Babu.

To mungode.

State 1, LGA 2, Facility 5

To

Ashirin da biyu

Me kika sani game da Ciwon Hanta/ shawara? Kin taba ganin wanda ya kamu da wannan cewon, ko hanyoyin da ake kamuwa da ce.

Ban taba ganin wanda yakamu da shi ba

Amma kina gani cewon in mutum ya kamu da shi?

Yanas kasala,

Iya abinda kika sani Kenan a kan ce?

Aee. Wani lokaci kuma zaka ga yanayin mutum yana chazawa, zaka ga idon ce yana canzawa, akwai yayana kam ma idonsa yana chanzawa yazama yellow

Me kika sani game da allurar Rigakafin ciwon Hanta/ shawara da ake yiwa yara dazaran an haifesu?

Abin da nasanikam dai ance yana kare yarane

Shine iya abinda kekasani ko akwai wani abu?

Aee yana karawa yaro lafiya yin rigakafin. Kuma yana kare yare daga cewtottokan dazai biyo baya.

Meye ra'ayinki akan ita wannan allular rigakafin ciwon Hanta/ shawara da ake yiwa yara da zarar an haifesu?

Ni kam a rayinakam yana da kew ba matsala tunda zai kare lafiyane.

Shin a baya kin taba neman a yi ma yaro/ yarinyarki alular rigakafin ciwon Hanta/ shawara da akeyi da zaran haihuwa?

Aee, dakainama nakaishi

Shin Kina da shirin bada yaro/yarinyar ki ayi masa/mata allular rigafin ciwon Hanta/ shawara da zaran kin haihu?

Aee sosai ma

Meyasa zaki amince?

Saboda lafiyan yaron.

Kwai ko akwai wani abu?

Aee saboda lafiyan ce gudun samun matsala nan gaba.

Shin Kina da wani ra'ayi, ko damuwa, ko d'oki, akan ita wanan allular?

Ba wani raayi sai dai kawai in ga nayi ne kawai

Shin Kina ganin wannan allular regafin ciwon Hanta/ shawara da zaran an hahihu na iya kare yaro/yarinyar ki daga kamuwa da ciwon Hanta/ shawara?

Aee.

Shin kina ganin zaki samu ayiwa yaro/yarinyar ki allurar rigakafin ciwon Hanta/shawara Idan kika nema da zarar kin haifu?

Aee in so

Zaki nemi a yi ma yaro/yarinyar ki?

Aee

Me ya sa kika fadi haka?

Saboda kare lafiyan ce.

Cekenan?

Aee.

shin kina da wani tunani ko matsala da Kike ganin zai iya hana ki samun wannan allurar ta ciwon Hanta/Shawara da ake badawa da zarar haihuwa?

Aaa aaa

Ba wata matsala dakeke ganin zata hanaki samu?

Babu.

Shin Kina gananin wannan allular rigakafin da ake yi da zarar haihuwa ta ciwon shawara/Hanta tana da amfani ga yaro/yarinyarki?

Yanada amfani kam.

Meye dalilinki na fadin haka?

Sobada zaka yawanci yara na cikin gari dana kawye da banbanci, ko daga yanayin lafiyansu da kuzarinsu, zakaga akwai banbanci, saboda yaran da suke kawye basu damu dayin rigakafi ba.

A jumlace shin Kina ganin cewa mata masu juna biyu a nan yankin na samun ita wannan allurar rigakafin ciwon shawara/Hanta da zaran sun haihu?

Aee indai sun ziyarci asibiti suna samu

Me yabaki wannan tabbaci?

Saboda ni dana haifu nazu kuma nasamu anyimini.

Shin kina da wata shawara da za ki iya bamu ta yadda zamu kara wayar ma alumma da sauran jama’ar wannan yanki game da muhimmancin allular rigakafin cutar shawara/Hanta da ake badawa da zarar haihuwa?

Sobada dayawa mutane suna ganin regakafin nan Kaman baida wani amfani hakannan, wata tana ganinma tazu rigakafima bata lokacine gara tayi aikin gidanta kawai.

Meye shawarar dazake bamu? Ta yarda zaa wayar da mutani kai.

A dinga nunamusu amfanin yin alluran rigafin din, ana nunamus amfanishi da muhimmancin ce ga lafiyan yaransu, ko kuma su dakansu.

shin zaki so k’arin sani game da ciwon Hanta/Shawara?

Aee

Ta wace hanya? Labarai vedio ko majigi ko fosta da sauransu?

Ko ta labara, saboda yanzu anfi maida hankali akan kallon labarai

Ke tawace hanya kekafi so

Aee ne nafi so a ta labarai hakannan.

Ta redio ko newspaper?

Ko ta gidan TV ko labaru hakannan sabod ba kuwane yake iya karant abu.

Shin akwai wani abu da kike tunanin ya kamata mu sani game da wanan ciwon, ko hanyar yada bayanai akanta ko gameda allurar rigakafinta?

Ni dai kam a sonakam musan ya yanayin ciwon yake kuma yaya alamunci yake kuma yaya zaayi ka kare kanka daga abin da zai kaika ga irin wannan matsalan din.

Cikenan iya abinda zake fada?

Aee

To mungode da lokacinki.

Nima nagode.

State 1, LGA 2, Facility 6

Introduction

I am [name] and I am With [name] from CDC AFENET for Hepatitis Baseline survey.

What is your name Is [name], I am a Nurse midwife?

what are the biggest challenges with people living with Hep B in this community?

One is ignorance, second people can't afford money for the test and also money for the drugs. Some will have chronic liver disease with ascites

Ok what are the biggest needs to address hepatitis B in your community? To create awareness, and there is vaccine for its people should go for vaccination. People can also get it from sweats from a patient like in a vehicle.

Is there a strong need for hepatitis B birth dose?

There is a great need to prevent the children at birth

Why did you think that?

Beside the health care workers, do others see the need for Hep B birth dose?

Ther is a great need because it helps and it prevents one from contacting the disease once you have it at birth. And if there is awareness among people they will come and take the vaccination.

Can you tell me about the current programmes and practices for hepatitis B birth dose in your clinic?

Women are coming for the vaccine though most come late at 2 weeks but we emphasize for women to have it at the clinic after birth

Is Hep B dose easy to get within 24hours of birth?

Yes because when women give birth they stay in the clinic to get the vaccine tomorrow before going home. Once we request from LGA, we get it unless it is out of stock

I

Who can administer the hepatitis B dose?

The technical staff, nurses and CHEWS

Do you have any challenges/barriers of getting the Hep B birth dose? Yesm, when it is out of stock

Have you gotten any information from participants regarding their experiences regarding hepatitis B birth dose? For those have gotten immunization, some say it is effective because they don't contact the disease even with people having the disease

what is the community understanding of the Hep B birth dose?

Most ANC women are given awareness about Hepatitisi B BD to be taken at Birth

Can you say the people are interested in getting the Hep B BD? Yes, women are coming out from it, those that miss it also accepts that it is their fault

Is there is no any information or message from the community about Hep B BD that you heard?

They encourage their neighbours and friends to come for the zero dose.

Do you have any ideas or suggestions for improving Hep B BD vaccination rates?

To have a massive campaign on it. Not only cjhilderen bur asdultres dsholud also get the vaccine

Community level

At health care level

Health workers should take precautions

For the providers?

Vaccine should be available at all times

Are there any specific strategies for education or awareness that might be effective?

Yes, the should create awareness at the markets, mosques and churches

What's your experience with maternal tetanus vaccination? Vaccine ine is effective, once you have it at the right time and right dose. It is very effective for all women of child bearing age.

What are barriers or challenges you experience related to maternal tetanus vaccination?some women out of ignorance don't shoew up toimely for second d dose at 4 weeks

What successes have you experienced and what is working?

For those that have complete dose, say the vaccine is reffective

What type of health interventions have been most successful in your community, and why have they been successful?

Some of the services are, use of MAMA kids, for ANC women. Women accepted it and it was successful

Why have they been successful?

It was successful because women come to clinic for delivery

Is there anything else you think we should know about Hep B, sharing information about it or the Hep b BD?

If Government will provide free test kits, it will encourage people to take the test. Information could be shared best through town criers in this community

State 1, LGA 2, Facility 8

Introduction

Good morning, Ma, I will start by asking you your name.

my name is [name],

as I said I am [name], and with me is [name]. We are from CDC-AFENET for a baseline assessment of Hepatitis B Birth Dose. As part of our assessment to conduct interview with a health care worker.

How old are you Ma?

I am 51years old.

What is your designation?

I am the health facility manager.

what are the biggest challenges with people living with Hep B in this community?

The challenges are in terms of treatment and then feeding, and economic problem and how to take care of their selves.

Ok what are the biggest needs to address hepatitis B in your community?

First, immunization, then health talk and educate the community about how to contact hepatitis b and ways or measures from avoid getting hepatitis B.

Is there a strong need for hepatitis B birth dose?

Yes, there is a strong need for Hep B birth dose. Why did you think that?

That will help in preventing hepatitis b within the family before the child will be exposed, the baby is already immunized.

Beside the health care workers, do others see the need for Hep B birth dose?

Yes, the community see one who is affected and is down with it, when they see the situation, the person is faced with it, every one of them will desire to have his or her child immunized with hepatitis b vaccine to avoid any problem.

Can you tell me about the current programmes and practices for hepatitis B birth dose in your clinic?

What we have is the routine immunization. As hepatitis B part we have started implementing it and we will continue to implement it. It is given during sessions and outreaches just like today we have a session. When a woman gives birth in the facility, we give within 24hours, but where she gives birth in community outside the health facility, we will get them vaccinated during outreaches within two weeks. We also collaborate with our community leaders., village heads, religious leaders to mobilize women who deliver in the community to come to the facility during the naming ceremony.

Ok is Hep B dose easy to get within 24hours of birth?

It is easy to get because we have solar facility here.

Who can administer the hepatitis B dose?

The trained staff and any one that undergo the training.

Do you have any challenges/barriers of getting the Hep B birth dose? No

Have you gotten any information from participants regarding their experiences regarding hepatitis B birth dose? No

what is the community understanding of the Hep B birth dose?

They accepted it as a prevention that help to protect their children against hepatitis B, and as good advancement

Can you say the people are interested in getting the Hep B BD? Yes sir

Is there is no any information or message from the community about Hep B BD that you heard?

Yes, the information I heard is impressive, people like it and people accepted it and are willing to give their children for vaccination.

Do you have any ideas or suggestions for improving Hep B BD vaccination rates?

Just I have said earlier, by village head and religious leaders, and community leaders to pass information through them so that people will be knowledgeable about hepatitis

Community level

What of at Health care system level? Educate mothers during ANC and delivery about the vaccine for their children

For the providers?

They should inform us earlier if vaccine will be available

Are there any specific strategies for education or awareness that might be effective? Yes, town hall meeting and community influential people. The meeting with community leaders and stake holders

What's your experience with maternal tetanus vaccination? Mothers have been taking it especially during ANC from first visit and second visits etc they like talking it

What are barriers or challenges you experience related to maternal tetanus vaccination?

No barriers

What successes have you experienced and what is working?

They accept the vaccine and come for subsequent doses.

What type of health interventions have been most successful in your community, and why have they been successful?

ANC, labour and delivery and immunizations

Why have they been successful?

Is there anything else you think we should know about Hep B, sharing information about it or the Hep b BD?

You will need to update the knowledge of health workers about hepatitis B vaccination. Few women rejects vaccine for AEFI like kurga or garsa. This could happen within two weeks of birth

<u>State 1, LGA 2, Facility 8</u>

To hajiya hauwa

[name] munzo daga Abuja akwai wasu yan tambayoyi da zamu yimiki akan alluran rigakafin da ake bawa yara dazaran an haifesu.

Kinsan ciwon hanta ko shawara?

Ae,

Mekeka sani gameda ciwon? Ko ba abinda keka sani?

Aeee.

I dan kin haifu anaba yaronki alluran rigakafi?

Aee,

Daga an haifeshe kafin awa ashirin da hudu ana bashi alluran rigakafi?

Aee

Rigakafin me ake bashi?

Na hanta.

Meye raayinki gameda shi wannan alluran rigakafi?

Ana cewa yanada amfani.

Kin yerda da hakan?

Aee.

Kin taba tambaya ayiwa yaronki wanan allura, da zaran kin haifu?

Aa aa,

Kintaba tambaya?

Aaa aaa, bantaba tambayaba.

Amma da zaran kin haifu anayima yaronki?

Aee.

Cikin dakeke dashi yanzu in kin haifu zaki bari ayimas wannan alluran rigakafin ciwon hanta ko shawara?

Aee,

Meye dalilinki?

Don yasamu kariya

Shin akwai wani damuwa dakeke dashi game da shi wannan alluran rigakafin ciwon hanta ko shawara?

Aaa aaa, Babu.

Shin kina ganin wannan alluran rigakafi yana kare yara dage ciwon hanta ko shawara?

Aeee

Meyasa keka fadi haka

Yanzu haka duniya ta kuma in babu magani abubbuwa bazasu daidaituba.

Shin in kin haifu zake samu ayima yaronki wannan alluran rigakafi?

Aee in Allah ya yerda.

Shin zake tambay a yimasa?

Aee zan tambaya.

Shin akwai wani abu dazaisa a kasa yiwa yaronki wannan alluran rigakafi?

Ina ganin kamar babu. Sai dai in ban kawociba.

Shin wanan alluran rigakafi yanada amafani ga yaronki?

Yana amfani sosai.

Meyasa keka fadi haka?

Kamar ciwon daake kira meshi meshi, ko tarin puka, duk yana karewa daga kamuwa da wadannan.

Shin awanan yanki naku duk mata masu juna-buyu in sun haifu suna samun wannan allura na rigakafin ciwon hanta ko shawara?

Ae suna samu.

Meye dalilin dayasa keka fadi haka?

Muna tare dasu ina gani suna kawo yaransu, wani lokaci muna zuwa tare.

Shin kinaso akaramiki illimi akan shi wannan ciwon hanta ko shawara?

Aee ina so.

Ta wace hanya keka fi so a garamiki ilimi akan ce wannan ciwon? Ta hanyan makaranta ko vedio ko fosta?

Kota wace hanya dai, yadda zan fihimci abind ake fada.

Gameda sauran harkan kiwon lafiya kuma, ta wace hanya keke so akaramake illimi akai?

Inso inji,

Kamar ta wace hanya?

Kota wace irin hanya, yadda dai zan samu sakon kuma in fahimta.

Akwai wani abu da keke so musani gameda shi wannan ciwon shawara?

Aea

Kamar me? Wani abu da zake gayamana wanda bamu saniba?

Aaa aaa, ni banda wani abu da nasane.

Babu wani abu dazaki kara mana bayani akanasa?

Babu,

To

Mongode.

Transcription for State 1, LGA 2, Facility 10

Sannun ki da zuwa ko.

[name] daga Abuja don mu tambayeko akan alluran da akeyiwa yaranku da zaran an haifesu wannan kuma sunansa [name].

Akoi wane ciwo da yake kama hanta an shemasa ciwo shawara, anayiwa yara alluran rigakafin sa aranan da aka haifesu kinsanshi?

Ae

Mekeka sani a gameda shi? Menene masaniyanki a gameda shi, ko yadda yake kama mutane, ko yadda ake kamuwa dashi ko yadda yake wahalarda mutane da sawransu

Amsa:

Ina jin labarin amma bantaba ganiba.

A koi alluran rigakafin da akeyiwa yara na wannan ciwo a ranan da aka haifesu. Mekekasani akan wannan alluran?

Duka ban sani ba.

Meye raayenki a game da wananan alluran rigakafi?

Raayi kaman? Zaki yadda ayiwa yarunki ko ba zaki yedda ba? Sannan meye dalilinki?

Zan yadda ayiwa yaruna.

Meye dalilin ki na cewa zake yedda a yiwa yaronki?

Don ya samu lafiya.

Shene kawai dalilinki?

Ae.

Shin a baya kin taba neman ayiwa yaronki wannan allura?

Ae na taba.

Yaushe?

A shekaran daya wuce.

Yazu kum kina da sherin an kika haihu ayiwa yaronki wanan allura?

Ae.

Meyasa zaki amenche ayimasa?

Don cewtanda yake a jikinsa ya fita.

Kina da wani rayi ko damuwa akan wannan allura?

Babu.

Bawani damuwa dakeke dashi?

Ae.

To kina ganin wannan allura yana kare yara daga kamuwa da ciwon hanta in an yimusu

Ae.

Kina da wannan raayi cewa in anyimusu wannan allura bazasu kamu da ciwon hantaba? Kefada inji mana da bakinki.

Ae, ina da raayi

Shin kina gani zake samu ayiwa yaronki wannan allura idan kin haifu?

Ae, in gainin haka

Meyasa kikeda wannan raayi?

Don kada insha wahalan jinya.

Zake nema ayiwa yaronki in kin haifu?

Ae

Meyasa kika fadi haka?

Don kar insha wahala ne.

Shan wahala na me?

Don irin kananan damuwa a jikin yaro

Shin kinada wani tunani ko matsala da keke ganin zai iya hanaki samun wannan allura in kin haifu.

Babu kam.

Kin tabbata?

Ae kama.

Shin kina ganin wannan allura da akeyiwa yara idan an haifisu yanada amfani?

Ae, yanada amfani.

Meyasa kika ce yanada amfani?

Hmmm, don ko yaro yana da damuwa inkazu in kayimas ko akwai hanyan kudin dazaka iya kashwa, idan kayimas alluran nan zai taimaka maka

A jumlace shin kina gainin cewa mata masu juna-biyu a yankinkun nan suna samun ita wannan allurar rigakafi?

Babu.

Basa samu?

Ae

Meyasa kika fadi haka?

Don basu kawu yara asibiti.

Basu kawu yara asibiti?

Ae. Wadanda suka kawu yara asibiti suni kawai suke samu? Ae, wadanda basu kawu yara asibiti ba ba’a binsu a wani wuri ayimusu. Ana binsu wasu ana samun so wasu baa samunsu

Shin kina da wata shawara da zaki iya bamu ta hanyan dazamu wayar da kan allumman da sauran jamaam wannan yanki game da muhimmanchin wannan allura

Banda wani shawara?

Bawani hanyan da zaabi ace in an zuda wani abu na asibiti ko na kiwon lafiya zaa iya fitarada sakon ga matan da suke a anguwanko? Babu wata hanya dazaa bi a samu haka?

Idan sun ji labari zasozo, zaa iya tarasu ace to gashe ga abinda ana so ayi yau sodaka soyi abun tukum

Ta wace hanya raa samu soji labari musamman a saninki?

Zaa iya gayamusu

Shin zaki so ganrin sani game da ciwon Hanta, zaki so a garamiki sani gameda ce?

Ae.

Ta wace hanya kikeso a garamiki sani? A garamiki sani ko hanyan labarai, video ko posta da sauranso?

A labarai dai.

Kinfi so a yimiki ta hanyan Labarai

Ae

Bawata hanya kuma dakeki tunani?

Babu

Shin a kwai wani abu dakeke so musani gameda wannan ciwo?

Ae

Kaman Me? Wanda keke gani bamu saniba kinaso ki karamana bayani?

Ni ban san wani abu gameda hakan ba.

Gameda alluran rigakafin fa, babu wani garin bayani da zakiyi mana?

Babu.

Gameda hanyan yada labaran ciwon fa, a kwai wani garin bayani da zakiyimana?

Kai Babu.

Mongode sannunki.

So

Sannunki da zuwa

[name] tareda ne akwai [name]. Munzone daga Abuja. Zamuyi meki tambayoyi a kan alluran rigakafin daakeyiwa yara daga ranan daka aka haifesu. Kin fahimceni?

Aee

A yankin kunnan menene matsala babba da mutane suke fuskanta kan cewon shawar ko ciwon Hanta?

A nan kam da ne ake samun cewon chanye hannunan, daga lokacin da anka fara rigakafi, yanzu kam bamu samun cewon din, sai wadanda suka girma dace, yanzu kam ba cewon din.

Gameda cewon hanta fa ko shawara?

Gameda cewon hanta, akwai ciwon hanta a wurin manya suna samun ciwon hanta, wadansuma har ya kaisu ga mutuwa domin mutaninmu sonada rashin fahimta kadan, idan angayamusu wannan ciwon hantane sai su shega suna shan gargajiya, sai ciki ya kumbura, wadansu sai su mutu, amma acikin yara da ake masu rigakafi kam kai bamusamun wannan damuwa.

Menene abinda mutunenku suke da bukata sosai akan abinda ya shafi ciwon hantan nan?

Mutanenmu suna bukatan ataimakesu da magani na manya, don in anje rigakafin yara anshe ana yima yara rigakafin ciwon hanta sai suyi ta tambay suna cewa su manya kam ya baa yimusu ai suma suna kamuwa da ciwon, suma a kawomusu magani na manya gameda ciwon hanta nan a dan taimakeso, suma suna bukatan magani.

Kina gani mutane suna bukata ayima yaranson wannan alluran rigakafi in an haifesu?

So sai.

Meyasa kika fadi haka?

Dumin da ance regakafi ba a fitowa, ko arin na gida gidan nan ba duka zasu kasance a gida ba, amma yanzukam, jiya acikin ruwa ana ta fitowa ana zuwa rigakafi anan. Domin son san amfanin rigakafi yanzu.

Wadansu mutane da kuke taredasu kina ganin suna bukatan ayima yaransu wannan allura?

Suna bukata, kewaye damu kam ba wadanda basa zuwa, ko bururin daji ma suna zuwa su tambaya a kai musu rigakafi

Zaki iya gayamine tasre tsare da ake dashi awan asibiti, ta yadda akeyima yara wannan alluran rigakafi?

Kamar?

Aee Kamar yadda akeyi ayimusu wannan allura?

Ee to, irin in an haifi yaro ne?

Aee,

In an haifi yaro, daga an haifi yaro anayimas allura, ana gayamusu ko a gidane in kuskure ya kama baa haifi yaro a asibitiba, daga an haifishi a daukushi a kawuci absibiti don ayimasa wannan alluran ciwon hanta,

Ci wannan alluran rigakafin ana samunsa da sauki anan?

Aee ana samu, aje Local government a dauko a zo ayimana rigakafi.

Meyasa kika fadi haka?

Ina gani a duk rana dazaayi rigakafi wani yanazuwa ya dauko magani da vaccine carier kafin a zu ayi rigakafi anan, ba wai ana ajiye ce anan ba. Kuma ana samu ba randa zaa je ba a samuba.

Da waye da waye kika sani yake bada alluran rigakafin din?

A nan yanzu hope ne takeyi in batanan manager tanayi

Sune kadai kika sani suke bayarwa?

Aee, a yanzu.

Shin akwai wadansu matsaloli dakike ganin zasu hana samun wannan alluran rigakafi?

Kai bansaniba dumin ban taba iske wani matsalaba.

Daga mutanen da suke karban wannan allura kin taba jin abinda suke fada na amfani ko rashin amfanin wannan allura?

Sai dai abinda ya amfanesu, don har bororin daji ma son zo anan sunce da suna gefin Lau yaransu suna ta mutuwa, domin sunki rigakafi achan amma da sun zo nan anabinsu a hankali a hankali har sunzo sun karba yanzo ko yaronsu baya mutuwa, sonata zuwa suna godiya ma.

Mutanen yankin ko anan meye tunanin su akan wannan allura? Ku kuma meye suka fahimta akan wannan allura?

Mutanenmu sun fahimci abubuwa daban daban, domin da kamar a watan bakwai zaka ga yara suna ta mutuwa, a ce an takasu abin gargajiya meye meye, da aka fahimci kan rigakafi yanzu, kai ya dauki shekaru ban ji yaro ya mutu acikin bincolan nan ba,

Mutane sona da marmarin a basu wannan allura?

Aee, akwia lokacin ma da aka daina kawu rigagin har an zuwa ana tambaya. Suna son rigakafi sosai.

Wasu irn labaru ku maganganu kike ji mutane suna fada akan wannan allura.

Mutane suna fada, ko a church, sana fad cewa rigakafi ya taimakesu, kuma sun cewa wanda ya kawu rigakafi a duniyan nan an godemasa sosai domin baza ga tsananin ciwo sosai a jikin yaro ba. Irin su bakon dawron nan in ya shego gari zai wuce kawai ba zai yi wani abu ba.

Kina da wata shawara da zaki bayar a gameda mutanenku ta yadda zaa kara yawan yin wannan allura?

Aee

Meye shawaranki akai?

Na daya ina zuwa in gayamusu wannan rigakafi taimakonmu akeyi ba kudi zamu biya ba gara mu hadakai mu dinga fita muna karban rigakafi dumin lafiyan tya tyanmu, do min inganta rayuwanmu, na sha fadin wannan kam ko a church, ko a anguwan Fulani ina fadamusu, ko a masallaci, in sun aminci suna zuwa rigakafi.

Wace shawara zake bayar ta hanyan da zaa bunkasa wannan aiki na alluran rigakafin yara na ciwon shawara dazaran an haifesu?

Ko baki da wata shawara?

To ina so inbako, a dan taimakemu dai a karamana magani, har manya ma suna sun rigakafin din ya zaayi, manya ma suna so ba ranan da bazasu tambayaba.

A nan asibitin tayaya kike ganin yakamat a bunkasa yin wannan alluran rigakafi don kuwa da kuwa ya samu?

Yadda?

Baki fahimciniba?

Aee

Wace shawara zaki bayar dun a bunkasa yin wannan alluran rigakafin a wannan asibitin don kuwa da kuwa ya samu?

Yanzu kina illimin yadda ake allruan ai

Aee

Tayaya za bukasa yin wanana allura?

To, ni dai ina munada bukatan a karawa asibitin mu girma kuwa ya zo ya ga wurin da dan girma.

Su kuma masu kawo alluran, wani shawara zaki bayar ta yadda zasu bunkasa kawo alluran?

To ina musu godiya su riga tunawa damu, kada amanta da mu dumin mutanenmu suna son alluran. A rika kawumana dumin muna karbansa hannu bibbiyu, mongode musu kuma.

A gainink akwai wasu hanyuyi da kike ganin su suka fi inganci wujen yada labara ko yada ilimi akan wannan ciwon da kuma alluran rigakafin?

Hanyuyin Ilimi wanda zaa fahimtara da mutane ne?

Ae,

Dumin anan muna da mobalizers, wadanda suke ciga kawyuka, kawyuka, suna gayama mutane, ana wayar da kansu gameda alluran rigakafi.

Kina ganin wannan hanya ita tafi, ko kina ganin akwai wata hanya da ta fi?

To Wannan shene wanda mukeyi, muna wayar ma mutane kai batun rigakafi, sai dai ban sani ba in akwai wata hanya.

Ki kike wannan community ai

Ae,

To wace hany kike gani yakamata a bi wajen wayar da kan mutane ko sanardasu gameda o ciwon?

A je anya wayara da kan mutane ko a church ko a masallaci yana taimako sosai.

Zake iya gayama experience dinky akan alluran da akeyi na tetanus.

Ae, tetanus tana da amfani sosai. Ko masu ciki in abu ya yankesu, in an yimusu titanus yana da taimako, ko wani mutum wanda kusa ko wani abu ya wanda zai kawu mishe damuwa a jiki in an yimas alluran tetanus yana da taimako.

A kwai wasu matsaloli dasuka shafi wannan allura a wannan yankin?

Matsaloli?

Aee,

Alluran tetanus din?

Rashin yinshi?

Aee

Aee to akwai, wadada basaso soyi in abu yatabasu kadan, sai kafa yayi ciwo

Meye nasarurin daaka samu na wannan alluran rigakafin.

Waii, dayawa kawai.

Ki kirga manasu mana.

A nan kam alluran tetanus, akwai wanda fartanya ya sare shi ya wuce ya tafi gida, daya ji gida kafnshi ya kumbura, aka kawuce anan asibiti, alluran tetanus nan ne kawai akayimishi, ya warke, harya fada a church cewa alluran tetanus da suke ganinnan yanda amfani sosai kar ayi wasa da shi. Wai a se abinda ake renawan nan shene zai nakasar dasu. Wai jiya jiya akayimasa alluran tetanus wai gashinan

kafanci ya warke, akwai wand ma kwanu ya yanke shi anyimas allura tetanus ya warke, kai akwai dayawa kawai abind alluran tetanus yayi anan kam.

Daga cikin savices na kiwon lafiya da ake kawuwa anan, wannene kekaga yafi amfani ma mutane?

Duka abubuwan da ake zuwa anyi, ba sai wannan allura ba me keke gainin ya fi ammfani sosai?

Kai rigakafi yanada amfani sosai. Dumin abinda zai kawu lafiya tya tyanmu, da gidan sauro yana taimako anan sosai.

Wadanana biyu su suka fi amfani?

Duka saunada amfani. Ga mama kid da a ka kawunnan mutane suna rushin batun mama kid dinnan sosai.

A kwai wani abu dakeke su ma gara sani akan wannan ciwo a tunaninki? Yanzu kam an bude miki kofa. Akwai wani abu da keke so ke gara gayamana akai? Gamed shi ciwon hanta?

Abinda zan kara fadi?

Aee

To saidai shawara dumin wanan ciwon hanta bamusan ci yaya zaa rabudashiba, ta wace hanya ko wani irin abincine in mun ci zamu kamu da shi. Ko kuma, kai dai wane irin yanayi ne zamu shiga cike da zamu kamu da wannan ciwon hanta. Ni kuma in je in wayar ma mutane kai.

Mongode da hera damukayi dake.

Mongode.

State 1, LGA 3, Facility 1

Moderator: Assalamu alaikum! Sunana [name] ina aiki tare da hukumar NCDC Affenet. Muna wani bincike ta wani rigakafi a wannan jahar ta Adamawa. Bayan jahar ta amince da wannan bincike da muke, Muna godiya da bamu lokaci da kayi na wannan tambayayo da zamuyi ma Maka. Wannan tambayayo da zamuyi maka ba dole ba ne ka ansa ta Kuma zaka iya tsaya a kowani lokaci ka daina cigaba da amsa mana tambayo yin har zuwa karshen tambaya da zamuyi ma. Babu amsa da yake dade ko ba dade ba kawai muna so mu gane mene ne tunanin ka da kuma raayin ka akan wannan bincike da zamuyi. Wannan hira da mukeyi muna recording nashi sabida mu tabbatar da inganchin abunda zamu rubuta Akai ba tare da mun rasa wani abubuwa da zaka fada ba sabida muhimmancin sa, duk abunda ka fada ba zaa gane waye ya fada ba bare a nemo ka. Wannan hira da zamuyi muna da tsahon minti 30, kana da wani tambaya kafin mu fara?

Respondent: Akwai tambaya, Inace kace batun akan rigakafin yara na hanta. Lalle primary health care na gari anayi, gaskia ana kokari.

Moderator: Yanzu ka amince ayi ma tambayo yin?

Respondent: Eh na amince.

Moderator: Munaso musan shekaran ka yanzu da farko.

Respondent: Shekaruna inada hamsin da biyu (52).

Moderator: Menene matsayin ka a wannan garin?

Respondent: Matsayina dai na mai anguwa a wannan yankin da asibiti take.

Moderator: So wani matsaloli ne mafi girma da kake gani wanda mutane masu dauke da ciwon hanta suke fuskanta.

Respondent: Ehto, Gaskia maganan ciwon hanta a asibiti nan bamu da irin wannan case din. Baa taba kawo mana case irin na ciwon hanta ba Kuma Na yara da kake magana ana kawo su sabida rigakafin ana kawo su suna samu.

Moderator: Yanzu kaman manyan mutane da suke dauke da wannan ciwon wani matsaloli suke fuskanta acikin al'umma?

Respondent: Irin haka idan har ya faru, anzo anyi gwaji anga kana dauke da wannan ciwon toh atake ana cewa akai mutum asibiti babba cen suje ayi masa kome da kome don ciwon yafi karfin wannan karamar asibiti.

Moderator: Ya mutanen gari suke kallon wanda yake dauke da wannan ciwon din?

Respondent: Toh! Mu muna gani kaman ita ciwon hanta, bata yadu ba kaman su AIDS or wasu ciwon ba bamu san menene matsalan ta ba sai dai muce ciwon hanta ya kama wane bamu san menene matsalan ta ba bare har aguji mutum.

Moderator: Toh wasu manyan bukata kuke dashi na magance wannan matsala awannan gari na cigari?

Respondent: Tunda Allah ya kawo Ku, Kuma in akwai dai hanyar da zaabi a taimaka mana idan akwai magani da zaa bayar kaman na rigakafi da akace na yara muma manya ya kamata ayi damu.

Moderator: So kana gani akwai bukata sosai na ganin cewa anyi ma yara da zarran an haife su wannan rigakafin ciwon hanta?

Respondent: Sosai akwai bukata.

Moderator: Mesa kace haka?

Respondent: Duk abunda ka gani ance ayi ma yaro toh lalle yana da amfani inda ace baa son tayi amfani ba zaa kawo abun ba. Anga amfanin Shi sosai shiyasa akace azo ayi ma yara.

Moderator: A ganin ka sauran mutane suna ganin muhimmancin kaman yanda kake gani?

Respondent: Eh suna gani sosai Kuma duk raayin yayi daya sabida wannan I charge namu yana kokari Yana kiran manyan Yan anguwanni yace ga abunda aka kawo kuma Yana roko dan Allah ayi sabida muhimmancin sa, Dan Allah afadawa talakawa su kawo yara dan Allah ayi musu kaza kaza Kuma haka muma Muna kokari ana kawo yaran sabida samun sassaucin cutan.

Moderator: A ganin ka akwai wani tsari da asibitin nan take dashi wajen da zai taimaka yara su samu rigakafin ciwon hanta da zarran an haife su?

Respondent: Yanda dai na gaya maka dai I tsarin nan shi mutumin asibitin nan da na gaya ma yana kokari. Yana da basira tunda dai har yana iya ya neme mu, ya neme mutanen da suke kasan mu, watau karkashin mu azo mu zauna a kawo mana yara ayi abun nan kaga anyi basira anan.

Moderator: Kana ganin shi rigakafin ana samun shi cikin sauqi idan mace ta haihu ana mata da wuri?

Respondent: Sosai.

Moderator: Mesa kace haka?

Respondent: Wani lokacin ma idan mace ta haihu anan shi ba zai yarda ba sai anyi allurar kafin ta bar asibitin nan in kace ma sai gobe zaka dawo da ita zaice aah gwara ayi a gama a tafi sai wani lokacin dawowa in yayi Kuma azo ayi amma ba zai taba yarda a bar asibiti baa yi ba.

Moderator: So kana ganin Ko yaushe akwai rigakafin nan a asibitin nan Kenan?

Respondent: Ko yaushe akwai.

Moderator: A ganin ka su waye suke ma jinjiraye rigakafin idan haife su a asibiti?

Respondent: likitocin.

Moderator: Ok, su likitocin su suke Yi.

Respondent: Eh, su suke Yi abunsu.

Moderator: Kana ga kaman akwai wasu matsaloli da ake samu dake hana yara kanan da aka haife su jarirai su samu rigakafin ciwon hanta cikin awa ashirin da hudu (24hours) da haihuwan yaro?

Respondent: Toh duk dai mun riga munyi ma mutane gargadin cewa dan Allah idan har mace ta haihu a gida ayi saurin kawo ta asibitin don ayi wannan rigakafin.

Moderator: A matsayin ka na mai anguwan wannan gefen, akwai wasu labari da mutanen da suke kasan ka suka fada ma game da rigakafin ciwon hanta da akeyi da zarran an haihu?

Respondent: Gaskiya sai godiya baa taba ba.

Moderator: Yanda kake gani, Me kake ganin mutanen ka suka fahimta game da rigakafin ciwon hanta da zarran an haihu?

Respondent: Toh sun dauke shi abu mai muhimmanci.

Moderator: Kana ganin suna kawo matan su asibiti su haihu ayi musu rigakafin kuma wanda basu haihu a asibitin ba suma suna bukatan su kawo matan da yaran sabida ayi allurar rigakafin?

Respondent: Suma mata sufi gane wa yanzu suzo asibitin su haihu ana basu kulawa mai kyau da Kuma hada su sabulu dasu klin sabida tasan idan ta haihu a gidan ba lalle ta samu ba.

Moderator:Toh Mai anguwa kana da wata shawara da zaka bamu ta yadda zaa inganta rigakafin da zarran an haihu a wannan anguwan ka?

Respondent: Eh gaskia yanda kuka zo kukayi mana munji dadi, har yanzu roko muke akan Ku yanda kuka zo kuka taimaka mana har yanzu taimakon muke nema Dan Allah kadda maganin ya yanke mana a asibitin sabida yanzu idan mutum yazo da matar sa da yaron aka ce baayi ma yaron allurar ba zaayi iya fada da shi.

Moderator: Yanzu ace ana rigakafin ciwon hanta acikin yaran da aka haifa cikin Kashi tamani (80) ana musu rigakafin na ciwon hanta wace hanya ce zamu bi ya zama ya koma dari (100) da zarran an haife su ace anyi musu daga kashi tamani ya koma kashi dari?

Respondent: Akan abunda nace zamu koma kan gargadi da jawo hankali nan ne zamu samu muyi covering da abunda muke so din ya samu.

Moderator: Kaman cikin al'umma, cikin gari ya kake gani zaa taimaka ya bunkasa daga tamanin din ya koma dari?

Respondent:Yanzu Misaki ka gaya min naji dadi haka zanje na fada ma wani shima yaji dadi haka zai fada ma wani cewa "Toh Dan Allah matar ka kar ta haihu a gida ta haihu a asibiti sabida kare abin da muke tsoro ya faru watau cutar ciwon hanta".

Moderator: A yanzu ganin ka mene ne asibiti zatayi da zamar daga tamanin ya koma dari?

Respondent: Har yanzu dai yanda yake yi yayi tsayin daka ya jawo mu kuma yake cigaba da jawo mutane da suke zuwa asibitin ya cigaba da kokarin in Allah ya yarda zaa samu yayi covering yanda duk ake nema.

Moderator: So a matakin maaikatan asibitin wani shawara ne zaka basu da cewa daga 80% din ya koma 100%?

Respondent: Har yanzu dai su din ma su su rinka jawo hankalin matan da suke zuwa awo muma mazajen duk a hada ayi mana.

Moderator: A ganin ka ta wani hanya zaa bi a wayarwa mutane kai ta wajen isar musu da saqo a sauwaqe.?

Respondent: Munada wanda yake mana shela idan an tura mana saqo irin haka muna neman masu shela su zo anguwanni suyi shela kan kaza da kaza duk dai akan abinda ake son isar ma jamaa su sunayi.

Moderator: Banda shela, Shin akwai wani hanya da kake ganin zai inganta zuwa wajen isar da saqon?

Respondent: Ehto gamu nan, muma muna iya kokarin mu wajen isar saqo.

Moderator: Zaka iya gayamin ko wani masaniyar ka kan rigakafin da ake wa mata masu ciki na tetanus?

Respondent: Anayi musu. Don shima ana gargadi ana cewa ko qusa ko kwano ko Kuma aah wuqa ta taba yanka mace idan har batayi wani abu watakila zai iya zuwa ya shafi abin da yake cikin ta ya samu matsala don haka abunda yasa ake wannan allurar.

Moderator: So kana ganin akwai matsalolin dake sa mata basu samu wannan rigakafin ba?

Respondent: Idan har Allah yasa mace tazo anan an yi mata bayani babu gardama anayi musu.

Moderator: Kana ga akwai nasara da aka samu wajen aiyuka da akeyi?

Respondent: Sosai ma. Kamar awo idan sunzo muna ganin nasara tare da su.

Moderator: A matsayin ka na mai anguwa wanda asibitin nan take anguwar ka, wasu abubuwa ne wanda aka kawo a wannan asibitin ya amfani al'umma sosai?

Respondent: Toh gaskiya abubuwa da yawa. Kaman yanda Na fada ma babu abunda baayi mana ba ga ruwa an kawo mudai asibitin nan Sai dai godiya.

Moderator: Cikin abubuwan da aka kawo wannan yafi amfanan al'umma?

Respondent:Kome ba zaace bai amfana ba.

Moderator:A ganin ka akwai wani abu da zaka ilimantar damu game da ciwon hanta mussaman rigakafin da akeyi ma yara da zarran an haife su, wani ilimi da zaka kare mu dashi?

Respondent:Toh kaman dai yanda na roka shine dan Allah acigaba da kawo mana magani dan muna jin dadi wannan Mungode da ku kawo mana ziyara Domin duba abunda akeyi Toh kome anayi, Alhamdulillah.

HAUSA TRANSLATION FOR NOTE TAKING TEMPLATE FOR PREGNANT WOMEN KII GUIDE State 1, LGA 3, Facility 2

(KII NA MATA MASU JUNA BIYU/ MATA MASU CIKI)

Jadawallin bayanan wacce ake ma tambaya

Shekarun ki nawa? 20

Tambayoyi:

1. Me kika sani game da Ciwon Hanta/ shawara?

   Abinda na farinta a ciwon hanta shine yake kawomana damuna a cikin ciki. Shine yake sa muna zuwa asubiti adubamana batun hanta

2. Me kika sani game da allurar Rigakafin ciwon Hanta/ shawara da ake yiwa yara dazaran an haifesu?

   Rigakafi yana da kyau a asubiti saboda in muka Haifa muka zo muna samun rigakafi da magunguna wanda zai taimakesu da lafiya

   ----

   2a. Meye ra'ayinki akan ita wannan allular rigakafin ciwon Hanta/ shawara da ake yiwa yara da zarar an haifesu?

   ----yana taimakon su ---suna samun lafiya a jikin su kuma yana basu kwari----dun insuna da damuwa---

   2b. Shin a baya kin taba neman a yi ma yaro/ yarinyarki alular rigakafin ciwon Hanta/ shawara da akeyi da zaran haihuwa?

   -----------ee yaron da na Haifa, yaro daya aka yi mishi --

3. Shin Kina da shirin bada yaro/yarinyar ki ayi masa/mata allular rigafin ciwon Hanta/ shawara da zaran kin haihu?

   -------ee ina da raayi ayiwa yarona --

   3a. Meyasa zaki amince? Meyasa bazaki amince ba)?

----saboda yana da taimako in aka bar yaran mu da su----------------------
----

3b. Shin Kina da wani ra'ayi, ko damuwa, ko d'oki, akan ita wanan allular? Meyasa?

----

4. Shin Kina ganin wannan allular regafin ciwon Hanta/ shawara da zaran an hahihu na iya kare yaro/yarinyar ki daga kamuwa da ciwon Hanta/ shawara? Meyasa kika amince? Meyasa baki amince ba)?

Ee yana hanawa. Magunguna da ake bamu yana kwantar da ciwon hanta

---

----

5. Shin kina ganin zaki samu ayiwa yaro/yarinyar ki allurar rigakafin ciwon Hanta/shawara Idan kika nema da zarar kin haifu?
   -----baya bada wahala a asubiti---

   5a. Zaki nemi a yi ma yaro/yarinyar ki?
   ---------------ee..zan bukata--

   5b. Me ya sa kika fadi haka?
   ----saboda yaro in ya gira da shi akwai matsala. Yaronka bazaka gane lafiyan shi ba. Ciki yana kumbura---
   ----

   5c. shin kina da wani tunani ko matsala da Kike ganin zai iya hana ki samun wannan allurar ta ciwon Hanta/Shawara da ake badawa da zarar haihuwa?
   --babu wata matsala---

   ----

6. Shin Kina gananin wannan allular rigakafin da ake yi da zarar haihuwa ta ciwon shawara/Hanta tana da amfani ga yaro/yarinyarki? (Meyasa kika amince? Meyasa baki amince ba)?
-ee yana da amfani. Lokacinda da na haifi yaro/ko yarinya -------------------------
----
----
7. A jumlace shin Kina ganin cewa mata masu juna biyu a nan yankin na samun ita wannan allurar rigakafin ciwon shawara/Hanta da zaran sun haihu?
-----------suna samu ayankinnan kam. Amma ba duka ba------------------------------
----

7a. Me ya baki wannnan tabbacin (ko rashinsa)?

--------wani raayin shi daban daban ---

----

8. Shin kina da wata shawara da za ki iya bamu ta yadda zamu kara wayar ma alumma da sauran jama'ar wannan yanki game da muhimmancin allular rigakafin cutar shawara/Hanta da ake badawa da zarar haihuwa?
---shawara na ina rokon mu mu mata idan muka haihu mukawo yaran mu amusu rigakafi tunda dai rigakafi ba da kudi ba---
----

8a. shin zaki so k'arin sani game da ciwon Hanta/Shawara? (Ta hanyar Labarai, vidio/majigi, fosta, da sauransu)

Hanyanda yafi isar da sako TV, labarai---

8b. Ta wace hanya kika fi son Karin ilimi game da sha'anin kiwon lafiya?

Yanyoyi dayawa, wani daga gidama muna samun -----------------------------
----
----

9. Shin akwai wani abu da kike tunanin ya kamata mu sani game da wanan ciwon, ko hanyar yada bayanai akanta ko gameda allurar rigakafinta?

-babu---

State 1, LGA 3, Facility 3

Moderator: Good morning, Sir!

Respondent: Good morning!

Moderator: My name is [name]. With me here is my colleague, [name]. We work for CDC-AFENET. We are doing an assessment about vaccines in [State name]. [State name] has approved this assessment.

We are here today to talk with health care providers and community health care workers about their experience and views about antenatal care, vaccines for pregnant women and newborns, and your ideas about how Ministry of Health can do a better job reaching all pregnant women and children with vaccines. You are being asked to participate because of your knowledge of maternal and/or child health.

If you agree to participate, we would like to ask you a few questions about the services provided in your facility for Hepatitis B birth dose to newborns and Tetanus diphtheria to pregnant women. This questionnaire will take about 30 minutes to complete. The questions will cover a range of topics including immunization services, policies around Hepatitis B birth dose and Tetanus diphtheria administration, vaccine, and data management and, trainings.

There are no risks or direct benefits to participants for participating in this interview. If you choose not to be interviewed, you will not be affected in any way. We will not use your name in any analysis and your name will not be presented with your answers to our questions. If you have any questions about your rights, this assessment or anything else, please contact the State Immunization Officer.

Do you have any questions about this assessment?

Respondent: No, you may go ahead.

Moderator: May I know your age?

Respondent: 33

Moderator: Your Designation?

Respondent: Medical Officer

Moderator: Alright, now, what do you think are the biggest challenges for people living with hepatitis B in your community?

Respondent: Well, I have been in this facility for the past 3 month. I have seen a quite a lot of patients with hep-B and to my surprise, most of them are not even aware they have the infection.

So I think there is lack of awareness. People do not know what hepatitis I, and I know this is a rural setting so once you see a patient try and educate them more Know what the disease is, okay another challenge we are facing is the investigation, normally when you see someone with hepatitis b virus, you try and do what we called combo-test. Okay after you have done that will be able to tell you whether the patient has an anti- body, from there you proceed to do the test like LFT, plain abdominal USS emphasis on the liver, if the liver is badly damage with the derange value from the LFT. Then you do the fibroid scan which I doubt if there is any place in Adamawa state that has the fibroid scanning machine. May be Adamawa German hospital. So it is actually very cumbersome. The only thing we do, maybe we can scan and then pick liver cirrhosis, may be LFT we can see the deranged value. Whenever we send them over to go to the state capital that is near Yola/Jimeta to go and get adequate care most of them turn it down due to distance, financial constraint and before you know it, the next time you are going to see the patients they are already in decompensated chronic liver disease. And is already gone too far. Its quite pathetic. We are going to appreciate more effort to adequately create awareness to those who should be vaccinated. Is like I said, I know we have the vaccine here, I only just resume about 3 months ago. I know this is something that I have spoken before and we are trying to see how we can address the issue of people having the vaccine. I have spoken with the PMO we are going to make it prerequisite that any patients that come to us, we will write viral markers such as RVS, HCV, HBSA once we see someone HBS negative, HCV negative and RVS negative. There is no vaccine for HCV but for the hepatitis B we can start the vaccine.

Moderator: What are the biggest needs to address hepatitis B in your community?

Respondent: Basically, that means we are going for the investigation like I said the only thing we can do here is the USS and may be LFT. So, if we can have a center that has some of the facilities, equipment, that we can use so that we can check the liver adequately and you know it is not enough, there is issue of stable power too and may be the provision of the vaccine as well

Moderator: Is there a strong need for the hepatitis B birth dose? (Why or why not?)

Respondent: Because of what we eat like I told you the body need too much. There is one pregnant woman that come yesterday, she was with jaundice all through, in fact, there was ascites and then there was pregnancy, this could lead to preterm labour.

Moderator: Do others see a need for the hepatitis B birth dose?

Respondent: Of course they do, as I earlier mentioned I spoke with the PMO. At the community level, most people they don't know about hep-B is so I have been like have to see them and them and educating them like you have to combine and you can see a lot of patients like 100. So the manpower is something else. It is not every patient that you need to start educating about hep-B, so it really is going to take a lot of energy and time to achieve that.

Moderator: Can you tell me about the current programs or practices for the hepatitis B birth dose in your clinic?

Respondent: No, presently no. I am not aware but that of HIV I think we have

Moderator: Is the hepatitis B birth dose easy to get within 24 hours of birth? (Why or why not?)

Respondent: I don't think so, seriously, I don't, perhaps the availability of the vaccine birth dose

Moderator: Who can administer the hepatitis B birth dose?

Respondent: We have the nurse is a woman in charge of ANC and we also have the midwife they were all thought how to do it

Moderator: What are challenges/barriers to getting the hep B birth dose vaccine?

Respondent: I think that will be best if there been interviewed people that administer the vaccine because as long as I have been working here, I don't think there is any issue with respect to that because if there is, they would have told me

Moderator: Have you gotten information from participants regarding their experiences with the hepatitis B birth dose?

Respondent: No

Moderator: What is the community understanding of the hepatitis B birth dose?

Respondent: Ok yeah, you know normally we don't have any issue much issue with the one giving immediately at birth. But the subsequent ones after 6 weeks, 10weeks, 6 months and the rest. This is when they start this fallacy that ones they give them thus vaccine they may be the child will not be able to conceive. But the one at birth people don't have problem with that

Moderator: Are people interested in getting the hepatitis B birth dose?

Respondent: Yes, they are.

Moderator: Can you describe what kind of specific information/messaging you have heard?

Yes, they often claim this is another way to stop us from reproduction by the white man and we should not allow our children to be vaccinated. In fact, I have seen some one last two months when I came here she put to bed, a Fulani woman, in fact she said that the nurses should not touch her child and funny enough she had a C/S, that mean she was weak so she could not breast feed the child immediately so we wanted to just put some fluid for the child 10% d/w so that the child will not get into hypoglycemia after doing RBS, but she refused. She claimed she was told that if they give the child injection if the child grows up he will not be able have children. So they

don’t want that one, they want to go home with their child and be giving him “fura da nono” instead.

Moderator: Do you have any ideas or suggestions for improving hepatitis B birth dose vaccination rates?

Respondent: The major problem is manpower, ok if the incoming organization can get some someone that will work on daily basis maybe we will give that person a time, because most of our vaccines are taking in the morning, or if there will be something that will motivate that person like have a station and ensure that there is adequate vaccine on ground, then ensure that, in fact the office should be very close to the labor ward so that any child they will be given birth to will be attended immediately. (1) availability of the vaccine (2) the manpower I was talking about (3) then may be health education to the mothers that are pregnant that are near term whenever they come for ANC if they will have a good health talk about the important of all these vaccines, I think that will go a long way in the acceptance of the vaccine.

Moderator: At the community level?

Respondent: It will also go a long way if TBA organization can bear with them, ok all these TBA about 25% of the birth in this hospital are coming from outside. I can beat my chest and tell that most of them like to deliver in the hospital but we still have like 25% that usually deliver in the hospital, they only come to the hospital when they have complications. So, if we can those people may be get the attention of the royal fathers and then maybe he will send his town criers to inform people there is a gathering and the TBA are also educated about the vaccine so that whenever they conduct delivery they take to the hospital so that they can get vaccine

Moderator: Are there specific strategies for education or awareness that might be most effective?

Respondent: Health education during ANC, about 25% of delivery are in this facility so we can capture a lot of pregnant woman. You see if you are pregnant, you are expecting a baby very soon in a week or two. Once this baby is born either at home or hospital ensure that this baby has giving injection because of work load the nurses can forget about the inject just reminded this baby has not been giving any injection so that he can receive the hep-BD at birth and the other related vaccine. The royal fathers as well, that one can capture the TBA, they know them self, they communicate, get one or their representative, sit them down, discus these findings with them so that it’s okay for them to conduct. Because you know most of them will feel threaten and they will to carry their clients away but you will tell them that no, I think there was an outreach that was done last year that most of them were summoned and they were told how to carry out an aseptic procedure in child birth and it help them a lot so if you can just have got one of them gather them tell them and tell them that you doing a good job and you have received

a thousand deliveries before the advent of hospital. Yes, you can also do this. Once you deliver a woman let them bring the child there is an injection that we give.

Moderator: Can you tell me about your experience with the maternal tetanus vaccination?

Respondent: Yes, we have been capturing them a lot, like any woman that comes for antenatal care there are five doses, so the T1 is at first contact so most of them with focus antenatal care, most of them will come may be in 7th month in this rural setting. What I normally do is that this is your first T1 you're your T2 will come after 4 weeks, write the date on the card and come back even after delivery and T3 after 6 months you're your T4 is one year and T5 after one year. I apply it with their card. I have seen someone that received T1 and T2 even though I'm 3 months old in the facility.

Moderator: What barriers or challenges have you experienced related to the maternal tetanus vaccination?

Respondent: Most of them are coming from far places. Some come from as far as Gurin. Most of them if you tell them to come after four weeks and then they don't feel anything, the husband will tell you he doesn't have money. So, financial constraint and lack of proper education, illiteracy. They assume that if you are pregnant you only come to hospital if you have fever or headache. But I know two women that come for their second TD.

Moderator: What successes have you experienced, what is working?

Respondent: So far so good. Because of the time I spent here is not much

Moderator: What types of health interventions have been the most successful in your community and why have they been so successful?

Respondent: RVS, HIV we have CHEMONICS they are doing a wonderful job here ranging from health education, counselling, the availability ARV, I have a lot of them that have treatment failure. support for those that don't have money. Training of my staff just last week a representative from the lab. They lodge them in a hotel, fed them and train them and they came and gave us feedback. Last 2weeks they trained a midwife and my self was also train on PMTCT. The training should be done from time to time

Moderator: Is there anything else you think we should know about hepatitis B, sharing information about hepatitis B, or the hepatitis B birth dose?

Respondent: I think I have said it all. This program is welcome. We appreciate you guys will come sooner. We can use this opportunity to vaccinate a lot of children at birth which will prevent it spread. Like for the other people that have a family member with hep B. if there is vaccination,

there is no way people will get it. The spread can be curtail. We hope we are going to have an organization like CHEMONICS

Moderator: Thank you very much for your time.

HAUSA TRANSLATION FOR NOTE TAKING TEMPLATE FOR PREGNANT WOMEN KII GUIDE State 1, LGA 3, Facility 3

(KII NA MATA MASU JUNA BIYU/ MATA MASU CIKI)

Jadawallin bayanan wacce ake ma tambaya

Shekarun ki nawa? 27years

Tambayoyi:

1. Me kika sani game da Ciwon Hanta/ shawara?

   Inajidai ana maganshi, baitaba samun dan uwana ba, yana da illa sosai, yana kawo damuwa. Ana samun magungunansa yanzu haka ko? Zan ansa miki tambayoyin ki in mungama wannan tattaunawa

2. Me kika sani game da allurar Rigakafin ciwon Hanta/ shawara da ake yiwa yara dazaran an haifesu?

   Yana da muhimmanci sosai, saboda kare lafiyan yaran mu da mu din ma gaba daya

   ----

   2a. Meye ra'ayinki akan ita wannan allular rigakafin ciwon Hanta/ shawara da ake yiwa yara da zarar an haifesu?

   ------muna -godiya ga gwamnati- sosai Allah ya saka da alkhairi da ya tuna haka. Mun gode sosai- --

   2b. Shin a baya kin taba neman a yi ma yaro/ yarinyarki alular rigakafin ciwon Hanta/ shawara da akeyi da zaran haihuwa?

   ---------------muna kawo su ana musu. Dazaran an haifesu. Kuma anayi musu shi--

3. Shin Kina da shirin bada yaro/yarinyar ki ayi masa/mata allular rigafin ciwon Hanta/ shawara da zaran kin haihu?

   ------------------------insha Allahu, da haihuwa da yinshi in Allah ya yarda--

   3a. Meyasa zaki amince? Meyasa bazaki amince ba)?

-----sabo da kare lafiyanta, nan gaba. insha Allah--------------------------------
----

3b. Shin Kina da wani ra'ayi, ko damuwa, ko d'oki, akan ita wanan allular? Meyasa?

Alhamdulillah sosai, munji dadin wannan taimako. Munji dadin shi sosai

----

4. Shin Kina ganin wannan allular regafin ciwon Hanta/ shawara da zaran an hahihu na iya kare yaro/yarinyar ki daga kamuwa da ciwon Hanta/ shawara? Meyasa kika amince? Meyasa baki amince ba)?

Yana kare su sosai, doncututukan ba Kaman da ba sun ragu ajikin yara

----

5. Shin kina ganin zaki samu ayiwa yaro/yarinyar ki allurar rigakafin ciwon Hanta/shawara Idan kika nema da zarar kin haifu?
   ----insha Allahu tunda akwai a asubitotinmu kam. Muna samun shi------------
   ----

   5a. Zaki nemi a yi ma yaro/yarinyar ki?
   -----insha Allahu, zai sami lafiya ingantacce---

   5b. Me ya sa kika fadi haka?
   ---saboda in kayiwa yaron ka taimaki kanka da yaron ka -------------------
   ----

   5c. shin kina da wani tunani ko matsala da Kike ganin zai iya hana ki samun wannan allurar ta ciwon Hanta/Shawara da ake badawa da zarar haihuwa?
   ------------------------- ---

   -

6. Shin Kina gananin wannan allular rigakafin da ake yi da zarar haihuwa ta ciwon shawara/Hanta tana da amfani ga yaro/yarinyarki? (Meyasa kika amince? Meyasa baki amince ba)?
----------inkayima ya ron ka taimakeshi ---

----
7. A jumlace shin Kina ganin cewa mata masu juna biyu a nan yankin na samun ita wannan allurar rigakafin ciwon shawara/Hanta da zaran sun haihu?
-------ee… muna kokarin fitowa. In a asubitin akayi haihuwanma ana kawoshi koma agida da zaran an haifeshi---
----

7a. Me ya baki wannnan tabbacin (ko rashinsa)?

-----inkazo ofishin zaka mata acike da yara --

----

8. Shin kina da wata shawara da za ki iya bamu ta yadda zamu kara wayar ma alumma da sauran jama'ar wannan yanki game da muhimmancin allular rigakafin cutar shawara/Hanta da ake badawa da zarar haihuwa?

Shawara dai guda yadane. –muna godiya gareku da kuke kawomana maguguna.
Adinga sanar wa a rediyo, telebijin, --

8a. shin zaki so k'arin sani game da ciwon Hanta/Shawara? (Ta hanyar Labarai, vidio/majigi, fosta, da sauransu)

--duka hanyoyin suna da sauki. Amma redio da telibijin don yanzu mutane sunfi mayar da hankali akai--
----

8b. Ta wace hanya kika fi son Karin ilimi game da sha'anin kiwon lafiya?

--ina shigeshigen intanet din ma. Sannan kuma inna sauraron radio da telibijin---

9. Shin akwai wani abu da kike tunanin ya kamata mu sani game da wanan ciwon, ko hanyar yada bayanai akanta ko gameda allurar rigakafinta?
--kunga ku manyan likitocine kunsan komai..muna rokon ku dai ku kara kokari. Mun gode sosai---

Note taking template for Health care worker KII guide State 1, LGA 3, Facility 4

Demographic information of interviewee

Age:25

Sex: F

Designation: MIDWIFE

Questions

1. What are the biggest challenges for people living with hepatitis B in your community?

____Isolation….isolating them

2. What are the biggest needs to address hepatitis B in your community?

We are telling them it will affect their life and also

3a. Is there a strong need for the hepatitis B birth dose? (Why or why not?)

yes now….Because is good. So that they will not be affected with the disease

3b. Do others see a need for the hepatitis B birth dose?

________yes…we are counselling them about the disease before giving birth

4.Can you tell me about the current programs or practices for the hepatitis B birth dose in your clinic?

______MANCHIW which was started recently

____
_

4a. Is the hepatitis B birth dose easy to get within 24 hours of birth? (Why or why not?)

_______not always. Some times within 2 weeks because we don't have cold chain, we don't have solar..we get ours vaccines from the secretariate____
____
____
___

4b. Who can administer the hepatitis B birth dose?

___________RI incharge

CHO

JCHEW

CHEW

MIDWIFE____
____
____

4c. What are challenges/barriers to getting the hep B birth dose vaccine?

_____lack of the cold chain
____
____
____

5. Have you gotten information from participants regarding their experiences with the hepatitis B birth dose?

____no any information
____
____
____

5a. What is the community understanding of the hepatitis B birth dose?

They are understanding. We are giving them to prevent their child from catching the disease____

____

____

______

5b. Are people interested in getting the hepatitis B birth dose?

______yes including those delivered at home____

____

____

5c. Can you describe what kind of specific information/messaging you have heard?

_____No____

____

____

___

6. Do you have any ideas or suggestions for improving hepatitis B birth dose vaccination rates?

_______yes…provision of the cold chain so that within 24hrs after birth we should give it. Some people are from village if they go back they will not able to come. After measles and yollow fever vaccine we use to give them net (mosquito) which will make them to come and deliver in the hospital, but now its not available____

____

____

6ai. At the community level

___community mobilization

____

____

____

6aii. Health care system level

May be they will organize an outreach and counselling them they will come

____

____

____

6aiii. Provider level

__no idea

____

____

____

6b. Are there specific strategies for education or awareness that might be most effective?

No

____

____

____

7. Can you tell me about your experience with the maternal tetanus vaccination?

#Some they will not come back to complete their doses____

____

____

_____

7a. What barriers or challenges have you experienced related to the maternal tetanus vaccination?

_may be their understanding is importance during pregnancy only, but still we are encouraging them to complete (hausa ”gani suke don ciki akayi shi in sun haihu baza su dawo ba. Gani suke amfanin shi aciki ne)____

____

____

__

7b. What successes have you experienced, what is working?

_during ANC muna kara gaya musu amfanin shi, biyar ne kawai ne kawai, wassu suna dawo wa
____
____
____

8. What types of health interventions have been the most successful in your community and why have they been so successful?

____

_toh..may be growth monitoring. PBF was successful. “ana basu free abubuwa. Mace tana haihuwa free komai, everything. Sai yazama daga mace tayi ciki suna zuwa complete har su haihu”____
____
_____

9. Is there anything else you think we should know about hepatitis B, sharing information about hepatitis B, or the hepatitis B birth dose?

____NO____
____
____
___

HAUSA TRANSLATION FOR NOTE TAKING TEMPLATE FOR PREGNANT WOMEN KII GUIDE State 1, LGA 3, Facility 4

(KII NA MATA MASU JUNA BIYU/ MATA MASU CIKI)

Jadawallin bayanan wacce ake ma tambaya

Shekarun ki nawa? 32

Tambayoyi:

Moderator: Me kika sani game da Ciwon Hanta/ shawara?

Respondent: A takaice, bisa ga yadda su ma'aikatan lafiya suke mana bayani akan yana da illoli kala kala. Kuma suna mana bayani game da abunda zamu kare lafiyar kammu daga gare shi

Moderator: Me kika sani game da allurar Rigakafin ciwon Hanta/ shawara da ake yiwa yara dazaran an haifesu?

Respondent: Suna gaya mana da zarar in mace ta haihu, musamman in a asibiti ne, ana yima yaro allura kafun a tafi gida.

Moderator: Akwai wani Karin bayani game da ita wannan rigakafin?

Respondent: Tana da maturar amfani da kuma taimako sosai. In ana ma yaro rigakafi zakaga yana cikin koshin lafiya.

Moderator: Yaranki nawa?

Respondent: Yarana biyar

Moderator: Shin a baya kin taba neman a yi ma yaro/ yarinyarki alular rigakafin ciwon Hanta/ shawara da akeyi da zaran haihuwa?

Respondent: Eh. Duk yarana an yi musu. Babu wanda ba'a masa ba.

Moderator: Shin Kina da shirin bada yaro/yarinyar ki ayi masa/mata allular rigafin ciwon Hanta/ shawara da zaran kin haihu?

Respondent: In Allah Ya yadda

Moderator: Meyasa zaki amince?

Respondent: Don naga muhimmancinsa da kuma amfaninsa a gun sauran 'ya'yan nawa

Moderator: Shin Kina da wani ra'ayi, ko damuwa, ko d'oki, akan ita wanan allular?

Respondent: Eh to, babu wani illa ko damuwa dangane da yenta, sai dais au daya da aka yi ma yaro na sai gurin allurer ya kumbura. Da ya kumbura, naje gida sai baban sa yayi fada. To daga baya kuma da muka dawo muka samu ita ma'aikaciya lafiya muka yi mata bayani sai ta samo kankara ta goga mana, daga na sai kumburin ya sauka. Da na koma gida sai baban ya yadda naci ci gaba da kawo yaron.

Moderator: Shin Kina ganin wannan allular regafin ciwon Hanta/ shawara da zaran an hahihu na iya kare yaro/yarinyar ki daga kamuwa da ciwon Hanta/ shawara?

Respondent: Eh! Yana karewa.

Moderator: Meyasa kika amince?

Respondent: Toh, game da nawa yaran, ni dai abunda na gani Kenan. Bansan na wasun ba.

Moderator: Shin kina ganin zaki samu ayiwa yaro/yarinyar ki allurar rigakafin ciwon Hanta/shawara Idan kika nema da zarar kin haifu?

Respondent: Eh. ana samu ba bu matsala.

5a. Zaki nemi a yi ma yaro/yarinyar ki?

Respondent: Eh zan nema ko in Allah Ya yarda in dai akwai dama

Moderator: Me ya sa kika fadi haka?

Respondent: Sabili da wancan karon nayi kuma naga amfani. Yanzuma kuma ina son in nemi kariya

Moderator: Sau nawa ake yin wannan rigakafin?

Respondent: A'a. Bansan ko sau nawa akeyi ba.

Moderator: Shin kina da wani tunani ko matsala da Kike ganin zai iya hana ki samun wannan allurar ta ciwon Hanta/Shawara da ake badawa da zarar haihuwa?

Respondent: A'a ba'a samun wata matsala. In dai ka taho kam zaka samu. In dai akwai kam ana yi ma yara shi. Daga nan har sheka ana yima 'ya'ya bayan watanni.

Moderator: Shin Kina gananin wannan allular rigakafin da ake yi da zarar haihuwa ta ciwon shawara/Hanta tana da amfani ga yaro/yarinyarki? Shin yana hana yara kamuwa da cutar?

Respondent: Ee yana hanawa.

Moderator: Me yasa kika fadi hakan?

Respondent: Toh game da nawa luran, akan yara namkenan da kuma yara makwabta da suka karba, ya hana su kamuwa da cutar. Bansan na wasun ba. In mutane suna zuwa suna kai yara ana musu rigakafi, basa samun illoli game da damuwan kananan cututtuka haka.

Moderator: A ganinki in kika bukaci wannan allurar rigakafi da ake badawa da zarar haihuwa na cutar hanta. Kina ga zaki iya samu?

Respondent: in Allah Ya yadda.

Moderator: Ana bada ta a asibiti?

Respondent: Eh. A na bayarwa a cikin wannan asibitin.

Moderator: So, zaki bukata?

Respondent: Daga zarar mace ta haihu ma a asibiti kafun ta koma gida, za a diga ma yaron maganin a baki, sannan za ayi ita wannan rigakafin ciwon hanta, da kuma wata wacce ake kira allurar BCG.

Moderator: Me yasa kikace zaki bukata?

Respondent: Saboda naga amfaninta a kan yara na. kuma akwai bambanci tsakanin yaro da ake ma rigakafi da kuma wand aba a yi mar.

Moderator: Kina ga akwai wani akasi da ake iya samu wurin neman wannan rigakafi na cuta hanta wacce ake bayarwa da zarar haihuwa?

Respondent: Eh to, wani lokaci akan samu. Kasan suna yin rigakafin ne ranar Laraba, to misalign idan mata tazo rana alhamis ne sannan ta haihu, kaga baza ta samu a ranar ba, sai dai ace ta dawo ranar laraba din. Amma in akayi sa'a akazo bayan laraba din, kuma wanda akayi amfani dashi ranar laraba din ya rage, to ana yi mana shi, in yana cikin sanyinsa.

Moderator: Kina nufin in akayi haihuwa ba ranar rigakafi ba yana yiwuwa baza'a a samu wannan rigakafin ba?

Respondent: Eh. Wani lokacin kuma ana iya samu.

Moderator: Shin Kina ganin cewa mata masu juna biyu a nan yankin na samun ita wannan allurar rigakafin ciwon shawara/Hanta da zaran sun haihu?

Respondent: Ana yi. Balle yanzu kai ya waye.

Moderator: Ko da wadanda sukayi haihuwan gida?

Respondent: Eh. Koda haihuwan gida ne, suna zuwa.

Moderator: Me ya baki wannnan tabbacin?

Respondent: Saboda muna zuwa tare da su. Muna ganinsu suna zuwa, kuma muna haduwa da su. Ko basuzo suka haihu a asibiti su kan kawo yaransu asibiti ana musu rigakafi.

Moderator: Shin kina da wata shawara da za ki iya bamu ta yadda zamu kara wayar ma alumma da sauran jama'ar wannan yanki game da muhimmancin allular rigakafin cutar shawara/Hanta da ake badawa da zarar haihuwa?

Respondent: Eh to. Akwai guda daya da nake son in bayar. Tunda mutane suna yawan kawowa haihuwa a asibiti, ya kamata kar a dinga rabuwa da ita wannar allurar kuma ana son daga haihuwa ayishi toh in anzo lokacin bai kama daidai lokacin da akeyin shi ba, awon din ba in an kawo Magani kuma wani lokacin baa samun allurar sai ace sai wani bayani lokacin kaza ace a dauko yaron lokacin kaza ayi masa allurar.

Moderator: Shin akwai wani Shawara?

Respondent: Na biyunsa kuma gomnati ta taimaka mana da magunguna kamar da yanda take bawa yara na taimako kamar su paracetamol din da ake bayarwa idan an yaro ba lafiya ya sha sabida zazzabi kafin a buga atafi a asibiti in anzo rigakafin zaa ana taimakawa ana bamu sabida yaran mu susha kafin ayi musu allurar ya rage musu zafin jiki da anayi, ana bayarwa amma yanzu baayi.

Game da mata muna da yawan da muna samun damuwa domin wani mijin bayida karfi. In mace zata zo awu, da maganin awunnan ana bayarwa kyauta, amma yanzu sai mun siya. So, wani mijin in har shi zai bada kudi domin siyan Magani, to kuwa ba za ki zo awun ba.m Ko da kuwa kina da damuwa a jikin ki baza ki samu zuwa awun ba in mijin bashi da shi. Don haka, gomnati ta taimaka, kamar yadda takeyi da ana bada magunguna kyauta a ci gaba da taimaka mana da wadannan magunguna. Ko da ba a bayar duka ba, a akamanta.   .

Moderator: Shin wani shawara zaki bamu game da ilimantar da mata ko alumma gaba daya wajen samun bayanai game da wannar rigakafin?

Respondent: Shi de riga kafi yana da muhimmanci. Amma idan anaso mutane su kara fahimtar hakan a ringa taimaka mana. Kaga irin su maganin zafin jiki wanda zamu ringa bawa yara in an bamu shi kyauta, zamu san kuna kula da mu, kuma kun san taimakon mu. Da kuma in muna da ciki ana bamu magunguna muna sha.

Moderator: Kina nufin in ana bada wadannan taimakon za a samu saukin ilmantadda jama'a?

Respondent: Za a samu sauki ilimantarwa. Domin kowa zaiji cewa ana bada magunguna kyauta a asibiti, sai kowa zaiyi marmarin zuwa.

Moderator: So, in suka zo sai a samu daman yi musu bayanai Kenan.

Respondent: Eh, ai dama in munzo asibiti akan yi mana bayanai na ilmantarwa

Moderator: To ta yaya kike son ki kara samun ilimi kan harkar rigakafin da zarar an haifi yaro?

Respondent: Ta hanya rediyo. Sau da yawa in ka bude rediyo zakaji ana fadakarwa. Amma fadakarwa na asibiti yafi gamsarwa

Moderator: To ta yaya kika fi son ki samu ilmantarwa dangane da sha'anin Ifiya baki daya?

Respondent: In nazo asibiti, zanyi dukkan bayanin abun da ke damuna, kuma za a sanadda ni abunda zanyi ya amfaneni

Moderator: Akwai wani abu na Karin bayani da zaki so mu sani game da ciwon hanta, ko kuma rigakafinta na da zarar haihuwa ko kuma hanyoyin yada bayanai akanta?

Respondent: Toh. Kamar karkara, irin wanda basu da asibiti ya kamata ana shi ana musu bayani kan muhimmancinsu.

Moderator: Mungode da wannan lokaci da kika bamu da kuma bayanai masu muhimmanci.

State 1, LGA 3, Facility 5

Moderator: Good afternoon, Ma!

Respondent: Good afternoon!

Moderator: My name is [name]. I work for CDC-AFENET. We are doing an assessment about vaccines in [state name]. [state name] has approved this assessment.

We are here today to talk with health care providers and community health care workers about their experience and views about antenatal care, vaccines for pregnant women and new-borns, and your ideas about how Ministry of Health can do a better job reaching all pregnant women and children with vaccines. You are being asked to participate because of your knowledge of maternal and/or child health.

If you agree to participate, we would like to ask you a few questions about the services provided in your facility for Hepatitis B birth dose to new-borns and Tetanus diphtheria to pregnant women. This questionnaire will take about 30 minutes to complete. The questions will cover a range of topics including immunization services, policies around Hepatitis B birth dose and Tetanus diphtheria administration, vaccine, and data management and, trainings.

There are no risks or direct benefits to participants for participating in this interview. If you choose not to be interviewed, you will not be affected in any way. We will not use your name in any analysis and your name will not be presented with your answers to our questions. If you have any questions about your rights, this assessment or anything else, please contact the State Immunization Officer.

Do you have any questions about this assessment?

Respondent: No, Sir. You are welcome.

Moderator: So do you agree to participate.

Respondent: Yes, Please.

Moderator: Ok. I have here with me, [name] who will assist in taking some notes.

Respondent: Ok. You are both welcome.

Moderator: May I know your age?

Respondent: 44 years

Moderator: Ok. So, what are the biggest challenges for people living with hepatitis B in your community?

Respondent: Challenges for people living with hepatitis B…Hepatitis B is a killer disease. Whenever someone is affected, the person will be frequently ill, sometime they present with oedema, abdominal swelling, and sometime they will be irritable.

Moderator: How does that affect how they live?

Respondent: They live with problems. They will always have to be buying drugs, and they will not be able to eat certain food but not others.

Moderator: What are the biggest needs to address hepatitis B in your community?

Respondent: people will have to take vaccines for Hepatitis B. We also need to educate people about the importance of the vaccine, and how to live in a health way to avoid getting infected. It is good to start since birth, so that we get optimal protection.

Moderator: So, why do you think this Hepatitis B birth dose is necessary?

Respondent: Because Hepatitis B is a communicable disease. So, if a woman give birth, in our tradition, every woman in the community will go and congratulate this mother for a successful delivery, and they usually like to hold the baby, and some of them might be infected with Hepatitis B without having any symptoms. Hence, when they hold the baby, they may infect it.

Moderator: So, do others see a need for the hepatitis B birth dose?

Respondent: Yes. Most people in this community are aware.

Moderator: Can you tell me about the current programs or practices for the hepatitis B birth dose in your clinic?

Respondent: Our Own birth dose in our Clinic as of now, we are giving hepatitis B within 14days but if the woman delivered at ANC health care at immunisation date we give the injection at birth.

Moderator: How can you ensure that child is vaccinated and you don't miss out that child?

Respondent: We are telling them at the ANC clinic days even if you don't deliver at the hospital on that day please bring in your child during the week for the Immunisation day and even if she delivers at the hospital and is not the day of immunisation we tell her to come back.

Moderator: So, is the hepatitis birth dose easy to get within 24hours of birth?

Respondent: Is easy to get because we are having solar freezing.

Moderator: So why do you think it is difficult for you to be giving them within the 24hours after birth, what make it difficult?

Respondent: The difficult there is that sometimes if the person that is working is not enough, if there is one person working at night and you are the only one person receiving delivery maybe there is another one waiting for you then the keys for the fridge is with the immunisation officer, this is the problems we sometimes have.

Moderator: So, have you gotten any information from the participants regarding their experiences with the hepatitis birth dose?

Respondent: Some are saying that their children are too young but some that come for antenatal days, they are already aware because we are educating them on the disease. So those that are coming to the clinic have no problem but those that are not attending the clinic because of their traditions or somehow are not being educated on the issue of the disease.

Moderator: What is the community understanding of the hepatitis B birth dose, do you think that the community understand it?

Respondent: Some members in the community understands it well but not all, only few.

Moderator: So, are people interested in getting the birth dose?

Respondent: Yes, some are interested.

Moderator: Can you prescribe what kind of information you have heard concerning it from the people of the community?

Respondent: The information I have heard from the people of the community is that the hepatitis is important since that the disease if somebody have it then it is not a durable disease.

Moderator: Do you have any idea or suggestions for improving hepatitis B birth dose vaccination rate, any information that you can give us or suggestions on how we can improve it?

Respondent: The suggestions is by advocating about hepatitis B and having dialogues with the community elders and women in groups it will also help.

Moderator: What you mentioned are all community levels, what about the health facility level?

Respondent: At the health facility level, we do educate the mothers, pregnant mothers most especially on the immunisation day. We are telling them at the ANC clinic days even if you don't deliver at the hospital on that day please bring in your child during the week for the immunisation day and even if she delivers at the hospital and is not the time of immunisation, we tell her the right day of the immunisation she will come back.

Moderator: So is the hepatitis birth dose easy to get within 24hrs of birth?

Respondent: Is easy to get because we are having solar freezing.

Moderator: So why do you think it is difficult for you to be giving them within 24hours after birth, what makes it difficult?

Respondent: The difficult there is that sometimes if the person that is working the staffs is not enough, if there's one person, let's just say at night and the person at night and you are the only person receiving delivery maybe there's another one waiting for you then the keys for the fridge is with the immunisation officer this is the problems sometimes we have!

Moderator: So, have you gotten any information from the participants regarding their experiences with the hepatitis birth dose?

Respondent: Some are saying that their children are too young but some that come for antenatal days they are already aware because we are educating them on that disease those that are coming to the clinic have no problem but those that are not attending the clinic because of their traditions or somehow.

Moderator: what is the community understanding of the hepatitis B birth dose, do you think the community understand it?

Respondent: Some members in the community understand it but not all.

Moderator: So are people interested in getting the birth dose?

Respondent: Yes, some are interested.

Moderator: Can you prescribe what kind of information you have heard concerning it from the people of the community?

Respondent: The information I have heard from the people of the community is that the hepatitis is important since that the disease if somebody have it is not a durable disease.

Moderator: Do you have any idea or suggestions for improving hepatitis B birth dose vaccination rate, any information that you can give us or suggestions on how we can improve it?

Respondent: The suggestion is by advocating about Hepatitis B and having dialogues with the community elders and women in groups it will help.

Moderator: What you mentioned are all community levels what about the health facility level?

Respondent: The health facility level, we will educate the mothers, pregnant mothers most especially on the immunisation day.

Moderator: So what of the at the provider level, the service provider, is there anything you can do regarding them so we can improve them?

Respondent: Yes, at the time of giving the vaccination, we also tell the mothers educating them that we are giving them these vaccines as it is important and have no problem it protects their child from hepatitis.

Moderator: Are there specific strategies for education or awareness that might be most effective?

Respondent: Communication along is also good, so that you hear what people think.

Moderator: Can you tell me about experience with the maternal tetanus vaccination?

Respondent: Women, some are trying but some are refusing.

Moderator: What barriers or challenges have you experienced related to the maternal tetanus vaccination?

Respondent: There's no barriers as of now, is only the pain that they hear that's why some women are refusing the vaccination.

Moderator: What successes have you experienced, what is working?

Respondent: If there is motivation, I know some that have said they have feel pains and they also receive and complete it. If to say that a woman come at the first contact, you know she is coming for her ANC and after 4weeks she comes back for her ANC also then at 6months some have delivered and they will not come back. If they see some are receiving motivation they will come back.

Moderator: What types of health interventions have been the most successful in your community and why have they been so successful?

Respondent: Receiving of the vitamin A and the worming tablets

Moderator: Is there anything else you think we should know about hepatitis B, sharing information about hepatitis B, or the hepatitis B birth dose?

Respondent: The only thing I will like to add is that we need to be sharing more information through mass media, community dialogues, etcetera.

Moderator: Thank you very much for your time.

HAUSA TRANSLATION FOR NOTE TAKING TEMPLATE FOR PREGNANT WOMEN KII GUIDE State 1, LGA 3, Facility 5

(KII NA MATA MASU JUNA BIYU/ MATA MASU CIKI)

Jadawallin bayanan wacce ake ma tambaya

Shekarun ki nawa? 27

Tambayoyi:

Moderator: Me kika sani game da Ciwon Hanta/ shawara?

Respondent: Cutar tana sa matsalar hanta ce

Moderator: Me kika sani game da allurar Rigakafin ciwon Hanta/ shawara da ake yiwa yara dazaran an haifesu?

Respondent: Rigakafinta yana da kyawu. Dukkan riga kafi ma yana da kyau domin kariya daga cututtuka.

Moderator: Bayan wannan akwai wani abu kuma da kika sani game da ita wannan riga kafi? Misali, suwa nene ake ba ma it?

Respondent: Akan bada ita ga me ciwo da kuma marar ciwo. Ko yaro ko babba, dukka ana iya yi musu.

Moderator: Meye ra'ayinki akan ita wannan allular rigakafin ciwon Hanta/ shawara da ake yiwa yara da zarar an haifesu?

Respondent: Ya kamata mutane su dinga karban ta

Moderator: Shin a baya kin taba neman a yi ma yaro/ yarinyarki alular rigakafin ciwon Hanta/ shawara da akeyi da zaran haihuwa?

Respondent: Eh. Akwai 'yata guda daya wacce na taba kai ta akayi mata

Moderator: Shin da akayi mata allurer riga kafin cutar hantar nan akwai Karin bayani da akayi miki ne?

Respondent: Eh. Anyi min bayanin cewa yana da kyau anayin shi wannan rigakafin kuma ni bayan anyi banga wata matsalar yin ba.

Moderator: Shin Kina da shirin bada yaro/yarinyar ki ayi masa/mata allular rigafin ciwon Hanta/ shawara da zaran kin haihu?

Respondent: ee ina da niyyar hakan

Moderator: Meyasa zaki amince?

Respondent: Sabida anyi min bayanin muhimmancinta

Moderator: Shin kinsan ko yaushe ne ake bada ita?

Respondent: bansan da yaushe ba ne a gaskiya?

Moderator: Baki taba tambayar ma'aikatan lafiya game da lokacin da ya kamata a bada wannan rigakafin cutar hanta ba bayan haihuwa?

Respondent: Ban taba ba.

Moderator: Me yasa baki tambaya ba?

Respondent: Kawai

Moderator: Shin Kina da wani ra'ayi, ko damuwa, ko d'oki, akan ita wanan allular?

Respondent: Bani da wata damuwa, tunda duk wani rigakafi in ya fito in dai aka sanar da mu ana zuwa ana yinshi zamu zo muyi domin mun gamsu da anfaninsu

Moderator: Shin Kina ganin wannan allular regafin ciwon Hanta/ shawara da zaran an hahihu na iya kare yaro/yarinyar ki daga kamuwa da ciwon Hanta/ shawara?

Respondent: Yana bayar da kariya sosai.

Moderator: Meyasa kika amince?

Respondent: Saboda Ni shaida ce. Wasu da mukasani suna dauke da wannan cuta mun tabbatar basu yin riga kafi. Mu kuwa da yardarm Allah, tun kafun mu cutu munyi rigakafi kuma Allah Yak are mu.

Moderator: Shin kina ganin zaki samu ayiwa yaro/yarinyar ki allurar rigakafin ciwon Hanta/shawara Idan kika nema da zarar kin haifu?

Respondent: Eh. ana samu ba bu matsala.

Moderator: Zaki nemi a yi ma yaro/yarinyar ki?

Respondent: Eh zan nema ko in Allah Ya yarda in dai akwai dama

Moderator: Me ya sa kika fadi haka?

Respondent: Sabili da wancan karon nayi kuma naga amfani. Yanzuma kuma ina son in nemi kariya

Moderator: Sau nawa ake yin wannan rigakafin?

Respondent: A'a. Bansan ko sau nawa akeyi ba.

Moderator: shin kina da wani tunani ko matsala da Kike ganin zai iya hana ki samun wannan allurar ta ciwon Hanta/Shawara da ake badawa da zarar haihuwa?

Respondent: A'a ba'a samun wata matsala. In dai ka taho kam zaka samu. In dai akwai kam ana yi ma yara shi. Daga nan har sheka ana yima 'ya'ya bayan watanni.

Moderator: Shin Kina gananin wannan allular rigakafin da ake yi da zarar haihuwa ta ciwon shawara/Hanta tana da amfani ga yaro/yarinyarki?

Respondent: Ee yana karewa.

Moderator: A jumlace shin Kina ganin cewa mata masu juna biyu a nan yankin na samun ita wannan allurar rigakafin ciwon shawara/Hanta da zaran sun haihu?

Respondent: Eh, suna karba. Yanzu ai kai yaw aye.

Moderator: Me ya baki wannnan tabbacin?

Respondent: Saboda Karin ilimi da aka samu a cikin al'umma

Moderator: Shin kina da wata shawara da za ki iya bamu ta yadda zamu kara wayar ma alumma da sauran jama'ar wannan yanki game da muhimmancin allular rigakafin cutar shawara/Hanta da ake badawa da zarar haihuwa?

Respondent: Ana yin shela. Sannan kuma kamanni haka in naje in rika gaya wa mutane yan uwa na. in ce anayin riga kafi kaza do mutane suzo don neman kariyar kansu.

Moderator: Shin zaki so k'arin sani game da ciwon Hanta/Shawara? (Ta hanyar Labarai, vidio/majigi, fosta, da sauransu)

Respondent: A ke dinga mana bayani duk lokacin da muka zo asibiti. Kuma yanzumma dai alhamdulillahi ana kan yi mana a ko da yaushe. Akan mana bayani akan abunda ya kamata mu dinga ci, mu ringa tsabtar gidajenmu.

Moderator: Shin akwai wani abu da kike tunanin ya kamata mu sani game da wanan ciwon, ko hanyar yada bayanai akanta ko gameda allurar rigakafinta?

Respondent: Shi de riga kafi yana da kyawu. Kuma da an haifi yaro a soma yi mishi rigakafi yanada muhimmanci domin kariya. Shi koma na ciwon hanta yana da kyau kowa ya yishi sabili da kariyanshi a cikin al'umma gabaki daya.

Moderator: Mungode da wannan lokaci da kika bamu.

State 1, LGA 3, Facility 6

Moderator: May I know your age?

Respondent: I am 30years old.

Moderator: 30years old. Your designation?

Respondent: Junior community health extension worker.

Moderator: Your position in the facility?

Respondent: RI in charge

Moderator: RI in charge, okay. So I will to know what are the biggest challenges for the people in your community about hepatitis B?

Respondent: The biggest challenge for the people in our community for hepatitis B is just because some of them did not delivered in the hospital, some of them delivered at home and if they delivered at home and we did not know they have delivered before the 24hours so it will give us the problem children will not get immunisation within the 24hours but if they bring the children we can immunised them before the 24hours but if they delivered in the hospital there is no problem we will give them the immunisation immediately of the hepatitis B.

Moderator: Do you think there is other challenge apart from the one that you have mentioned?

Respondent: There is no challenge only if some people they find it difficult to come because they are too far from the hospital if there is raining season between the time that they deliver so they won't come early because they are too far from the hospital some of them are too far that's why they can't come to the hospital.

Moderator: Thank you very much. But what about the people living with the hepatitis B, Do they have any challenge in this community?

Respondent: There are people that has the hepatitis B but if we found out they have it, when they come to the hospital and we do test and find out they have it the hepatitis B we refer them to hospital but if she is a pregnant woman and she comes for ANC we give her advice that they should make sure that she delivers at the hospital when they deliver immediately we give the children the hepatitis B injection.

Moderator: What are the biggest need to address the hepatitis B in your community?

Respondent: There is need, the need is that we need to educate the people so that they will know more about it. Some of them they don't know even about it and they don't know what is the solution you know they are some people who don't know how to take care of themselves or know what they would do so the need is that we should health educate them first. If we give them education about the hepatitis and we should give them advice to go to the hospital or nearest place health centre that they would do the hepatitis B test. And if they do they test if there is no problem it is negative, we give them advice to go for immunisation if they go for immunisation we believe there is no problem because everybody that take the hepatitis B injection and if there is we used to refer them to hospital.

Moderator: is there a strong need for the hepatitis B birth dose in these community?

Respondent: Yes! There is very strong need because there are some people that have the hepatitis B already so there is need that we should give our children hepatitis at birth because it will prevent our children's future from the hepatitis.

Moderator: So do you think other seek a need from the hepatitis B birth dose?

Respondent: Yes, there is because even though you don't have it, it is good for you to take the immunisation so that it will help you and will prevent you for the hepatitis not to gather.

Moderator: Can you tell me about the current programme or practices for the hepatitis B birth dose in your clinic?

Respondent: We are having the health education during the ANC, we are giving our pregnant mothers health education about it so that they will know how to take care of their selves.

Moderator: Is the hepatitis B birth dose easy to get within the 24 hours of birth?

Respondent: Yes, it is very easy if the women deliver at the hospital because we used to give them immediately after the delivery.

Moderator: So you have the vaccine in the hospital?

Respondent: Yes, we have it at the hospital.

Moderator: Who can administer the hepatitis B birth dose?

Respondent: RI in charge will administer it or any J2 who is in charge or CHO.

Moderator: What are the barriers or the challenges in getting the hepatitis B birth dose?

Respondent: The challenges is like the way I said it before if the women did not deliver at the hospital or they are too far from the hospital and during raining season that time and then they doesn't know about it but if they know about it there is no problem, the major problem is to know about it because if they have the knowledge of it they do come also the major problem is he distance from the hospital and various other places and the problem about education some of them don't know about it.

Moderator: So have you gotten information from participants regarding the hepatitis B birth dose?

Respondent: Yes.

Moderator: On what experiences?

Respondent: Experiences, there is time when we were training, they trained us about there is good in giving the hepatitis B birth dose it very important for us.

Moderator: Have you gotten any feedback from mothers that bring their children here or have heard about the hepatitis B birth dose?

Respondent: Yes, there is because some women that can testify that the children who get the immunisation fully from the birth and to the 9months there is difference between them and the children that do not get it because they do not have fever at times more like the other children.

Moderator: what is the community understanding the hepatitis B birth dose?

Respondent: The community understood that there is good for them to get the hepatitis B at birth dose because it will save their children from getting hepatitis.

Moderator: Are people interested in getting the hepatitis B birth dose?

Respondent: Yes! They are interested.

Moderator: Can you describe what kind of information or messages you have heard?

Respondent: There is a one woman that came for hepatitis and she said that her last child that she delivers she did not give her the hepatitis and she heard that it is good for her to get the child immunise because it is very important.

Moderator: Do you have any idea or suggestions on improving hepatitis B birth dose vaccination rate?

Respondent: Yes it is good as I said before to give our women or a community to help with education about it and to mobilise them during ANC and during the immunisation because if there is mobilisation like a support, something that we give them during the immunisation, you know some of them there is problem they don't even have transport to come for the immunisation if there is something we show them to motivate them there is a need if we get something like Motivation during the immunisation if they come it will be good.

Moderator: So Anything at level of community, something to do at level of the community to increase the rate of hepatitis B birth dose?

Respondent: There is because if we are having a home visit we can visit them, if there a woman especially during ANC and we know that this woman is at her 9months and for the weeks she did not come, is good for us to visit them and ask about for example during a home visit if we visit them at home and she delivers she didn't come for immunisation when we visit them we know and we get them to have the immunisation.

Moderator: What can you do at the level of your facility to make the rate of hepatitis B birth dose increase?

Respondent: Must it to give the health education, to have a home visit and to have a good motivation between us and the community.

Moderator: What of the at the provider level, what can you do differently to make people come to facility to deliver so that their children will get the immunisation?

Respondent: To Give them health education that it is good for them to deliver at the hospital because if they deliver we can give the children the hepatitis immediately so we will be able to get them before 24hour or visit them at home and be their friends.

Moderator: Is there any specific strategies or awareness with most effective?

Respondent: Yes, there is for example we can do either like posters or like a banner to educate them when we place it so that they will know some of the people when they see posters they will ask "Why is this?" And "What is this?" and when they ask we will explain to them and is good because some of them can hear from the radio and listen to all new information. I think it will be easier for us without spending anything to see the poster and if they see it they ask "what's this?" And "what's the meaning of this?", We explained to them about it, the banners and the posters.

Moderator: Can you tell me about your experiences about the maternal tetanus vaccination?

Respondent: The maternal tetanus vaccination happens if the women did not receive the vaccination during her ANC, during her pregnancy if she did not receive her TT and she did not visit the hospital,

she didn't come for her ANC maybe she has a problem she might have injured herself in some way she might have the tetanus with her and she did not take any TT as an immunisation it will affect her child.

Moderator: What barriers or challenges have you experienced related to the maternal tetanus vaccine?

Respondent: The barriers is that some of them they don't know about the TT and they don't know how to take care of themselves. You know in some village we go to farm even the cutlass we are using may hinder the tetanus. So it is good we educate our community if they use the cutlass or anything harmful they are using they should keep it in a clean place and in a way that it will not harm us.

Moderator: What successes have you experience and which one is working?

Respondent: We have success because before there is a problem in getting the hepatitis B at birth because for use here in hospital before we don't have fridge so there is no place to keep the immunisation vaccine clean or to keep it potentially but now we have it we are successful because now we are giving it any time the woman delivered immediately and we already give the woman education during the ANC and some of them are really trying.

Moderator: What types of health interventions have been the most successful in your community and why have they been successful?

Respondent: There is a program even now we are having a program that the government are doing during the OBR, during the immunisation campaign there is I-mop that they have been doing some campaign, there is program it will help us to reach house to house and to locate some people.

Moderator: Which one has been the most effective in the programs you have mentioned?

Respondent: During the outreach.

Moderator: Is there anything else you think we should know about the hepatitis B?

Respondent: We are giving them health education; we are giving them advice for the hepatitis B. The hepatitis B which we know it is contagious, it will affect somebody like somebody will get it through a partner, through relations. So we should know how to take care of ourselves, we should try to prevent that during a child birth let's say we deliver at home, we should then bring children for the immunisation and we should also know when we are supposed to bring the children for the immunisation not just when we deliver after some days or weeks before coming to the immunisation, if you did not deliver at the hospital you should try to make your best so that you reach the hospital before 24hours of birth and anytime you come to the hospital your child will be given the immunisation immediately.

Moderator: Thank you very much Ma'am for your time, we really appreciate!

HAUSA TRANSLATION FOR NOTE TAKING TEMPLATE FOR PREGNANT WOMEN KII GUIDE State 1, LGA 3, Facility 7

(KII NA MATA MASU JUNA BIYU/ MATA MASU CIKI)

Jadawallin bayanan wacce ake ma tambaya

Shekarun ki nawa? 25

Tambayoyi:

1. Me kika sani game da Ciwon Hanta/ shawara?
   Wani lokacin zaace dutse yashiga hantan mutum zaayita shan ruwa, da sauransu zaayita amai jinni. Babban ciwonne kuma ciwo wanda yake da hatsari
2. Me kika sani game da allurar Rigakafin ciwon Hanta/ shawara da ake yiwa yara dazaran an haifesu?
   Yin rigakafi taimako, ance rigakafi yafi magani

   2a. Meye ra'ayinki akan ita wannan allular rigakafin ciwon Hanta/ shawara da ake yiwa yara da zarar an haifesu?
   -agani na. iyaye mata su goyi da baya adinga yi wa yaranmu rigakafin ciwon hanta don bakarin ciwo bane-----------------------------------
   ----

   2b. Shin a baya kin taba neman a yi ma yaro/ yarinyarki alular rigakafin ciwon Hanta/ shawara da akeyi da zaran haihuwa?
   -
   Bansan da cewa akwai rigakafin ciwon hanta ba. Amma duk rigakafi da naji ina kai yara amusu---
   ----
   ----
3. Shin Kina da shirin bada yaro/yarinyar ki ayi masa/mata allular rigafin ciwon Hanta/ shawara da zaran kin haihu?
   --ee ina da burin yi--

3a. Meyasa zaki amince? Meyasa bazaki amince ba)?

Domin riga kafi kariya—kuma zai kareshi daga ciwon hanta na nan gaba--

3b. Shin Kina da wani ra'ayi, ko damuwa, ko d'oki, akan ita wanan allular? Meyasa?

------------gaskiya taimakone yana da kyau sosai, iyayesu goyi da baya adinga yiwa yara rigakafi-. Adaina jahiltan abin---

4. Shin Kina ganin wannan allular regafin ciwon Hanta/ shawara da zaran an hahihu na iya kare yaro/yarinyar ki daga kamuwa da ciwon Hanta/ shawara? Meyasa kika amince? Meyasa baki amince ba)?

Insha Allahu yana karewa. Don mutanen mu na da suna fama da ciwon hanta da zaran ya girmama. Amma yaran mu na yanzu alhamdulillah

----

5. Shin kina ganin zaki samu ayiwa yaro/yarinyar ki allurar rigakafin ciwon Hanta/shawara Idan kika nema da zarar kin haifu?
------ee zan samu cikin sauki--

5a. Zaki nemi a yi ma yaro/yarinyar ki?
--------ee ina da burin haka..-domin rigakafi kariya, zai kare shi daga ciwon hanta nan gaba-------wanda bamusan mai gaba zatayi ba--

5b. Me ya sa kika fadi haka?
----gaskiya taimakone. Iyaye su goyi da baya- ayiwa yara. Su daina jahiltar abin---

5c. shin kina da wani tunani ko matsala da Kike ganin zai iya hana ki samun wannan allurar ta ciwon Hanta/Shawara da ake badawa da zarar haihuwa?
-------------------babu--

----

6. Shin Kina gananin wannan allular rigakafin da ake yi da zarar haihuwa ta ciwon shawara/Hanta tana da amfani ga yaro/yarinyarki? (Meyasa kika amince? Meyasa baki amince ba)?
Ee yana karewa. Lafiyan yaran yanzu ba kamar na da ba-. Yakamata kowace uwa ta tsaya ayi wa yaranta rigaba---
----
----

7. A jumlace shin Kina ganin cewa mata masu juna biyu a nan yankin na samun ita wannan allurar rigakafin ciwon shawara/Hanta da zaran sun haihu?
----sai dai wanda baiji labara ba. Amma kowa yaji yana wa danshi riga kafi--
----

7a. Me ya baki wannnan tabbacin (ko rashinsa)?

-duk ranar rigakafi zaka samu mata sun tattaro yaynsu sun kawo riga kafi --
----
----

8. Shin kina da wata shawara da za ki iya bamu ta yadda zamu kara wayar ma alumma da sauran jama'ar wannan yanki game da muhimmancin allular rigakafin cutar shawara/Hanta da ake badawa da zarar haihuwa?
------adinga fadakarwa anunawa mata muhimmancin rigakafi----------------------
----

8a. shin zaki so k'arin sani game da ciwon Hanta/Shawara? (Ta hanyar Labarai, vidio/majigi, fosta, da sauransu)

Wani lokacin sai anabi gida gida, koma ana sanarwa--

8b. Ta wace hanya kika fi son Karin ilimi game da sha'anin kiwon lafiya?

--1. Ana tara mata ana fadakar dasu ana ilman tar dasu---

9. Shin akwai wani abu da kike tunanin ya kamata mu sani game da wanan ciwon, ko hanyar yada bayanai akanta ko gameda allurar rigakafinta?
--bani da wani abu na Karin illimi. Sai shawara, yadda kukeyi Allah ya kara muku ci gaba ---

Key Informant Interview of Officer in charge at State 1, LGA 4, Facility 1

Interviewer: Good morning

Respondent: Morning Sir.

Interviewer: Ah My name is [name]. And I'm here at [facility name] in [LGA name] with the officer in charge, we're about to do an interview and I just want to ask ah, do you agree to participate in this assessment?

Respondent: Yes, i have agreed.

Interviewer: All right, thank you very much. Like i earlier said, my name is [name]. I work with AFENET CDC. And we just doing an assessment about vaccines here. So uhm, sorry, Ma, can you help me with your age?

Respondent: I am 49

Interviewer: All right. And you are the officer in charge of this facility here?

Respondent: Yes Sir

Interviewer: Alright, thank you very much. (**Her**: thank you sir). So, question one. What are the biggest challenges for people living with hepatitis B in this community?

Respondent: Well, the challenge the people have, those that were able to know that statue, that for hepatitis B, we used to send them to Cottage Hospital for their drugs. And after giving their drugs, the fact that the treatment take longer time. Because of poverty, some of them, they will decide to drop the treatment halfway. And as a result, some will lead to dead, death. So far, the people we sent to cottage, most of them go and state it. (He: OK). But i have only a record of one person that drops the treatment half way, later, he begin to develop complications, that's the only thing i can say about it.

Interviewer: Thank you very much Ma. So, ah what do you think are the biggest needs to address Hepatitis B in this community?

Respondent: Well, the biggest needs to address it, you know, sometimes even the money to do the testing (He: OK) Even the money to do the testing is a problem because in the previous year, maybe, let me say last year, we use to bought the kits and do the testing for them. But with coming of this erh, Agaji Global Funds, they help us with some little amount of money. So, we decided to do the test for only pregnant women we do to them free. (He: OK). So, if you are a pregnant woman you came for clinic, you will have a free testing of hepatitis, Both A and C and B free, except, maybe you came from the town then we begin to say okay, you have to pay for the since the kits, we bought them, we need to retrieve the money to go and buy again. So, the challenges people have is sometimes when we tell them that they erh, they should redo the testing they will say, toh they don't have the money. (He: OK) And maybe they may had

hepatitis, they don't even know until maybe they're sick, it becomes so serious. Then after going through medical treatments, you know when somebody is sick, he lie down now. (**He**: Yeah), Ehen, if they tell him to do anything he can do, but by the time he on his feet, he will always complain no money. (**He**: OK). But the challenge of money, to do the testing, mm...

Interviewer: Ah, Thank you very much. Do you think there is a strong need for hepatitis B birth dose?

Respondent: I don't understand this.

Interviewer: Do you think that there is a need that we need to be doing this Hepatitis B birth dose is it important?

Respondent: It's important.

Interviewer: Okay. Why do you think it is?

Respondent: It is important because by the time you, we do it, it will help us to address the spread, of the hepatitis

Interviewer: So, uh do others see a need for these Hepatitis B birth dose? The one we give pregnant women ah when they deliver, at the time of birth? Do the people see the need for it? The importance?

Respondent: Yes. Like women that have antenatal clinic here with us. We, always advise them immediately after birth. If they deliver in the hospital, we give them immediately after birth, but if they deliver at home, uh, you know because of the maybe they are hard to reach areas or are not close or they are not privileged to come to the facility. I, I really appreciate the community, because immediately a woman deliver, even if it is at home, you'll find that they will give the baby to another, a caretaker, to bring the baby for the hepatitis. They're trying that and is i know it is because of the orientation the awareness they creates That is why, for the children they are trying. (**He**: OK). Mm.

Interviewer: Thank you very much Ma. So, question four. Can you tell me about the current programs or practices or services for hepatitis B birth dose in this your clinic?

Respondent: The, the current program is just, immediately after birth, we give them and, in a situation, whereby maybe they woman deliver somewhere and she couldn't collect, her first contact to the clinic, we use to give her (He: OK). Mm

Interviewer: Alright, so uhm, is the hepatitis B birth dose easy to get within 24 hours of birth?

Respondent: It's because it's always available.

Interviewer: Ok, (**Her**: Mm) So because it's always available? (**Her**: Mm). Is there any other reason you think that is making it easy here...

Respondent: It's making it easy because the women come to understand immediately after birth there is a vaccine a child supposed to get. So, it it makes it easy, because even if, as I said, even if they deliver at home, they know that there is a vaccine that a woman supposed to a child supposed to collect at birth. So immediately they will bring her we give her the hepatitis B with the OPV zero, then we schedule them because we schedule them for BCG since it has a, the doses are many so we schedule it in a week. So, every week we'll give them the BCG.

Interviewer: Ok. So, who can administer the hepatitis B birth dose? Here in your facility?

Respondent: It's a, any health worker. (He: Okay) Trained community health worker can do that.

Interviewer: Ok. So, what are the challenges or barriers you ca… you normally get in uh getting the Hep B birth dose vaccine? Yeah.

Respondent: Well, for now, since I (giggle) came to this place, we don't have any barriers, except maybe we'll have a stock out in the local government. But for now, there's nothing like stock out.

Interviewer: And how long have you been here?

Respondent: I have been here two years now. Mm

Interviewer: Okay. wow that's wonderful. So, have you gotten information from participants regarding their experiences with their Hepatitis B birth dose?

Respondent: Participants in the...?

Interviewer: Yes. The people that you do vaccinate their children with this hepatitis birth dose. The caregivers? Have you ever gotten any information regarding any experience they have had with the administration of this hepatitis B birth dose?

Respondent: There is nothing. i didn't heard of anything.

Interviewer: Okay. So, what is the community understanding of hepatitis B birth dose?

Respondent: Well, mm, the understanding they have is that the need to prevent liver disease. So, and, one of the way to prevent liver disease is to get hepatitis vaccine. So they are, they are kind of interested maybe in ha..having the vaccine, especially for their children.

Interviewer: Okay. Thank you very much.

Respondent: Thank you Sir.

Interviewer: Are people interested in getting this hepatitis B, birth dose?

Respondent: They're interested. Mm

Interviewer: Okay. Can you describe what kind of specific information or messaging that you had about this from the community, from people? the caregivers?

Respondent: Where there's nothing so precisely to say, (**He**: Okay), but all that I can say is that they are almost happy for their children to be vaccinated.

Interviewer: Ok. Thank you, Ma. So, do you have any ideas for any suggestions for improving Hepatitis B birth birth dose vaccination rates here in this community and facility?

Respondent: Toh, (heaved a sigh) erh...i don't know what to suggest ooo...

Interviewer: So just anything that you think ah, we can do to improve the rates since you been here in this community and this facility, how can we make it better?

Respondent: So, the only way we can make it better i think is this issue of TBAs or we use the head of settlements because most women, sometimes they deliver at home, even though they try to bring their chi children, that is if the woman attended ANC, she would make sure she bring her child for the hepatitis, but in a situation whereby those people that, that they couldn't attend ANC, sometimes when they delivered, they relax at home. So my suggestion is, let's more kind of awareness to be created to these TBAs and there are some women now, woman sometime, they don't even go to TBA, even in their home like that, labour started, any other woman in the home, she can collect the labour, the..they encourage the woman to bring the children. That is what I can say now.

Interviewer: Alright. So uhm, this idea or suggestion at the community, do you have any specific of this idea at the community level?

Respondent: Well, we had a meeting with the WDC's that is Ward Development Committee, and you know, the representative from different villages. We try to encourage them to mobilize women for us, one for ANC, second for RI. And we try to tell them the importance of these erh children vaccination, right from birth, that is what we have done so far

Interviewer: So, any, inside these ideas or suggestions that you gave, or any more that you can give, ah what do you think can be done at the healthcare system level to increase these ah rates to make it better? At the healthcare system level?

Respondent: You know, you know village people, they always needs, motivations, they always needs motivations, because ah, if they come, they were able to be given something as incentives to, to make them happy, it will now encourage the other ones. Maybe they're feeling lazy, they will now come up

Interviewer: Ok. That's, that's wonderful. So, what about at the provider level? The people that bring these vaccines to us? What do you think they can do to improve or make it better?

Respondent: what they can do to make this issue better, they should ensure that there is no stock out. (**He**: OK). When there's no stock out, the vaccines are available, this erh hepatitis B at birth, will be given, but when we have a stock out along the line, it will now become a problem.

Interviewer: Alright. Thank you very much. So, are there specific strategies for education or awareness that you think might be most effective?

Respondent: Well, the strategies i may want to say, talk about is if ah, maybe at the LGA level or representatives from the state level will be able to call the attention of this erh our rulers, traditional rulers and really talk to them, then they will, them too they will come back and talk to their subordinates i think it will help.

Interviewer: Alright. Thank you very much. Can you tell me about your experience with maternal tetanus vaccination?

Respondent: Ahhh.... This maternal tetanus injection is becoming an issue… (He: okay) And I said an issue is because of you know, human attitudes, you know, sometimes behavioral attitude is difficult to change. When a women came for antenatal, they will collect the first dose, they will collect the second those but coming back for the third dose, fourth dose and fifth dose is now an issue, because you find out that when they collected till when they are pregnant. If you are able to catch them up during their child welfare and give them the third one, the fourth one when they are not pregnant, they will not comeback. Except maybe we'll begin, if we if we are less busy or if we, if we remember to maybe track them through phone calls, but if not, kaiii. Most of them don't complete their doses. They will stop it halfway, it's just only few.

Interviewer: Ok. Alright. So, what barriers or challenges have you experienced related to maternal tetanus vaccination?

Respondent: The only challenge is getting them to collect the next, the, the the fourth dose, especially the fourth dose, that is the challenge. And sometimes we don't even have enough resources to be calling everybody. Sometimes even if you try to call the number two track the person that is supposed to come and collect, you'll find out network, they will say is not reachable, not reachable, a'a, the number does not exist. That's the problem we used to face and it's not only within these Banjiram that people came, some some came from other villages (He: OK). So, getting them...

Interviewer: Ok. So, what successes have you experienced so far? What have you been doing that is working for you on this maternal tetanus vaccination?

Respondent: The only success that we have is when we are able to track a woman, maybe we know that she's supposed to come for her next erh, TD, and she didn't come, or we're able to get her on the phone. Or if she is within this settlement, we ask one of our staff that they're

living here. And I, they know their house, they will now go on call them. They will come and collect. That is the one

Interviewer: Alright. So uhm, what types of health interventions have been the most successful in this your community? And why have they been so successful?

Respondent: The health intervention is just like this issue of RI and the free drugs that we use to give women during ANC. It really helps because it makes some woman to attend ANC, especially with this erh AUGUF intervention that started last year November. A lot of women that there, they used to refuse coming to clinic, because they'll say we go and do testing for hepatitis, go and do PCV you know, go and do syphilis testing. Some of them, they said you don't have money they will decide to stay at home. Then with this integration of erh Agaji global Fund, we use to do the hepatitis testing free for them, we used to do the urine glucose, both and urine test free, we used to do the syphilis free. So, with this intervention makes woman to come to ANC more than before, even though the program has ended this month.

Respondent: So, the little resources that remains, toh, when it finished, we don't how we're going to do again (laughs), but it really helped us

Interviewer: So, um, is there anything else you think we should know about Hepatitis B?

Respondent: Toh, babu fa (laughs)

Interviewer: Okay. So, is there anything else you think we should know about sharing information about the hepatitis B?

Respondent: Mm...toh, sharing information has to do with maybe individuals when somebody has the knowledge of how hepatitis is, then he will be able to maybe, we encourage that they will be discussing with ah, a friends, as we sat, there is needs to have the test so that you protect yourself from getting it.

Interviewer: Okay. So, is there anything that you think we should know about ah, the hepatitis B birth dose itself specifically?

Respondent: Kai, babu (laughs), babu

Interviewer: Ok. Alright, Ma, thank you very much ah, we have come to the end of our questions, and I must appreciate your time and your honest and very resourceful answers.

Respondent: Thank you Sir.

Interviewer: Thank you very much.

Respondent: Thank you, sir. I'm grateful.

Transcription of KII with Pregnant Woman at State 1, LGA 4, Facility 1

HAUSA

Jadawallin bayanan wacce ake ma tambaya

Interviewer (I): Sannu malama

Preg. Woman (P): Yawwa

I: Sunna na [name], muna aiki ne da AFENET CDC, mun zo nan Banjiram health centre, mu zo mu yi tamboyoyi ma mata masu ciki, bisa ga yadda suna zuwa ga asibiti sus amu kulawa ko kuma taimkon da gwamnati take yi ta wajen duba mata masu ciki, so da yaddar ki ne za mu yi maki wasu tambayoyi za mu yi recording bisa ga abin da an aiko mu?

P: E

I: Toh, sai ki daga muryar ki

P: Toh

I: Bari mu soma tambayoyin mu, tambaya ta daya shine, Me kika sani game da Ciwon Hanta/ shawara?

P: Gaskiya, ni dai ina jin Magana akan ciwon hanta amma ba'a taba yi mana explanation a kanshi ba, ban san komai a game da shi ba

I: Toh, tunda haka

Me kika sani game da allurar Rigakafin ciwon Hanta/ shawara da ake yiwa yara dazaran an haifesu?

P: Da an haife yaro ana bamu rigakafi na yaro kamar BCG

I: Ina jinki

P: Shine abin da ana bamu

I: Kina nufi, shine rigakafi da ana baku idan kin haihu?

P: E

I: Toh

Tambaya na biyu shine

Meye ra'ayinki akan ita wannan allular rigakafin ciwon Hanta/ shawara da ake yiwa yara da zarar an haifesu?

Meye ra'ayinki?

P: Yan taimakon lafiyar yaran mu

I: Toh

Shin a baya kin taba neman a yi ma yaro/ yarinyarki alular rigakafin ciwon Hanta/ shawara da akeyi da zaran haihuwa?

P: Ban taba yi ba

I: Akwai dalili da baki taba yi ba

P: Babu, domin ba'a taba kwayar mana ba, inda za'a kawo yaro ayi masa rigakafin hanta ba

I: Toh, tambaya ta hudu

Shin Kina da shirin bada yaro/yarinyar ki ayi masa/mata allular rigafin ciwon Hanta/ shawara da zaran kin haihu?

P: E, ina da niyar yi

I: Toh, Meyasa zaki amince?

P: Domin lafiyar su

I: Amma da'a ce baki amince ba, meyasa?

P: Saboda ban san dalili ko ilar shi ba

I: Shin Kina da wani ra'ayi, ko damuwa, ko d'oki, akan ita wanan allular?

P: Ba'a taba yi min kwayarsuwar sa ba, bazan sani damuwar sa ba

I: Meyasa baki sani ba

P: Abu ne wanda ba'a taba kwayar mini ba, balle in sani

I: Toh, mun zo tambaya ta hudu mallama

Shin Kina ganin wannan allular regafin ciwon Hanta/ shawara da zaran an hahihu na iya kare yaro/yarinyar ki daga kamuwa da ciwon Hanta/ shawara?

Shin Kina ganin wannan allular regafin ciwon Hanta/ shawara da zaran an hahihu na iya kare yaro/yarinyar ki daga kamuwa da ciwon Hanta/ shawara?

P: Yana iya kare

I: Meyasa kika amince?

P: Abu ne wanda likita ya kawo kuma sun karanta cewa wanan na da illa ko ba illa domin taimakon lafiyar jikin yaro

I: Amma idan baki sani ba meyesa baza ki amince ba

P: Saboda ban san illar shi ba

I: Toh, tambayar ta biyar

Shin kina ganin zaki samu ayiwa yaro/yarinyar ki allurar rigakafin ciwon Hanta/shawara Idan kika nema da zarar kin haifu?

P: E, zan nema

I: idan kin ce E, zaki yarda ayi

P: E

I: Me ya sa kika fadi haka?

P: Saboda lafiyar yarona

I: shin kina da wani tunani ko matsala da Kike ganin zai iya hana ki samun wannan allurar ta ciwon Hanta/Shawara da ake badawa da zarar haihuwa?

Ina jirar amsar ki

shin kina da wani tunani ko matsala da Kike ganin zai iya hana ki samun wannan allurar ta ciwon Hanta/Shawara da ake badawa da zarar haihuwa?

P: Babu, ba wata matsala, zan nema

I: Toh, tambayar ta shida

Shin Kina gananin wannan allular rigakafin da ake yi da zarar haihuwa ta ciwon shawara/Hanta tana da amfani ga yaro/yarinyarki?

P: Rigakafin na ciwon hanta na da amfani

I: Meyasa kika amince?

P: Domin yana da amfani, shi yas aka kawo mana

I: Idan dama, baki amince ba Meyasa?

P: Killa domin bazan iya aiki da shi ba

I: Tabaya ta bakwai

A jumlace shin Kina ganin cewa mata masu juna biyu a nan yankin na samun ita wannan allurar rigakafin ciwon shawara/Hanta da zaran sun haihu?

Tambayar in shine mata masu juna biyu, a yankin ku anan, suna iya nema alurar rigakafi d zarar sun haife yaro?

P: Idan an haife su a asibiti kam, suna kokari taimakawa

I: Me ya baki wannnan tabbacin ?

P: Saboda idan ka haifu a asibiti suna taimakon duba lafiyar ka, da yaronka

I: Tambaya ta takwas

Shin kina da wata shawara da za ki iya bamu ta yadda zamu kara wayar ma alumma da sauran jama'ar wannan yanki game da muhimmancin allular rigakafin cutar shawara/Hanta da ake badawa da zarar haihuwa?

Kuna zuwa wannan asibiti kuyi awo, akwai wata shawara akan inda za'a kara wayar da aluma akan rigakafin na ciwon hanta

P: hmm

I: Kamar da kika ce baki samu kwayarsuwa akan wanan cutar hanta, shine muna tambaya, akwai wata hanyar da za'a iya kawo muku wani jawabi?

Ta wane hanya

P: A nawa kam, yana da kyau kamin a yi maka rigakafi a bayana maka karfin alurar, a bayana maka hasken shi domin ka san amfani da illar s.

Amma idan mun zo babu shawara, babu tautaunawa, anyi maka gwaji ko awo . Kamar yadda kuna tambaya, zan iya in bada amsa mai kyau. Yaro bai ji dadin jiki bazan iya sani yadda zan yi ba. Ya kamata, a yi dan bayanai kadan mene wannan ko wancan.

I: Toh

Tambaya ta takwas, shin zaki so k'arin sani game da ciwon Hanta/Shawara? (Ta hanyar Labarai, vidio/majigi, fosta, da sauransu)

P: Ina son in sami domin ko a baya zai taimake ni in bada amsoshi

Masu bayannai akan ciwon hanta, ta wane hanya

P: Ina son in san ko ta wanne gefe ana samon

I: Toh, tambaya ta takwas

Ta wace hanya kika fi son Karin ilimi game da sha'anin kiwon lafiya?:

P: Kamar yadda muka zo asibiti zuwa awo, za'a iya yi maka bayanai a lokachin

I: Toh, ko ta ina kika fi so

To kin fi so wane hanya ne

P: Shine ina gan kamar yadda na zo awo, zan iya sami kwayarsuwa

I: Toh, tambayar ta karshe

Shin akwai wani abu da kike tunanin ya kamata mu sani game da wanan ciwon, ko hanyar yada bayanai akanta ko gameda allurar rigakafinta?

Kin gane tambayar ?

Akwai wani abin da kina so ki gaya man

Ko daga bayani da , Kina gan akwai wani abu

P: Amma, yadda basu yi mana bayani, ni nawa kafin a fara bamu rigakafi nah anta, ya kamata ayi mana bayanai batun ciwo mai barna. Domin mu sani game da ciwon

I: Toh, ta wajen yadda labaru akan inda za'a dinga yi mana bayani na alura rigakafi domin mu gane

Muna so a dinga yi mana bayani dala dalla domin mu san amfanin shi kuma ya bamu kuzari mu kawo yaranmu.

Ka gan idan mu kawo su, tunda bamu gane kan ma’anan ko muhimmanci shi ba

Ya kamatamu sani muhimancin maganin, idan bamu Sani ba baza mu kawo yaro ba saboda bamu san darajar shi ba.

Amma ka gan idan mun san amfanin shi, ko bamu iya kawo yaroba, zamu iya turo wata da yaro domin mu nema lafiyar yaro, shine nawa. Idan za’a iya bamu bayani akan amfanin rigakafi kafin a fara zai yi taimako

I: Toh mun gode malama, mung ama tambayoyin daza mu yi maki.

Mun gode

Key Informant Interview of Midwife at State 1, LGA 4, Facility 4

Interviewer: Good morning.

Respondent: Good Morning.

Interviewer: My name is ah [name]. I'm here with [name]. Like I said earlier we work for AFENET CDC. And we're doing a baseline assessment about vaccines in [state name], which has been approved by the state government. Thank you for your interest and time for this interview, the interview is voluntarily and you can't stop or choose not to answer any questions throughout the interview. There are no right or wrong answers. We just want to get your honest thoughts and opinions; the interview is being recorded for our accuracy. And nothing will be traced back to you. We will keep all materials private, and will not share with anyone. It will take approximately 30 minutes or less. Ah, do you have any questions about this interview?

Respondent: No.

Interviewer: All right. Do you agree to participate in the assessment?

Respondent: Yes.

Interviewer Alright, thank you very much ah. S o I am here in Chikila PHCC with a healthcare worker, a midwife for the baseline assessment, ah, please can help us with your age?

Respondent: I am 30.

Interviewer 30. All right, thank you very much. So, like I said uhm, there are nine questions and ah we'll quickly just run through them. And ah just give us your honest thoughts and ideas and suggestions. Ah, Question one. So, what are the biggest challenges for people living with hepatitis B in your community?

Respondent: The challenge we have here is that if we test a person and we found out that he has hepatitis, we usually advise or refer him to either Guyuk or Numan for proper treatment.

Interviewer: Ok

Respondent: So, the challenge here is that some of them, usually don't go.

Interviewer Ok.

Respondent: Mm, thats all

Interviewer Thank you very much. So, ah borrowing from that, question two, what are the biggest needs to address Hepatitis B in your community? In your opinion, what do you think are the biggest needs that ah need to be put in place to address hepatitis B in this community?

Respondent: And the need is they should make people to be aware of hepatitis B, and also to advise them to come for checkup to make a diagnosis.

Interviewer: Ok. Thank you very much uhm. Is there a strong need for the hepatitis B birth dose?

Respondent: You said?

Interviewer: Is there a strong need for hepatitis B, birth dose?

Respondent: Bad dose?

Interviewer: The one that is being given to ah, ah women immediately they deliver? They give hep B vaccine to women immediately when they deliver babies within 24 hours.

Respondent: Ok.

Interviewer: So, do you think there is a need for that? Do you think there is a need for that vaccine? Birth dose

Respondent: Yes, there's a need. Mm.

Interviewer Ok. Why do you think so?

Respondent: Because when they come for antenatal, we do testing for hepatitis B and when we find out they are negative, we advise, them to have the vaccine to protect them from getting infected.

Interviewer: Okay, so assuming there is no need for it. Why do you think so?

Respondent: Uh

Interviewer: Just to sample your opinion on the other side of why you think it is a need? Why do you think there is no need for it? Do you have any anything about why you think it shouldn't be a need?

Respondent: No.

Interviewer: Okay. All right. So, question three b. Do others see a need for hepatitis B birth dose? Do other people see the need for this vaccine?

Respondent: Yes, some. Mm, some

Interviewer: Ok. All right. Question four. Can you tell me about the current programs or services or practices for hepatitis B birth dose in your clinic? Is there any that you're aware of?

Respondent: No

Interviewer: Any program, any service, any activity?

Respondent: No, there is none

Interviewer: around Hepatitis B?

Respondent: No

Interviewer: Ok. Next question. Is the hepatitis B birth dose easy to get within 24 hours of birth in this facility?

Respondent: Yes

Interviewer: Okay, why do you say so?

Respondent: Because we have it here. Mm

Interviewer Okay. All right. So, if ah, it easy to get within 24 hours, why do you think that may not be So? If there is a situation where it's not easy to get within 24 hours, why do you think, what will be the reason why it won't be easily accessible?

Respondent: Maybe it has finished. Yeah.

Interviewer: Okay.

Respondent: Out of erh or short of it

Interviewer: Thank you very much. So, in this facility who can administer the hepatitis B birth dose.

Respondent: Other colleagues

Interviewer: Okay. All right. What designations is that colleague? that rank?

Respondent: She's a CHEW

Interviewer Okay. Apart from her, any other person.

Respondent: Yes. I have some CHEW that we are also working together.

Interviewer Okay. All right. Thank you very much. So, what are the challenges or barriers to getting Hepatitis B birth dose vaccine? Are there any challenge or barriers you encounter here?

Respondent: No

Respondent: Okay, Question...

Interviewer: What my, what my colleague is saying is that since sometimes, as you mentioned, earlier, it may, it may finish. So, or even, if, if it is present, do you encounter some challenges or even barriers for people to assess, or even get this hepatitis B birth dose

Respondent: No, apart from maybe if we, we did not have the vaccine, we don't have any barrier or challenge

Interviewer: Alright. Thank you very much. So, question five. Have you gotten information from participants, from people who received this vaccine? regarding their experiences with this Hepatitis B birth dose?

Respondent: No.

Interviewer: Have you ever gotten any feedback from people? Or had anything from the community concerning it?

Respondent: No

Interviewer: So, what is the community understanding of the hepatitis B birth dose?

Respondent: They know that the vaccine protects one from getting hepatitis B

Interviewer Any other thing?

Respondent: No

Interviewer Okay. So, are people interested in getting this Hepatitis B birth dose?

Respondent: Yes. Okay. Yes, they are.

Interviewer: All right. Can you describe what kind of specific information or messaging that you have heard from your community? concerning this? Like you said they're interested in it.

Respondent: Mm

Interviewer: So, is there anything you have heard them said or listen to from the community or from ah caregivers concerning what they feel about this vaccine?

Respondent: Yes, there, they are much interested in getting the vaccine because the the they are aware that it will protect their children from getting infected

Interviewer let me just come in, how did they get that awareness? That's what this question is trying to ask i guess, how did they get the awareness, that's the information they heard, through which means or how did they heard the information about the...

Respondent: usually when they come for antenatal and immunization, routine immunization, will, we do tell them

Interviewer Alright, thank you very much. Next question is Do you have any idea or suggestions for improving Hepatitis B but birth dose vaccination rates? Do you have any suggestion you can give us any idea you can give us, maybe first at the community level, how how can we improve it? Then at the healthcare system level, how can we improve it then at the provider level, how can we improve it? Let's start with the community level. How do you think what idea or suggestion do you have to help us improve the rate of this vaccination?

Respondent: at the community level the, especially the, the leaders, they should make them to be aware of the importance of the vaccine. So, they are the leaders of the community. They will be the one to tell other people and to influence them to make them to be more interested in getting hepatitis B vaccine.

Interviewer Thank you very much for that. What about the health care system level?

Respondent: At the health care system, usually when they come for antenatal, antenatal, they do give them health talk on vaccines so that's how

Interviewer So, what about at the provider level? The people that give us these vaccines people that supply these vaccines, is there any suggestion you have for them to improve the rates of this vaccine?

Respondent: Ok. they should make it available for for us to assess

Interviewer Thank you very much. uhm, question six B, are there specific strategies for education or awareness that you think might be most effective in this community? What what kind of methods do you think will be very effective in passing out messages or creating awareness to people?

Respondent: Mmm... This, it should be, we should just inform like maybe in a church, in a mosque, in, anywhere, especially the health workers, when they are among People, they should also enlighten them about the the vaccine.

Interviewer Ok. Thank you. What barriers or challenges have you experienced related to the maternal tetanus vaccine? Now we have shifted to tetanus vaccine. The one that ah, maternal tetanus vaccine given to women. Are there any challenges or barriers we have experienced with it, what's your experience with the maternal tetanus vaccine?

Respondent: The challenge we're experiencing here is that when the the they came for antenatal, first visit, they're usually receive that first dose of the of TD and the subsequent ones, they'll come and receive the second one, but when they deliver, they usually stop, the say that they have they forgotten, mm, so that

Interviewer Ok, Are there anymore?

Respondent: Mm mm (No)

Interviewer Ok. Thank you very much. So, what successes have you experienced so far? What what has been working for you? In these maternal the case of this maternal TD? Is there any success that you people have recorded in ensuring that it's making them to come and get this thing? Have you ever tried something that has worked for you?

Respondent: Yes, when they come for immunization, that immunizing their children, we usually tell them those that started this vaccine, they should come and collect the remaining ones.

Interviewer: Thank you very much for that. Question 8, so what types of health services or interventions have been the most successful in your community? You know there are different interventions or services being brought by different partners, government and international NGOs to this community. Which one do you think has been the most successful?

Respondent: Like the PMTCT.

Interviewer Okay

Respondent: Mm they, we we have started it on April. It has been a success. Yes

Interviewer Okay, so can you just throw a little bit light around this PMTC?

Respondent: Okay. Er, that program is usually for to identify ah, positive case during pregnancy. So, we usually...

Interviewer Positive case for which disease?

Respondent: HIV

Interviewer Okay. All right.

Respondent: So, when they, they have provided us with, with testing kits, thats for HIV, and the medication (OK) they are provided it all. So, when women come, we usually test them, and we found some cases in this community, and they are coming to collect their medication.

Interviewer OK. Thank you very much why, why, why, do you think it was successful?

Respondent: It was successful because the, the at antenatal, we do encourage them to to do HIV testing. And the lab technician? She usually tests all the women, that's first visit. She usually tests them all for HIV, first visit.

Interviewer Thank you very much. Is there any other reason why the program was successful? Why you think it was very successful?

Respondent: Yes, there, they staff here also are contributing, because if the other person is not around, the other person will do the job.

Interviewer Thank you very much. So now, our last question. Is there anything else you think we should know about Hepatitis B? Anything you think we should know that we don't know? We, government partners don't know.

Interviewer2: Okay, like now ba, let me just interject here. What he's asking here is that, do you think, because you are in the health facility, (Midwife: Mm) we, we are not. So, this question now is asking, do you have anything else you think, government should know, because we now represent the government that came ti intervene, that you want to share, so that they can improve, understand or enhance about this er hepatitis B birth dose, in this community or in this health facility? Do you have any additional information apart from what we have asked?

Respondent: is it birth dose?

Interviewer: Yeah, so first is hepatitis, you have hepatitis in general before we now come to birth dose.

Respondent: Well, the only thing that i can think of is that the testing kit for hepatitis B are not available and we usually buy and do the test for people so the they, especially the pregnant women, they complain that they don't have money to do this Hepatitis B. So, I think, maybe if we have enough testing, testing kits, yes

Interviewer: Alright. Thank you very much ah, what about anything else for us to know about sharing information about hepatitis? The manner in which we share information? Is there something you need for us to know?

Respondent: About how we?

Interviewer: We share the information, how we tell people about hepatitis? Is there something you think we can do that is better than what has been going on so far?

Respondent: The only thing is to create awareness to people (He: OK), Mm...that they should go for hepatitis B testing and they should be tested

Interviewer: Okay, so specifically now about the birth dose of hep B. Is there anything you think we should know to improve it?

Respondent: In that but the the only thing is just to make the vaccine available. (He: Ok) Mm.. Because the people of this community don't have problem of receiving the vaccine, if only the vaccine is available.

Interviewer Ok. It's alright. Thank you very much, ah healthcare worker, thank you very much for your time. We appreciate your honest opinions and answers and ah, we are grateful. Thank you

Respondent: Ok. You are welcome, Sir

Transcription of KII with a Doctor at State 1, LGA 4, Facility 5

Interviewer: (I) and the Respondent: (R)

I: Just as I said, my name is [name], and I work for CDC-Afenet. We are doing this assessment about Hepatitis B in [State name]. And [State] Government has approved this assessment. This interview will take approximately 30 minutes to complete. Do you have any questions about this interview?

R: No

I: So do you agree to participate in this assessment?

R: Yes

So thank you. So I am with the doctor in charge of [facility name]. We are going to interview him based on what to the question that we are keeping on to us so that you can give us what he knows about what I just saw? Can you tell us how for those two?

I: So sir, can you tell us how old are you?

R: 34

I: So the first question here is, what are the biggest challenges for people living with hepatitis B in your community?

R: Lack of knowledge about the disease. And then it's management, paucity of knowledge concerning the disease condition,

I: Can you project your voice so that it can record

I: So the second question, what are the biggest needs to address hepatitis B in your community?

R: Health education of the community about mode of transmission, and then my management of my hepatitis B infection, majority of the people are being deceived. Okay, by some traditional people, and then probably some other health related workers, per se, we had poor knowledge about the disease condition. So basically, health education, massive health campaign and awareness of hepatitis B virus and then its management line.

I: Is there a strong need for the hepatitis B birth dose?

R: Sorry!

I: Birth dose, that is immediately when mothers give birth?

R: Yes. i think it should, especially for positive mothers.

I: Okay! And if not, why not?

R: If not, if there's no need for the hepatitis B, i think there is need for it oh, especially like I said for positive mothers, who probably their viral load, that is their Hepatitis B viral load at the time of delivery is greater than 20,000, is it, International Units microgram per liter international unit, right. As I said, especially for a woman who has viral load or greater than 20 International units, as of the time they are putting to bed. I think there is great need for this thing.

I: So do others see a need for data? But those are?

R: Well, you know, just like I said that they have paucity of knowledge about the disease in itself entirely. So even if you're talking to them about it, they think as if you're just saying that for me, maybe moneymaking sake, considering the fact that they will pay some little stipends to get the vaccine. So sometimes they think along that axis, okay.

I: Can you tell me about the current programs or practices for the hepatitis B birth dose in your clinic?

R: Ah! Actually, for this particular facility, you know, we are constraint; one that most of the women who comes to deliver, especially the hepatitis B reactive ones, when you talk to them about need for the viral load, which I think is a little bit expensive, so they feel like that is waste of money. So in the real sense of it in this setting, we don't get to do the viral not to know which woman or which pregnant woman is eligible to get probably to be commenced on viral suppressors and then subsequently when she gives birth. So ah like I said, the knowledge is very paucity, and as such. The practices, obviously, in this facility for known is not optimal. When you're talking about issue of the practices in this facility regarding the birth dose of hepatitis B vaccine.

I: So is the hepatitis B birth dose easy to get within 24 hours of birth?

R: Not readily available.

I: Why?

R: I think you know, we do get from the cold chain, but it's not as if and I think it's only Mondays and Thursdays - (pause) that she does go to the cold chain or thereabout if I'm not mistaken. So imagine what happened to women who comes around to deliver on Wednesdays, Tuesdays, Fridays, Saturdays and the rest of them? So usually what you expect that these ones are not, they may not really get within the first 24 hours.

I: So who can administer this vaccine?

R: In this facility or you're talking about?

I: Yes, in this facility

R: Okay is the community health extension officer in charge of immunization. Ah in she's sorts that out.

I: So what are what are challenges or barriers to getting there, but it goes,

R: Like I said, immunization days, this particularly I think, if I told you if Monday's I chose this, so we got it from the agenda, that is one of the sometimes there are issues of OS, you get to do culture and as well out of stock. And again, manpower, we wish these will be sustained 24 hours in the facility, we have only one person who is mounting the immunization sector as a result of lack of manpower. So these are some of the barriers.

I: So have you gotten information from participants regarding their experiences we did, but I disappeared, but those for no no longer tend to stick. So what is the community understanding of the hepatitis B?

R: Like I say, it is plenty lack of information about the disease, the disease itself in an entirety, they don't have much knowledge about not to talk about the birth dose of the vaccine. However, I think recently, not up to two weeks. Within the last two months, I think I visited two congregations, which we talk to ourselves about hepatitis as a whole. So we're hoping that with more sensitization and then lectures, people should be aware on the birth dose of hepatitis B vaccine, and subsequently it will increase but for now, the information to the community about is low.

I: So are people interested in getting the hepatitis B birth dose?

R: Hmm, Yes! Just like I said, you know, we're rolling out campaign on the hepatitis as a whole. Actually, our focus was not on the birth dose, per se. But in the last series of lectures, we had the focus was on hepatitis as a whole and I think, maybe subsequently, when we have discussions like these we will include birth dose necessity for the birth dose as part of our lecture campaigns.

I: So can you describe what kind of specific information or messaging you have heard?

R: From my audience?

I: Yes, from the people accessing the services in your clinic?

R: For hepatitis or for the birth dose?

I: Both whether hepatitis or birth dose?

R: Generally, you know for the hepatitis, they have plenty misconceptions about hepatitis frankly speaking, they assumed viral suppressors to be cure for hepatitis B, which that is not true we all know. But they assumed the virus suppressors we do give like telbivudine, lamivudine for people who have greater than 20 International you do they assume it to be for cure, others resolved into herbal medications, which is so paradoxical. That herbal medication seems to be one of the things that injured the liver, and then they are still giving, you know, herbal medications in treatment for hepatitis B, then the last thing is the myth behind diet. That is one of the challenges we have, they are used to this concept that for any person who has Hepatitis B, virus should not eat meat, should not eat oil, you know, this, this, these are just wrong perceptions that the community has about Hepatitis B as a whole, which the teaching has since moved from that axis long ago.

I: Question six Sir. Do you have any ideas or suggestions for improving Hepatitis B-BD rates?

R: Massive campaigns and availability of vaccines

I: So what are the ideas or suggestions you have at the community level?

R: For the community level?

R: Okay, is to engage gatekeepers of the community, the Traditional rulers, probably CAN, Muslim council. Okay, and then let's talk to them through health education and the need for this hepatitis B vaccine, especially the birth dose.

I: What of the healthcare system level?

R: Well, at the health facility level, I think, you know, like the birth dose now, I think is something coming in much more new in the management of hepatitis. So the health worker should also be opt mated about these developments through health education too.

I: What of the provider level?

R: Hmmm?

I: At the provider level, that's hepatitis B-BD provider, who administer this drug, so what ideas or suggestions do you have for them to improving the Hepatitis B-BD there too?

R: To ensure that, the birth dose is getting before 24 hours and then the cold chain systems should be maintained in order to maintain the potency of the vaccine.

I-Colleague: Sorry, if I may chip in again. And then on this. Also the provider level for those supplying the vaccines. Do you have been any suggestion idea to help them improve?

R: Well, for now, no, I have not really monitored supply chain live to know whether there has been gaps in their supply or whatever to make suggestions on that.

I: So are there specific strategies for education awareness that might be most effective?

R: In this community? Specific strategies!! I said, basically, is the health education using the gatekeepers, the traditional council, the CAN and the rest of them. Because when you know when, like the Muslim council now by the time you have the chairman, okay, selling the idea to them in the Mosque. And then of course, you give you free flow to pass the information to the gathering, the same thing with the CAN and the traditional councils, there was a time I gather them here, the Traditional Council, Muslim Council, and then the Christian Council, we discussed on some issues. So we're actually preparing to, to, to stage out massive campaign, especially that one was for HIV. But I think we'll still see how we can incorporate hepatitis next time.

I: Question seven. Can you tell me about your experience with the maternal tetanus vaccination?

R: Maternal tetanus vaccination!! And he coughs

R: That is during ANC you know, in an ideal situation, pregnant woman suppose at least she supposed to get three doses during the course of our first pregnancy, but because the awareness will ANC in the villages, as I speak to you is still very poor, most of them booked at six months. Some book even at seven months, the earliest you get for booking is around four or five months ago, so you now look at the timeframe. They hardly the complete the three course, at least, of the tetanus toxoid vaccine in the first pregnancy, but however, for the few that comes up for anti Natal, we try to give the ones she can take within that period of antenatal care.

I: So what barriers or challenges are experienced related to the maternal tetanus vaccination?

R: Not much for the people who present themselves for the ANC. That has been going on in order except for some few naive people who actually said no, that “me ma suna ce” or what do they used to call it “da cewa muna da harbin daji” so we don't take injection except for some few naive clients like that. But after much health education some of them accept and some few still believe once they get injection, they believe the “harbin daji” will be worsened and they may die off, except for those few cases, otherwise, they acceptability and then experience from the ANC has been good.

I: So what successes have you experienced?

R: Successes in terms of the vaccine?

R: Of course they prevention. We haven't had any recent case of neonatal tetanus. Neither have we had ah…. Yes! (Intruder: Excuse! somebody just barged into through the door. Oh! sorry)

R: Of course, it clearly shows in the delivery. All our aim is to prevent neonatal tetanus in these babies. And of course, we haven't seen any recent case. That is to say that yes, the vaccine is actually making headway, is giving results and is great successor.

I: What types of health interventions have been the most successful in your community. And why have they been so successful?

R: Actually preventive, which is the immunization, that's been one of the key interventions that I've heard in the community. We have noticed our recent success on polio that Nigeria has been declared polio free. And this was as a result of the

successes recorded in the preventive aspect, which is the immunization. So I believe the preventive aspect is coming to stay, we're gaining more acceptability on like before that some people decline people not to go into their houses and the rest of them.

I: The last question, now, sir. Is there anything else you think we should know about hepatitis B?

R: Huh (Deep sigh) and beating the desk/table

R: Think you people should know? (Laughs)

I: Yes.

R: Like what now! I don't think oh!

I: (Laughs) about the government or anybody?

R: For you people, I don’t think there is anything but?

I: Because we are taking this thing being recorded, we are going to tease it out and we deliver to them, so if there is anything that you think we should know that is, they should know not we as per se but they should know. Because we are reporting or relating to them. So I think you can tell us about hepatitis B?

R: Just that, is frankly speaking this is nothing new that they should know that many people out there have very wrong perception about this hepatitis B and especially I repeat about the treatment of hepatitis B

I: What about sharing information about hepatitis B?

R: Yes, sharing information. I think there is, I have said that earlier there is paucity of knowledge generally in most communities about Hepatitis B. Some of them just the anxiety, the fear of the hepatitis B is what ends up you know, bringing them down that the hepatitis B itself.

I: So what about the hepatitis B birth dose? Is there anything you think you can let us know?

R: Yes, more awareness, more awareness should be created to everybody I think so.

I: So thank you Sir

R: My pleasure, alright

I: So we thank you for the time you have taken and gave us audience to answer the questions. We really appreciate

Transcription of KII with Pregnant Woman at State 1, LGA 4, Facility 6

HAUSA

Jadawallin bayanan wacce ake ma tambaya

Interviewer (I): Sannu madam

Preg. Woman (P): Yawwa

I: Sunna na [name], muna aiki ne da AFENET CDC, mun zo ne muyi miki tambayoyi da wasu mata masu ciki game da ciwon shawara ko hanta. Amma kafin mu fara, ina so in tambaya, zaki bamu amsoshin tamboyoyin daza mu yi miki a cikin mintin talatin, zaki bamu zarafi?

P: E, na baku

I: Tun da kin yarda, za mu fara tambayoyi game da ciwon hanta ko shawara

Tambaya ta daya, Me kika sani game da Ciwon Hanta/ shawara?

P: Ok, game da shawara, sun ce kamar ruwan da muke sha wanda ke da datti da kwayar cuta, shi yake kawo shawara. Toh yana da kyau mu tsabtacce ruwan da zamu sha kadda ya zamma da datti, ko inda muke zama ya zama da tsabta

I: A nufin ki kenan, ruwan da ake sha ne ke kawo ciwon hanta ko shawara?

P: E, ciwon hanta, bisa ga ababen da mutum ke ci ko cin nama idan ya yi yawa.

I: Toh, tambaya ta biyu

Me kika sani game da allurar Rigakafin ciwon Hanta/ shawara da ake yiwa yara dazaran an haifesu?

P: Abin da na sani shine, idan mace na da shi zai iya ya kama yaro, shi yasa ya kamata a yi ma yaro rigakafi don kada ya kamu da ciwon sai ya girma.

I: Toh,

Meye ra'ayinki akan ita wannan allular rigakafin ciwon Hanta/ shawara da ake yiwa yara da zarar an haifesu?

P: Yana da kyau idan an haife yaro a yi masa, domin idan ya girma zai kiyaye wa yansu ababen da zai same shi

I: Wani tambaya shine

Shin a baya kin taba neman a yi ma yaro/ yarinyarki alular rigakafin ciwon Hanta/ shawara da akeyi da zaran haihuwa?

P: Wanda aka yi wa yaro:

I: E, wanda kika yi wa yaron ki ko yarinya, kin taba?

P: E, na taba

I: Toh tunda kin taba

Shin Kina da shirin bada yaro/yarinyar ki ayi masa/mata allular rigafin ciwon Hanta/ shawara da zaran kin haihu?

P: E, zan yi

I: Tambaya na uku

Meyasa zaki amince?

P: Domin yanzu, ciwon yayi yawa kuma ana mana kwayaswa cewa yana da kyau ga yaro

I: Toh,Meyasa bazaki amince ba?

P: Ko don rashin sanni

I: Wani tambaya kuma

Shin kina da wani ra'ayi, ko damuwa, ko d'oki, akan ita wanan allular?

P: Kamar nawa:

I: E

P: Ni dai bani da dokoki, kamar da aka gaya mini, haka zan amince

I: Toh, Meyasa?

P: E

I: Meyasa?

P: Abin da yasa, domin kowane dokokin da an bani zai taimaka mini

I: Tambaya ta hudu

Shin kina ganin wannan allular rigakafin ciwon Hanta/ shawara da zaran an hahihu na iya kare yaro/yarinyar ki daga kamuwa da ciwon Hanta/ shawara?

P: E

I: Meyasa kika amince?

P: Domin kwayaswar da ake bamu akai

I: Meyasa baki amince ba?

P: Ban amince ba a rashin sani

I: Tambaya ta biyar

Shin kina ganin zaki samu ayiwa yaro/yarinyar ki allurar rigakafin ciwon Hanta/shawara idan kika nema da zarar kin haifu?

P: Kamar

I: Ace yanzu, gashi kina da ciki, idan kin haihu zaki nema ayi wa yaron ki alular rigakafin hanta

P: E, zan nema

I: Toh, zaki nemi a yi ma yaro/yarinyar ki?

P: E,

I: Me ya sa kika fadi haka?

P: Abin da yasa na fadi haka, domin kwayaswar da ake bamu na da amfani

I: Shin kina da wani tunani ko matsala da kike ganin zai iya hana ki samun wannan allurar ta ciwon Hanta/Shawara da ake badawa da zarar haihuwa?

P: Matsala kamar

I: Shin kina da wani tunani ko matsala da Kike ganin zai iya hana ki samun wannan allurar ta ciwon Hanta/Shawara da ake badawa da zarar haihuwa?

P: Babu

I: Akwai dalilin da kin ce babu:

P: Daliin shine tunda an riga an nuna mana inda zamu yi idan na haihu, shi yasa nace ko agida ne ma na haihu, zan kawo complain a yi wa yaro

I: Tambaya ta shida

Shin kina gananin wannan allular rigakafin da ake yi da zarar haihuwa ta ciwon shawara/Hanta tana da amfani ga yaro/yarinyarki?

P: E

I: Meyasa kika amince?

P: Abin da yasa na amince shine, na san idan ya samu rigakafi, zai taikmaka ga ciwo idan yayi girma

I: Toh, Meyasa baki amince ba?

P: Toh abin da na fada shine ko rashin sani

I: Toh, tambaya ta bakwai

A jumlace shin kina ganin cewa mata masu juna biyu a nan yankin na samun ita wannan allurar rigakafin ciwon shawara/Hanta da zaran sun haihu?

A Jumlar ku nan, mata masu juna biyu suna zuwa rigakafi?

P: Ban gane ba

I: A nan yankin ku, mata masu zuwa awo, idan su haihu, suna iya samu wannan alular na rigakafi

P: Akwai masu yi, akwai wayan da basu yi

I: Me yasa kin fadi haka

P: Kamar yadda na fada, wayansu na yi wayansu basu yi

I: Me ya baki wannnan tabbacin?

P: Domin ina ganin wayansu suna karba, wayansu basu karba

I: Me ya baki tabbaci

P: Wani ya karba kwayaswa yana amfani dashi, wani baya yi

I: Tambaya ta takwas

Shin kina da wata shawara da za ki iya bamu ta yadda zamu kara wayar ma alumma da sauran jama'ar wannan yanki game da muhimmancin allular rigakafin cutar shawara/Hanta da ake badawa da zarar haihuwa?

P: Shawara kam, kune kun san abin da ya kamata a yi

I: Muna so mu sani idan kina da wata shawara da zai taimaka domin mu kai wa wanda suka aiko mu, domin su kawo taimako. Shawarwarin nan ba shine final ba, kuna da shawarwari domin ya inganta wannan aiki

Idan kina da shi, sai mu sani

P: Toh, kwayaswar da kuna bamu ne, mu kuma muna sani ba mu yi.

Ban san ko akwai wata hanyar da za'a bi ba. Saboda haka shawar da za'a bamu domin mu bi doka

---

I: Shin zaki so k'arin sani game da ciwon Hanta/Shawara? (Ta hanyar labarai, vidio/majigi, fosta, da sauransu)

P: Ina so

I: Ta wanne hanya ne kina so ki sani game da ciwon hanta

P: Yana yiwa, ko kamar idan kana sharing riga ko zufa, ciwon zai iya ya same ka. Kamar wadda yake da shi, idan kun ci abinci zai iya kawo m mutum ciwon hanta

I: Shin zaki so k'arin sani game da ciwon Hanta/Shawara? (Ta hanyar labarai, vidio/majigi, fosta, da sauransu)

P: Kowane hanya, amma ina so ta wajen vidio

I: Ba wani

P: Ko vidio ko fosta

I: Tambaya ta takwas

Ta wace hanya kika fi son karin ilimi game da sha'anin kiwon lafiya?

P: Kiwon lafiya, kamar ko yadda muka zo haka ana iya bamu karin ilimi game da kiwon lafiya

I: Shin akwai wani abu da kike tunanin ya kamata mu sani game da wanan ciwon, ko hanyar yada bayanai akanta ko game da allurar rigakafinta?
P: Babu
I: Hmmmm!
P: Babu
I: Ko ta wata hanya
P: Ni kam abin da na sani shine cewa idan ka sha ruwa a kofi daya ko ka ci abinci a waje daya zai iya kawo maka ciwo
I: Ta hanyar rigakafi fa?
P: Ban sani ba
I: Malama bisa ga tambayoyin da aka bani in tambaya mata masu ciki,wannan ne guda tara, da wannan tambaya muka zo karshe a wannan sashi na mata masu ciki
P: Ok
I: Toh mun gode

Key Informant Interview of Community Health Volunteer at State 1, LGA 4, Facility 7

Interviewer: So, hello, my name is [name]. I work for CDC AFENET, we are doing an assessment about vaccines in [state name]. The state government has approved this assessment. Do you have any questions about this interview?

Respondent: I have a question.

Interviewer: Do you, Okay.

Respondent: My question here is that what the essence of this interview and what government wants to do for the volunteer in the health facilities?

Interviewer: That's the, that's the ah essence of us coming here to do a base line interview about a vaccine called, Hepatitis B birth dose. So that if this health facility workers know, any information about it or want to tell us what they know about it, we want to take it up, for government to analyze it. After the analysis, government will come to either intervene or either to give other help or any other assistance but for now we are being sent to the field to come and ask you this basic information about ah hepatitis B, that's the essence of our coming. So, do you agree to participate in this assessment?

Respondent: Yes, I agree.

Interviewer: Ok. Thank you. How old are you Sir?

Respondent: 25 years old

Interviewer: 25 years old, thank you. So, we are here in the [facility name] with my colleague [name], we want to interview a community volunteer in [facility name]. So, I will start by asking a question. The first question Sir here is what are the biggest challenges for people living with Hepatitis B in your community?

Respondent: The biggest challenges that we getting in our community is that we have lack of, erh, lack of prevention. Most of the community they do not receive the vaccines to prevent against uh, hepatitis B. And another challenge is lack of awareness about it.

Interviewer: Thank you.

Respondent: Okay

Interviewer: So, question two, what are the biggest needs to address hepatitis B in your community? Since you have told us the challenges. (Vol: Mm) So what are the ah biggest needs are address hepatitis B in about, in uh community?

Respondent: And, my biggest need is that we want government to, to carry out the vaccine that to give the community to prevent them from hepatitis B. And to create social awareness,

or issues orientation, to give the community to know the risks of the hepatitis B. Most of the people they do not know the risk of hepatitis B.

Interviewer: So, is there a strong need for the hepatitis B birth dose?

Respondent: Yes

Interviewer: If yes, why?

Respondent: Because most of the people in our community suffering from hepatitis B.

Interviewer: Ok. And if you say no, and if we say, No, why not?

Respondent: If I say no...

Interviewer: Uh, why not?

Respondent: If i say uh...

Interviewer: Okay, let's proceed to another question. Do others see a need for the hepatitis B birth dose?

Respondent: Yes.

Interviewer: Ok. They see the need for the hepatitis B birth dose?

Respondent: Yes

Interviewer: Question 4, Can you tell me about the current programs or practices for the hepatitis birth dose in your clinic?

Respondent: Current programs they are doing? (**Int**: Yes). For the hepatitis B birth dose? (**Int**: Yes)

Interviewer: What i am trying to say here is that, is there any activity carried out in your own health facility here or health clinic here about hepatitis B?

Respondent: There are no any program carried out about the hepatitis B

Interviewer: Even the practices?

Respondent: Yes

Interviewer: What about services?

Respondent: Services?

Interviewer: Yes. Rendering of services to people about hepatitis B. Have they ever carried it out in your own facility?

Respondent: Ok, they carry it. They carry the services. Yes.

Interviewer: Which one? Do you know it? Do you know any of the services they do here regarding hepatitis B?

Respondent: Yes, the services is, they test, the com, the people of this community, if they positive of hepatitis B, they referring them to general hospital in order to get the treatment.

Interviewer: So, the next question, is the hepatitis B birth dose easy to get within 24 hours of birth?

Respondent: Yes.

Interviewer: If yes, why?

Respondent: Because we have, we have the vaccine for hepatitis B in our in this facility

Interviewer: Ok, and if no, why not?

Respondent: If no, we, we inform the government to bring the vaccine for this.

Interviewer: Ok. So, who can administer the hepatitis B birth dose?

Respondent: Our RI in charge in this facility...

Interviewer: Any other person? Apart from the RI provider, any other person can administer hepatitis B birth dose?

Respondent: Yes

Interviewer: Who are they, apart from RI?

Respondent: Apart from RI in charge we have the in-charge facility, in-charge of this facility. (**Int**: OK). Mm

Interviewer: So, two of them or any other one?

Respondent: And another one is the health volunteer, community health volunteer.

Interviewer: So, what are the, what are the challenges or barriers to getting the hepatitis B birth dose vaccine?

Respondent: Uh, the challenges is uh, we did not have any uh, solar that we installed the vaccine in time, that's why we have get these challenges. The vaccines they do not come always that we, as the way we want.

Interviewer: So, have you got, have you gotten information from participants regarding their experiences with the hepatitis b birth dose?

Respondent: Yes, we have get it.

Interviewer: So, what are the experiences that they have told you or maybe you must have heard from them?

Respondent: The experience that we, the complain about, they tell us, most of the community they do not bring their child at the birth but some few of them, they bring their child during the birth.

Interviewer: Ok, so, what is the community understanding of the hepatitis B birth dose? What is the community understanding about the hepatitis B birth dose?

Respondent: The committee understand that the hepatitis B prevent the children against hepatitis. They, some of them, they know that but most of them, they do not know, they do uh, they do not understand that.

Interviewer: If that's the case, are people interested in getting the hepatitis B birth dose?

Respondent: Yes, some of them, they are interested

Interviewer: Can you now describe what kind of specific information or messages you have heard?

Respondent: Uh, the message that I hear on the community, the claim that, some of them if they receive the vaccine, they receive the injection of this hepatitis B, their child will be suffering from the hotness of the body. And that's why if some of them here about this. They do not bring their child if they are birth

Interviewer: So, question six, do you have any ideas or suggestions for improving hepatitis B birth dose vaccination rates? Okay (Vol: No), let me just, let me come in, do you have any idea or any suggestion or any uh, advice for people in the community to improve uh, coming down here to receive hepatitis B birth dose vaccinations?

Respondent: Yes, i have advise. My advice is, i want government to come close to, to improve, what they need, or what they challenges that the community getting, ge.. community get it. And my second advice, is that, we need some motivation that if they bring their children to receive hepatitis B vaccine, that they motivated them to give the community some motivation. So that, they encourage them to bring their children at, at done has give birth to their children.

Interviewer: So, and what advice do you, can you give to health center system here, since you are the one that is between the health center and community, so, what advice or suggestion do you tell health center here?

Respondent: My advice that I give to my, to health center is that to check throughout the villages, where, where they gave birth to the children to orientate them to carry out their children to the health facility at any time during, they gave birth to the children.

Interviewer: Okay, then, uh, then the other one, what advice would you give to the person giving the injection for the children, the hepatitis B? Maybe you come in, you just came now and you just see uh RI provider, even in charge given the uh, the hepatitis B birth dose. So, what ad… the person, advice will you give him about hepatitis B vaccine?

Respondent: Uh, my advice that i will give giving is that to create good rapport to the mother child, to, to teach them or to tell him that this vaccine cannot harm his child her child. So even he realized after the inject that children, that child, if she realized some of the action. They don't worry to bring back the child. They know what they give this child to recover. So

Interviewer: So, uh, 6B. Are there specific strategies, or or ways for education, or awareness that might be most effective? Do you think that, there are, do you have any specific ways that you can educate people or create awareness for them, for them to know more about this hepatitis B vaccine?

Respondent: Yes

Interviewer: Ok. What are the ways?

Respondent: Uh, uh ways to educate them is through uh through the churches.

Interviewer: Ok, through churches?

Respondent: Uh, they teach the community to understand the advantages of this and then their mosque like, mosque, the advice them and they teach them and other this our school that we do here. After this during the RI they came here and they teach them about the importance of receiving of vaccine of hepatitis B.

Interviewer: So, can you tell me about your experience with the maternal tetanus vaccination? This one now we are out of hepatitis now, we are about maternal tetanus now. Mothers do come for, for that uh, for that tetanus injection too. So, can you tell me about your experience with the maternal tetanus vaccination?

Respondent: Yes, my experience about tetanus is, we give the pregnant woman, woman to prevent her and her unborn baby from getting the tetanus infection.

Interviewer: So, what barriers or challenges have you experience related to the maternal tetanus vaccination?

Respondent: The barriers is most of the pregnant women, they do not agree to receive this vaccine of tetanus.

Interviewer: Ok. So, what successes have you experienced? Since the challenges mother they don't want to come to receive this vaccine. So, what other, what successes have you experienced ward mothers too?

Respondent: Some of the mothers those they are receive this vaccine, they tried to inform others that do, they do not receive, the importance of the receiving of this vaccine.

Interviewer: Okay, so, what was it, what was working? What was working then recording all these successes? What was you doing, working that make these successes to be achieved? Okay. Let me put it this way, since other mothers will come to receive the, receive the tetanus vaccine, they will go and inform other women to come and receive it, is there any other ways that was working more effective than this one?

Respondent: Any other way that is very effective?

Interviewer: Yes.

Respondent: No

Interviewer: So, question 8. What types of health services have been the most successful in your community?

Respondent: Health services?

Interviewer: Yes, health services that have been rendered by this center that have been successful in your community?

Respondent: Type of services that are successfully in our community is through, uhm, health education that will educated the community the importance, the importance of any, the importance of any kind of problem that they will face and then the services, the services has been through antenatal, through antenatal. During the antenatal if they women came to this facility for antenatal, we educated them the importance of antenatal and then another services is re-immunization when we are, during the immunization period we educated the woman about the importance of receiving the vaccines of immunization.

Interviewer: So, and why have they been so successful? As you said through ANC and immunization. So, what have these two ways have been successful?

Respondent: Because it is very, uh, because it is near to, it is very close for them to come at any time they want

Interviewer: Ok. The last question now, Is there anything else you think we should know about hepatitis B?

Respondent: Yes

Interviewer: Can you mention them?

Respondent: The thing that I know about the hepatitis B is that this hepatitis B is really risk to the people, it can easily transfer from one person to another but if they person get the hepatitis B, is if he come in time, he receive the curative and he will recover.

Interviewer: So, and is there is there any information that you want to share with me about hepatitis B?

Respondent: The information that I will share is that, uh, the people to know the, the risk about the hepatitis, if any sign of, if any sign of this disease appear to them. They do not waste their time at home. They come to hospital; the they will test them to know whether they have hepatitis.

Interviewer: Or is there or, or they what of about the information or anything you know about hepatitis B birth dose that you want us to know, do you have it?

Respondent: The hepatitis B?

Interviewer: Birth dose

Respondent: Birth dose? It prevent the child from getting the infection of hepatitis B.

Interviewer: Ok. So, thank you uh, our health community leader. I think i have exhaust the question that i have come to ask you. Thank you for the, for your time and the cooperation you gave me to answer these questions.

Respondent: Thank you Sir

Key Informant Interview of Pregnant Woman at State 1, LGA 4, Facility 7

(Rainfall sound)

Interviewer: Inna kwanan mu?

Respondent: [name]

Interviewer: [name], ah, inna aiki ne da CDC AFENET, inna nan anan tare da colleague na, sunan sa [name]. Muna dan yin karamin aiki ne akan muna son mu san abubuwa akan rigakafi. Munan nan anan [facility name] a Dumna ward na [LGA name]. Uhm, kaman yanda na gaya miki, akwai tambayoyin da za mu yi miki game da rigakafi, da cututuka, mu sanni mene ra'ayin ki a kan su. Idan kin yadda mu chi gaba da wanan interview, sai ki aminche

Respondent: A, na yada, sannun ku da zuwa

Interviewer: Toh nagode sosai. Ba tare da bata lokaci ba, za mu shiga tambayoyi koh

Respondent: a

Interviewer: So tambaya na fari, shekarun ki nawa ne, yi hakuri ko, sammana shekaran ki

Respondent: Shekaru na ashirin

Interviewer: Ashirin?

Respondent: A

Interviewer: toh, mungode. Toh, tambayoyi na fari. Ah, me kika sanni ga me da ciwon hanta ko shawara?

Respondent: Ciwon shawara? Abubuwa ne, ciwon shawara da yana kawo, akwai in da malaria ne yakan iya in ya yi wanan, sai ya kawo maka ciwon sharawa, a yanda kana yin aiki dayewa… ka siya a rana, abinchin da kana chi, mai dayewa kamar mai geda yakan iya costin ya kawo maka ciwon shawara

Interviewer: Ok, toh mungode. Uhm, Tambaya na biyu…Me kika sanni ga me da alluran rigakafin ciwon hanta ko ciwon shawara da a ke yi wa yara da zaran an haifesu?

Respondent: Wanan yakan kawo, tun rigakafin yanayin ko da baka da shi, yana da kyau, ka karba domin ka, ya samu in ya shiga jikin ka, ya samu kana da magani a jikin ka, bare iya ya samu karfi ya shiga cikin jikin ka ba, shi ya sa ana karban rigakafi, domin yana da kyau

Interviewer: Ok. Ama kin san abun akan wanan rigakafin na ciwon shawara? Da kan shi, kin sanni wani abu a kan shi?

Respondent: Babu

Interviewer: Babu ko? Toh ba damuwa. Ah, Tambay ana biyu da daya, menene ra'ayin ki a kan ita wannan alluran rigakafin ciwon hanta koh shawara da a ke yi wa yara da zarar an haifesu?

Respondent: Ra'ayi na yana da kyau domin a karbi wanan rigakafin. Ciwo ba wanda ana siyawa wai gashi wai koh bara bai kama ka bara ka karba wanan ba, yana da kyau ka karba alluran domin ka samu karfi, ka samu yanda za ka yi wanan abu ya zo ba zai same ka ba

Interviewer: Ok. Nagode da amsan ki. Tambaya na biyu da biyu, shin a baya, kin taba neman a yi ma yaron ko yarinya ki alluran rigakafin ciwon hanta koh shawara da a ke yi da zaran an haifu?

Respondent: Toh, ni kam, ban taba haihuwa ba, yanzu wanan ne na farko

Interviewer: OK

Respondent: Ama gaskiye zan so da in karba

Interviewer: Ok, toh sannu...

Respondent: Mm

Interviewer: tambaya na uku koh, shin kina da Shirin bar ma yaron ki ko yarinka ki a yi masa wanan alluran rigakafin ciwon hanta ko shawara da zarar kin haihu?

Respondent: A, zan karba. Wanan kam ra'ayi ne

Interviewer: Me ya sa za ki aminche da haka?

Respondent: Domin yaro na ya samu karfi, ya samu yanda zai yi, zai feeling a cikin rayuwan sa, yayi rayuwa mai kyau kuma abu ba re, cuta ba re sa me shi ba, wanan cuta banzan cuta ne

Interviewer: Toh a a che kuma uhm, ba ra ki aminche ba, me ne zai sa ba za ki aminche?

Respondent: Ah, duk wanda bai san abun da yana da kyau ba, shine wanda ba re aminche ba aman dai har ka san wanan abun mai kyau ne, ba yanda za ka yi ba za ka aminche ba

Interviewer: OK. So, ah tambaya na uku da e, shin kina da wani ra'ayi ko damuwawi ko dokoki akan ita wannan alluran?

Respondent: Erhhhh...amaaa...

Interviewer: kaman kina da wanni, wanni dokan da maybe kin dauka a rayuwan ki chewa a kan wanan rigakafin ko wani rigakafin ko kuma kina da wani dan, dan damuwa, a kan rigakafin?

Respondent: tohhh...

Interviewer2: Bari na dan shiga cikin maganan, a nufin shi ba, tunda kin che anan chewa kin so ki ba ma yaron ki domin ya samu uh uh karfin gwiwa da yada zai iya maganta cututuka da zai adabe shi. Shine aka fada chewa toh yanzu a ganin ki, kina gani akwai abun da zai sa ba za ki ba

ma…shin kina da wani (ko wani damuwa) dalili neh ko wani abunan da zai sa ki ba za ki ba wa yaronki din ba

Respondent: Ba abunda za isa, wanan ya zama, na kuduta neh dolle ne, in karba ne, domin yaro ya samu karfi

Interviewer: Toh a, tambaya na hudu ko, toh shin kina gani wanan alluran rigakafin ciwon hanta koh shawara da zarar an haihuwa na iya kare yaro ko yarin ki daga kamuwar da ciwon hanta? Wato kina kina kina kina gani kama idan an yi wanan rigakafin ma yaronki, yana da daman zai kawas mishi da kamuwa da wanan cutar?

Respondent: A, yana da daman kawas da wanan cutar domin ya riga ya karbi rigakafi, ba yanda zai yi, in ya zo zai samu ya riga ya yi wanan, ba rai iya kama shi ba

Interviewer: Ok. Toh, abun da za isa kika aminche kenan?

Respondent: A

Interviewer: Toh in kuma ba ki aminche ba fa?

Respondent: Zai iya ya kama shi, ba wani huja domin bai sa mu rigakafi din ba, shi ya sa zai iya samun karfi ya shiga jikin sa

Interviewer: Ok. Toh tambaya na biyar ko, shin kina ganin za ki samu ayi wa yaro ko yarinki alluran rigakafin ciwon hanta ko sharawa idan kika nema da zaran ki haihu?

Respondent: A

Interviewer: Me ya sa kika, umh, za ki nema a yi ma yaronki? Za ki tambaya, ayi ma yaronki?

Respondent: Mm, zan yada in nema ko da aya zai nema domin a na san wanan yana da kyau in karba shi ya sa zan nema ko a ya, in zo asibiti don in zo in karba

Interviewer: Ok. Toh, In zan dan kara tambaya, wato me ya sa kika fadi haka?

Respondent: Abunda ya sa na fadi haka domin na gane, na sanni yana da kyau, yana da muhimumci domin zai taimake yaruwan yaro, shi ya s azan zo in karba

Interviewer: Ok, toh tambaya gashi, shin kina da wani tunani ko matsala da kike gani zai iya hana ki samun wanan alluran ta ciwon hanta ko sharawa da ake badawa da zarar an haihu?

Respondent: Clears throat…Toh…(coughs)

Interviewer: Kina gani akwai wani abu wani matsala ko wani abunda zai hana ke? Samun wanan?

Respondent: Ba abinda zai hana ni domin mungode, gomnati yana iya taimako ya kawo mana wanan, in mun zo asibiti za mu samu, so shiya sa ba na shaka zai iya in zo in samu idan na zo, ba adunda kuma zai hana ni da bazan zo ba

Interviewer: Toh mungode. Tambay ana shida ko, shin kina ganin, kina gananin wanan alluran rigakafin da ake yi da zarar haihuwa ta ciwon hanta ko shawara tana da amfani ga yaro ko yarinya ki?

Respondent: A, tana da amfani

Interviewer: Me ya sa kika aminche?

Respondent: Don na riga na sani wanan ciwo ba ciwo mai kyau bane so kuma yayi kyau wadanda su fi mu ilimi sun fitor da wanan abu domin ya taimake yara. Shiya sa zan karba, na gane wanan abu yana da muhimanci, yana da kyau kuma zai taimake rayuwan yaro, shiya sa na aminche da wanan kuma in na karba, ba re taba ya same shi ba, shiyasa na bada zan zo in yi

Interviewer: Toh mungode. In kuma ba ki aminche, me ya sa haka?

Respondent: Wanan zan che ban san yadda wanan abu yana da muhimanci ba shiyasa ma ba ran ma aminche ba ma, idan ban san wanan yana da muhimanci ba

Interviewer: Ok. Toh, tambaya na bakwai, a jumlanche, shin kina ganin chewa mata masu juna biyu a nan yanki ku, na samun ita wanan alluran rigakafin ciwon hanta da shawara da zaran su haihu?

Respondent: A, suna samuwa sai wayanda da ba su yada ba, su basu sanni ba, shine basu zo ba, sunne wanda basu samuwa

Interviewer: Ok. So tambaya na bakwai da daya, me ya ba ki wanan tabache?

Respondent: Akwai wadanda basu sanni ba, yaransu sunna iya su kamuwa da shi, sunna iya su kamu dashi muna gani, shiyasa abun ya zo ya zo, da, ba a samun irin wadanan, ama yanzu kam ana samu; shiyasa, kuma mun gani yaran da gaskiya basu samu wanan din ba, suna kamu da wanan ciwo… Yan zu kam, wanan zamani ya changa kuma ana samu shiyasa na yadda wanan abu mai kyau ne a zo ana karba kuma ana fada a sanar a zo a karba shi ne ana zo a karba

Interviewer: Ok. Toh na nagode. Kamar yanda kika che, mata anan suna samun wanan rigakafin in sun zo sun nema ko?

Respondent: Mm

Interviewer: Toh yanzu me ne ya baki wanan karfafuwa da suna samuwa?

Respondent: Wanda ya yi me?

Interviewer: Me ne ya sa ki kin kina da wanan karfin jiki chewa za su samu ko suna samuwa?

Respondent: Abinda ya sa, ana ana fada so da dama, ana che idan yaro, ya kamata shi ya sa ana zuwa domin a yi gwaji idan rana ya kai, ana zuwa nan a haifu, domin muche abu zai dame wanan din, ana haihu zara ana haihuwa ana ba wa yaro din a maida, ana zuwa a haihu anan.

Akwai wayanda ma suna haifu a gida, ranan ma yana yi, suna iya su kawo, azo, su zo su karba. Domin sun gani yana da muhimanci.

Interviewer: Toh mungode… Tambaya na twakas, shin kina da wata shawaran da zaki iya bamu ta yadda zamu karar wayar ma allumma da sauran jama'ar ta wanan yanki game da muhimancin alular rigakafin cutar hanta ko shawara da ake bayar, badawa da zarar haihuwa? Akwai wani shawaran da zaki bamu, za ki bawa gomnati, a, a yanda za ki iya karar muku da ilimi game da wanan rigakafin hanta, ciwon hanta?

Respondent: A toh, mu muna godiya domin in gomnati yana iya ya kawo mana wanan, duk mungode domin ana iya kawo mu karba. Mu gaskiya, wayanda basuwa zuwa su zo su karba, har suna ganin yaron su ma, da wanda ya karba yana zama da banbanci, domin wanda ya karba yaron shi wayon ma yana fin yada, wayanda sun karba. Wanan ina bada shawara, duk matan da sun haifu, basu karba, su duba yan wayanda suna karba, yana nan difference. So zasu iya su zo suna karba, wanan ya zama tabas, in sun karba, yaron su changyuwa kam, yana yi; domin ko wanan ciwo ya zo, bare samu yaron din ba

Interviewer: Toh mungode. Ah, tambaya na twakas da daya, shin, za ki so Karin bayani ko sanni game da wanan ciwon hanta ko shawara? Za ki so ki kara samun bayani ko ilimi akan wanan ciwon?

Respondent: A

Interviewer: Ta wani hanya ne kina ganin zai fi miki a kara miki wanan ilimi? Ko ta hanyan labarai, ko a kawo miki video, ko poster, ko kuma sauran su, wanene kina ganin za zai fi miki?

Respondent: Inna son in gane mene wanda yana kawo wanan ciwon din domin ko ata cin abinchi ne ko yarda kana fita, abubuwa din ni ban fa'inta ba

Interviewer: Ok. Toh ta wani hanya ne kina son a kawo miki wanan din?

Respondent: Ko ta bayani, za iya a bani

Interviewer: Ok. Toh shikenan. Ta wace hanya kika fi son Karin ilimi game da sha'anin kiwon lafiya?

Respondent: A ta……

Interviewer: Ta wani hanya ne, kamar yanda kika fada, wanene, a cikin su duka kin fi so, in yanzu ina son in kawo miki wana ilimin?

Respondent: Ta, a ta bayani, yanda abubuwan za su iya su kama ka ko kuma abinda za ka iya ka yi yanda wanan abun bare iya ya kama yaron din ba ko kuma ya kama wanan din ba

Interviewer: Shin bayanin din nan, kin fi so a zo a zauna da ke ne a yi miki, ko kuma a yi miki video ko kuma ayi miki a pepan poster, ko kuma akwai wani hanya da kina gani kaman ya fi, ya fi miki?

Respondent: A'a, ko bayani a ta baki ma ya, an iya yi mini, zan dauka

Interviewer: Toh sannu. Tambaya na tara, kuma tambaya na karshe koh. Shin akwai wani abu da kike tunanin da ya kamata mu sanni game da wanan ciwon? Ko wani, ko ko akwai wani abunda kina gani ya kamata mu sani, game da wanan ciwon hanta?

Respondent: (Soft sounds)

Interviewer: Dan daga murya, kin che? Ba damuwa, kamar yanda na fada miki, ba amsa daidai, ba amsa ba daidai ba, ra'ayin ki kawai

Respondent: Wannan din ne inna son in sanni game da wanan ciwon…ban gane sosai ba

Interviewer: Ok. Toh, Akwai abunda kuma kina tunani ya kamata mu sanni game da hanyan yarde muke bada bayanin wanan abun? Akwai shawaran da kina ganin ya kamata za ki bamu game da yanda ake kara miki ilimi, kamar yanda kin che, kina son ki san wanan abu da kyau, ko ba haka ba?

Respondent: A

Interviewer2: Ba ri in zo dai, na ce ba, yanzu kina zuwa asibiti awu. Awun da a ke yi mukun nan ana gaya muku (not clear) wadansu abubuwa, toh, mu maza ne ko, abunan, bamu zuwa awu, kina ganin akwai wadansu abubuwan da kina son ki sanni game da wanan ciwon hanta, wanda mu, yakama ta mu sanni, ko kuma wanda kin gani, za ki bamu shawara, saboda muma mu kai gaba, ko gomnati zata iya wani abu a kai, shine muna so, in kuma akwai wani bayani da kina da shi, da za ki iya ki gaya mana, muna son mun dauka mu kai gaba, domin a yi aiki da shi (not clear..raining)

Respondent: A, shawaran shi ne za ku koya mana yanda za mu iya mu yi domin za mu iya mu sira ko daman bamu karbi rigakafin din ba, za mu iya mu sira a wurin da kuma kawo magunguna, ku sanni lailai wanan magunguna ya samu ya kawo, an zo ana bama mutane.

Interviewer: Toh, munngode sosai koh.

Respondent: Mm

Interviewer: Kamar yanda na fada, tambaya na karshe kenan mu ka yi wanan yanzu. Mungode sosai da time na ki da ra'ayun ki da kin ba mu. Sannu koh

Respondent: Uh Mm

Key Informant Interview of Pregnant Woman at State 1, LGA 4, Facility 8

Interviewer: Toh, inna kwanan mu?

Respondent: [name]

Interviewer: Kaman yanda na fada, sunana [name], inna nan anan da colleague na [name], muna aiki ne da CDC AFENET. Mun zo muyi dan tambayoyi ne akan rigakafi, especially na hepatitis. Wato ciwon hanta (She: Mm), ko shawara koh (She: Mm). Gomnatin [state] ta bada izinin wanan aikin da muna yi anan. So za muyi magana ne da ke game da abunda kin sanni da shawarin ki da ra'ayin ki game da abunda kin sanni da ana yi miki a wurin antenatal. Wanan tambayoyi ba zai fi minti talatin ba kuma tambayoyi guda tara ne. Kamar yanda na fada, ra'ayin ki za mu, za mu, muna son mu sanni, uhm, ba wani abun da ze, za ayi daganan da zai attaching da keh, ya hadu da key a nuna chewa ga wanda ya yi abu kaza. Ah, idan kin aminche ki yi wanan interview da mu, sai ki fada

Respondent: Na aminche

Interviewer: Ok toh, za ki dan taimake mu ki daga murya ko, don mu samu muryan ki da kyau koh

Respondent: Mm

Interviewer: Inna nan uhm, [facility name], Kola PHCC a Kola ward na [LGA name] da mace mai juna biyu. Don Allah shekarun ki nawa?

Respondent: Ashirin da daya

Interviewer: Ashirin da daya. Toh mungode. So ga tambaya na fari, me kika sanni ga me da ciwon hanta ko shawara?

Respondent: Kai, ciwon hanta ban sanni

Interviewer: Ba ki sanni ba koh?

Respondent: Mm

Interviewer: Ah, me kika sanni ga me da alluran rigakafin ciwon hanta ko ciwon shawara da a ke yi wa yara da zaran an haifesu?

Respondent: Wanan ma ban sanni ba

Interviewer: Ba ki sanni ba koh

Respondent: Mm

Interviewer: Toh menene ra'ayin ki a kan ita wannan alluran rigakafin ciwon hanta koh shawara da a ke yi wa yara da zarar an haifesu? Wato idan uh, mataye sun haihu anan, ana yi wa yaran su rigakafi, ana yi ma yara rigakafi daya daya daya har ga dan numbobi ko (She: Mm), So a cikin

su daya, akwai rigakafin da ake yi, ana kiran shi hepatitis B ko (She: Mm), so ah, me ne ra'ayin ki a kan shi? Rigakafin da ake ma yara, kina da wani ra'ayi akan shi?

Respondent: Babu, hakan ya yi

Interviewer: Hakan yayi?

Respondent: A

Interviewer: Wato yana da kyau kenan?

Respondent: A

Interviewer: Toh, shin a, a baya, kin taba neman ma yaron ko yarinya ki alluran ciwon rigakafin nan?

Respondent: Ban nema ba

Interviewer: OK, domin, me, me, me ya faru, me me ya sa

Respondent: Na haife yaron ya koma

Interviewer: Ok, ayya, sannu. Toh ga tambaya na uku, shin kina da Shirin bar ma yaron ki ko yarinka ki a yi masa wanan alluran rigakafin ciwon hanta da zarar kin haihu?

Respondent: A

Interviewer: Me ya sa kika aminche da haka?

Respondent: Domin saboda kara yayi girma ya dame shi

Interviewer: Ok. In kuma ache ba za ki aminche haka ba, kina da dalili?

Respondent: Babu

Interviewer: OK. So, shin kina da wani ra'ayi ko damuwa ko wani dokoki akan ita wannan alluran?

Respondent: Babu

Interviewer: Ok. Me ya sa haka?

Respondent: Saboda su wayanda suke bayarwa su sun nan abunda ya sa su sunna bayarwa

Interviewer: Ok, toh mungode, ga tambaya na hudu ko, shin kina gani wanan alluran rigakafin ciwon hanta da shawara da zarar an haihuwa, yana iya kare yaro ko yarin daga wanan ciwon?

Respondent: Ba ya karar wa

Interviewer: Kina ganin zai iya karewa, ya ya ya ya sa ah, yarinya ko yaro, indan, da zarar yayi girma, ba zai kamu da ciwon ba?

Respondent: Babu

Interviewer: Ok, me ya sa haka?

Respondent: Zai kamu da shi

Interviewer: Idan anyi mishi rigakafin?

Respondent: Mm

Interviewer: Ok, toh me ya sa kina ganin haka?

Respondent: Bisa ga irin chinye chinye da yaron yayi girma yana ki (He: OK), ko irin su mai haka nan

Interviewer: Ok. Toh mungode. Ah, question na biyar, shin kina ganin za ki samu ayi wa yaro ko yarinki alluran rigakafin ciwon hanta ko sharawa idan kika nema da zaran ki haihu? A wanan wuri?

Respondent: Gaskiya wanan ban sanni ba

Interviewer: Ok

Interviewer2: Which question

Interviewer: 5. Za ki nemi a yi wa yaron ki ko yarinya ki?

Respondent: A, zan nema

Interviewer: Toh, ah, kaman yanda kin che ba ki sanni ba, me ya sa kika gani haka?

Respondent: Domin ban taba kawo yaro nan ba

Interviewer: Ok. 5c. Shin kina da wani tunani ko matsala da kike gani zai iya hana ki samun wanan alluran ta ciwon hanta ko sharawa da ake badawa da zarar haihuwa?

Respondent: Ba na tunani

Interviewer: Ba wani matsala, ba wani abunda da ah, a turanchi ana kiran shi challenge?

Ba wani abunda zai hana ki?

Respondent: Babu

Interviewer: Ok. Question na shida ko, shin kina ganin, wanan alluran rigakafin da ake yi da zarar haihuwa ta ciwon hanta ko shawara tana da amfani ga yaron ki ko yarinyan ki?

Respondent: A, akwai

Interviewer: Ok, Me ya sa kika aminche haka?

Respondent: Saboda zai iya kare yaro da yawan cutar jariri

Interviewer: Ok, In kuma ache ba ki aminche haka, akwai dalili?

Respondent: Babu

Interviewer: Ok. A jumlanche, shin kina ganin chewa mata masu juna biyu a nan yanki ko, na samun ita wanan alluran rigakafin ciwon hanta ko shawara da zaran su haihu?

Respondent: Ban sani ba gaskiya

Interviewer: Ok. Me ya sa kina ganin haka?

Respondent: Saboda ni, lokacin da na haihu, ban zo nan ba, shiyasa ban sanni ba

Interviewer: Ok. Mungode. Ga tambaya ta twakas koh, shin kina da wata shawarin da zaki iya bamu ta yadda zamu karar wayar wa allumma da sauran jama'ar ta wanan yanki game da muhimancin alular rigakafin cutar shawara ko hanta da ake badawa da zarar haihuwa? Akwai wani shawaran da zaki, kina son ki gaya mana, a yanayin yanda zamu kawo muku ilimi da wanan cuta?

Respondent: Babu, ban da shawara

Interviewer: Ok, shin, za ki so Karin bayani da Karin sanni game da wanan ciwon hanta da shawara?

Respondent: Inna son in sanni

Interviewer: Ok. Idan kina so ki sanni, ta wani hanya ne kin ganin ya fi a kawo miki wanan ilimi? Kaman ko ta video ne, ko ta labarai ne ko kuma ayi poster ne, wanne ne kin gani ya fi miki?

Respondent: Ta labarai

Interviewer: A labarai?

Respondent: Mm

Interviewer: Ok. So, ta wace hanya kika fin son Karin ilimi game da sha'anin kiwon lafiya? Gabadaya, wanan ba na hanta kawai ba, gabadaya ci, a kan ah, shanin lafiyan ki, ta wani hanya ne kin gani ya fi so a kawo maki Karin ilimi?

Respondent: Wanan ban gane ba

Interviewer: Wato ah, abunda ina, ina, inna nufi shine, game da sannin komai sa, akan lafiyan ki ko, ta wani hanya ne kin fi so gomnati ko mutanen da sunna kawo muku taimako, ta wani hanya ne kin fi so a kawo miki wanan eh, labarin, wanan information?

Respondent: A gidan radio

Interviewer: Ta gidan radio?

Respondent: Mm

Interviewer: Ok. Toh, ah, question na tara koh. Shin akwai wani abu da kike tunanin da ya kamata mu sanni game da wanan ciwon?

Respondent: Babu

Interviewer: Toh, Akwai wani abunda kike son mu sanni game da hanyan yarde ake bayana a kan ta? Ake bayani akan wanan ciwo?

Respondent: Babu

Interviewer: Ko kuma a, akwai abunda kike son mu sanni game da wanan a, alluran ah, ciwon shawara da ake bayar ma mace da zarar ta haihu ake ba yaro, da zaran an haihu?

Respondent: A'a, babu

Interviewer: Ok, toh, mungode sosai koh. Mungode da lokacin ki da kin bamu da amsosin da kin bamu. Thank you very much

Transcription of KII with a RI Provider at State 1, LGA 4, Facility 10

Interviewer: (I) and the Respondent: (R)

I: As I said my name is [name] working with CDC-Afenet. I would like to ask you some questions about, how knowledgeable you are about Hepatitis B birth dose, but before then I need to ask you, will you, do you agree to participate in this assessment?

R: Yes, I agree

I: So I'm here in the [facility name]. I'm interviewing the RI In-charge, so we are starting by asking the first question.

I: What is your age Ma?

R: Age?

I: Yes, your age?

R: 23 years

I: You are RI in-charge?

R: Yes

I: So the first question; What are the biggest challenges for people living with hepatitis B in your community?

R: The biggest challenge, the one government should provide vaccine for them, adult vaccine, only for the child, children vaccine which they used to immunize them. So we are lacking vaccine for adult

I: So second question, what are the biggest needs to address Hepatitis B in your community?

R: Like she said we need more vaccine for adult in our community, we need more vaccines for adults. That’s all

I: Question 3a, is there a strong need for hepatitis B birth dose?

R: Yes

I: Why?

R: Because it helps us to prevent hepatitis B infection, if we have to get we could do it.

I: If no, why not?

R: If not, we are going…. I don't know how to put it (laughs)

I: RI, do you have any reason?

R: If not, we are going to educate them on how to prevent themselves from getting hepatitis B infection

I: Question 3b; Do others see a need for the hepatitis B birth dose?

R: Yes, they need

I: Question 4; Can you tell me about the current programs or practices for the hepatitis B birth dose in your clinic?

R: Current one?

I: Yes

R: I don't understand about the current one but….

I: To say now, if may be a woman gives birth to a son or a daughter here, ah in your own health facility, do you think, you have this vaccine ready to give within …to the baby of the mother?

R: Yes! We give the child at birth, because we have SDD here

I; Question 4a; Is the hepatitis B birth dose easy to get within 24 hours of birth?

R: Yes.

I: If yes, why?

R: Because we have the vaccine here inside the SDD

I: Okay if no, Why not?

R: If not! Paused

I: Who can administer hepatitis B birth dose?

R: Technical staff; RI technical staff, RI focal person, manager, RI In-charge that related with, giving or administer the vaccine. Because we have different technical staff, most staff like Lab technician wouldn't administer vaccine, pharmacist wouldn't administer vaccine but others they will

I: So 4c; What are the challenges or barriers to getting the hepatitis B birth dose vaccine?

R: What is the?

R: Lack of transport or eh transport (coughs) For example, now we are in this health facility, transport that will carry us from here to go and carry the vaccine from cold chain sometimes it would mm……laughs

I: May be a challenge?

R: Yes, it is a challenge

I: So no. 5, have you gotten information from participants regarding their experiences with the hepatitis B birth dose?

Let me repeat, have you gotten information from participants (caregivers) regarding their experiences with the hepatitis B birth dose?

R: No, we haven’t got it, only for penta but for hepatitis, and penta is a normal vaccine

I: Question 5a; what is the community understanding of the hepatitis B birth dose?

R: The community?

I: Yes

R: They understood because it is the vaccine that prevent them from getting hepatitis

I: So, this one now lead us to question 5b now, that say are people interested in getting the hepatitis B birth dose?

R: Yes, they are interested

I: So, 5c, if they're interested, can you describe what kind of specific information or messaging you have heard?

R: We have all, they have, they already know the potency of the vaccine. The vaccine is preventing the hepatitis; they have a lot of information about the hepatitis

I: So that's what I'm not trying to ask based on the question that can you describe what kind of specific information or messaging you have heard?

R: You or them?

I: Maybe from you have heard from them specific information they're getting about this vaccine from them since they have been coming to assess it here. So what's this information or messaging or getting from we heard from them?

R: I didn’t understand the question

I: Can you describe what kind of specific information or messaging you have heard?

2nd I: If I may chip in. We are just trying to find out, is there any feedback?

R: After the vaccine? Or maybe reaction/effects?

2nd I: Yes, after the vaccine or just whatever it is. So these are the information we're trying to get. Did they ever come to tell you thank you for this vaccine or whatever, you know, this vaccine? We don't like it or to tell, you know, remove these facilities from Yes. Right. So what specifically did they ever say about hepatitis vaccine? Is there anything specific?

R: No

I: Question 6. Do you have any ideas or suggestions for improving Hepatitis B birth dose vaccination rates?

I: Do you have any ideas or suggestions for improving Hepatitis B, birth dose vaccination rates?

Okay, there is a rates at the community level, at healthcare system level, at the provider level, these are the rates of the vaccination about hepatitis B birth dose. I don't know whether you have an idea or suggestion for improving them for improving the hepatitis B birth dose at the community level, do you have any idea or suggestion to increase or decrease the uptake of hepatitis B birth dose vaccination at the community level, health system level and provider level?

R: At this situation, our suggestion is to educate them to prevent themselves from contact to contact from getting hepatitis with the infected person for the community level

I: What about health care system level?

R: Health care system, we the health care workers, we have to prevent ourselves from getting the hepatitis infection from infected person from wearing hand gloves, regular hand washing and other safety ways for prevention

I: What of the provider level?

I: Well, it's all the same provider is the one that immunize the vaccine ba! so, he, he, he the provider also tried to avoid himself from getting banned using handwashing, hand gloves, hand sanitizers and others from infected person and injections safety when immunizing somebody you have to put the syringe inside the safety box, don't recap the needle

I: Question 6b; Are there specific strategies for education or awareness that might be most effective?

R: Through health education, through mobilization and the rest

I: Question 7; Can you tell me about your experience with the maternal tetanus vaccination?

R: My experience is, we know that the tetanus toxoid used to prevent, tetanus vaccine is use to prevent tetanus for the mothers and child within its womb

I: 7a; What barriers or challenges have read experience related to the maternal tetanus vaccination?

R: Barrier and challenge, the one we know it lead to death for the child or mother, it leads to convulsion for the child will jack like shaking, shaking. Also the mother, it leads to death for her

I: Now, question 7b; What successes have we experienced. What is working?

We mentioned about the challenges.

I: So now question 7b; What successes have you experienced? What is working with you?

R: Come again I don't understand

2nd I: The initial question I was trying to ask you about what are the challenges that you have experienced around this? And now this question is trying to ask you, based on what is it that you are doing about this, we'll move in a body that is working, it's making your work easier when it comes to tetanus vaccine in pregnant women.

R: Okay!

2nd I: Right! So there are best practices, there are things that is making it a lot easier for you know, like, we can go somewhere now. And then the challenges would be that the people are rejecting the vaccine. Right? So what are the things they did that worked for them? So they can be able to say, we were able to build houses for the people, and then the people who accepted the vaccine? So we're going to find out to you here in this locality now. What is it that you are doing that makes it easier for you, based on this? Like the experiences you have with all these vaccines, especially on this Td?

R: The easier way is when we immunized them with the vaccine they used to appreciate because it prevents them from tetanus diphtheria. And also the one I know we used to educate them on, about using the sterilized things in the community, like razor blades, knife, syringes and needles in the community and health facility.

I: Question 8, what types of health interventions have been the most successful in your community?

R: Types of interventions?

I: Yes. What services, what services you know, you have been working here and things keep coming. So

R: The most important intervention in our community is one about the ANC, the pregnant mothers they used to like drugs free one, two they like mosquito nets for themselves and their babies then drugs for treatment not for ANC routine, for treatment. They need free drugs in the community and the RDT

I; So and the second question that follow that one, and why have they been so successful?

R: They so what?

I: And you know, what types of health services have been the most successful in your community, and why have they been so successful?

R: That successful is ANC, the reason why it has been so successful is that, there is free drugs for routine ANC and immunization (RI) they are free, because our communities they need free, free things without charging them with money

I: So, the last question. Is there anything else you think we should know about hepatitis B?

R: You should know?

I: Yes, you think we should know, not we think you should know you understand

R: (Laughs) provide yourself (laughs)

2nd I: This question is we want to learn something from you, because you have been doing what we should know about it

R: Yes, the thing that we have to inform you with it to know is to help us to provide vaccine for us in the community for our people. If there are drugs for free, with you there, you should help us with it, drugs for hepatitis and vaccine for hepatitis plus

I: Then, because this question lead to another, one question but lead to one another, is there anything else you think we should know sharing information about hepatitis B? Is there anything you think you should be sharing information about hepatitis B?

R: Sharing information is, we have to join our hands and educate our community about the danger of hepatitis B. How it infects somebody easily in our community and we are trying to protect ourselves from hepatitis B and immunize ourselves

I: And the last one for the hepatitis B birth dose, is there anything you think we should know about it, hepatitis B birth dose? You know, hepatitis is just a general this thing, but hepatitis B birth dose is the one given to children within 24 hours. So do you think is there anything you should let us know about it?

I: About the hepatitis birth dose the one given within 24 hours, you know when we say hepatitis B that one is a disease but the B birth dose is a vaccine. So is there anything you think we should know about it?

2nd R: If i may say something, this vaccine B birth dose, mostly some of the women need that vaccine so…. I think if you can favour us you can help us by getting something that will attractive them something like gifts, I'm, I'm drugs - free drugs, and many things that will attract them, if you give them mostly the Fulani people if they still think free, free gifts they will keep on coming because of what you are giving them. But if to say there is nothing you just come inject their child, without anything they complaint that we only inject their children without

given them anything. So they need something that will attractive them, as for my own case I will say, like if you inject the person you give them PCM injection use this for free that will calm the pain, (“inna gani kamar”) Laughs…… PCM tabs – old school!

I: Thank you for giving us this opportunity to interview you, I think the questions are over, thank you

2nd I: CMAN In-charge thank you

TRANSCRIPTION OF OIC KII

FACILITY: State 2, LGA 1, Facility 2

S/N	MODERATOR	RESPONDENT
1	What are the biggest challenges for people living with hepatitis B in your community?	Well, the biggest challenges I guess is that they don't know where to access treatment and how to access the treatment, they don't know.
2	What are the biggest needs to address hepatitis B in your community?	The biggest need is eh eh one, creating awareness about Hepatitis B, provision of vaccine for prevention, I think
3a	Is there a strong need for the hepatitis B birth dose? (Why or why not?)	Yes, there is a strong need because it prevents the person from contracting the Hepatitis as he grows up.
3b	Do others see a need for the hepatitis B birth dose?	I as a health worker sees it as a need but non health worker may not see as a need unless you give the person correct information.
	But do you think others see it a need? Is there any need? Do you see a need for the Hepatitis, do others see a need?	Yes, others will see a need because if you tell the person that this is a killer disease, the person will see it as a need, others will see it as a need.
4	Can you tell me about the current programs or practices for the hepatitis B birth dose in your clinic?	Ok, current practice, formerly we were told that it is eh from birth to two weeks, so we used to give them birth to two weeks, at a point they say is eh at birth, we gave it to we gave it at birth then we were we were also told again that we can give it out to two weeks so as at now we give them from birth to two weeks
	That is the current practice in your clinic	Current practice, yes, ehn
4a	Is the hepatitis B birth dose easy to get within 24 hours of birth? (Why or why not?)	Eh eh is not all that easy unless the person born in the clinic but if the person did not born inside the clinic, the person might not be able to transport the child to the health facility for the vaccination, so it is not all that easy.
4b	Who can administer the hepatitis B birth dose?	Anybody, any midwife that gives that deliver the child can administer the birth dose, in the health center, the health personnel working in the health centers can also administer the birth dose
	Health personnel?	Yes
	Like?	Registered Nurse, CHEWs, JCHEWs

4c	What are the challenged/barriers to getting the hep B birth dose vaccine?	Well, the barrier, I guess none, because is always available in the cold chain in Nsukka, so the only barrier is transporting it from eh cold chain Nsukka to here, that is the barrier
5	Have you gotten information from participants regarding their experiences with the hepatitis B birth dose?	No, I have not gotten such information
	Nothing, there is no an information at all from any from those who have been coming here to have their hepatitis B dose	I haven't gotten information
	No information	Unh
5a	What is the community understanding of the hepatitis B birth dose?	Their understanding? In fact, they don't know because once you tell them that you give from 0-2 weeks, their own understanding is that within 1-2 weeks of birth they should provide their child for immunization within birth to 2 weeks.
5b	Are people interested in getting the hepatitis B birth dose?	They are not, specifically because they don't know
5c	Can you describe what kind of specific information/messaging you have heard?	Messages I have
	As per ah this hepatitis B birth dose, any information or any message you've heard about, any rumor	Ok I have heard and I just as I told you before that we are giving it from but to 2 weeks, then lately they told us that it is at birth we started practicing it then to some extent they say that we can give for grace those people that come within 1-2 weeks we can give so we started giving again 0-2 weeks Unh.
6	Do you have any ideas or suggestions for improving hepatitis B birth dose vaccination rate?	Yes, the idea I have is that there should be increase awareness, there should be increase awareness to the mothers in the village and even to the health workers, once the awareness is created that immediately you give birth try to look for where to give the child a vaccination

6ai	At the community level	At community level, once we disseminate that information they will be able to look for where to vaccinate immediately after and it will also help out in turning out the rate of delivery in the health facility
6aii	Health care system level	Yes
	Ok	Yes, because they will believe once they deliver here they will give them that vaccine, so there is no need moving around for the vaccine
	So, what is your own role here now to increase	My own role now is to increase sensitization and mobilization of women, pregnant women o
6aiii	Provider level	They should increase the rate of availing the hep B so that whenever they come it will be available
6b	Are there specific strategies for education or awareness that might be most effective?	Yeah, specific awareness like radio you know if they come to health facility you tell them but once they move out to the media they will be hearing it in media hearing it until the jingle when the jingle goes round that within the 24 hours you suppose to give your child something yeah it will make their morale to be higher they will say they've heard it in the health center o, so this thing is true, ok, I will do it.
7	Can you tell me about your experience with the maternal tetanus vaccination?	My experience?
	With the maternal tetanus, TT now	Well, TT is given once the pregnant woman comes to the health facility you give, then a month interval you give, six-months interval that may be after delivery, six-months interval you give, one-year interval you give then the other one is one-year interval so is given five doses for complete vaccination.
7a	What barriers or challenges have you experienced related to the maternal tetanus vaccination?	The barriers and challenges, sometimes the women, the pregnant women will forget, you give them appointment they will forget when they come they will come two weeks postdate you have to like start afresh or you have to like start afresh because they have sometimes they will just give one-month gap from the day you gave them so you have to like start afresh when you start afresh the

		person will come again forget it again you know so that is the most challenges we use to encounter
7b	What successes have you experienced, what is working?	The success, what is working is that you keep on jingling it you keep on telling them, re-echoing it once they come you re-echo it in health talk you keep on telling them telling them to remember
8	What types of health interventions have been the most successful in your community and why have they been so successful?	Ok, the health interventions sometimes when they were giving net free for those that have completed immunization women use to like yes today is my I must keep to the routine enh enh so because of all those stipends we use to give them like net it will make them to be trying to like come for immunization come for immunization so I belief also if there will be something that you give the woman if she brings her child for first immunization at birth it will also make the person to be looking for where to source for immunization
	So, that is routine immunization, then the reason why you think it is successful is because of the incentives they give to them	Yes
9	Is there anything else you think we should know about hepatitis B, sharing information about hepatitis B, or the hepatitis B birth dose?	I guess you will be the, you will be more in position to tell me eh about hepatitis B I guess you people should be more in position but specifically, there is none anyway

TRANSCRIPTION OF PREGNANT WOMAN KII

FACILITY: State 2, LGA 1, Facility 2

Age: 23yrs

S/N	MODERATOR	RESPONDENT
1	What do you know about hepatitis B	The only thing I know it is that, is a disease of the liver and its transmitted through fluid, like amniotic fluid from mother to the baby, then blood through sexual intercourse
2	What do you know about the hepatitis B birth dose?	I don't really know anything about that one
	ok, you have not heard it before, ok the hepatitis B birth dose is given to child at birth to prevent against hepatitis B	
2a	How do you feel about the hepatitis B birth dose? Do you think its important	Yes, I think its important because this birth dose I believe it will prevent the hepatitis B from getting to the child, so by giving it's a form of vaccines against hepatitis B, I think its important.
2b	Have you ever asked for the Hepatitis B birth dose before for other children?	No this is my first pregnancy
3	Do you plan to get the hepatitis B birth dose for your baby	yes, if its affordable
	They don't pay for it	ok, ok
	Do you plan to get the hepatitis b birth dose for your baby	Yes
3a	Why	If the hospital offers it, I will accept because I know it will be important
	Ok , but you will not ask them for it	Its just now that I know that they are giving birth dose to…
	So now that I have explained, will you ask them for it	yes, I will try and ask them, hehe..
3b	Do you have any feeling of concern, stress, and excitement having told you this?	eeh, it's a form of excitement because I know like we mothers should not take anything vaccine for granted because I know it will help the baby in future
4	Do you think the hepatitis B birth dose will work to prevent hepatitis B?	Yes, if it does not work, that means they should not be giving it now
	Why do think it will	I just it believe it will work because they normally give other vaccines for other sickness and all of them still work for the baby, so this one too should still work.

5	Do you think you could easily get the hepatitis B birth dose vaccine if you ask for it	I don't know, I will just ask for it
	As for the day of delivery if you ask do you think you will get	This is the first time so I will ask, so I don't know…
5a	Will you ask for it	Yes
5b	Why do you say that?	Just that it will help the baby
5c	Is there anything that might come in the way or challenges you might face related to accessing the birth dose?	I don't know whether the hospital, currently if the hospital has it, have the hepatitis B birth dose, so that's the main challenge, what if you ask them and they say they don't have it.
6	Do you feel the hepatitis B birth dose is important for you to get for your child?	Yes now
	Why do you think its important	So maybe the baby will be immune to that sickness now
7	In general, so do you think pregnant women in your region get the hepatitis B birth dose for their babies?	I don't think so, I don't know, but I don't think so
	Ok, you don't think they get it?	Yes
7a	What gives you that level of confidence?	Because this last one that yellow fever started killing people, everybody was rushing to health center to get the this thing. So had it been they have been giving it after birth maybe it will not have gone the…..
8	Do you have any suggestion for us if we want to educate people in your community about the hepatitis B birth dose	Yes, the suggestion is, we have like health centers in our communities, our rural areas; maybe you people should go there and enlighten people more about the hepatitis B birth dose, because last time the thing killed many people, especially in my mum's side, it killed many people, so I think you people should go there, ask the nurses there, the day that people normally comes in the clinic, maybe you will teach them more about this thing.
8a	Would you prefer to learn about hepatitis B? (Through stories or videos or flyers) how do you prefer to learn about it?	Stories, because I know videos might not be eeh available
	For you how will you prefer to learn about it?	Stories, if just tell me about it, I will learn
8b	How do you prefer to learn about health issues?	Just stories, if you just tell me about the health problem, there is no problem, I will know about it
9	Is there anything else you think we should know about hepatitis B, like sharing information about hepatitis B, or hepatitis B birth dose?	I want to know about this birth dose in particular and how it really works

TRANSCRIPTION OF PREGNANT WOMAN KII

FACILITY: State 2, LGA 1, Facility 5

Age: 32yrs

S/N	MODREATOR	RESPONDENT
1.	Kedu Ihe mara gbasara oria imeju a n'akpo iba ocha na anya	Ihe mu mara bu na afubeghim onye the oria na eme, mana otu ndi mmadu na ekwu, na the oria, na bia turn mmadu ahu a na achasi odo odo, ma ona anyu mmamiri ha ncha nile anacha odo odo, ma anya ya ana acha odo odo,So ha na akowata na the oria bia, na a ga akaba ahu zo onye ahu maka na o na egbu mmadu
2.	Kedu ihe i maara gbasara ogwu mgbochi iba ocha na anya?	Ihe mu maara bu na ndi noosu na akuzi anyi, na anyi ga na agbo mbo ka anyi bia ikute nwa je gba ya ogwu, nine months, maka na a na agba ya na nine months, ha na si gi ka ba ahu ka I di sure na igbazuru nwa gi ogwu, ka e wee gbochie the oria, maka na oburu na o na eme ndi mmadu ka oghara ife gi si te na ndi ozo.
2a	Olee otu obi di gi maka ogwu iba ocha n'anya?	Obi dim anwuri maka na nkea mere, Nke adult asi onye obula gba maka the oria, anyi ncha nile kabara ahu gbacha the ogwu, mu onwem na ezu n'ulo m, oge anyi gbachara the ogwu, na anyi enwetero ahu oku iba ocha n'anya ahu, oge ahu omere ndi mmadu, gbuokwa ndi mmadu. Obi di anyi anwuri na ogwu ndi goomenti na ebutere anyi na aru oru.
	Iba ocha n'anya nke anyi na ekwu okwu ya, enwere iba ocha n'anya abuo enwere n'ala igbo, nke a na ekwu okwu ya bu nke a n'agba umuaka oge amuru ha ohuru, amutu ha na ha, agba nke ahu	
2b	O nwetula mgbe I rioro ka agbaa umu gi ndi ozo ogwu iba ocha n'anya	N muo nwa, n ga agba onye ahu ogwu mgbochi
	O nwetula mgbe I rioro ka agbaa umu gi ndi ozo ogwu iba ocha n'anya	A bia go m rio ha mana ha gbara anyi ogwu
3.	I nwere atumatu obula iji nweta ogwu mgbochi iba ocha n'anya maka nwa gi,<br><br>O bu mu juo gi ozo,<br>I nwere atumatu obula iji nweta ogwu mgbochi iba ocha n'anya maka nwa gi, Nke I di ime ya	E nwere m atumatu inweta the ogwu, maka na a na agba anyi TT, maka TT ahu a na agba anyi ga eme na ahu ama n'eme nwatakiri, na iga agbali na aga ante natal, maka na ihe mere ana agara ante natal bu ka ha nwe ike na enye anyi ogwu, nke ga emekite nwatakiri no n'afo.

3a	Gini mere ma obu gini kpatara na I n'enwe atumatu ahu maka ije enwete ogwu mgbochi ahu	Ihe mu ji enwe atumatu, maka onye obula choro aru isi ike, maka I ma na oria karirizi n'uwa kita , ya mere anyi agbasi ike ka anyi buru ndi na agba ogwu ofuma, ihe nile asi anyi mee na health center, na anyi ga na agba mbo eme ya, a si anyi bia , anyi abia
3b	I nwere mmetuta o bula gbasara nchegbu, nrugide, obi uto?	Aghotarom question nkea
	I he n'aju bu ka I nwere mmetuta o bula gbasara nchegbu,dika ihe n'eche gi gbasra ogwu mgbchi ahu?<br>I ghotara ajuju m	Mba oo¸oge m muru nwa izizi, ujo na atuwa m, maka na ndi mmadu na asi anyi ejekwala gba ogwu mgochi maka na I gba nwa gi ogwu o nwuo , so mu onwe m wee kuru nwa m oge ahu gba ya ogwu , nwetazie experience, o kazie m obi, m mara zie na nwa obula mu muru na aga agba ya ogwu mgbochi
4	Ichere na i gba ogwu mgbochi oria iba ocha n'anya ga aru oru ma oburu na agba ya nwa amuru ohuru	Echere m na agba ya n'oga aru oru
	Gini mere iji chie n'oga aru olu	Ihe mere m ji chie n'oga aru olu bu na asi na mgbochi oria ka ogwugwo, na ibido n'oge zuo nwatakiri ndu , na onweghi ihe ga eme ya, mana I hapu nwatakiri n'ahu nwere ike ime ya
5	Ichere na I ga enweta ogwu mgbochi iba ocha n'anya B n'uzo di mfe ma o buru na I choo ya	Oge obula mu choro n'ulo ogwu a, maka oge mu onwe m biara n'ulo ogwu a, ana m enweta the ogwu, ma ndi ozo na akowa na ha n'enweta kwa ogwu ahu ebe a, I bia igba ya na a ga agba gi ya ebea
5a	Iga ario maka ya	I bia kowara ha, rio ha si ha biko, I ma n'enwere oge eji agba ogwu umuaka, ma na oburu n'ibia ma oge ahu agafuela, I bia gwa ha, ha ga si gi kute nwa ahu ka ha gba ya ogwu, ma obere nwata ma okenye
	I ma na Ogwu nkea anyi n'ekwu okwu ya, a n'agba ya ma a muo nwa, amutu hana nwa ka an'eji agba ya, oburo oge a na agba ogwu mgbochi , na obu ozugbo ozugbo amuru nwa ka an'agba ya, so nna aju gi ka I ga ario ka ha gba ya ka ha ga eji aka ha gba	Like oburu na I muo nwa ebea, ha ga agba ya, ozugbu I muru nwa, ha agba ya, mana oburu na I no ebe ozo muo nwa, na ikute ya, gwa ha, na ha agba ya
5b	Gini mere i ji kwuo otu a	Ihe mere mu ji kwuo otu a bu maka na amugo mu nwa ebea
	Ha gbara ya ozugbu ozugbo i muru nwa ahu	Eeh (yes)
5c	Onwere ihe obula nke puru ibia n'uzo ma obu nsogbu ndi ozo nwere ike igbochi gi i nweta ogwu mgbochi iba ocha n'anya nke a n'agba nwa a muru	Na oge a, onweghi ihe nwere ike igbochi ya

	ohuru	
6	O di gi ka igba nwa gi ogwu mgbochi iba ocha n'anya B di mkpa?	O di mkpa n'aru mmadu
	Gini kpatara I ji kwuo otu a	Ihe mere m ji kwuo otua bu na, maka na agba ya nwatakiri n'oge, ogwu ahu ga adi ha n'aru, oria a ma na aria ha
7	Na mkpokota, ichere na umu nwanyi di ime na mpaghara gi ga- enweta ogwu mgbochi iba ocha n'anya B maka umu ha?	Mu onwem n'echekwa oo, Maka na oburo onye obula choro igba ogwu mgbochi, ma na ndi nile choro, mu onwem n'eche na ha onwe ha ga acho igba ya
7a	Kedu ihe na-enye gi obi ike ahu (ma o bu enweghi ntukawsi obi inwere, I gwa m	Mu onwe m nwere obi ntukwasi n'igba nwa m ma obu mu onwe ogwu iba ocha n'anya n'omara nma. Ihe mere obi ji sie m ike bu na oburu na ahu oku n'eme nwatakiri, n'ikute ya na health center, enye ya ogwu na ona aru oru, ya mere obi ji sie mu ike na mgba ogwu ahu, na oria bia na ogaghi emetuta nwa m, ihe an'azo n'ogea bu ijide nwa, ihe obula asi gi mee na oge amuru nwa ahu na health center maka ogwu a bu ogwu eji echekwube nwa maka oria, na ijisike gba ogwu ahu otu okwesiri inye nwa, na gi onwegi muru nwa na obi ga esi gi ike, na oburu na oria ahu abia, dika measles, o buru na igbara ogwu nwa measles dika nwam nwanyi n'agbara m ya ogwu measles, mana oge measles na aria ndi mmadu, omechara puta ya na aru mana onweghi ihe omere ya
8	Inwere aro obula i enye anyi ma o buru na anyi choro ikuziri ndi mmadu n'obodo gi gbasara ogwu mgbochi iba ocha n'anya nke a n'agba nwa a muru ohuru?	Aro m ga enye unu bu na unu ga ezisie ozi, zie ndi mmadu ka ha bia na health center,na achoro ikuzi ihe gbasara ogwu iba ocha n'anya B, maka na onwere ndi na abiabeghi ka onuru, mara ihe ogwu ahu na aru, o noro n'ulo, ndi mmadu ana asi onye ahu agakwala ebe ahu, mana akuo ogene si ndi mmadu gbakuo na onwere ihe di mkpa achoro ikuzi na health center, na onye obula ga abia ka o ge nti, na nti ahu onyea ga ege, ga akuziri onye ahu otutu ihe, mekwa ka obi sie onye ahu ike gbasara ogwu ahu
8a	O ga amasi gi imu gbasara iba ocha n'anya (site na akuko ma obu vidiyo, ma obu akwukwo okwa, n'uzo ndi ozo di iche iche, I oga omasi gi imu gbasara ihe iba ocha n'anya	O ga amasikwa m
	Kee nke ka masi gi	Di ka i na bia nkuzi, a na akuzirim ihe ufodu cha, gbarasara ogwu ahu, oga a dim nma, ka mu onwem nwe ike imuta, matakwa ihe o na aba
8b	Kedu otu kachasi gi nma imu gbasara nsogbu ahu ike? Nsogbu ahu ike nile	O buru na asi anyi biawa ka akuzibem, maka mu onwem, amaghim maka onye ozo, mana mu onwe

	an'enwe, O buro soso iba ocha n'anya, kee otu kachasi gi nma imu gbasara nsogbu ahu ike nile a n'enwecha<br>O bu mu kwu ya ozo<br>Kedu otu kachasi gi nma imu gbasara nsogbu ahu ike nile an'enwe, kee ka i ga esi choo ka ana akuzi gbasara nsugbo ahu ike	m ga abia
	Kee ka isi choo ka akuzie gi ya	I biawa ebea ka ana akuzi anyi , maka na aburom noosu o buru na mu na abia , ha si mu weta akwukwo na pen, ka na akuzi anyi obere obere, o na abanye, maka na anyi na ha aburo otu, ha onwe bu ndi noosu gara akwukwo gbasara health, mana mu onwem agaghi akwukwo, ha si m biawa ebea ka ha kuzie mu, na m ga abia
9	O nwere ihe ozo I chere na anyi kweisri ima gbasara iba ocha n'anya, ikekorita ozi gbasara iba ochan.anya, ma o bu usoro igba nwa a muru ohuru ogwu mgbochi iba ocha n'anya<br><br>O nwere ihe ozi iche n'anyi kwesiri ima gbasara ya	Ima, Aghotaghim ajuju gi
	Ihe anyi n'aju bu, ka onwre ihe ozo ichere na anyi kwesiri ima gbasara iba ocha n'anya	O bu ima?, di ka igbasa ozi
	Ma obu igbasa ozi, otu oria ahu si eme mmadu	Abia n'ulo uka , enye okwa,maka ndi n'eje uka kariri ndi na adighi eje uka , oburu na enye okwa n'ulo uka agwakwa ndi oje ozi ka ha kuo ogene zisie ozi maka ndi na adigh eje uka nuru, nke ga eme ka ndi mmadu nuta rie nne gbasara ozia, ma obu izi ozi na rediyo ka ndi na ege rediyo nutakwa

TRANSCRIPTION OF RI PROVIDER KII

FACILITY: State 2, LGA 1, Facility 5

S/N	**MODERATOR**	**RESPONDENT**
1	What are the biggest challenges for people living with hepatitis B in your community?	Hepatitis B?
	Or you don’t know what hepatis B is all about	I know, I know
	So What are the biggest challenges for people living with hepatitis B in your community?	In that community I am working ba? The challenges we have about hepatitis B, they are coming for immunization, once they are immunized against hepatitis B, they are covered
	So are you telling us that no any	We have not seen any case of hepatitis B in that place
	There is no any case of hepatitis B in your community	Yes
2	What are the biggest needs to address hepatitis B in your community?	Biggest?
	What are the biggest needs to address hepatitis B in your community?	Need is that we address them to come for immunization, because once they are immunized they are fully protected
3a	Is there a strong need for the hepatitis B birth dose? (Why or why not?)	No
	Birth dose, hepatis B birth dose, you don’t understand what hepatitis B birth dose is	I don’t understand
	You are the RI focal person	Focal person, hun
	Hepatitis B birth dose	Birth dose, ok dose, dosage
	Yes birth dose	Ok, ok at birth
	Yes, ok is there a strong need for the hepatitis B birth dose? (Why or why not?)	Strong?
	Strong need, need for hepatitis B	Yes, there is need now, there is need because once they are immunized, it will help them to prevent the disease
	So there is a need	Yes
	Then why is there a need? You said once they are immunized, they are protected?	Yes, they are protected
	So that is why there is strong need for it	Need, yes
3b	Do others see a need for the hepatitis B birth dose?	Do
	Others, do you see it as a need	Yes yes
	Do others see it that there is a need	Yes, they see it, they are coming for the immunization
	They are coming for the immunization	Yes
	Ok, they are so used to the other routine	Yes

4	Can you tell me about the current programs or practices for the hepatitis B birth dose in your clinic?	The current program?
	Or practice for the hepatitis B birth dose in your clinic	Current, we are doing the this thing, immunization every Monday
	Every	Every Monday
	Every Monday	Enh, you collect the vaccine, those people that came for the immunization injection even other antigens we include that hepatitis B
	We are talking about birth dose o	Birth dose? Yes, once they deliver we give them hepatitis B if there is hepatitis B immediately or if it is on immunization day
	Ok how do you do it in your own facility	Immediately after birth we give them
	If the mother deliver in the night?	In the night
	Yes	That is what I say, if it is the time of immunization but if the mother deliver in the time we are not doing it may be they have gone home, they will come within 2 weeks, we advise them to come to the clinic within 2 weeks
4a	Is the hepatitis B birth dose easy to get within 24 hours of birth? (Why or why not?)	Yes, because we have solar, we use to collect it from the cold chain and store it in the solar
4b	Who can administer the hepatitis B birth dose?	Every health worker can do it our facility
	In your facility	Yes, every health worker
	Every	Health worker
	Either trained or no training	That is why I say health worker, trained one
4c	What are the challenges/barriers to getting the hep B birth dose vaccine?	No challenge
	No challenge?	Unless, the challenge is not rare, is not rare
5	Have you gotten information from participants regarding their experiences with the hepatitis B birth dose?	No
5a	What is the community understanding of the hepatitis B birth dose?	They are complying once you tell them what the hepatitis may cause they come
	Ok they will come?	Hun

5b	Are people interested in getting the hepatitis B birth dose?	Yes
	How do you know?	They are coming out for the immunization, they are coming once they give birth even those deliver at TBA they use to come
	At TBA too	Yes, they use to come
5c	Can you describe what kind of specific information/messaging you have heard?	Repeat the question
	Ok can you describe what kind of specific information/messaging you have heard?	We use to tell them receive that hepatitis B immediately after birth is most important thing because that hepatitis B does not have any medicine it is only preventive medicine that is the vaccination
	What is the feedback you hear from the people in the community or the mothers	They will accept now, they will bring the child for immunization
	So there will not be anything negative	No
6	Do you have any ideas or suggestions for improving hepatitis B birth dose vaccination rate?	Idea I have is that the WHO or whoever is providing the vaccine should make sure that the vaccine is available all the time
	They should ensure that the vaccines are available	For replete
6ai	At the community level	I will encourage them to bring their child
6aii	Health care system level	You mean those working in the
	Within the health care system level, what is your own, what is your suggestion?	My suggestion is that we the health workers should make sure that we vaccinate the child at the appropriate time
6aiii	Provider level	Those who
	At the national level the provider	Is for them to supply the vaccine
6b	Are there specific strategies for education or awareness that might be most effective?	Repeat
	Are there specific strategies for education or awareness that might be most effective?	Strategies
	What strategies or	Strategies we use, at times we conduct outreach session so that those people that may delay or those at hard to reach areas we reach them during outreach

	Ok do you think it is effective?	It is effective
	Most effective?	Yes
7	Can you tell me about your experience with the maternal tetanus vaccination? TT now	Enh it is very important because once a woman is pregnant the woman is supposed to take that TT at least twice before delivery
	Is that the practice	Yes
	Do they now come for it?	Enh they come
7a	What barriers or challenges have you experienced related to the maternal tetanus vaccination?	No. I don't think there is any barrier I have experienced
	So the vaccine is always available	The vaccine is always available
	And people always	And people will come to take the vaccine
7b	What successes have you experienced, what is working?	Success?
	Yes	Enh enh, the success that we now, yes you mean the hepatitis B abi the TT
	No, TT, we are now at the TT	The TT, enh enh, since that TT has been established we are not seeing cases of the maternal tetanus or neonatal tetanus
	So what now is working? What is working? For them to come, for the pregnant women, from their own side, what is working?	It is working
	They come at the normal time to have the	The TT
8	What types of health interventions have been the most successful in your community and why have they been so successful?	What type of what?
	What types of health interventions have been the most successful in your community and why have they been so successful?	Health?
	Yes	Health interventions
	What types of health interventions have been the most successful in your community and why have they been so successful?	Health interventions
	Do you understand what health intervention mean, like malaria elimination program, you know	We are conducting that malaria session
	Which one has been so successful?	Immunization
	Ok	They use to comply. Malaria, they are coming one by one but immunization they use to come enh, enh in big number

	Is it routine immunization or campaign?	Routine, routine, even routine immunization even outreach they use to comply
9	Is there anything else you think we should know about hepatitis B, sharing information about hepatitis B, or the hepatitis B birth dose?	No, no again
	You don’t have any	Hun

TRANSCRIPTION OF COMMUNITY VOLUNTEER KII

FACILITY: State 2, LGA 1, Facility 6

S/N	**MODERATOR**	**RESPONDENT**
1	What are the biggest challenges for people living with hepatitis B in your community?	Convulsion and the eye problem and liver
	You say what?	Eye problem and liver
	You first said convulsion	Convulsion, convulsion
	Convulsion, eye problem and liver	Yes
	And Liver?	Yes
	Liver what?	Liver problem
	Liver problem	Yes sir
2	What are the biggest needs to address hepatitis B in your community?	I don't know
	You don't know?	Yes
3a	Is there a strong need for the hepatitis B birth dose? (Why or why not?)	Birth dose, 0.5
	No, the dose you received at birth	Birth, 0.5
	They are not asking about that dose	Hun
	But once a child	Ok once
	Once a child is delivered, that child should be able to have access to that vaccine immediately	Yes
	That is what we call birth dose	Ok, ok
	Is there a strong need for the hepatitis B birth dose? (Why or why not?)	Yes
	Why?	The thing help the child to prevent hun another sickness like infection, other sickness
	Ok, the vaccine will help that child	No, if you give the child the injection after birth, the injection will help the child to prevent any infection
	To prevent any infection	Infection. Yes
3b	Do others see a need for the hepatitis B birth dose? You see it as a need now	Yes
	As you see it as a need do other people also see that need?	Yes
	How do you know?	Because, everybody, if everybody become informed the person will try to give the her baby the injection because the thing help the baby because of all the sicknesses from sicknesses

4	Can you tell me about the current programs or practices for the hepatitis B birth dose in your clinic?	Because the injection, the drug is here so every time, any time you need it so is here
	You have it readily available	Yes is here after birth you give the child
4a	Is the hepatitis B birth dose easy to get within 24 hours of birth? (Why or why not?)	Yes
	I said how	Because is around is here is here
4b	Who can administer the hepatitis B birth dose?	Nurse, health worker, health worker
	Health workers like	CHEW, JCHEW
4c	What are the challenged/barriers to getting the hep B birth dose vaccine?	Because sometimes the thing is not here, you go for Enugu or Nsukka to get it
	To get it?	Yes
	So sometimes it is not readily available	Yes
5	Have you gotten information from participants regarding their experiences with the hepatitis B birth dose?	Yes, because some people come back because they complain the baby crying because of that thing or the leg just swell
5a	What is the community understanding of the hepatitis B birth dose?	The community understand the birth dose because the birth dose helps the children because of the infection other disease
5b	Are people interested in getting the hepatitis B birth dose?	Yes sir
	How are sure?	Because everybody since I came here everybody like to give her baby the injection
5c	Can you describe what kind of specific information/messaging you have heard?	Outside?
	Yes	About the injection?
	Yes	I have no idea
6	Do you have any ideas or suggestions for improving hepatitis B birth dose vaccination rate?	Yes
6ai	At the community level	Hmm try to bring the injection come here, the injection dey here any time because sometimes you will not see it here you go for Enugu or you tell person come back next

		time and before person come back next time the thing don ready pass so that is the thing
	So now	Like last month now almost two days the thing has not been, we go to Nsukka the thing is not there that is the thing
6aii	Health care system level	Hmm your own people
	No no no within your own	My own work
	Yes here in the health center here	We try to complain, complain about it so those people should bring more about the vaccine
6aiii	Provider level	We tell them to bring more, more vaccine about it because this vaccine some people, every day we have child almost three persons, three people or four so you try to bring more, more vaccine here
6b	Are there specific strategies for education or awareness that might be most effective?	Yes
	So what can we do?	We go to any Church announce that place or any villager meeting announce that place so
	Will that one be most effective?	If you go Church announce in that place and you go any village announce so the thing will everybody will hear about it
	Everybody will hear about it	Yes
7	Can you tell me about your experience with the maternal tetanus vaccination?	Enh, I don't understand
	I said Can you tell me about your experience with the maternal tetanus vaccination?	My experience about it
	Enh, what do you understand by, you own experience	Ok, the TT is just help all the pregnant woman to prevent to her baby all the some disease and pregnant woman is supposed to inject at least twice before birth or three times before birth or if another person want to get herself as just complete the all dose, she will come this, and if she come this month like this month August, she will come next month September, like today is 25th, she will come 25th next month, that is one month. after that one month, after that second dose, she will come on six month she will come complete the three doses, she will come one

		year to complete four dose because the thing have 5 dose so, but if a pregnant woman cannot complete it like that but at least two is ok for the pregnant woman
7a	What barriers or challenges have you experienced related to the maternal tetanus vaccination?	Any challenge? Oyes some people will come back complain they have like her hand just weak she cannot use the hand carry anything because of the injection. Some people have that problem but sometimes we give that person paracetamol the thing will go like that
	Any other thing you want to add?	No
7b	What successes have you experienced, what is working?	About?
	The maternal TT we are talking about, what success? People coming?	Yes
	What success	Yes
	How many of them coming the way you asked them to be coming?	Some people come if the somebody have any problem the person will come inject herself
	You said they ought to complete like 2 or 3 do they really come and complete it?	Yes
	All of them?	Yes. If the person come this month, you tell the person that the person is supposed to come next month, so September the person come back again come inject herself, you give the person the normal date if the person want to complete all the doses you give the person the date any day the person come back and bring that card because we have, sometimes you bring some just paper like that you write for them, the person use it
8	What types of health interventions have been the most successful in your community and why have they been so successful?	Health?
	Health interventions. All the programs you have been doing in health	Like
	Malaria	Treatment
	Yes which one either malaria treatment	Malaria treatment
	It could be treatment, it could be elimination, distribution of nets , interventions like that, what government brought to people	Government brought up
	Like the program government brought to people, they normally bring to people	Like net?
	Distribution of net	Net

	Health intervention, that this one is used to prevent this one, it could be ivermectin distribution all those kind of, which one has been the most successful?	Well, since I came here I never see any net here only as in
	So they never do any program	Yes
	Like NIDs	Mectizan, Mectizan
	Ok they distributed Mectizan	Yes
	Which of these ones has been most successful, which of them has people embraced, and they really like, you don't need to trouble them, you don't need to	Mectizan, because some people came here come collect mectizan, sometimes we carry it go for church
	Ok they normally collect it	Because of those people like, these people they are use to go farm
	They like Mectizan	Yes
	You don't trouble them, you don't run after them before they come	Yes
9	Is there anything else you think we should know about hepatitis B, sharing information about hepatitis B, or the hepatitis B birth dose?	I don't have other thing to say

TRANSCRIPTION OF PREGNANT WOMAN KII

FACILITY: State 2, LGA 1, Facility 6

Age: 37yrs

S/N	MODERATOR	RESPONDENT
1	What do you know about hepatitis B	Hepatitis B is liver disease
2	What do you know about the hepatitis B birth dose	Hepatitis B birth dose is the vaccine which we use to give baby that is delivered immediately
2a	How do you feel about the hepatitis B birth dose	Hepatitis B birth dose is very nice to baby
2b	Have you ever asked for hepatitis B birth dose before for other of your children	No
3	Do you plan to get the hepatitis B birth dose for your baby	yes
3a	Why	Because they said that hepatitis B is good for a baby
3b	Do you have any feelings of concern, stress, excitement?	Yes
4	Do you think the hepatitis b birth dose will work to prevent hepatitis B	Yes, that's what they said
	Why	Because they said that hepatitis B birth dose prevent hepatitis B disease
5	Do you think you could easily get the hepatitis B birth dose if you asked for it?	Yes
5a	Will you ask for	Yes, when I deliver I will ask for it immediately I deliver I will ask for it
5b	Why do you say that	Because you told me that hepatitis b vaccine will prevent my baby from hepatitis disease.
5c	Is there anything that might come in the way or challenges you might face related to accessing the birth dose	I don't know, because I have not received it before
6	Do you feel the hepatitis b birth dose is important for you to get for your child	yes
	Why	Because you told me that hepatitis B vaccine will prevent my baby from having hepatitis B disease
7	In general, do you think pregnant women in your region get the hepatitis B birth dose for their babies?	No
7a	What gives you that level of confidence	Because eem…you people , you are not used to come to our side and tell us that you have hepatitis B vaccine that the hepatitis B vaccine will prevent this kind of disease except today that you come to our side and tell us

8	Do you have any suggestion for us if we want to educate people in your community about the hepatitis B birth dose?	Yes, if you want to and educate us about hepatitis B vaccine, you will come and meet our community chairman
8a	Would you prefer to learn about hepatitis B? (through videos, flyers, other methods	I will like to learn it through flyers, through stories and even through video self
8b	How do you prefer to learn about health issues	Health issues like …
	Anyone, any health issues at all, how would want to learn about them	Like AFP, I will like it by flyers , and videos and through contact, close contacts
9	Is there anything else you think we should know about hepatitis B, sharing information about hepatitis B, or the hepatitis B birth dose	No

TRANSCRIPTION OF PREGNANT WOMAN KII

FACILITY: State 2, LGA 1, Facility 8

Age: 26yrs

S/N	MODERATOR	RESPONDENT
1	What do you about hepatitis B	I don't really know much, I think it's infection or whatever, I don't know
2	What do you know about the hepatitis B birth dose?	No idea
	No idea, how do you feel, no you have never heard of it, no hepatitis b birth dose as I said is vaccine given to children at birth and it given to prevent against hepatitis B, children born at the first day of delivery are given this vaccine and most times mother do not know that they are supposed to give their children that vaccine and most health workers do not know, they will always tell you to come back during the RI day	
	So, how do you feel about the hepatitis B birth dose	Feel as in how
2a	How do feel, do you think that its important	Of course, it's important , if it is going to help the child
2b	Have you ever asked for the hepatitis B birth dose	No
	You have never	This is my first
3	Do you plan to get the hepatitis B birth dose for your baby	Sure, if there is no side effects
	As I have explained, the important of the vaccine, do you plan to get that for your baby	yea
3a	Why	I think like I said, maybe to prevent the child from having the hepatitis, because if it's not important I don't think the doctors will want to give the dosage or the vaccine
3b	Do you have any feelings of concern, stress, excitement	No, just normal
	You don't see it as an important something?	Of its important I have said that before
4	Do you think the hepatitis birth dose will work to prevent hepatitis B	Yes, of course
5	Do you think you easily get the hepatitis B birth dose vaccine if you	Hopefully it will be available by then

	asked for it	
5a	Will you ask for it	Sure, since its important
	You will remember to ask for it	Of course
5b	Why do you say that	It's an important vaccine
5c	Is there anything that might come in the way or challenges you might face related to accessing the birth dose	I don't think so, Unless maybe its problem coming from the hospital or whatever
6	Do you feel the hepatitis B birth dose is important for you to get for your child	Definitely , I said that its important
	Why	It's something that's being brought by medical practitioners, so I think it's important for them to be vaccinated
7	In general, do you think pregnant women in your region get the hepatitis b birth dose for their babies	I don't really know about that
7a	What gives you that level of lack of confidence	I don't know, this is the first I'm hearing this
8	Do you have any suggestion for us if we want to educate people in your community about the hepatitis B birth dose	Of course, since it's an important vaccine, I think most pregnant women should know about it, because majority of them including me, don't know about it, since its important, I think when you tell them the benefit of it, so everybody want to take it for their child
8a	Would you prefer to learn about hepatitis B? (Through stories or videos, flyers or other methods	Through teachings now, like during antenatal they can come and teach us and ... so many people will want learn more about it
8b	How do you prefer to learn about health issues	What me I do most times, is I go through the internet, but if there is any other means available maybe teaching face to face or maybe yea, it will be better
	You prefer face to face	Yea, at least you can ask question
	Just like the ante natal too	yes
9	Is there anything else you think we should know about hepatitis B, sharing information about hepatitis B, or the hepatitis B birth dose	I think, just what I know is it should be.., a lot of persons should get to know more about it, like, there should be more flyers or publicity about it, so more persons should get to know about it

TRANSCRIPTION OF DOCTOR KII

FACILITY: State 2, LGA 1, Facility 10

S/N	**MODERATOR**	**RESPONDENT**
1	What are the biggest challenges for people living with hepatitis B in your community?	They are not covering any specific thing; we discover them by chance. Sometimes they don't even know, they don't know that they have such a case
2	What are the biggest needs to address hepatitis B in your community?	I agree that they from childhood they should get some vaccination and they if it may be routine, it may be routine, if they can make it a routine something to test some patients whenever they come here because they complain just like others, so the complain the lay looks like as if it is like any other case except when they have liver problem and enlargement or yellowness of the eyes or significant weight loss you start investigating to know the cause you know to know whether it is or not, and we use to carry out laboratory investigation especially those who are having retroviral cases use to show it more, what I mean by retroviral cases that is HIV, immune and response, yes it manifests sometimes, others don't even as unless it shows just routinely find out that they have it and usually they don't belief, they don't belief that they have it until it start disturbing them and the symptoms and signs do not differ from other common sources of ailment around them.
3a	Is there a strong need for the hepatitis B birth dose? (Why or why not?)	Yes, if it is so it there is need, they can make it compulsory just as the va
	Ok why is there a need for it?	Enh to prevent infection, and you say is eh neonatal, at neona, they are neonate mainly because just within few hours they could be doing it along with eh this eh antiretroviral, antiretroviral is immunity after birth enh enh so it never appeal, if they shoot in that whether you have it or not you will receive it
3b	Do others see a need for the hepatitis B birth dose?	Enh?
	Do other people see the need for hepatitis B birth dose?	Other people?
	Others may be the health workers or even people around you do they see the need for it?	Do they know herself? The most important thing is that do they have the awareness? They don't. But you say birth dose but they give eh other routine vaccination
	Ok, they are so used to the other routine	Yes
	Not the birth dose	Not birth dose, I have not heard,(stammering)
	You are just hearing it for the	Eh no o
	It might not be the first time but you are not used to it	But you say within 24

	Yes	I am not used to it o
4	Can you tell me about the current programs or practices for the hepatitis B birth dose in your clinic?	I don't know, I said no, because they don't even do the vaccination, it is not carried out
4a	Is the hepatitis B birth dose easy to get within 24 hours of birth? (Why or why not?)	What they are practicing I say I do not know. Is it here in the hospital?
	Yes, this facility. Do you know whether it is easy to get within the facility?	I don't know, I don't know that is why I said I don't have enough eh
	You don't so	I don
	You don't have enough knowledge?	Yes about what they do the activity, whether they give it or do not give it
	But patient that eh like neonate they don't em	Hun, they must if they are practicing it here they may be, they may be giving it, that is the, that is the issue here. If to say they will get awareness a kind of eh , yes awareness through workshop or visitation here then they will know the needs
	But you don't know actually know whether it is accessible here in this facility	No. But you see the (stammering) what you say is birth dose
	Yes, within 24hrs, you know if it is handy, if it is handy it will be accessible within 24hrs	Hepatitis B
	Yes, birth dose	What are the other vaccine
	At birth, as they are giving other eh you know BCG too is at birth, OPV too should be at birth but hepatitis B too at birth, if you go, there is a guideline, if you get to that their this thing it is pasted there I just look at it the routine Immunization when and when the child should receive each of these thing at birth you will see that hepatitis B is there	BCG, yes, at birth, enh<br>I think even Meningitis
4b	Who can administer the hepatitis B birth dose?	It is the, it may be the midwife or nurses now
	Midwives or nurses	Or CHEW even CHEW, CHEW personnel or JCHEW, any of them
4c	What are the challenged/barriers to getting the hep B birth dose vaccine?	Number one is the knowledge of the mother about the need and the availability of that vaccine at every facility that conduct en, you know labor and delivery
	Is at the facility or at the labor room which one	The labor room is inside the facility now so if they know it, it will always be there

5	Have you gotten information from participants regarding their experiences with the hepatitis B birth dose?	No, have you ever taking interest in that on your own
5a	What is the community understanding of the hepatitis B birth dose?	Community? The community?
	Community, the understanding of it	No, they don't discuss, do they know are they, do they know what they are discussing? They will just say may be you know my child was vaccinated
	But you know if they know, if the community is aware of the birth dose when they come to facility they will insist that should be given at birth not to wait till..<br>so what do you think the community is not understanding of the Hep B birth dose?	Yes<br>If there is enough knowledge<br>They know, they have known about immunization so along with other routine immunization<br>I don't think anyone know, they don't but nurses may know o because I don't go at their immunization these days so I don't know what they are doing there. And they have a kind of cold is it cold chain system that they every time women are coming here for post natal administration of other vaccines
5b	Are people interested in getting the hepatitis B birth dose?	Yes, anybody who knows what it is cannot unless the person did not deliver at the hospital but they do not object vaccination, they don't.
5c	Can you describe what kind of specific information/messaging you have heard?<br><br>You've not heard any info, nothing?	Messages I have heard<br>No<br>About first dose or what do you call it birth dose no<br>Maybe it is due to the terminology you are using
	No, that is the right terminology, birth dose it was given at birth that is why it's called birth dose	Yes now listen when you say birth dose you know so some people will think that there's a different dosage do you understand? Is it correct?
	No no no that's not what it means No it's not a different dosage birth dose means that dose is given at birth	Ehn okay
6	Do you have any ideas or suggestions for improving hepatitis B birth dose vaccination rate?	The rate?
6ai	At the community level	Okay. Education we educate them during which we demonstrate and tell them the importance of that and tell them the consequences. The consequences if they fail to they can lead to certain disease conditions as you said like

		Hep B and C. Immediately they hear it nobody will like to be lost (stammering).
6aii	Health care system level	Ehn, it should be use as a public public, it go under public ehmm..<br>It depends on the qualities does it need cold chain system? Eh eh so the temperature of the this thing must and then maybe giving date they use to announce it most of the things in the churches, do you understand? during service
	No I'm talking of the health facility now	Okay okay okay<br>If ther's cold chain system like I said it should be there and there must be safety supply of energy. Solar is more stable
	Ok	At facility level all workers must know about it.
6aiii	Provider level	Through workshops they will know because tis health related they will know
6b	Are there specific strategies for education or awareness that might be most effective?	Yeah, the most effective will be when inviting them together and bring it as a topic.
	As in no, with regards to the even the community which are there any there specific strategies for education and `awareness that might be most effective?	Direct direct discussion I don't know how to. What do you mean by do you mean the instrument of what?<br>Ward meetings meetings when you meet, when the workers have some time to meet en en<br>They may have skills now. There will be skills for demonstration come and see that thing come and see that thing what we are telling you<br>If there are past history to be demonstrated so that they will know that it is not a joke. Flyers can be and en if you go to the….
7	Can you tell me about your experience with the maternal tetanus vaccination?	Yes<br>What do you want me to talk about?
	Your experience as a clinician	En.. that maternal I think is still the nurses take care of that we give at least two times or three separated at the you know the maternity just when they come for ante natal but some don't take up to 3 times so depends in the day they booked. Some people use to be at in labour extreme cases o some people come at six months and so far they don't complete it
7a	What barriers or challenges have you experienced related to the maternal tetanus vaccination?	Not at all because they are aware of it the nurses are always talking to them educating them. They are aware of it, the awareness is a major issue and some people don't even

		know when they should come for ante natal. They come at any time.
7b	What successes have you experienced, what is working?	We have not have a case of maternal erm erm tetanus do you understand for the child for neonatal nor maternal<br><br>There may be passive dis immunity you know there is something called passive immunity
	Yes that is what I'm asking now, what is actually working? why have you not recorded that	It is because of this antenatal care and beside as well when born there is a prodromal period even if they get it but is a slow something is not when you give to a child he just starts so mostly is from the maternal the child has to develop immunity now but I had several case not here o but in the North where we have neonatal tetanus which is very very dangerous 8 days 8 day old child this and that and that and that and I know they don't take but is with those people nomadic Fulani
	Okay yea.. they don't take the TT	That's what I'm saying infact infact when they come to the hospital there's a way they behave you know their culture affects whatever they are doing
8	What types of health interventions have been the most successful in your community and why have they been so successful?	As regards?<br>All vaccinations<br>Children vaccination<br>With my own experience en?<br>All vaccination …<br>Its vaccination now
	Okay so why has it been so successful	It depends on erm they have now known because at the beginning there are several programs for that they have now known that it prevents so many sicknesses especially childhood sicknesses like tetanus or infact measles it was in this part of the world measles and pertussis, diphtheria all those cases then and ermm infact they do no more get polio is a miracle because bin those day they use to say that these people are not human beings they have come to disturb them<br>So when they see this type of thing they don't you see they cant whatever you tell them and they are seeing examples en en.. those who fail to do it use to get some problems they start reacting<br>So vaccination is the all vaccination is very very important.<br>You see there was a time no childhood diseases we don't get them so that is that

9	Is there anything else you think we should know about hepatitis B, sharing information about hepatitis B, or the hepatitis B birth dose?	You know the Government is they are trying their best they don't pay for such things do you understand? If they don't pay for such thing you know free drugs and infact there may be explanation of consequences if they do not and eventually when they I've said it before that when they hear about cancer they don't want to hear about it to prevent it in future they will rush and it depends on the level of awareness the these young mothers now most of them are taking very high and they understand things faster education may have been the problem before but whenever they hear something about their child they are ready to do the thing.<br>If the government now jingle jingle as they did in HIV cases paaannn over the this thing.<br>Some people cannot read but they can hear so over the radio just mention it.<br>I don't think all maternity has storage system so the centre must be mentioned especially in township because it is those people in township that will go home and talk. If you go to the village they may not understand and they might not have that vaccine. So. Is it the infact dose you're saying?
	Yes	First dose<br>Flyers and they should be within the hospital premises because they do not know when a pregnant woman will come in and go into labour and deliver you don't know they can come any moment if it is not there<br>Also there's time limit as you said but why that time?
	Is because that is the only you know a child when it is given at birth the immunity it grows it tends to because the child does not have maternal immunity the next thing is vaccinate again because the child can be exposed within that same period	But suppose a child is not exposed<br>Is it attenuated or live vaccine? Wait o I want to know the type of vaccine
	It is attenuated	Okay now why the time? Because that you say within 28 hours
	24 hours	24 hours and after that supposing the virus is not there to infect the person the time is limiting
	The time is limiting but it is important that the child gets it because the child can be exposed within that same period	I don't understand<br>In timing is really 24 hours

TRANSCRIPTION OF MIDWIFE KII

FACILITY: State 2, LGA 1, Facility 10

S/N	MODERATOR	RESPONDENT
1.	What are the biggest challenges for people living with hepatitis B in your community?	
	You know Hepatitis B?	Yes, is a virus
	What are the biggest challenges for people living with hepatitis B in your community?	Yea.. challenges are erm…poor access to errm…utilization of health facility due to maybe because of drugs.
	Is it the drugs or the vaccine?	The Vac…. No where…
	Do they pay for vaccine?	No, they don't pay for vaccine but some challenges is that some are not even aware that there is errm.. vaccine for Hepatitis B
2.	What are the biggest needs to address hepatitis B in your community?	Adequate provision of the vaccine
3a.	Is there a strong need for the hepatitis B birth dose? (Why or why not?)	Yes
	Do you need Hepatitis B birth dose?	We do, but is during our immunization
	Is there a strong need for it? For the Hepatitis B birth dose?	Yes, is need…there is need for giving the vaccine
	Why?	To protect the baby from getting the virus
3b	Do others see, do others around, do they see a need for the hepatitis B birth dose? Do they see a need?	Yes
	Aside you, do you think others see a need for that Hepatitis B?	Yes now, everybody. The people working with us, they're aware that birth dose is good for the baby
4	Can you tell me about the current programs or practices for the hepatitis B birth dose in your clinic?	We do routine immunization , now they are planning for IPDS
	Do you…is it part of errmm Hepatitis B, is it part of the birth dose… is it part of the errrmm IPDS?	No, is the routine errrmm..
	No, I'm talking of the current program/practice for that ermm...the birth dose itself	Yes
	Not ermmm… Penta or nPv	For Hepatitis B?
	Yes	Because if you see a new born during the campaign, is being given to the child
	So, there is no current program/practice with regard to birth dose? Hepatitis B birth dose?	No, is only the routines that we are doing

	Only the routines, Okay	Yes
4a	Is the hepatitis B birth dose easy to get within 24 hours of birth? (Why or why not?)	Yes, is within the facility only that the health workers, we will wait till the immunization day
	Why?	So that we will not open the vaccine for only one child
	Okay	
4b	Who can administer the hepatitis B birth dose?	Pardon?
	Who can administer that hepatitis B birth dose?	Health workers
	Which type of health workers? Anyone?	Nurses, Midwives, CHEWS, JCHEWS. They administer it
	Okay	Yea
4c	Now what are the challenges/barriers to getting the Hep B birth dose vaccine? What are the challenges that youv've seen, that is making people not to get the Hep B birth dose vaccine?	Sometime they…they…<br>We don't have challenges because we have vaccine, the vaccine is available. You understand, we have the vaccine, but sometime the health worker that is in charge may not be living in the….within the premises. So because some people you conduct delivery at night
	Okay…	Then if we conducted delivery at night, during the following day we now give to the baby, not that the same night. But what you're talking of is immediately after the birth you give to the person.<br>So we normally do it maybe the following day
	The following day…	Yes..
	So the challenge now is having, is it ermmm about having access to the vaccine or	All the work…because all the health worker, they don't have access to where the vaccine is.
	Eh eh…okay all the health workers do not access to where the vaccine is…	Yes…
	Okay…	
5	Have you gotten information from participants regarding their experiences with the hepatitis B birth dose?<br>Like errm.. the pregnant women or the… have you gotten any information from participants regarding their experiences with the hepatitis B birth dose?	Information on what? Is it the side effect or what? Is it the.. how the Hep B is protecting the
	The importance, the whatever … do you have	Erhn.…they're aware of the importance because will come for the immunization. They know that Hep. B vaccine
	Not…the birth dose	Okay, the birth dose
	Not the..all we're asking is all about the birth dose	If there are?

	If they're aware.. if they've gotten any information in regards to that.. maybe people, even in your facility people that went for training, or they have anything, they have any… have you been informed? Do you have any information with regards to that.	Nooo, we don't have any information. But during training o, they use to tell us that we should at birth. Ehn ehn.. thay use to, we are aware that the normal thing is at birth
5a	Okay, so what is the community understanding of the hepatitis B birth dose?	I don't know their understanding only that if they deliver, they will just come and ermmm that they are for immunization
	Do they ask? Maybe a mother, a premium mother… ensuring that	Okay…few ask of the ermmm… Hep B at birth
	Okay…	Few of them that.. people that are learned. They'll ask that they supposed to take this thing at birth
5b	So are people interested in getting the hepatitis B birth dose? Are they interested in getting the birth dose?	They are interested<br>Yes<br>Yes
5c	Now can you describe the kind of specific information/messaging you have heard with regards to Hepatitis B birth dose? The information you've heard either in training, can you describe what you've actually heard?	Yes, because they will normally, they will inform us too whether is only one person that we must open the Hep B and give to the the baby, they have been telling us about it during training. Any training, they continue to inform us that we should give at birth whether one or two we should not wait till many of them comes together
6.	Okay… Do you have any ideas or suggestions for improving hepatitis B birth dose vaccination rate?	Yes
	So, what is your suggestion?	Reducing the dosage for each errmm.. child<br>Yes. For each vial. Instead of 10 dosage, you can make it one, two, it will be easier. If you just deliver, just go and bring that one dose and give to that child. They will not be missing it
	Okay.. how do you think we can improve it at the community level	Creating of awareness now. You create awareness in doing health talk
6ai	At the community level?	Yes
6aii	What of at the health care system level, that is the…	The same method now, they'll create awareness and..
	No we mean at the health care system level now that is the Primary Health care as a whole, how do you think it can be improved?	Ehn en..
	As in PHCs, the Primary health care board, how do you think the vaccination rate can be improved?	They know what they will do now

	What do you think? What's your own suggestion?	I suggested that they should reduce the dosage from the vial and they should even make the vaccines available to us to the health facilities
6aiii	Okay… what of for individuals like here, how do you think we can improve it for individuals working in the facility, like individual that deliver, that do the delivery and all that, how do you think they too can…..?	Making the vaccine available to them so that after the delivery, they will now give it to the child. Then how we count for the safety of the vaccine, I don't know about that one.<br>If you allow everybody to have access to the cold chain, I don't know how they account for it, I don't know…so it depends on what you want
	Okay, what do you think like health workers that are taking delivery, how do you think they can…	Is not only one health worker now, you know we are doing shifting
	Ehnn… I know	En en..
	But..	All the health workers having access to the vaccine. Okay! If they can account for the safety, is okay.
6b	Now are there specific strategies for education or awareness that might be most effective? Which strategy do you that that will be most effective in reaching out to people to ensure that they understand the important of errr…	Jang…jingles on the radio, people will be more aware and even during health talk. Doing health talk on that
	Jingles on radio and health talk	Uhn..
7	Can you tell me about your experience with the maternal tetanus vaccination?	They are coming for it, even during the weekend the mother they will come
	For maternal tetanus vaccination?	Yes<br>If you give then date because you warn them that you did not come for the particular day, you're going to start afresh. So at that particular date they will come whether during weekend they will come to collect their tetanus fortified vaccine
7a	So What barriers or challenges have you experienced related to the maternal tetanus vaccination? What challenges have you experienced?	I've not
	You've not experience…?	Because the vaccine is always available and..
	You've not experience any barrier or challenges?	When they are around we use to give it to them
7b	What successes have you experienced, hun.. with regard to maternal tetanus vaccination? What Successes have you experienced?	Reduce in neonatal tetanus, yes. We have not been having such case because of the proper vaccination.

	So, what is actually working?	The vaccine is working for the mothers because they're coming for the….
	No, in ensuring that they are not missing it, what do you think is making them to…	Because we are giving them health talk seriously on that, that they should not miss it. If you miss it you are going to start afresh for that they are coming because the vaccine is working, we have not been having this neonatal tetanus
8	What types of health interventions have been the most successful in your community? Do you understand what I'm saying?<br>I said what types of health interventions have been most successful in this your community, your catchment like…	Intervention like health talk now, because the vaccine is free, they will always want…
	No, I'm talking of health intervention, not vaccine now. Which type of health intervention have been very most successful in your community? Health intervention!	Like?
	Health intervention that they usually, you people carry out this facility that has been most successful	Okay…<br>Like during flag off, if you invite them on flag off
	Flag off for what?	If we are having maybe campaign they will come
	Campaign on? Which of the campaign?	Maybe exclusive breastfeeding..
	Which one is the most successful in your community? Which one do you think is the most successful among all the health interventions?	……
	Is the most successful one you know?	Yes
	You said distribution of, which one?	Mosquito net, mosquito treated net. That's malaria intervention
	Okay…That's is the most successful one?	And if you're telling them to come and collect malaria drug free! They will come
	Okay… that's the type of intervention that has been so successful	
	So, why has it been so successful?	Because is the most errmmm.. Malaria affects almost everybody.<br>En en.. so, if you hear about ernn malaria and mosquito net for you to protect yourself , they will come, all of them they will like to collect the net and collect the drugs
9	Is there anything else you think we should know about hepatitis B, sharing information about hepatitis B, or the hepatitis B birth dose?	Ehnn.. you were talking of Hepatitis B but this ermm.. Hepatitis C. we are seeing such cases but we don't have the vaccine.

	How do you think we can share information about this Hepatitis B or the Hepatitis B birth dose?	How we can share the information?
	Ehn.. sharing of information about it	For every health workers to engage in health talk. When they are doing the health talk they should include the birth dose of Hepatitis B it will help.
	But they too should know about it, the Hepatitis B birth dose	Yes now<br>They are aware about the Hepatitis B<br>They know that they supposed to give Hepatitis B birth dose, ehn ehn..<br>They are aware of that but it's not being practiced.
	Why?	I told you now that not all the health worker that conduct delivery that have access to the vaccine

TRANSCRIPTION OF PREGNANT WOMAN KII

FACILITY: State 2, LGA 1, Facility 10

Age: 31yrs

S/N	MODERATOR	RESPONDENT
1	Kedu ihe maara gbasara oria imeju a n'akpo iba ocha n'anya	Ihe m mara maka ya bu , dika mmadu n'enwe obere ahu oku, oburu n'ogworo ya, n'onuro paracetamol, onwero ka osi nedo onwe ya anya, o ba ya ahu ofuma, ma obu oke oyi itu mmadu, onwere ka oga anote aka , o mezie onye ahu iba ocha n'anya
2	Kedu ihe maara gbasara ogwu mgbochi iba ocha n'anya	Onweghi ihe m maara
2a	Olee otu obi di gi maka ogwu mgbochi iba ocha n'anya	
	Iba ocha, ka m koro gi ihe iba ocha n'anya bu, iba ocha n'anya bu iba n'eme mmadu, o bu oria nne nwere ike ibunye nwa ya, o na eme na liver, I ma ihe bu liver, iba ahu na ebido na liver, o bu obido na liver, liver gi ebue ibu, obido mewe gi, o bata gi na anya, anya gi a na acha yellow, o na egbu ndi mmadu, mana a na agba ogwu mgbochi, I diri ime, I muo nwa gi ubochi I muru nwa ka a na agba ogwu mgbochi ahu, I ma na otutu ndi mmadu a maro na a n'agba ogwu mgbochi ahu. Dika otu I si kwuo n'imaro na an'agba ogwu mgbochi. A n'agba ubochi ahu I muru nwa ka an'agba ya, o buro na aga asi gi bia echi ma obu nwanechi, ubochi ahu a muru nwa ka an'agba ya.<br>So, olee otuobi di gi maka ogwu mgbochi iba ochan'anya?	Obi dim nma ka agba ya, maka na agba ya, iba ocha n'anya ahu ama putazi
2b	O nwetula mgbe I rioro ka agbaa umu gi ndi ozo ogwu iba ocha n'anya?	O nwebeghi
3	I nwere atumatu o bula iji nweta ogwu mgbochi iba ocha n'anya maka nwa gi	Atumatu m nwere bu maka igba nwa m ogwu mgbochi bu ka enwee ike gba nwa m ogwu mgbochi
3a	Gini mere ma o bu gini kpatara ya	Atumatu m nwere bu na agba nwa m ogwu mgbuchi ma oburu na mu amuo nwa bu na oburu na onwere oria o bula dim n'ahu dika nne ya , ka oghara ife nwa ahu , ka oghara isi otua me nwa nkocha no , na agba ya ogwu mgbochi ahu na oga akwusi
3b	I nwere mmetuta o bula gbasara nchegbu, nrugide, obi uto,	Eeh, maka na nwa m ama nwezi oria iba ocha n'anya ahu, oma di ya n'ahu
4	I chere na I gba ogwu mgbochi oria iba ocha n'anya ga-aru oru ma o buru na	Eeh, echere na oga aru oru

	agba ya nwa a muru ohuru?	
	Gini mere I ji chee otu ahu	Ihe m chere bu na ubochi ahu amuru nwa ahu ka oga ebido nawa ikuku uwa, ya bu na agba ya ogwu ahu, na oga eme ya, oburu na onwere nke di ya na ahu sitere na ahu munwa bu nne ya, o ma putazi ihe, o ma mee ya
5	I chere na I ga enweta ogwu mgbochi oria iba ocha n'anya B n'uzo di mfe ma o buru na i choo ya	Eeh , o ga adi mfe, ma m bia n'ulo ogwu a
5a	I ga ario maka ya	E chere m na oga adi mfe,na ha ga agba ya nwa m ma mu muo ya
5b	Gini mere I ji kwuo otu a	Maka n'ulo ogwu ebea, na ana elekota ndi di ime na ndi na amu nwa anya
5c	Onwere ihe obula nke puru ibia n'uzo ma obu nsogbu ndi ozo nwere ike igbochi gi I nweta ogwu mgbochi iba ocha n'anya nke an'agba nwa a muru ohuru?	O nweghi ihe m chere na oga egbochi, na oge nke mu onwem, o buru na onwereihe ga egbochi, na mu amaro
	Onweghi ihe ichere na oga egbochi gi igba nwa gi ogwu mgbochi iba ocha n'anya	Mba, O nwere ike oburu na m muo nwa, o nwere o buru na ebutebeghi ya , na aga ebute ya ebute tupu, anyi amana ata ndi na agba ogwu uta, onwere ike o buru na odiro oge m ga amu nwa
6	O di gi ka igba nwa go ogwu mgbochi iba ocha n'anya B di mkpa?	Eeh (yes)
	Gini kpatara I ji kwuo otua	I di mkpa ka o wee gbochie ya, ka iba ocha n'anya ghara ime ya, ma agba ya ogwu mgbochi ahu
7	Na mkpokota, I chere na umu nwanyi di ime no na mpaghara gi ga -enweta ogwu mgbochi iba ocha n'anya B maka umu ha?	
	Kwuo ihe bu uche gi, I chere na ha ga eneweta	Onye o bula ga enweta ma ha bia n'ulo ogwu ebea, ebe anyi na amu nwa
	Ndi n'abiaro, kedu maka ndi n'abiaro	E cherokwam na ha ga enweta ya na ebe ozo, n'aburo ebea
	I ma n'onwere n'amu nwa na aburo n'ulo ogwu, ha n'ano n'aka ndi n'amu na midwife, I chere na ha ga enweta ya ebe ahu	E cheghim na ha ga enweta ya ebe ahu
7a	Kedu ihe na-enye gi obi ike ahu (ma o bu enweghi ntukwasi obi)	Anam enwe ntukwatusi obi , Ihe n'enyem obi uto bu na ndi noosu, ha n'akowara anyi otutu ihe, k'anyi ga n'esi enete onwe anya, dika ndi di ime, ha n'enye anyi ogwu, nke anyi n'anu maka nwa anyi bu n'afo, ha na enene anyi ofuma , ka ha mara etu nwa anyi si noro na afo. Ha na akowara anyi otutu ihe nke anyi kwesiri idi n' eme na nke anyi n'ekwesighi idi n'eme. O bu ya na enye m obi uto idi na abia ebea.
8	Inwere aro o bula I enye anyi ma o buru na anyi choro ikuziri ndi mmadu n' obodo	Aro nwere ike enye unu ikuziri ndi ozo bu, onye obula gba mbo na aga na ulo ogwu gooment, nwanyi obula

	gi gbasara ogwu mgochi iba ocha n' anya (Site na akuko, vidiyo, akwukwo okwa, uzo ndi ozo)?	di ime, ya na eje ebe ahu ka ana akuzikwara ya na akowakwara ya, ka omuo nwa ya, ka enwere wee ike igba ya ogwu a, ka ohara ibu emechea ya ekuru nwa ya na agbahari.
8a	O ga amasi gi imu gbasara iba ocha n'anya(Site na akuko, vidiyo, akwukwo okwa,uzo ndi ozo)?	O ga amasi m, site na vidiyo ya, oburu na a ga enye m vidiyo ya.
8b	Kedu otu kachasi amasi gi imu gbasara nsogbu ahuike?	Otu kachasi amasi m imu gbasara nsogbu ahu ike bu, i kowara m ya n'onu, ka m ghota ya. Na ulo ogwu, mbia n'ulo ogwu, otua m biara antenatal akowara m ya.
9	O nwere ihe ozo I chere na anyi kwesiri ima gbasara iba ocha n'anya, ikekorita ozi gbasara iba ocha n' anya, ma o bu usoro igba nwa a muru ohuru ogwu mgbochi iba ocha n'anya.	Onwero aro ozo m ma n' aburo idi na akowa ya ka ndi mmadu n' aghota na odi mkpa ka ha na aga na ulo ogwu goomenti, ndi di ime,ka ha n' aga ebe ahu, na anu nkowa, muta kwa ihe, site n'ebe ahu ka ha ga aghota n' igba ogwu mgbochi ocha n' anya di mkpa ria nne.<br>Otu ozo m chere bu, oburu di ka enye ya ozo na news dika redio ndi n' anubeghi maka ya, maka n'onwere ndi na amaghi na an'aga n' ulo ogwu goomenti, ihe ha ma, bu na ha di ime, ha ga anoro ebe ahu, noosu obula no ha nso ha n'eje, ya bu na enye ya na news , dika rediyo, otutu ndi mmadu n' ege rediyo ndi adi ege rediyo di pere mpe, ya bu na ha nwere ike inuta ya ebe ahu ,onye nuta ya ebe ahu, ogwa onye di ime, si ya nekwe ihe ya nuru, site out ahu onye n'amoro o mara.

Note taking template for Pregnant women KII guide State 2, LGA 2, Facility 1

Demographic information of interviewee

Age:28

Questions

1. What do you know about hepatitis B?
   The way that I understand my own, you know that because I'm not speaking igbo, like when they teach us in the class, like the antenatal day, I do ask people, so my own view is that when they say that that that ehhhh…. *sighs* (Coordinator: Hepatitis B) B is that, is about your health, as in how you take care of yourself for you not to have the disease, you have to keep yourself safe and neat and environment, that's what I know about it (Coordinator: about Hepatitis B) yes

2. What do you know about the hepatitis B birth dose?

   No

   (Coordinator: you've never heard about Hepatitis B Birth dose vaccine)
   I have heard it but I couldn't remember it again
   (Coordinator: okay, good. Now, the pregnancy is this the first one?)
   No, the second one
   (Coordinator: So, when you had the previous pregnancy, did you hear anything about when you come for immunization you gave birth to that child, did you hear anything about Hepatitis B birth dose vaccine)
   Yes
   (Coordinator: What did you hear about it)
   Uhmmmmm, what I know about that is about the health of the baby and the mothers, that's what I know about it.

2a. How do you feel about the hepatitis B birth dose?

Pauses (Coordinator: like when you gave birth to that your first child, you said you heard about the vaccine and what it means is for the health of the child and the mother) Yes (Coordinator: Did you vaccinate that your child) Of course (Coordinator: which health facility did you vaccinate the child, was it here?) No, in Port Harcourt, it's still health centre. I attend my antenatal here, then I went to Port Harcourt the same health centre to put to bed in Port Harcourt. (Coordinator: Okay, what do you feel about the hepatitis B birth dose?) It's okay because when you do that, the child will look healthy you'll not have any communication(complication) of your baby again.

2b. Have you ever asked for the hepatitis B birth dose before for other children?

What I notice, I just told the nurse, the midwife that she should attend to me that the way that my baby will be okay, that will not have issue on my baby, that anything that you know that is a, as you are a mother before me that you can take care of my child, as in I give everything to you, is only God that can control and I want you to take care of this my baby for me, that the one that you know that is the best, do it and he say Yes.

3.Do you plan to get the hepatitis B birth dose for your baby?

pauses (Coordinator: what I mean, will you allow the health workers to vaccinate your child, this baby with hepatitis B vaccine when the child is born) Of course

3a. Why or why not?

Because I want my baby should as I said it earlier because I want my baby should look healthy and be okay. How am I going to worry when the doctors and nurses attend to me in the way that I want it and my baby will be okay, I'll not have any worry, the only thing I'll have a joy.

3b. Do you have any feelings of concern, stress, excitement? Why?

Yes. It's just be like when you want, when you know that you are feeling headache already, as in the headache was just coming and you need something that can kill that it immediately, you can rush to that place immediately for it not to get the disease. That's my own understanding.

4. Do you think the hepatitis B birth dose will work to prevent hepatitis B? (Why or why not?)

Yes.

5. Do you think you could easily get the hepatitis B birth dose vaccine if you asked for it?

If its available in the Hospital, if not available, they will tell that its not available to them, can come another day or they'll directed me to another health centre which I can get it. I can get it in health centre, in any health centre.

5a. Will you ask for it?

I told you that what I did normally, when I come to hospital when I put to bed, I'll tell the nurse should take control, that whatever that you know that is good for my baby – do it.

5b. Why do you say that?

Because, I am the kind of person that things bothering me quick, when I put something in my mind, I will not sleep over it. So, when I want to do something, I know that I want to do it let that one clear on my side, I don't want issue that I'll be running up and down, while maybe the nurse was told me that do this ma that I say No that I don't want, them know more than me. That's why I always give them go ahead, do whatever that you know that it will be good for my baby, I don't doubt doctor or nurse.

5c. Is there anything that might come in the way or challenges you might face related to accessing the birth dose?

Eh maybe the nurse told me that I cannot give my baby, which is I cannot doubt. Anything they say, I'll do it, I'll not doubt them.

6. Do you feel the hepatitis B birth dose is important for you to get for your child? (Why or why not?)

Yes of course. Cos its necessary for my baby.

7. In general, do you think pregnant women in your region get the hepatitis B birth dose for their babies?

Yes, because is only if its available, the nurse will ask them to take it. That's my own belief.

7a. What gives you that level of confidence (or lack of confidence)?

I'm a mother, they too they're a mother, we cannot feel the same thing. Everybody is a different people that they can feel the way they feel, but I feel that anything that is good, I think that another person can support it because what I notice is that if something is not good, they'll not introduce us to us to that it.

8. Do you have any suggestions for us if we want to educate people in your community about the hepatitis B birth dose?

Yes, of course, because you know we human beings we're all different, when you want to introduce something to somebody, you have to explain it to your own level of understanding because they may scared like how am I going to take this, that I am as in when I put to bed the first one, the second one, the third one, I did not give my baby, why are you saying that I should give my baby in this fourth baby now. There's a way that ah madam, you know then, by then we upgrade in our system, many things happening. There's a way that you can encourage them, they can be in the party to. But there are some people they are very stubborn, when you tell them they should do this, they'll never do that. Is what they will outsider. Me whatever they do to me I do come to the hospital and confirm before, can I take this, can I do this, they'll say okay go ahead this one is good for you, this one is not good for you. I don't doubt with doctors or nurse.

8a. Would you prefer to learn about hepatitis B? (Through stories, videos, flyers, other methods)

Okay, like the way that I think I have known you today, I have seen you, I have seen your face, that if I have any, that I want to know more about it, I can come to my doctor or go to my nurse to ask much depth and maybe like facebook part, all those things that I can be able to ask. If it's a real facebook account that I can able to call, if there's any number or anywhere that I can login and check a website, I can do that.

8b. How do you prefer to learn about health issues?

Uhmm, you know, you know there's a way they, some like, it just be like, some like when you want to go to a work, there's a training they'll say that today we want to teach on some health problems or health issues, they'll say that by so so time they will want to announce from so so time today that maybe next week we want to have a seminar that we can teach about health. Some people that have interest, they'll come, some people will not come. So, because if you didn't come close to such you will not know, but when you come close to such you will know much better and it is very .....that you know what they are doing. Eh that's why I say that, like a seminar or training? Is it not the same? Okay seminar or training. Like a hall, like you organize like a meeting that you can teach people around you, that people that are ready to learn from you.

9. Is there anything else you think we should know about hepatitis B, sharing information about hepatitis B, or the hepatitis B birth dose?

Ehhhhhhh, nothing much, nothing much but just that, a lot of people doesn't know about it, if there's anyway like maybe like flier or to do like is it promo, how am I going to said it, for them

to know much better. You know some people the way that they live like we don't care, some people doesn't care, so if there's a way that they will know about it much better, we'll try to bring it up, and they will be still teaching us when we come to antenatal at least let people that doesn't know about it let them know much better, so that its very important.

Note taking template for Health care worker KII guide State 2, LGA 2, Facility 1

Demographic information of interviewee

Age: 50 Years

Sex: Female

Designation: Routine Immunization Focal Person

Questions

1. What are the biggest challenges for people living with hepatitis B in your community?

I think the biggest challenges is how to live with the hepatitis b itself

They need health education, they need support, they need their drug too ehe, that is the challenge they have

2. What are the biggest needs to address hepatitis B in your community?

The biggest need is vaccination, immunization, we need to vaccinate them

3a. Is there a strong need for the hepatitis B birth dose? (Why or why not?)

Yes, there is a strong need

Eh because eh at birth, the children are tender, we suppose to immunize them, and some of them, they may even be exposed child. The mother may even have it and you will not know that the mother is hepatitis positive so is very important we immunize them at birth dose

3b. Do others see a need for the hepatitis B birth dose?

They may see it, I may not speak for them nah. Yes, when we give them health education on the importance of the immunization, they may see the need for it.

4.Can you tell me about the current programs or practices for the hepatitis B birth dose in your clinic?

In my clinic, we do give them hepatitis b vaccine at birth, that is within 24 hours. But if the other people that are coming because those that we delivered here we make sure we give but others that come from outside we do give them up to two weeks, the birth dose.

4a. Is the hepatitis B birth dose easy to get within 24 hours of birth? (Why or why not?)

Yes,

Here in our Health Facility, it is easy to get because we do have it in our solar all the time and apart from that, the cold room for the LGA is within this facility so we can easily get it and give.

4b. Who can administer the hepatitis B birth dose?

I think almost every trained person here can administer it.

Probed for designations: the RI focal person used to give, our officer-in-charge used to give, the midwife used to give, then any trained health worker that is around after the delivery of a baby give the hepatitis b vaccine, any trained person around use to give.

4c. What are challenges/barriers to getting the hep B birth dose vaccine?

Well, the challenges, at times if you run out of stock, the cold chain people may tell us that they don't have that we should wait if they go to State they will bring so at times it used to be like that so whenever they bring it, we give.

Probed for more challenges:

For now, I don't know any other one o.

The period stock-out is occasionally, it doesn't last.

5. Have you gotten information from participants regarding their experiences with the hepatitis B birth dose?

No

My experience is that some mothers may not know the need of taking the vaccine at birth, that is, those that come from outside, they may not know the need but those that deliver here, we do explain to them and give the vaccine but some that come from outside, some people may even stay up to three weeks before coming for BCG, so they don't know the need and we have to health educate them. Some of them don't know the need at birth.

5a. What is the community understanding of the hepatitis B birth dose?

You know all these things depend on awareness, everything depends on awareness. If they are not aware of it, they may not know anything about it, but if they are aware of it then, we can now talk about their understanding.

Their understanding is that they know that it is to prevent the disease which is hepatitis b. They know that the vaccine is to prevent it, I can say that they know that.

5b. Are people interested in getting the hepatitis B birth dose?

Yes, they do come

5c. Can you describe what kind of specific information/messaging you have heard?

I don’t know

Probed:

No

6. Do you have any ideas or suggestions for improving hepatitis B birth dose vaccination rates?

Uhmm, yes, what I will suggest is that hepatitis b vaccine should be available in all the health facilities, that is, even in the labour room so that as the child is being delivered, you give the the hepatitis b, in that way they will not be missing the birth dose, even the private hospitals, we supposed to have it available.

6ai. At the community level

Yes, at community level, we need awareness, we need awareness, that is, health educate them and tell them the importance of the vaccine.

6aii. Health care system level

At the ministry, at the agency, what I will suggest is that number 1, there is no staff o, we don’t have staff doing……, all these people you are seeing there, they are volunteers and students. We don’t have staff, we need staff that will be carrying out all these activities. There is no staff at all, as big as this place is, we have only, we are not up to 7 trained staff here, about 6, others are volunteers. So we need staff that will be carrying out all these activities. That is the major problem that we are having.

6aiii. Provider level

Eh we need eh continuous training

6b. Are there specific strategies for education or awareness that might be most effective?

Yes, you can go into the community, do it as eh announcement, we have town criers, we have eh town announcers with eh this eh megaphone, you can tell them too through that medium.

Probed for more:

I don’t know oo.

7. Can you tell me about your experience with the maternal tetanus vaccination?

My experience about that one is that eh, that maternal tetanus dose, in this community, the awareness, that I the live one, that 5 doses, the awareness in this area is very low, is very very low. So we need sensitization and eh, eh community awareness. What they are used to in this area although we use to give them health education about the TT live eh this thing, but what they are used to generally here is once they are pregnant, they take TT1, TT2, that's all. That's what they are used to. The awareness of TT1, TT2, TT3, TT4, TT5 is very very low in this community.

7a. What barriers or challenges have you experienced related to the maternal tetanus vaccination?

No, except this one I told you now that they are not aware of taking it 5 times.

7b. What successes have you experienced, what is working?

Eh, I can say that I myself, since I have been here, I have never witnessed any neonatal eh tetanus eh this thing, I have never witnessed any mother that delivered having tetanus, that's number one, then the vaccines are always available, so I think there is reduction in people that is having the……,I have never witnessed any so that is number one success which means the vaccine is… they are working.

8. What types of health interventions have been the most successful in your community and why have they been so successful?

What are the health interventions? Like eh immunization, antenatal and eh family planning, the most successful. Many of them are successful, I don't know the one I will say is the most successful now, (laughter). Okay let me start from my own point, immunization, yes because all the vaccines are readily available, once they come here they will be immunized and go, so I think it is a success. Ehe! because all these eh child killer diseases is reducing, there is growth reduction in IT, most of them I have never witnessed, I used to hear o with ear. So there are success.

9. Is there anything else you think we should know about hepatitis B, sharing information about hepatitis B, or the hepatitis B birth dose?

No

State 2, LGA 2, Facility 2

Moderator: my name is [name], my colleagues name is [name] we are from AFENET(African Field Epidemiology Network), we are in this health facility [facility name] in [LGA name] to conduct Hepatitis B birth dose assessment and to conduct a Key informant interview for the Officer in Charge of the health facility now we want to start with the KII with you the officer in charge [facility name] in [LGA name] this interview will last for about 30-45 minutes and the questions we ask you are for you to respond in ways that you understand they are not exams questions so feel free anyone you don't understand, may I know so that we could possibly ask it in a way you could understand thank you we don't want you to mention your name while we are discussing, just tell us whatever you feel about the question we are going to ask you. Do you permit us to start the interview?

OIC: Go ahead

Moderator: thank you

OIC: (coughs)

Moderator: Sorry please so may we know your age ma,

OIC: it's forty five

Moderator: what is your designation here

OIC:OIC

Moderator: you are the officer in charge of [facility name] in [LGA name]

OIC: yes

Moderator: thank you ma

OIC: (coughs)

Moderator: sorry ma

Moderator: the first question we want to ask is, What are the biggest challenges for people living with hepatitis B in your community?

OIC: the challenges ?

Moderator: of people living with Hepatitis B

OIC: (coughs) the challenges are that emmm, there is no challenge because we are giving enough vaccines to protect children from death so as a result of that we don't have any challenges

Moderator: okay now what you say is okay but what do you think are the possible challenges of people who already have Hepatitis B face in the community

OIC: (coughs) whenever I eat groundnut it scratches my throat

Moderator: sorry, so people that already have this disease. You know about Hepatitis B

OIC: yes, yes

Moderator: so what are the possible challenges you feel they can have?

OIC: one of the challenges is emmmm when they have facility like this, then it will be good for the facility to have a competent hands then when we get such people either through lab or through appearance then the challenges is that how we refer the people to a higher level for management.

Moderator: so how do they appear you talked about eh that it can either be by during test or

OIC: like pregnant mothers through pregnant mothers you can get it through pregnant mothers

Moderators: you say you can identify them through the way they look, is their anyhow they look that you feel maybe a challenge to them

OIC: the challenge to them is that emm emm sme of them may not have the money to emmm treat themselves, they may not have the money to treat themselves and mmmm even if you tell them to go outside they will be no transportation(clears throat) and availability of the drugs will be there in case if they are referred back when they are referred back for us to continue with what they did, we may not have emmmm drugs for any other thing like that

Moderator: okay, that's okay, that's okay. What are the biggest needs to address hepatitis B in your community?

OIC: what is what…?

Moderator: what are the biggest needs, the needs to address hepatitis B in your community

OIC: emmmm its emmmm I think mobilization, we mobilize people and tell them what is, (clears throat) what is existing so when those people, when you mobilize them they can even have the access because some of them you don't mobilize them they cannot know that heath facility can identify their problem, so mobilization is mostly dead and health workers to mobilize

Moderator: Is there a strong need for the hepatitis B birth dose vaccination, do you think there is a strong need

OIC: Yes,

Moderator: Why?

OIC: because if you check them during birth as we were told during training emmm that emmm from day 1 from birth to fourteen days we give hepatitis B vaccine zero dose so when you get the there at six weeks, ten weeks and fourteen weeks you get them, I don't think they will have it in future time.

Moderator: Do others see a need for the hepatitis B birth dose? Do you think other people see a need for hepatitis B birth dose vaccination

OIC: Yes because of the health education that is going around so people now started knowing that while people that are (ehh) have it, maybe adults or those people that doesn't have the immunization at birth that is have complete dose of immunization some of them have it and when because of mobilization we tell that why these people have is because they were not covered so those people that are covered do not have challenges, those people that will have challenges are those people that did not cover hepatitis B either during birth maybe they are ignorance of that health education help to eliminate those things.

Moderator: Can you tell me about the current programs or practices for the hepatitis B birth dose in your clinic?

OIC: what?

Moderator: Can you tell me about the current programs or practices the things you do, for the hepatitis B birth dose vaccine in your health facility.

OIC: as I said earlier immediately a child is born we now cover that person, that child with hep B Zero.

Moderator: that is what you do in this health facility

OIC: off course and even those people that born somewhere because of the mobilization even they born anywhere or TBA some like that because they know that we are giving vaccines here, the health education is going round they do bring before 2 weeks of birth.

Moderator: Is the hepatitis B birth dose easy to get within 24 hours of birth in your health facility?

OIC: of course

Moderator: why

OIC: we have solar

Moderator: you have solar? okay

OIC we have solar so it's always available here so immediately a child is born you now give it

Moderator: Who can administer the hepatitis B birth dose in this your facility? Who and who can administer it?

Answer: the OIC is there, the focal person RI CHEW is there she is the one that is on training now.

Moderator: What are challenges/barriers to getting the hep B birth dose vaccine? Are there barriers or challenges in getting it in this your health facility?

OIC: the only thing is that transportation, its transportation the solar is already there, then, although we don't have light, because of the energy power, the sun, the energy power we don't have any problem about the storage, only the problem we have is to get it and store. Like Abakpa is our Zonal area for collection

Moderator: that is transporting the vaccine?

OIC: transportation is our major problem and sending the data is also, transportation

Moderator: transportation for bringing the vaccines, the antigens and also transportation for sending the data to the LGA

OIC: yes to the LIO

Moderator: can you expand more on this transportation problem, can you tell us more

OIC: on this transportation

Moderator: hmmmm

OIC: hmmmmm

Moderator: why is it a challenge

OIC: it's a challenge because hmmmmmm if you are not paid and you don't have your own money to go and collect it. It becomes a challenge, because no money to transport yourself, because you I don't have the money and government is not giving me the money, like emmm what do I call it overtime or overhead

Moderator: Organize logistics for bringing the vaccines and taking your data

OIC: yes

Moderator: so it's a challenge

OIC: big challenge

Moderator: Have you gotten information from participants, those women who are coming for ANC, who are coming for immunization, have you gotten information from them regarding their experiences with the hepatitis B birth dose? Have you gotten information during the course of

your interaction with them as they come ANC as the come for immunization as they come child birth have you gotten information from them regarding their experience with the hepatitis B birth dose? Are there any information that you have gotten from them, their views, their beliefs, their perceptions, their reactions, their acceptance or rejection anything you want to tell us?

OIC: no they accept it because many of them when you give them health education and tell them instances, some of them will say ewooo, is this what that killed their brother or killed their son or killed of their children they have seen it somewhere and the believe its maybe witches and wizards and they now go for emm native drugs so is it that this common thing that have treatment that killed many people so those people that come accept it but my view is that some of them do tell their experiences elsewhere not here.

Moderator: What is the community understanding of hepatitis B birth dose?

OIC: community what?

Moderator: community understanding this Ugboezeji now, how do they understand this hepatitis B birth dose vaccination, what can you share with us of their understanding of it

OIC: if a child is covered immediately after birth and during the course of routine immunization there will be low death in children and it will not, maybe even if the child grows up and be in the midst of people that have it, the person will not contract it as those people that do not have it.

Moderator: Are people interested in getting the hepatitis B birth dose in your understanding in this Ugboezeji?

OIC: yes

Moderator: you think people are interested ?

OIC: yes much

Moderator: why do you say so?

OIC: because they have seen the good part of it their children.

Moderator: can you describe what kind of specific information you have got concerning Hepatitis B birth dose vaccination what I mean is can you describe for us some specific information that you have got or some messaging ways of maybe communicating you about Hepatitis B birth dose are they some you want to share with us some ways you have got information concerning it?

OIC: not yet

Moderator: Okay I mean all this information that you have been sharing with us the knowledge you have about it can you give us some specific means through which you got this knowledge

OIC: okay from interaction

Moderator: okay interaction with who

OIC: my clients or the community when we go for outreach then from emmmm….

Moderator: I mean the things you know about Hepatitis B also how did you get to know them Hepatitis B birth dose are there some specific means or ways that you were able to get to know about this things that you are telling us because you have shared a lot of knowledge about it. So may we know the ways you got have this knowledge you are sharing.

OIC: through training, its through training, social media

Moderator: okay training social media interaction with client when you go for outreaches when they come to health facilities, that's okay lets move forward to the next question. Have you do you have any ideas or suggestions that you would want to give to us for improving hepatitis B birth dose vaccination rates are there some suggestions you want to give to us on how Hepatitis B birth dose vaccination rates can be improved so that we look at it and see how it will help?

OIC: hmmmmm

Moderator: are there some things you feel when they are done it will help in increasing Hepatitis B birth dose vaccination rate this are the things you want us to do this this this…?

OIC: what we want you people to do is to give us health workers when we have the health workers we will have remote areas to cover so we would go to those hard to reach area and the people will know more about this, so what we need is mostly is health workers to go inside the interior communities and mobilize

Moderator: and mobilize people?

OIC: and mobilize people only two persons cannot do the whole job

Moderator: you are just only two in this health facility?

OIC: we are only two, the OIC and then RI

Moderator: Okay are there any other things you feel we should do?

OIC: transportation

Moderator: okay so what do you want us to do about it?

OIC: please employ workers and give us transport, jingles those people that can make people to create awareness even vehicle is there, keke if you give we would us mic and go round and tell people

Moderator: so at community level, I want you to break them down you have spoken generally, what do you suggest we can do at community level to increase the hepatitis B birth dose vaccination rate?

Moderator: community level at Ugboezeji level

OIC: outreach and mobilization

Moderator: what about at the healthcare system level like ministry of health, agency, at facility level what are the things you feel we can do? What do you feel we should advice, recommend to the ministry of health to the agency, to the health facility, what are those things you think we should recommend that if those things are done, they will help to improve the hepatitis B birth dose vaccination rate

OIC: hmmmm I still talk on manpower

Moderator: okay The ministry of health the agency should employ more people

OIC: tribble yes then and availability of vaccine although they are trying

Moderator: they are trying but they should do more

OIC: yes they should do more, we don't lack expect in future but has of now they are supplying us enough

Moderator: what about at the health care provider level at the routine immunization officer the OIC the other health workers not just restricted to ugboezeji, in ugboezeji and other health workers who are the ones giving the services, what do you suggest can be dine at that level that could help to improve the hepatitis B birth dose vaccination rate?

OIC: still health workers provide health workers because what am suffering now other people are suffering even if you don't go there, we all put hands together and tell ourselves this our problem and that transportation as well we must talk of transportation

Moderator: is there any other thing that can be done at the level if the health care worker that could improve the services of wanting to improve the hepatitis B birth dose vaccination?

OIC: then some of the health facility do not have a suitable place for giving the vaccine like that small place don't accommodate mothers had it been that you come tomorrow you won't have a place to work in unless you then we don't have benches and you can see our table

Moderator: because tomorrow is your immunization days

OIC: we have registers but things like these tables chairs and at times on immunization days I will stand up they will take all my chairs, the seat am sitting and even the one my will be sitting while

I am consulting I will just stand up and the patient will stand up while I give the seats to nursing mothers because they can easily fall down, so benches tables other tools, so they have to expand our place of immunization

Moderator: Are there specific strategies for education or awareness that might be most effective, creating awareness at the community level, at health facility level? Are there some specific strategies for educating them and creating awareness that you feel that might be most effective?

OIC: yes

Moderator; can you share with us please

OIC: posters, posters is there and giving them little little things, cause we believe when mothers come and after educating them, like now I saw this type of thing, face mask you give them they value it so if they can provide something that after being fully immunized, like I could remember 2015 or back when fully immunized you give a mother a net. Thank you for completing the immunization and it also helps to give information to other people after the immunization they will give you net free, government should know something they will be giving pregnant mothers so when they register, this is for you and your newborn baby i.e. the in-utero to sleep, and after when they deliver you give them maybe that one has torn or spoilt, you give them, then after the completion of immunization you give them again in one pregnancy they can get three nets then. At that time the immunization was encouraged and people were coming very well. They need incentives.

Moderator: thank you, Can you tell me about your experience with the maternal tetanus vaccination?

OIC: my experience

Moderator: yes your experience about maternal tetanus now they call it Td

OIC: yes Td

Moderator: when women come for ANC tell us you experience

OIC: all of it?

Moderator: anyone you want to share with us.

OIC: the experience I am having here is that when a mother is registered at 16 weeks we give the first Td after 4weeks we give the second Td and tell the mother and give the mother the date and after 6 months of the 2nd then the mother will come 3rd Td we now tell the mother the new date the forth then we give date the fifth one its now complete Td for the mother and that will help the mother even if the mother goes anywhere where health center is far away or doesn't know

any health center maybe the person packed in that place and the labor started the person can go anywhere and deliver and they person will not get what Tetanus. Then those people that do not have it my experience is that it if they deliver in a dirty environment and even if they deliver in a clean environment, You know that women this thing is always porous so there is chances of that person contracting it., then we will now educate them please its better for you to have the 5 doses in case you don't know where you will get yourselves in the next pregnancy.

Moderator: so do they comply to have the 5 doses in Ugboezeji

OIC: the educated people are complying, some educated people are complying expect they pack out and go another place, we tell them to continue

Moderator: What barriers or challenges have you experienced related to the maternal tetanus vaccination? Are there some challenges or barriers you have experienced in your health facility concerning the maternal tetanus vaccination

OIC: the only thing is that, the challenge is that some of them do say that after the immunization injecting them they feel pains and we tell that person that it shows that it is potent

Moderator: What successes have you experienced, what is working? What makes you joyful about this vaccination we have been discussing about, are there some success you have recorded or experience what is working for you can you share with us

OIC: things that are working for me is that when they give birth there is no problem even the, the baby will be free and the mother will be free till the baby starts 6 weeks so it's giving me joy.

Moderator: What types of health interventions have been the most successful in your community and why have they been so successful? The health interventions the health strategies that have been successful in this community ugboezeji, and why have they been that successful.

OIC: because they follow our instructions

Moderator: I want you to get the question first of all are there some methods or health intervention that have been successful in ugbezeji are you getting it. Are there some I don't know how else to put it, the strategies the health interventions, are there Are some things you have done to improve the health of the community that have been successful, why have they been successful.

OIC: Td or general

Moderator: general are there some things you have done at the level of the health facilities for the community that you feel were successful and why do you think they were successful, like you told us that some health intervention during the course of our discussion so well want you to share with us are their some intervention that you have conducted or you have done in this

community that were successful that why do you think they were successful We want to learn also

OIC: although when I was posted here 2 years ago, here was not moving at all, so what I did was to conduct free treatment for them to know that government hand of a thing in the community and after doing that people started utilizing this place and those they needed they will all get it here, the ones I can do. I don't know whether you have gotten what....

Moderator: yes, yes

Moderator: that's okay this the last question we want to ask Is there anything else you think we should know about hepatitis B, sharing information about hepatitis B, or the hepatitis B birth dose? Is there anything we didn't talk about that you would want to bring into this discussion let us know feel free to do that

Answer: materials to do the work like cotton wool is not there, they have not provided any cotton

Moderator: okay materials to do the work should also be supplied things like cotton wool

OIC: gloves although they supplied us something like that to us

Moderator: You mean sanitizers

OIC: yes

Moderator: thank you so much for your time we appreciate the responses you have given and we say a very big thank you

State 2, LGA 2, Facility 2

Interviewer: my name is [name] and my collogues name is [name]. we are from the African Field Epidemiology Network. We have come to this health facility [facility name] in [LGA name] to conduct a key informant interview for a pregnant woman, so feel free calm down and respond to the questions we are going to ask you

Respondent: No problem

I: thank you, before we start the questions may we know your age? How old are you

Age: I am 22 years old

I: 23 years

R: 22 years old

I: 22yrs old

R: yes sir

I: What do you know about hepatitis B disease?

R: the little knowledge I know about it is that. it is a disease that normally treated in pregnant woman during about 3 to 6 months of pregnancy to avoid some sicknesses after childbirth

I: What do you know about the hepatitis B birth dose vaccination?

R: I don't have the idea of that one

Interviewer: ok, you don't have idea of it?

Respondent: yes

Interviewer: Have you ever asked for the hepatitis B birth dose before for any other children?

Respondent: I have not

I: Do you plan to get the hepatitis B birth dose vaccine for your baby, this baby that you are carrying now when you give birth to the baby?

Respondent: when I gave birth to the baby if I get the actual information about it I will conduct it on my baby

3a. Why would you want to do it for the baby when you give birth to the baby?

Respondent: to avoid some sicknesses

Interviewer: are there examples of sickness you would want to avoid?

Respondent: like yellow fever, eemmm this running stomach of a thing

I: Do you have any feelings of concern, stress, excitement about this hepatitis B birth dose vaccination?

Respondent: No I don't have

Interviewer: Why don't you have?

Respondent: Hmm I don't have, I don't have a reason for that

Interviewer: Do you think the hepatitis B birth dose vaccination will work to prevent hepatitis B?

Respondent: Yes I think so

Interviewer: why?

Respondent: Because they say that prevention is better than cure

Interviewer: Do you think you could easily get the hepatitis B birth dose vaccine if you asked for it?

Respondent: Yes I think so

Interviewer: why do you think so

Respondent: I don't know the reason

Interviewer: But you feel that if you give birth and come to this health center and ask for it you will get it

Respondent: yes

Interviewer: Will you ask for it when you give birth to this baby?

Respondent: Yes I will

Interviewer: Why do you say that?

Respondent: If I ask the nurses and doctors here they will

Interviewer: What I mean is that you say you will ask for it, why.....

Respondent: Because I told you before that I want to prevent some sickness after my childbirth

Interviewer: Is there anything that might be a challenges that will face you related to accessing the birth dose, is there anything that will be challenge that will make you not access or get this vaccine that we are talking about when you give birth to this baby

Respondent: Something like money. If I ask for it and the money they are asking for is too much, that is the only thing that will stop me or that will be a challenge to that.

Interviewer: Do you think that hepatitis B birth dose is important for you to get for your child?

Respondent: It is important,

Interviewer: why is it

R: in order to prevent some sicknesses

Interviewer: In general, do you think pregnant women in your region Ugbuezeji get the hepatitis B birth dose vaccines for their babies?

Respondent: Yes I think so

Interviewer: why do you think so

R: Because the nurses used to teach us about this hepatitis B and how to avoid it after giving birth to a child

Interviewer: What gives you that level of confidence (or lack of confidence)?

R: Nothing gives me o but in my mind I believe it is good to follow the nurse's order than to do off-head

Interviewer: Do you have any suggestions for us if we want to educate people in your community about the hepatitis B birth dose vaccine?

Respondent: I don't understand

Interviewer: the next question I want to ask is that do you have any suggestions for us if we want to educate people in your community in Ugbuezeji about the birth dose of hepatitis B vaccination, is there any suggestion that you can give us on the way we can do it so that it will be good

Respondent: The only way I know is that number one thing is that you people need to gather some people talk about this hepatitis B. and after talking about it the money involved in the drugs, you people will make it to be cheaper n affordable that is the only way the people will love to participate in it.

Interviewer: Would you prefer to learn about hepatitis B Through stories, videos, flyers, other methods, how do you refer to learn about Hepatitis B

Respondent: I prefer practical aspect of it

Interviewer: like?

Respondent: Like when you come to this health center after teaching us, after teaching the people you people will practice it. As in give immunization and when people see that your drugs are working, they'll start following the order.

Interviewer: in general how do you prefer to learn about health issues, if we want to tell you or teach you about health issues in general how do you prefer to learn it

Respondent: You people will bring the equipment for it so that after teaching us u people will practice it. As in give drugs and we will go home to take it.

Interviewer: are there other ways that you would understand it better, like I have got this ne you have answered now. Is there another way that when they teach you about health issues that is normal easy for you to understand.

Interviewer: Is there anything else you think we should know about hepatitis B, sharing information about hepatitis B, or the hepatitis B birth dose vaccination that we may not mentioned during our discussion is there anything you want us to know, tell us

Respondent: I don't have any other one.

Interviewer: are you sure?

Respondent: I'm sure

Interviewer: you know we have asked a lot of questions is there something you feel is important that you may not have mentioned and also want to tell us about feel free

Respondent: They only uh uh the only thing is to go to the hospitals and let people know about it before childbirth.

Interviewer: okay so we should let pregnant women when they are coming for ANC

Respondent: That's the only suggestion

Respondent: Yes, yes, we educate them on it.

Interviewer: that's okay thank you for your time.

Respondent: You are welcome.

Interviewer: we appreciate

Note taking template for Health care worker KII guide State 2, LGA 2, Facility 3

Demographic information of interviewee

Age: 35

Sex: Male

Designation: Chief Medical Officer

Questions

1. What are the biggest challenges for people living with hepatitis B in your community?

You know the infection is something they don't actually know they have, sometimes they might be thinking they're charmed, sometimes they might be thinking they are charmed by witchcraft in the society, but its only when the doctor has a very good skill that he can send the person to the good investigation, maybe Hepatitis B Surface antigen assay, that is when you can now spot the individual with a positive case. So, on that note; one, ignorance, they are not aware, many are not even aware they have it, then for the people that have it, they still doubt if it is true because they are always influenced by the negative aspect of the practice.

2. What are the biggest needs to address hepatitis B in your community?

Number one is Group Awareness and Treatment

3a. Is there a strong need for the hepatitis B birth dose? (Why or why not?)

Yes, the reason is that if a person is given a birth dose, it will build up enough resistance for the individual towards catching that virus even if he's exposed to the society.

3b. Do others see a need for the hepatitis B birth dose?

Yes, for people that are not so resistant to immunization. You know some families are against immunization due to their faith or maybe its their practice, but for people who acknowledge that immunization is key to good health, they don't have any issue with that.

4.Can you tell me about the current programs or practices for the hepatitis B birth dose in your clinic?

For instance, during antenatal, we do some routine investigations and during those investigations Hepatitis B is one of the cardinal tests. For instance, we do Hepatitis B, we do the VDRL, we do the other relevant investigations but Hepatitis B is a must. So when you screen a woman during pregnancy and discover that she is positive, you will commence her on treatment and then counsel her through that pregnancy and her medications and the need for birth dose.

4a. Is the hepatitis B birth dose easy to get within 24 hours of birth? (Why or why not?)

Yes, it is easy because due to the help of these ehhhh satelite activities, one like SOML activities, they are making it possible to ehhh install freezers for storage of hepatitis B vaccine in all the facilities offering immunization.

4b. Who can administer the hepatitis B birth dose?

The nurse, nurse/midwife

4c. What are challenges/barriers to getting the hep B birth dose vaccine?

For us in Enugu, we don't have much challenges, or any challenge at all because it is always a must that we have that. So, in any functional facility offering immunization, Hepatitis B birth dose is always in stock waiting for any pregnant woman to deliver and its always given within 24hours of birth.

5. Have you gotten information from participants regarding their experiences with the hepatitis B birth dose?

Yes. For instance, some women who deliver at ehhhhh birth attendants, birth clinics maybe in the,you know here is covering some areas in the urban slums. Some people who patronize the the birth attendants, after delivery they don't get the Hepatitis B because they don't have it there. So, some of the attendant nurses who have the knowledge will always refer them to centres where they can receive but sometimes, those women don't access the vaccine. So, as a result, you have some people that will miss that birth dose ehhhh antigen.

5a. What is the community understanding of the hepatitis B birth dose?

Well, recently, the understanding is very good. More especially this time Hepatitis B is always advertised everywhere in radios, in jigles, you know within the ministry of health they carry out campaigns, series of campaigns about Hepatitis B, so the awareness is always on air, and people who attend antenatal, Hepatitis B is a key issue we discuss.

5b. Are people interested in getting the hepatitis B birth dose?

Yes and No. Yes, for the enlightened ones, No for the illiterates.

5c. Can you describe what kind of specific information/messaging you have heard?

Well, during the last Hepatitis B day, ehhhh if you just you're your radio you will hear jingles, need for Hepatitis B ehhh for pregnant mothers to attend antenatal, screen for Hepatitis B and when they deliver the necessity for them to get their birth dose, it's always on air. Then secondly, during antenatal we still like sing it to their ears "please make sur you receive your birth dose for Hepatitis B

6. Do you have any ideas or suggestions for improving hepatitis B birth dose vaccination rates?

Yes. For instance, we need to increase the awareness, like the SMAT survey is going on, the awareness is not too high in the society because they only understand that the only viral infection, we have is HIV and apart from HIV, Covid. Its only now that they are understanding that Hepatitis is even worse than HIV. So, the awareness should be there, create more awareness, that's number one. Then number two, empower the health workers on the need for them to reach out to their clients. For instance, series of training, training resource persons on the need for this.

6ai. At the community level

Okay, at community level, yes, we need to like carry out outreaches, you know immunization has a way of getting to outreach centres apart from the services you get from the hospital, you carry out outreaches, that's always done in churches and schools, maybe girls' schools, boys'

schools, and then in the market places then awareness, then you increase the rate at which we advertise in the radio, yes.

6aii. Health care system level

In the ministry of health, they are doing a wonderful work. I think they have a resource person in charge of Hepatitis B at the Ministry of health. Then for the Agency they should still have, like a synergy, they're working in synergy, they work hand in hand simultaneously like what the agency doesn't have, the ministry will augment, but the ideal thing is that the agency should have a resource person also trained on that, trained on Hepatitis B awareness.

Then, at the health facility level, like here in our own condition we have a resource person who is in charge of that health facility, and that resource person is doing a dual job like a Hepatitis B resource fellow and the immunization resource fellow, because it's always there that you can catch them young, maybe the women then we give them good awareness and the doctors should as well be be conversant with what is happening in the society about the scourge of ehhh Hepatitis B.

6aiii. Provider level

Yes, we can empower them, make sure that the test kits are there then if the test kits are there, then the medication, because if we don't supply them with medications, it's always very dangerous because if you ask them to go into the market and source for antiretroviral which is the key drug, it's always very difficult. So, if there's a way the organization can sponsor the provision of those drugs for Hepatitis B positive patients.

For vaccination, vaccination is very strong here. Its not something that is new, it is there ehhhmmm and functioning. Its only if we have like outreaches, new settlements and we're introducing the settlement for an outreach that we can now empower those going for the outreaches. That is empowering those going for those outreaches, because you know we have so many ehh settlements growing up around this Iji Nike because it's a new settlement and new sites are growing up and we're extending our services to those new sites. Then, you empower the health workers going to to reach out to those people.

6b. Are there specific strategies for education or awareness that might be most effective?

Yes, by use of churches and then schools. We have a good counsellor at the health facility setting and this counsellor can have an outreach, you know cave out time to you know visit the

nearby schools and churches once in a while to educate them on the need for testing for Hepatitis B and its medications.

7. Can you tell me about your experience with the maternal tetanus vaccination?

Yea, you know maternal tetanus, in this part of the country, the women don't receive live vaccine, so what they believe is that whenever a woman conceives, she'll receive the first dose then maybe a month after the second dose. So sometimes, if the woman did not attend a good centre for her antenatal, she may lose those two doses but eventually you see that a woman keeps on receiving those two two doses at each ehhh pregnancy. For instance, if she has just 3 pregnancies its just two times three, six shots, that is sporadically, it's not something that they do routinely. So, I believe that the ideal thing is that health education for our young girls in schools to be receiving normal tetanus eehhhh full vaccine.

7a. What barriers or challenges have you experienced related to the maternal tetanus vaccination?

Uhhhmmm, number one, for the illiterates, they deliver at poor centres, what do I mean by health ehh care services in the villages, that is they don't receive the the vaccine. Secondly, if you screen a woman and she's positive and you instruct her on how to get the drug for the vaccine *sighs* the drug for the disease, she may abscond and eventually land in the birth attendants care and by so doing she would still not receive any medication and the still breastfeed the the child, the baby, and at the end of the day the baby will end up receiving, getting Hepatitis B from the mother due to ignorance.

7b. What successes have you experienced, what is working?

Ehhhmmm, for instance, in the recent past I've discovered a family with almost all the siblings positive from Hepatitis B and the same Hepatitis B killed the parents. So, we as a result of that occurrence mounted a survey like it's on our schedule that any patient tha come sto the clinic will be screened. Any patient that comes, the way we're screening for HIV, we're at the same time screening for Hepatitis B. So, if we get any positive patient, we guide the patient, after counselling, we help the person in purchasing the drug and then make sure that the person is on the medication till the person recovers.

8. What types of health interventions have been the most successful in your community and why have they been so successful? Uhmmmm, you know health intervention is just like give and take. In our society, you know we have so many poor people there. They believe in offering free health services, so if we have an outreach, we do all these tests free of charge. Then for people that are positive, we counsel them and then commence them on our medications, maybe help them in providing the drugs though they will pay. Cos if you allow them to go and buy it, due to the fact that some of those medications are antiretroviral, they may not end up buying. They will just go and resort to herbs, herbal intervention, that kind of it.

9. Is there anything else you think we should know about hepatitis B, sharing information about hepatitis B, or the hepatitis B birth dose?

Well, the only thing you should know is that Hepatitis per say, though its treatable but you still need to be like, be consistent. If it is possible you will help us, as in help in providing for the test kits, and if there's any means of getting the drugs, because for us here ehh the CCCRN helped us in providing the test kit for HIV and their medications. So, if Hepatitis B can run concurrently with, like that of Hepatitis B *sighs* like that of HIV, it would be better so that if you detect somebody, you give the person drugs free.

For birth dose, I don't think, we're having much challenges now because the awareness is everywhere. Even people living with the positive thing, their babies are not even getting the virus because they are on medication. If we do, their panel test, you'll see that even though they're still positive, so they can't transmit it to their babies.

Yes, you'll empower us to strengthen our health education unit so that we can reach out to people still in there that have not heard anything about Hepatitis B

Note taking template for Pregnant women KII guide State 2, LGA 2, Facility 3

Demographic information of interviewee

Age: 27years

Questions

1. What do you know about hepatitis B?

From the little one I know is that they say that it's a deadly disease and it's contacted by through sexual intercourse and also breastfeeding of baby.

2. What do you know about the hepatitis B birth dose?

Birth dose vaccination. Ehhhh, what I understand there is that that vaccination they used to give them, help them to prevent the Hepatitis B, so that it will not cause harm to our children.

2a. How do you feel about the hepatitis B birth dose?

Well, the birth dose. I feel that whenever my kids take it, they are protected against the diseases.

2b. Have you ever asked for the hepatitis B birth dose before for other children?

Uhhhhmmmmm, No oh, it's ehhhh this my second son they inject it because that one, that Hepatitis B is not out from 2017 that I gave birth to my first daughter so I knew about it from 2019 that I gave birth to my second son and it was given to him even the one they call meningitis

3.Do you plan to get the hepatitis B birth dose for your baby?

Yeah, I'll give it to him because I heard that its protected them from that disease.

3a. Why or why not?

Why I want to give it is that to protect him or she against that disease so that He or she will not contact it.

3b. Do you have any feelings of concern, stress, excitement? Why?

No oh, I don't have because I have tested myself and I don't say that I am contacted to it, so I feel free that my child will not contact it.

4. Do you think the hepatitis B birth dose will work to prevent hepatitis B? (Why or why not?)

Uhmmm, I think that it will help because like when we gave birth, they advised us to protect eh our children this immunization of a thing they say that it protects from those diseases so that our children will not contact it. So, I strongly believe that by taking the injection, the vaccine, it will help them not to contact.

5. Do you think you could easily get the hepatitis B birth dose vaccine if you asked for it?

Hmh, I think, because it is now common in every hospital.

5a. Will you ask for it?

Yes of course, but there is a stages of that, its not the first dose that you'll protect your baby.

5b. Why do you say that?

The reason I have said it before, to protect them against the disease.

5c. Is there anything that might come in the way or challenges you might face related to accessing the birth dose?

sighs nothing

6. Do you feel the hepatitis B birth dose is important for you to get for your child? (Why or why not?)

Hmmmm, the same question and the same answer I suppose. Why is that to prevent them from getting the disease. I feel that it is important as they have taught us.

7. In general, do you think pregnant women in your region get the hepatitis B birth dose for their babies?

Heaa, I won't say yes because I'm not like them. Everybody has his own understanding, you understand? From my interaction with them today because it's today that I saw them, so, yea even the ones in the community, my friends yea, the ones I know I believe that they'll get it for their babies.

7a. What gives you that level of confidence (or lack of confidence)?

Yea, *laughs* as they said, because they're my friends and I knew them, we chat, we discussed, I believe that its important for them to give it to their baby when they give birth.

8. Do you have any suggestions for us if we want to educate people in your community about the hepatitis B birth dose?

Uhhhh, its good sha because not everybody. Okay, well the little one I know is that like these Wednesdays they used to Have this their thing. No this, I forgot, Antenatal day, so that's the best way I suggest that you should come and lecture them about it because there's no process for you guys to go and teach about.

8a. Would you prefer to learn about hepatitis B? (Through stories, videos, flyers, other methods)

Hmmmm, I prefer teaching, video is okay too. I don't like stories because with stories, its not what you say that another person will say. I prefer the professionals to teach me about.

8b. How do you prefer to learn about health issues?

Ehhhh, how I prefer is that like now I'm here in the hospital, I prefer that if I want to learn anything about health I'll go to the hospital, consult my doctor or the nurses.

9. Is there anything else you think we should know about hepatitis B, sharing information about hepatitis B, or the hepatitis B birth dose?

sighs I don't think, its like you have said it all.

Note taking template for Health care worker KII guide State 2, LGA 2, Facility 7

Demographic information of interviewee

Age: 28years

Sex: Female

Designation: Community Volunteer Health worker

Questions

1. What are the biggest challenges for people living with hepatitis B in your community?

We don't normally have eh this thing, we don't normally have ehhh as in have eh Hepatitis B patients here, so…

2. What are the biggest needs to address hepatitis B in your community?

The thing is that, you people should provide us with equipment like something that we use to be conducting as in to test them, you understand, yes. As in the strip for the we use and do the lab to know whether they….

3a. Is there a strong need for the hepatitis B birth dose? (Why or why not?)

Yes, you know some people will have eh Hepatitis, that Hepatitis but they will not know that they have it, but if you test them, the result will show whether they have it or not.

For prevention

3b. Do others see a need for the hepatitis B birth dose?

Yes.

4.Can you tell me about the current programs or practices for the hepatitis B birth dose in your clinic?

Repeat the question again. They used to come and take the injection as in the immunization. They give them as in the first ehhmmm after birth, so they give them the injection

4a. Is the hepatitis B birth dose easy to get within 24 hours of birth? (Why or why not?)

Yes, because we have eh this thing that we are storing vaccines so eeh every time of any day the vaccine is with us here.

4b. Who can administer the hepatitis B birth dose?

The health workers as in I mean the nurses that is working here.

4c. What are challenges/barriers to getting the hep B birth dose vaccine?

There is no any challenge

5. Have you gotten information from participants regarding their experiences with the hepatitis B birth dose?

No,Yes. Some people came, you know the hepatitis B vaccine ehen they say that there is a time it will be and the child will not take again because the other this thing, the other immunization that have it. So, the say that after birth if they did not get the Hepatitis B vaccine within like one month to two months that they are no more going to take it that its only BCG that they give them then the rest will be given them in that Pentavarous vaccine

5a. What is the community understanding of the hepatitis B birth dose?

What is there? Okay, they see it as a good thing, yes. For giving their children Hepatitis B vaccine.

5b. Are people interested in getting the hepatitis B birth dose?

Yes.

5c. Can you describe what kind of specific information/messaging you have heard?

Messages, nothing sha, they said that it's good, that its good, nothing like that.

6. Do you have any ideas or suggestions for improving hepatitis B birth dose vaccination rates?

Yes,

6ai. At the community level

okay by announcing as in going from inside villages and streets to announce it that everybody that gave birth should come and take Hepatitis B vaccine that its very very good for the health of the children

6aii. Health care system level

To make sure that they are providing the vaccine, that the vaccine is ready. That each time that parents bring their children come that there will be vaccine to give to them.

6aiii. Provider level

That the health worker be punctual to work, each time, they'll have to be at the right place, like today in the health centre. So, that if the people come, they'll be able to meet somebody, a nurse that will attend to them.

6b. Are there specific strategies for education or awareness that might be most effective?

You mobilize it, to mobilizing it. To mobilize, going using this eh thing to mobilize, going to inside villages, communities, streets, announcing there to come and take Hepatitis vaccine.

7. Can you tell me about your experience with the maternal tetanus vaccination?

It is good to take your tetanus vaccine

7a. What barriers or challenges have you experienced related to the maternal tetanus vaccination?

Some people did not used to come to antenatal till they get to nine months. That day they will come to deliver is the day they will come to register for antenatal by that time you'll find out that they are in labor. So, eventually, they will not take that Tetanus vaccine up to that time.

7b. What successes have you experienced, what is working?

Yes, things are working well. All I know is that that ehh tetanus vaccine is good and Hepatitis B vaccine is good so everybody that give birth should come and take eh that eh Hepatitis vaccine and those the pregnant women for that tetenus. Because I have seen somebody that is giving birth and in that process she start having convulsion, that I learnt that its because they did not take that eh tetanus vaccine. So, that is it, its good to take that drug.

8. What types of health interventions have been the most successful in your community and why have they been so successful?

Eh, they are doing good antenatal days and immunization days, they used to teach some the eh the importance that you give your child ehh immunization like that Hepatitis, BCG, hepatitis B vaccine and others. So, they're doing good by teaching.

9. Is there anything else you think we should know about hepatitis B, sharing information about hepatitis B, or the hepatitis B birth dose?

You have said everything all.

State 2, LGA 2, Facility 7

Interviewer: We want to conduct a Key informant interview for pregnant women in [facility name] in [LGA name], Please may we know your age? What is your age, how old are you

Respondent: 31 years old

I: 31 years old okay, What do you know about hepatitis B, the disease called Hepatitis B what do you know about it?

R: actually I don't know much, I know it's a disease of new born babies that affect them when they are born

I: What do you know about the hepatitis B birth dose vaccination?

R: no, I don't have an idea

I: no idea, How do you feel about the hepatitis B birth dose?

R: how I feel

I: yes about it

R: I feel it is a dangerous disease that need to be taken care of.

I: Have you ever asked for the hepatitis B birth dose vaccination before for other children, this hepatitis B vaccination have you ever asked for it for the other children you have given birth to, have you ever asked for it?

Answer: yes,

I: where did you ask for it

R: at the hospital

I: which hospital?

R: Chukwu asoka hospital

I: Do you plan to get the hepatitis B birth dose for your baby this baby that you are carrying now do you plan to get the hepatitis B birth dose vaccination for this baby when you give birth to him or her?

R: yes

I: Why do you say that why do you plan

R: well actually I plan because prevention is better than cure, I want to prevent it before it affect my baby as a mother that is the primary thing I have to do for him or her

I: Do you have any feelings of concern or stress or excitement about the hepatitis B birth dose vaccination? Do you have any concern or any stress?

R: my concern is that as government has started doing the work of it, they should take it more serious to avoid spreading of the disease.

I: Do you think the hepatitis B birth dose vaccination will work to prevent hepatitis B disease? Do you think that vaccination will work to prevent the disease?

R: I think it will work.

I: why do you think so

R: why I think so for the fact of making effort to prevent it, so government will not just bring what that cannot stop it. So What they bring I think it's for the betterment.

I: Do you think you could easily get the hepatitis B birth dose vaccine if you asked for it? Do you think you can easily get it if you ask for it

R: I don't think so

I: if you come to the hospital to ask for it, when you give birth to this your baby do you think that they can easily provide it for you? Assuming you give birth to this baby and you come to this hospital and say you want Hepatitis B birth dose vaccine for this your baby let them give it to your baby, do you think that can easily be done for you by the health workers?

I: do you understand the question

R: yes I understand but

I: my question is assuming you give birth to this baby.....

R: and they do not give him or her injection immediately....

I: nmmmm nmmmm the question is do you think that is if you give birth to this baby that you can easily get that baby to be vaccinated?

R: yes

I: why do you say it?

R: well I say yes because I think the vaccine has been in the hospital and when I ask of it they will give me

I: Will you ask for that vaccine when you give birth to this baby

R: yes

I: Why do you say that that you will ask for it when you give birth to this baby? What is your reason?

R: number one reason of asking for it has been the right of that my baby to be injected and second one is that I have been informed of the vaccine so if I did not ask for it then am denying that my child

I: Is there anything that might be a challenges you might have or face related to accessing this hepatitis B birth dose vaccine? Is there anything that might be a challenge?

R: for me to get it

I: yes, is there anything that may come in the way or challenges you might face related to accessing the birth dose vaccination, is there anything that can prevent you from getting it, when you give birth to this child is there anything that can prevent you both at your level, the level of the hospital, is there anything that can prevent you at the level of the community is there anything that can be of a challenge?

R: the only thing that can be of challenge is scarcity of it, if they say it is not here now that is the only thing that can a challenge

I: Do you feel the hepatitis B birth dose vaccine is important for you to get for your child?

R: Yes

I: why?

R: It is important for his or her health for his or her health living.

I: In general, do you think pregnant women in your locality region in Nchatancha get the hepatitis B birth dose for their babies? Generally do you think that women in Nchatacha community will bring their babies for hepatitis B birth dose vaccine do you think so?

R: I don't think that all do.

I: you don't think that all do? Okay why do you say so, what gives you that confidence to say that?

R: why I say so is that you that some pregnant women they take some things unserious. So I am not thinking that everybody will like to come and receive it as

I: but some

R: some ma8. Do maybe 50%

I: do you have any suggestions for us if we want to educate people in Nchatacha, pregnant women in Nchatancha about the hepatitis B birth dose, do you have any suggestion on how we

can educate them so that they will understand it is there any suggestion you would like to give us on the method we can use?

R: the only suggestion I have is that calling and educating them on it on how important it is. How safe it will make their baby to be.

I: how would you want us to do that education? which method?

R: calling them

I: okay calling them

R: you put information across so that all them will come all the pregnant women will come.

I: how Would you prefer to learn about hepatitis B, if somebody wants to give options on how you would prefer to learn about this hepatitis B? how will you prefer it which method to learn?

R: is there any other method?

I: like through stories, videos, flyers so which method works best for you that will make you understand it if you want to learn about hepatitis B

R: reading

I: How do you prefer to learn about health issues generally?

Answer: reading as well.

I: reading as well okay. Is there anything else you think we should know about hepatitis B, sharing information about hepatitis B, or the hepatitis B birth dose that you would like to share with us that we may not have talked about during our discussion with you?

R: No

I: no?

R: no

I:s thank you so much for your time

State 2, LGA 2, Facility 8

INTERVIEW TRANSCRIPT

Interviewer: Good afternoon ma. We want to conduct key informant interview for the midwives in [facility name] in [LGA name]

Respondent: Yes

I: Please may we know your age? How old are you?

R: I'm fifty

Moderator: Fifty. Okay. What is your designation.

R: I'm Deputy director community health

I: And you're the midwife that delivers children here?

R: Yes

I: What are the biggest challenges for people living with Hepatitis B in this community. What are the biggest challenges, the things you think are the biggest challenges for the people that have Hepatitis B disease in this community?

R: you know We don't treat them here, we normally send them for-to the bigger health facility like eh Annunciation or UNTH because when they go for test and they know that they have Hepatitis B, we normally tell them that hepatitis B is a sexually transmitted disease. That their partners can get it. That their partner supposed to go for test. So after the test, if the partner is negative, we now tell them that they will go and have hepatitis B--that the partner will go and have Hepatitis B injection and we don't give that one for adults. We give that one of children, newborn here. They will now go to Annunciation, they will go there, they will give them.

I: So, are there some challenges you feel they face?

R: Some of them doesn't go after telling the person everything. Some say they don't have money to have the injection--the partner that is negative. I will tell the person that you can get it from your husband if it's the woman that is negative. Some will say they don't have money to continue for the injection. Some do go and have the vaccine there.

I: Okay. What are the biggest needs to address Hepatitis B in your community? What are the biggest needs to address--what are the things that are needed to address this Hepatitis B disease in your community?

R: If you can come and eh give them lecture about it. Some of them doesn't even know about Hepatitis B. Some know it through us health workers when we give antenatal mothers health talk about Hepatitis B or during the immunization. But I know most of them that do not come for antenatal or immunization, some may have the disease and they don't know about it. But if you people can come and give them training about it, meet the community leaders or the women, they will know about it.

I: Is there a strong need for Hepatitis B birth dose vaccination in your community? Is there a strong need?

R: Yes

I: Why?

R: To protect the newborn from having that disease.

I: Do others see the need for Hepatitis B birth dose in this community? Do you think other people, other women see the need?

R: They need it. they see it but some of them doesn't deliver here. Some deliver to TBAs and when they deliver them, it is only those people that maybe came for antenatal here, that will come back for the hepatitis B immediately. But those people that have not heard about it will stay till on the immunization day, they will come. And I normally tell them that after two weeks, we cannot give you again because that hepatitis is included in pentavalent vaccine.

I: Can you tell us about the current programs or practices for Hepatitis B birth dose in your clinic? What are the things you do, the programs, the practices you do about this hepatitis B birth dose in your health facility?

R: This is, when somebody put to bed, before 24hours we give the child hepatitis B

I: Is that what you practice here?

R: Yes! And on immunization day, we give immunization every Wednesday, we normally give them.

I: Is the Hepatitis B birth dose easy to get within 24hours of birth?

R: Yes!

I: Why?

R: It's always available, we have solar fridge here. so the vaccine is always there in the fridge and somebody is always there.

I: Who are the people that can administer Hepatitis B birth dose in this your health facility?

R: I am the and my immunization focal person.

I: What are challenges or barriers to getting Hepatitis B birth dose vaccine? What are the challenges or barriers you have?

R: We don't have any barrier. We normally go to LIO and they normally give us the cold chain they normally give us. The only thing, we transport yourself. They don't give us money for collection of vaccine or anything. They only ask you not to take any 5 naira from any woman. And like this, em, during dry season, solar fridge can stop working, we normally call somebody. Pay the person, collect ladder and clean the panel. Because if you don't clean it, the vaccine inside the fridge will just damage.

I: Have you gotten any information from clients, pregnant women or people who are coming to your health facility regarding their experience with your Hepatitis B birth dose vaccination? have you gotten any information from them that you want to share to us? What do they say about hepatis B? these women that come for vaccination, these women give birth in your health facility?

R: They don't say anything. The vaccine is just okay for the children. they don't have any adverse event following that immunization.

I: What is the community understanding of the Hepatitis B birth dose? This community where we are, Onu-Ogba community, what is their understanding of hepatitis B birth dose in your own assessment, what do you think is their understanding of this Hepatitis B birth dose?

R: Their understanding is that, they know that, we educate them that this Hepatitis B is infectious if the child did not have the vaccine as soon as possible. so that is what I understand.

I: Do you think people are interested in getting Hepatitis B birth dose in this community?

R: Yes.

I: Okay. Can you describe what kind of specific information or messaging you have heard concerning this Hepatitis B birth dose? Can you describe the specific information or messaging YOU have heard?

R: The message I have heard about is that, it increases child's immunity and it protects the child not having the disease, maybe from the mother or from any other person.

I: Do you have any ideas or suggestions for improving Hepatitis B birth dose vaccination rate that you want to share with us? The ideas or suggestions you want to…

R: It's only educating people by way of maybe, you can come and do some dramas for us or training the community, educating the community. so that they will know Or even if you have posters, we can even put it

I: So at community level…

R: Yes

I: What do you say about community level, what can be done?

R: Educating them.

I: Okay. What about at the level of the health care system? Like the, at the level of the ministry of health, the agency, the health facility, what do you think can be done to increase the rate?

R: Just what we need is, we need more workers. We're understaffed. One person will do this, do this, if you're there in the delivery room maybe on immunization day you'll be on the delivery room, you'll be working for immunization, you'll be treating patients. We don't have enough workers at Enugu East here.

I: What about at the level of the health care worker? At the level of the healthcare provider, you people, what do you think can be done at your level to help increase Hepatitis B birth dose vaccination rate in this community?

R: If you people can help us in transporting it. It can help us. Like when I start job at Ezielo, that time, we have MPI bus, that bus normally go to the LGA collect vaccine. On the day, if you want to, the day we are going to give immunization the bus will just carry our drug; just like somebody that is selling bread. You come out with your Goi'style you put your vaccine. You show the person the requisition, they'll give you. After, at the end of the day, the person will come for the data. But here, we don't have such thing. We normally go on our own. For a whole day you'll be at Abakpa for collection. Because you have so many things to write there. You leave the facility open, if there is somebody that delivers, if I'm not there to give that 24hours…So if you can help us and let them know that we are understaffed, though we have been telling them. If they can employ people, if will help us in this system. So that when somebody delivers, immediately, after some hours, you will give the child that vaccine.

I: Okay. Are there some specific strategies for education or awareness that might be the most effective?

R: Even that drama is normally educative and simple to understand because when you do drama somebody may be laughing but maybe tomorrow, the person can think out something from that thing.

I: Okay. Can you tell us about your experience with maternal tetanus vaccination?

R: Maternal tetanus vaccination, this Td Tetanus Diphtheria* We normally give women at end of one month and give after one month. But there is another one called LIFER. We normally give that one five times but not in one pregnancy. That five times may be by three years the woman will cover it. We'll issue the woman with a card, that LIFER means if you finish that five times you will not have that immunization again for life. But that two times is what most of them are having. We give them at the end of four months and after one month we give another one.

I: What are barriers or challenges you have experienced related to maternal tetanus vaccination?

R: Some of them do not start their antenatal clinic on time. Some will come even by seven months, some will come by nine months. So when you give that person that vaccine, you know that the baby have that vaccine through--from the mother. If the woman did not take that vaccine on time, the child will not have that vaccine and the woman will deliver. That is, some of them, I've had experience, a woman umbilical cord, a child have umbilical cord that cannot heal. They said that, one doctor was saying that it's just because of the woman did not have Tetanus Diphtheria injection on time.

I: What successes have you experienced? What is working for you here? Can we know the successes that you've experienced, what works that you want to let us know?

R: What I normally tell women about that Tetanus Diphtheria is that they supposed to have it on time so that their baby will have it and it will work for their baby. Because that vaccine is for the baby and for the mother. If the mother does not have it on time, the baby will not have it.

I: So do you experience success in those...

R: Yes! Yes.

I: Okay, how? Can we know how?

R: Just about that umbilical cord, when we have that baby, we use this Chlorhexidine gel and the whole thing will fall out as at when due and the baby will be okay.

I: What types of health interventions have been the most successful in your community? And why have they been so successful? The health interventions that have been successful in this community and why do you think they have been so successful?

R: Well, health interventions, by the time, before I came to this clinic, this clinic was not functioning. No, even immunization, we were giving three persons in one month. So, but now, we know there is changes.

I: So why, why, how do you....

R: I normally stay from morning till night every day. If I came to work, I will not go until last. And when I'm going I know another person is there. So we normally stay that 24 ho...I give them that 24hours service. It helps people to be coming and going.

I: Is there anything else you think we should know about Hepatitis B, sharing information about Hepatitis B or the Hepatitis B birth dose vaccination that we may not have mentioned...

R: No

I: ...In the course of this discussion, you want us to know, feel free to share with us.

R: No oh, you people know almost everything about Hepatitis B

I: No, what I mean is there anything we didn't touch?

R: No

I: ...Bring up?

R: No.

I: Thank you so much for your time. Thank you for your responses, we really appreciate. God bless you ma.

R: Thank you.

State 2, LGA 2, Facility 8

Interviewer: Good Morning all, [LGA name] team we want to conduct key informant interview for a pregnant woman at [facility name] in [LGA name], so may we know your age. How old are you?

Respondent: I am 27, 27 years old

I: 27 years old, Ok. What do you know about hepatitis B?

R: all I know if that Hepatitis B is a disease of the liver, that's the main thing I know about Hepatitis B.

I: do you know about Hepatitis B birth dose vaccination?

R: Yes, Hepatitis is giving before 2 weeks, from birth to 2 weeks with BCG and OPV

I: OK

R: and is giving 0.5mil

I: how do you about Hepatitis B birth dose vaccination? How do you feel about it.

R: Is good because it protects our children against hepatitis.

I: have you ever asked about Hepatitis B birth dose before for other children outside this one you are pregnant for now.

R: those ones I have giving birth to before?

I: yes the other children you have given birth to before, have you ever asked about Hepatitis B birth dose vaccination before.

R: after the first dose

I: No, when you gave birth, do you ask for the vaccine to be given to your children

R: Yes the OIC in charge told me about it, so no need of asking so I just give my children the vaccine.

I: do you have plan to get Hepatitis B birth dose vaccination for this baby you carrying for now.

R: yes of course

I: why?

R: because I know is very important in other to protect my baby against Hepatitis B.

I: Do you have feeling or concerns or any stress or excitement concerning this Hepatitis B birth dose vaccination? Do you have feeling or concerns or any stress or excitement about it?

R: well I am happy about the Hepatitis B birth dose vaccination because is good.

I: why? Ok, because is good

R: yes. it helps our children.

I: yes. it helps our children? alright, do you think Hepatitis B birth dose vaccine will work toprevent Hepatitis B disease?

R: yes of course

I: why?

R: the reason because that first dose it will your baby the immunity he needs in other to prevent him or her from getting that disease Hepatitis B.

I: OK, do you think that you can easily get Hepatitis B birth dose vaccine if you ask for it? Can yu get it easily?

R: YES, because is already at the facility.

I: so will you ask for it when you have this baby?

R: yes I will ask for it because I know is very important for my baby to take it.

I: why you will ask for it is because you know it is very important?

R: yes

I: and it is good to take it

R: yes off course

I: is there anything that might come in the way or challenges you might face related to assessing the Hepatitis B birth dose vaccine, is there any challenge you might face if you want to give it to your child?

R: No, because the first one I tried I didn't have any challenge, so I don't think there is any challenges.

I: do the feel that the Hepatitis B birth dose is Important for you to get for your child?

R: yes

I: why do feel is important?

R: it prevents my child from getting that disease Hepatitis B,

I: ok

R: because I know that my first baby the one I gave to her will prevent her. so is good.

I: in general do you think that pregnant women in your area, your region, in this community get Hepatitis B birth dose for their babies? In general do you think that they do that?

R: yes, because during this at natal visit the OIC and other health workers they use to teach us about those vaccines not only the Hepatitis B vaccine and other vaccines.

I: what gives you this level of confidence that you just expressed?

R: what gives me the level of confidence that…..

I: what gives you the confidence that in general that people in this area get the vaccine ?

R: the health workers in this facility are doing very well, they are working well, and all the vaccines they give to us is working, that's why I have the confidence.

I: ok, do you have any suggestions for us if we want to educate people in your community about Hepatitis B birth dose vaccination, do you have any suggestion you want to give us if we want to educate them or ways we can go about it?

R: for me o, if you want educate them no problem but the health workers are trying their best teaching us what we need to know so I don't think you need to educate us because all those vaccine they are teaching us the need for it.

I: Ok the health workers are also teaching you people?

R: yes

I: I am also asking is there any other suggestion you want to give as a pregnant mother, are there other things you may want to suggest that if we do this one it may also be good oh as a way it will educate pregnant women in this community, Onu-Ogba

R: no, for me I I don't think so, No

I: how would you prefer to learn about Hepatitis B, how would you prefer to learn it if somebody wants to teach you about Hepatitis B how would you prefer to learn it?

R: if some wants to teach me about Hepatitis B

I: Yes if someone wants to teach you, which strategy do you really understand when somebody teaches you with, which method works for you when somebody wants to teach about Hepatitis B disease:

R: hmmmm, is like if someone wants to teach me about Hepatitis B

I: yes, which one do you understand, which method would help you to understand it very well?

R: well, during that at natal care of before the at natal they used to health educate us and I think that one is more important so I think that one is better because during that time they will ask you question if

you have any problem and if you have any problem then you will throw your question and she will now treat it.

I: ok, what about through stories, videos, flyers or other methods, do you?

R: that one is included because during that time of education all this flyers now they use to give us example show us all this flyer and also pictures.

I: ok, how do you prefer to learn about things about this issues generally, how do you prefer to learn it about things about health?

R: how I prefer to learn about the health generally:

I: yes,

R: hmmmmm, through videos, through community mobilization, all this, through flyers like this now and mmm, what do they call it? Hmmmmm hmmmmm Trainings, go to trainings about that particular disease or assuming they wanted to introduce a new vaccine you go to training about that one, I think that one is good.

I: is there anything you think we should know about Hepatitis B sharing information about Hepatitis B birth dose vaccine we did not mention, is there anything you want to talk to us about that w did not mention in this discussion that you feel want to contribute.

R: No

I: thank you so much for your time

R: you are welcome

I: we really appreciate, God bless you

R: thank you and you too.

Note taking template for Health care worker KII guide State 2, LGA 3, Facility 1

Demographic information of interviewee

Age: 29

Sex: Female

Designation: Community Volunteer

Questions

1. What are the biggest challenges for people living with hepatitis B in your community?

Respondent: Well, ermmm, in this part of the community most people having Hepatitis B doesn't even, they are not aware that they do have it. But most times when we find out they kind of shy away from receiving treatment for it, so I think that is one of the challenges they do have. They shy away from the treatment and all that.

Interviewer: okay are you through, is there anything you want to tell us again?

Respondent: Mmmm, no. Like most times like ermmmm, in partners, let's assume it's the woman that was diagnosed of hepatitis B, like when you want her to bring the partner into it, she becomes afraid about the partner's reaction and all that.

2. What are the biggest needs to address hepatitis B in your community?

Respondent: Uhmmm, the needs one are, from my view, I think ermmm there should be health education, yeah, there should be health education to let the mothers know that hepatitis B is not a death sentence and it is curable it's not like and ermmmm equipment for the test should be made available in the health facilities.

Interviewer: you've talked about two things. You have talked about health education, you've talked about provision of appropriate equipment for the laboratory, what other things do you want to mention?

Respondent: And ermmmm health workers should be trained about hepatitis B testing and all that so they should know how to handle it

Interviewer: so precisely, you've mentioned health education, we've talked about appropriate equipment, we've talked training, capacity building and testing. So that is what you have?

Respondent: Yes

3a. Is there a strong need for the hepatitis B birth dose? (Why or why not?)

Respondent: Yes

Interviewer: So why do you think there is a need for Hepatitis B birth dose?

Respondent: Why I think there should be need for hepatitis B birth dose is that at least with the birth dose, it will help to reduce the risk of hepatitis B in children

Interviewer: So, do you have any other reason?

Respondent: Nnnnnn No

3b. Do others see a need for the hepatitis B birth dose?

Respondent: Yes

Interviewer: why do you think others feel that need?

Respondent: I think for that same reason to reduce the risk of hepatitis B in children and as well reduce the transmission from one patient to another.

4.Can you tell me about the current programs or practices for the hepatitis B birth dose in your clinic?

Respondent: mmmm, none that I know of.

4a. Is the hepatitis B birth dose easy to get within 24 hours of birth? (Why or why not?)

Respondent: Yes, it is easy to get

Interviewer: Why is it easy to get?

Respondent: Because the vaccine is always available in the health facility

Interviewer: what other reason again apart from availability? For something to be available, availability is one then accessing it is another.

Respondent: yes, it’s as well accessible in the health facilities.

4b. Who can administer the hepatitis B birth dose?

Respondent: I think any trained health worker can

Interviewer: Can you list?

Respondent: ermm, the CHOs, the CHEWs, the nurses, the medical doctors. I think everyone, any trained health worker can administer hepatitis B birth dose.

4c. What are challenges/barriers to getting the hep B birth dose vaccine?

Respondent: Mmmmm ermmm, timing like down here, in other to avoid vaccine wastage we normally ask patients to come back on that immunization days. So, I think that's the only challenge we have here that can hinder people getting the birth dose of Hepatitis B

Ineterviewer: So, what you are trying to say in essence if I get you right, if for instance this is weekend maybe Saturday, Sunday and ermmmm a woman came and maybe is in labor, and delivered afterwards, so that woman will not get the vaccine immediately; the hepatitis B birth dose?

Respondent: Yes

Interviewer: because so why, you want other people to come?

Respondent: Yes, I want them to come together so that they will receive it like at a larger number

Interviewer: So, what other challenges again can you tell us?

Respondent: Welll I think ermmmm, some health workers do not have the knowledge of that ermmm hepatitis B birth dose. They are not knowledgeable about it and ermm even some parents as well they are no aware that their babies are supposed to receive the hepatitis B birth dose at birth.

Interviewer: Okay, you have given us two now. One is on the side of the health care worker not having adequate knowledge about hepatitis B birth dose and the other aspect is from the caregivers. The caregivers not being informed. So, one is knowledge and the other one is awareness from the caregivers.

Respondent: Yes

Interviewer: and do you have any other aspect to talk about?

Respondent: No

5. Have you gotten information from participants regarding their experiences with the hepatitis B birth dose?

Respondent: No

Interviewer: You have not gotten any information from participants?

Respondent: Yes

5a. What is the community understanding of the hepatitis B birth dose?

Respondent: mmmm, they understand it as just one of the immunizations, ermmm normal routine immunization they are being given. That's just the way they see it.

Interviewer: so the awareness is there that they see it as part of the RI for Under one?

Respondent: Yes

Interviewer: Apart from that, no other reason?

Respondent: nothing

5b. Are people interested in getting the hepatitis B birth dose?

Respondent: Yes

5c. Can you describe what kind of specific information/messaging you have heard?

Respondent: Apart from the parent telling you that ermm the children or the newborn has received the hepatitis B vaccine, during immunization session, they as well ask you is there a vaccine that is supposed to be given to a child like 24 hour or before 24 hours or immediately after birth so with that I get to know that they are knowledgeable about it maybe they have heard about it or they have been told about it.

6. Do you have any ideas or suggestions for improving hepatitis B birth dose vaccination rates?

Respondent: Okay, just like I said before, I think there should be more awareness created both to caregivers and ermmm health workers on the importance of Hepatitis B birth dose.

Interviewer: So, you know we have sub questions under these questions. The first is do you have suggestions for improving Hepatitis B birth dose vaccination rate. So, the first one you said is a yes, right?

Respondent: Yes

Interviewer: So now, we want to look at the different levels. At the community level, what the ideas or suggestions for improving Hepatitis B birth dose.

6ai. At the community level

Respondent: At the community level, there should be ermmm awareness and health education given to the community members telling them about hepatitis B birth dose and ermmmm when it should be received and the importance of it to their babies

Interviewer: Okay, this is at the community level. Then what about the health system level?

6aii. Health care system level

Respondent: There should be training. A health worker should be trained and then ermmm, appropriate

Interviewer: we are not talking about Health care workers now, we are talking about the system, the health system like the whole ministry of health in this state is a system, Enugu state primary health care board is a system, this PHC is a system. So that system, what ideas, or suggestions?

Respondent: mmm, okay, ermm I think there still should be more awareness among the system. They should create more awareness to other systems like health education should be extended and ermm there should be training and then appropriate equipment for testing ad vaccination should be provided as well.

Interviewer: So now at the provider level, that is staff that work within this facility, they are the providers they are the ones that provide the services, so what ideas or suggestions for improving the Hep B birth dose vaccination rate at the provider level?

6aiii. Provider level

Respondent: Okay, there should be more intensive training and then appropriate equipment as well should be provided and vaccines be made available at all times so that babies could receive the Hep B birth dose.

6b. Are there specific strategies for education or awareness that might be most effective?

Respondent: No I don't know o. More training that is being done.

7. Can you tell me about your experience with the maternal tetanus vaccination?

Respondent: Mmmmm, my experience, my experience. Well, ermmmm, to some extent, Ermmm most mothers they are aware of the tetanus vaccination but there are still people who are not aware and even some of them that are aware do not take it seriously, they neglect they feel it is not that important. It doesn't really matter. That is the attitude some of the mothers give to us.

7a. What barriers or challenges have you experienced related to the maternal tetanus vaccination?

Respondent: Well, ermmm, the barriers I think I have experienced is ermmmm, how do I put it…ermmmm. Just like I said before, some mothers don't see it as a serious thing. As in they see that information as not being necessary. They don't see it as something that is important. Most times they see it as they think that there is a charge attached to it which we'll inculcate into their fees, so they just see it as a means of extorting money from them. So, some of them don't even attach so much importance to it.

Interviewer: Do you have any other thing about the barrier?

Respondent: No

7b. What successes have you experienced, what is working?

Respondent: Is it about the hep B, or the TD, or everything?

Interviewer: for this we will be looking at both the TD and also if you have any other aspect about the Hep B birth dose. So, we will pick them individually.

Respondent: well for TD, I think there is improvement already in that aspect as a result of ermmm health education being given during antenatal session. Women are actually improving on it. They are responding. Sometimes they even ask for it like on their own without you the health worker reminding them. So through health education, there is improvement.

Interviewer: for TD, do you have any other success story. Something that you applied and it yielded result?

Respondent: mmmmmm, normally, what we do, the technique we use here normally is health education

Interviewer: now we are done with TD. What about the Hep B birth dose?

Respondent: The Hep B birth dose as well is health education. What we do most here is health education we talk to them and make them see the importance of those vaccines and then they are actually responding to it.

Interviewer: specifically for the Hep B birth dose?

Respondent: yes

8. What types of health interventions have been the most successful in your community and why have they been so successful?

Respondent: I will not (chuckles) I will just be truthful. I personally have not been involved in any I don't think I have been involved in any intervention outside this facility.

Interviewer: you know this facility provides services to this community. This facility has catchment areas that makes up this community. It might not really be outside the facility; it might be within this facility. So, what intervention has this facility conducted either outside or within in form of outreaches or whatever, anything you've done?

Respondent: (exhales heavily)

Interviewer: Okay, If you can't remember, we will make progress.

9. Is there anything else you think we should know about hepatitis B, sharing information about hepatitis B, or the hepatitis B birth dose?

Respondent: No

Interviewer: So, you have given everything?

Respondent: Yes

Interviewer: We want to thank you for making out time for this Key Informant Interview. Thank you very much for your time.

Respondent: you are welcome

Interviewer: And our regards to other staff of the facility

Note taking template for Pregnant women KII guide State 2, LGA 3, Facility 1

Demographic information of interviewee

Age: 38

Questions

1. What do you know about hepatitis B?

Respondent: I don't know

Interviewer: what do you say?

Respondent: I don't know

2. What do you know about the hepatitis B birth dose?

Respondent: Yes

Interviewer: So, what do you know about it?

Respondent: At least they have given us the first dose during the 4 months and the second one

Interviewer: so, is this your first pregnancy?

Respondent: no

Interviewer: So, the previous pregnancy you had, the moment you put to bed there is this vaccine they give to children. it ought to be within the first 24 hours. The moment you put to bed they give to you and they give it to your baby and also at the same time they give BCG. Do you know what we are talking about now?

So that is what we are talking about for children. Immediately after birth. So, I wouldn't know if your children accessed it the last time immediately you put to bed and they gave you the vaccine.

So, what do you know about that vaccine?

Respondent: the little I know it helps to prevent the baby, the sickness from the baby. It helps the baby to be strong and be healthy.

2a. How do you feel about the hepatitis B birth dose?

Respondent: it is good

2b. Have you ever asked for the hepatitis B birth dose before for other children?

Respondent: they normally give them

3.Do you plan to get the hepatitis B birth dose for your baby?

Respondent: Yes

3a. Why or why not?

Respondent: for the baby to look healthy and to prevent infection

3b. Do you have any feelings of concern, stress, excitement? Why?

Respondent: It is not stressing me. I am okay with it.

Interviewer: so why are you okay with it?

Respondent: I don't feel bad on it. It doesn't have side effects on me and even on the baby.

4. Do you think the hepatitis B birth dose will work to prevent hepatitis B? (Why or why not?)

Respondent: It will that is why they give it

Interviewer: why do you think so?

Respondent: why I said so is that for the government to give that Hep B they know the importance of giving it. So, I believe it is good and is good to be taken.

5. Do you think you could easily get the hepatitis B birth dose vaccine if you asked for it?

Respondent: I cannot say

5a. Will you ask for it?

Respondent: I will if it is necessary

5b. Why do you say that?

Respondent: because of its importance

5c. Is there anything that might come in the way or challenges you might face related to accessing the birth dose?

Respondent: Except financially, financial problem. But once there is money and if it is the one I can buy, I will buy it.

6. Do you feel the hepatitis B birth dose is important for you to get for your child? (Why or why not?)

Respondent: Yes

Interviewer: why do you feel so?

Respondent: because the vaccine helps them to prevent sickness let me just put it that way

7. In general, do you think pregnant women in your region get the hepatitis B birth dose for their babies?

Respondent: Yes

7a. What gives you that level of confidence (or lack of confidence)?

Respondent: I do not have any idea

8. Do you have any suggestions for us if we want to educate people in your community about the hepatitis B birth dose?

Respondent: I will create awareness.

Interviewer: It is about you now giving us suggestions. So, what suggestion would you give to us?

Respondent: number one, you have to create awareness within the community, so they will be aware

Interviewer: So, what else again?

Respondent: nothing for now

Interviewer: Do you want to learn more about Hep B?

Respondent: Yes

Interviewer: if we want to train people now, we have several methods of training people. What methods of training would you prefer?

8a. Would you prefer to learn about hepatitis B? (Through stories, videos, flyers, other methods)

Respondent: I think stories because you stay with the person the person will be seeing you one on one. You will be telling the person and the person will equally understand you. at times you may share flier, they read, and they may not understand.

Interviewer: apart from stories, which other method of training would you prefer?

Respondent: ermmm, radio

8b. How do you prefer to learn about health issues?

Respondent: through stories, through video and radio

9. Is there anything else you think we should know about hepatitis B, sharing information about hepatitis B, or the hepatitis B birth dose?

Respondent: No

Interviewer: thank you very much for giving us audience. We appreciate and wish you the best today. Thank you.

Note taking template for Health care worker KII guide State 2, LGA 3, Facility 2

Demographic information of interviewee

Age: 45 Years

Sex: Male

Designation: Medical Doctor

Questions

1. What are the biggest challenges for people living with hepatitis B in your community?

Respondent: The biggest challenges is ignorance and poor awareness

Interviewer: Sorry sir, my colleague stepped out; we want to find out the biggest challenge of people living with Hepatitis B in your community.

Respondent: Ignorance of the infection, that's the major challenges

Interviewer: So what other challenges or that's the biggest challenges?

Respondent: Yea

2. What are the biggest needs to address hepatitis B in your community?

Respondent: The need is just to create awareness, to create awareness at the at their settlement, at their villages, the mode of transmission, then the basic symptoms and if they have those symptoms, they should present to the hospital as early as possible instead of using local medication, em, herbs.

Respondent: Alright sir.

3a. Is there a strong need for the hepatitis B birth dose? (Why or why not?)

Respondent: Of course now, it helps to prevent the infection

Interviewer: So why is there a strong need for it?

Respondent: It gives, it gives immunity to the child against the virus

3b. Do others see a need for the hepatitis B birth dose?

Respondent: Of course, in a, in a pandemic situation

Interviewer: Sorry sir, its not about you now, we want to find if others see the need for it?

Respondent: Yes, there is need for it if there is sort of outbreak of hepatitis B virus infection in the society, there is need to get people prepared by vaccination to strengthen their immune system and also to sort of give them ermm immunity for them against the infection.

Interviewer: What about people within you, within your community, do you think they see that need, people you see every day. From their behavior, their attitudes, do you think they feel that need?:

Respondent: Yes Yes

Interviewer: They show that concern?

Respondent: Yeah because since one of the mode of transmission is through sexual intercourse and em em use of unsterilized sharp objects, so the the transmission is common. Base on that people should be adviced to take a vaccine that will protect them.

Interviewer: It is just for instance now, lets rephrase it this way, for instance now you know your need, you discovered that or you see a need for this because you know it is important and because you know about the virus and you know about the vaccine, everything about the benefits/importance, then other people within the community, either the health workers or caregivers. So from their attitudes and behavior, do you think they see the need for it, this is not from your perspectives but people within the community, people you work with, do you think they see the need for the hepatitis B birth dose, that it is important

Respondent: Please repeat your question

Interviewer: Okay, it is just like when we walked in here, my colleague was putting on his masked, pulled his chair closer you wanted to be protected and we gave you a face mask, if am asked if the doctor consulting sees the need that of covid, I will say yes because the attitude he puts forward immediately we walked in and you know covid is real and can be transmitted if we do not follow those non pharmaceutical protocol. So now what im asking is people within your community (within or outside the health care system) do they see need for hepatitis B birth dose? Hope you understand?

Respondent: Yes, I understand you now, like what I said ab-initio that the major, major challenges is ignorance, it is only when you are aware of illness, aware of the problem of the health problem of the society that you will be conscious, you know preventing it from getting it to you so if there will be as such exist I believed they will be very very conscious of preventing it from them.

4.Can you tell me about the current programs or practices for the hepatitis B birth dose in your clinic?

Respondent: Yea, yea. Birth dose, it is inculcated into child’s immunization, it is part of it so from there, the birth dose is given.

4a. Is the hepatitis B birth dose easy to get within 24 hours of birth? (Why or why not?)

Respondent: Well, I have never gotten any report that they have gone short of it before, so I believed they get it when needed because I have never gotten any report that they have run out of it, do you understand.

4b. Who can administer the hepatitis B birth dose?

Respondent: An Immunization officer here does so

Interviewer: We want their designation specifically, you know we have different cadre of health workers. Some people may be health attendant working there

Respondent: No No No, those immunization officers, the trained ones, they may be a nurse, mainly here, we trained nurses for it. That’s all

4c. What are challenges/barriers to getting the hep B birth dose vaccine?

Respondent: What I have told that I have never had a report that they ran out or they find it difficult to get it, do you understand, I have not seen it as a challenge for now.

Interviewer: The hepatitis B birth dose may be available at the immunization department but people may not be able to access it because there is a difference between availability and accessibility, it may be available always but people may not be able to access it. So what do you think might be the barriers or challenges that might stop people from accessing the vaccine?

Respondent: Really barriers as I used to tell you may be as poor awareness because during their ante-natal program or period we do educate them on the important of vaccination so those pregnant mothers, they see it as their responsibility to bring out their children for vaccination so once that awareness is created within that period and reinforced in the clinic anytime they bring a child, theres a way the doctor will ask, do you immunized your child, he said yes and you ask at what age, you look at the childs age and know what the child ought to have gotten, do you understand? So that the issue there once they have that awareness and anytime they comes to clinic they reinforced it eh, reminding them so whatever they may have gotten there, they will not see it as childs play, they will see it as very important and as duty they owe their children to make sure that it is given.

5. Have you gotten information from participants regarding their experiences with the hepatitis B birth dose?

Respondent: Actually, they have not specifically say their problem on that vaccine, the only thing some will say is that after given the vaccine the child will start having fever, do you understand, they have not say that it is hepatitis B or C or whatever vaccine but on a general knowledge, some of them will tell you that this fever started after receiving vaccine.

5a. What is the community understanding of the hepatitis B birth dose?

Respondent: Mothers they don’t have such deep understanding on it, they don’t have such understanding

Interviewer: They don’t have deep understanding on the vaccine?

Respondent: Yes, the only thing is that when you tell them about ermmm childs immunization, when you mention hepatitis B, they will hear then, do you understand but I don’t think there have been a situation yet except in some cases where like the world health day on hepatitis, the ministry of health is doing health awareness through radio, jingles, different this thing so for those thatb have access to radio in their villages they can get the knowledge of it because the ministry do give enough jingles on it.

5b. Are people interested in getting the hepatitis B birth dose?

Respondent: Yeah, people are very very interested in the vaccine for their children, during the children immunization day they are very very interested

Interviewer: Im talking about hepatitis birth dose, not just other or the whole RI immunization, not just other antigen but specifically the birth dose

Respondent: yes, they don’t discriminate, they don’t say don’t give this one, give this one

Interviewer: It is a general acceptance?

Respondent: Yes

5c. Can you describe what kind of specific information/messaging you have heard?

Respondent: The message we give to them or the mothers?

Interviewer: Yes, (Repeats question)

Respondent: First of all, we let them know theres something like Hepatitis B

Interviewer: No, the one you have heard

Respondent: The one I heard

Interviewer: Lets say you are aware, informed about hep B infection and the vaccine, so but this group of people outside, what kind of message they give as feedback?

Respondent Oh, most of them are scared of it, when they heard that somebody ermm has hepatitis B virus, they think there world has ended, that’s the issue, they think that their world has ended.

6. Do you have any ideas or suggestions for improving hepatitis B birth dose vaccination rates?

Respondent: What I may suggest is this; a lot of pregnant women go for ante natal but not all of them. Secondly, not all of them go to government centers where those things are supposed to be available, some end up in the private sector, some end up in the Traditional Birth Attendants (TBAs) area, some don’t even go, they deliver at home. So for those ones that are not have access to government establishment, most of them, they don’t usually hear it. So my suggestion is this, if organization can extends their their their help, maybe organize those traditional or although we discourage women from going to the TBAs but still some of them do go there. If we can incorporate them, give them a minimal knowledge especially on referrals over than may have gone there sometimes they may have call it ante natal or not. Should at the end of the day, advice the woman, please you still need to visit the so so so center, the government center that is supposed to have that such vaccine to visit this centers please for the sake of your child, I know you have delivered safely many children. So what I have come to say the organization need to extend their hands not to restrict the training or awareness to those working in the government establishment, if it can be extended to non-governmental areas like private clinics, emm, TBAs, erm give them their training. It is not that, im not advocating for them to be given the vaccines to administer but let them have the awareness so that they will be able to bring more children into the program.

Interviewer: so this question is divided into 3 parts, so the question should be (repeat the question), we want to break it at the community level, so what strategy do you use at the community levels?

6ai. At the community level

Respondent: At the community level, it is to create awareness about TBAs

6aii. Health care system level

Respondent: Awareness and training for the personnel, training the personnel, giving them a useful information about it so that some of them will not scare their patients away. Some of them when they go to the lab and they are told they have hepatitis B, some will say madam im sorry as if to say that the world of that woman has ended.

6aiii. Provider level

Respondent: What we usually do for example when a patient comes maybe with a result of hepatitis B positive, we educate the patient, we tell the patient, give them a brief knowledge of it, how to prevent it and what and what the person should be doing other to reduce the severity or to prevent the mortality of already existing infection then and advice the person on the treatment. Refer the patient to the appropriate center where the patient will get the treatment like here in the general hospital, we don't have the facility, we don't have the drugs for it so what we do, we refer them to the teaching hospital, either parklane or UNTH and that they erm erm encouraged the person tell that it is not a death sentence that the patient will definitely get well, however also advice the patient not to spread giving them the guidelines if it is like in adult, you tell them not to share object with their fellow, sexually active individuals, tell them to prevent or they should be using barrier method because they may think let me infect Okey since Okey is the one that gave me something not knowing Okey may have HIV or when in your back of your mind you are thinking of infecting him with erm hepatitis B virus but getting additional one from what you have. So we are advise them to use barrier method if they must engage in sexual activities so that's it.

6b. Are there specific strategies for education or awareness that might be most effective?

Respondent: The strategies, yea, the strategies we might be using, may be using pictorial presentations, drama in some cases, drama in some cases then pictorial presentations. That's all. Because those ones will make them to be interested and listen.

7. Can you tell me about your experience with the maternal tetanus vaccination?

Respondent: TT, okay, TT yeah

Interviewer: So we want to hear brief experiences

Respondent: The experiences we have are TT in children has reduced drastically

Interviewer: Here, we are concentrating on maternal tetanus vaccination

Respondent: Yeah, the mothers, yea, they take it. They don't reject it, they are aware of it, I have not seen any mother, any pregnant woman turning down tetanus injection, I have not seen any of them, however whenever we see a would we treat aggressively but I have never seen any of them having tetanus infection

7a. What barriers or challenges have you experienced related to the maternal tetanus vaccination?

Respondent: I believe it always available because I have never gotten any report on it that it is available. Accessibility depends on the hospital like for us here we have enough health center close to each other except in the rural areas, some of us we still have this centers close to them, so they access it, the barrier I may say among people in the rural in having access to those centre maybe because of bad roads or the

means of getting to that place then poverty is another problem because poverty has created a lot of problem. They have phobia of going to hospital, they prefers their neighbor maybe who is a TBA at the end of the day the money they are spending may be much more higher than what they are supposed to spend in the hospital, so phobia is another problem they are having. Fear of going to hospital because of poverty because of financial difficulty

7b. What successes have you experienced, what is working?

Respondent: Like maternal tetanus vaccination, I have never seen a mother

Interviewer: We are looking at the successes, good practice, best practice that works for you

Respondent: I don’t understand the question very well, I think

Interviewer: Actually if the vaccination rate for the hepatitis birth dose is high and the maternal tetanus vaccination, if the rates are very high within you community, so we want to know what are your successes, what you are doing differently to be achieving these, what successes you have experienced?

Respondent: Well one of it is we try to be friendly to them the mothers, we try to create enabling environment for them, so based on that whenever we are telling them or whatso ever messages we are giving to them, they see it as it is coming from their friends and or their brother or their relations, do you understand. They try to follow the right way of what we tell them.

8. What types of health interventions have been the most successful in your community and why have they been so successful?

Respondent: The intervention that usually gives us ere r good result is adequate of awareness, when you create awareness you get results because nobody wants to die, if you explain to them and prescribe and tell them the importance of what ere r what you want to give to them or the services you are providing to them, they will come for it. So awareness is one of the major strategy I have seen that works very well.

9. Is there anything else you think we should know about hepatitis B, sharing information about hepatitis B, or the hepatitis B birth dose?

Respondent: Yes, treatment of hepatitis B infection is not as available as I think it is supposed to be, because the medication for it are not as usually accessible, very costly, yea, yea yea, it is very very costly and it is not accessible so some of them always when we get them, they try to encourage them because of the poverty nature of the people when they go to private institutions, the physician that knows how to treat it gives them the bill to pay for it, they will run away. Some prefer carrying their infection and wait for the consequences so if there will be a way of bringing the cost of the treatment down and making the materials available, and referrals available and so many other drugs used in the treatment, I think it will go a long way to help some individuals. For those having the disease itself, for the birth dose, we keep encouraging cos prevention is far better than cure so it is better prevented than waiting for when it comes down with the infection at that time the cost will be high. Base on that it is better they received they

receive the vaccines when it is been given free of charge than waiting for the infection to come into their system they will go and start selling their property to prevent that they could have prevented free of charge. Do you understand, some of them do come up with it and follow up with the laid down procedures for the birth dose.

Note taking template for Pregnant women KII guide State 2, LGA 3, Facility 2

Demographic information of interviewee

Age: 20

Questions

1. What do you know about hepatitis B?

Respondent: No, I have heard of it but I don't know what it is all about

Interviewer: So what did they tell you about it?

Respondent: I think they say ehm, I think they say it is a disease that occur maybe in women and in men

2. What do you know about the hepatitis B birth dose?

Respondent: I don't know about it

2a. How do you feel about the hepatitis B birth dose?

Respondent: I don't feel anything

2b. Have you ever asked for the hepatitis B birth dose before for other children?

Respondent: It is only if the nurse gave it to me, then I will take it

3.Do you plan to get the hepatitis B birth dose for your baby?

Respondent: Yes

3a. Why or why not?

Respondent: So that if, if the vaccine is for prevention of disease or anything, I will like to get it so that it will prevent my child from contacting any infection

3b. Do you have any feelings of concern, stress, excitement? Why?

Respondent: Is it the vaccine? No

Interviewer: So why don't you have?

Respondent: I don't really know what the vaccine is all about, had it been I know what the vaccine is all about then I will tell you if I have concern

4. Do you think the hepatitis B birth dose will work to prevent hepatitis B? (Why or why not?)

Respondent: Sure

Interviewer: So why do you think so?

Respondent: Because it is a medicine

5. Do you think you could easily get the hepatitis B birth dose vaccine if you asked for it?

Respondent: Yes

5a. Will you ask for it?

Respondent: Yes

5b. Why do you say that?

Respondent: The reason is just because, like I said it may be safe. It is a vaccine given to children after birth so I will like to get one for my child to prevent them from having hepatitis

5c. Is there anything that might come in the way or challenges you might face related to accessing the birth dose?

Respondent: I did not get the question

Interviewer: Question was repeated

Respondent: I don't think there will be any problem except if maybe they don't want to give the vaccine but for now I don't think there is any challenge that I will have

6. Do you feel the hepatitis B birth dose is important for you to get for your child? (Why or why not?)

Respondent: yes

Interviewer: Why?

Respondent: Because it is good for my child to help my child to grow

7. In general, do you think pregnant women in your region get the hepatitis B birth dose for their babies?

Respondent: Yea, I think so, everybody wants the best thing for the child

7a. What gives you that level of confidence (or lack of confidence)?

Respondent: Because no parents will want his or her child to suffer from any illness so that's my reason

8. Do you have any suggestions for us if we want to educate people in your community about the hepatitis B birth dose?

Respondent: Yes, the advice is just for you to go to any village like that that don't have that knowledge in their area, that all I can say oo

8a. Would you prefer to learn about hepatitis B? (Through stories, videos, flyers, other methods)

Respondent: Yes

Interviewer: I think videos will be the first idea for me then second, storytelling. Both stories and videos, so if you watch the video very well you will be able to learn more other than listening orally

8b. How do you prefer to learn about health issues?

Respondent: If the government or if the government can provide teachers or people that will educate others based on based on the vaccine it will be better or even if carrying out a research or carrying out based on vaccine we will be able to learn more about it.

9. Is there anything else you think we should know about hepatitis B, sharing information about hepatitis B, or the hepatitis B birth dose?

Respondent: I don’t know much, I don’t know much about it so I don’t have anything else.

Note taking template for Health care worker KII guide State 2, LGA 3, Facility 4

Demographic information of interviewee

Age:52

Sex: female

Designation: Midwife

Questions

1.Interviewer: What are the biggest challenge for people living with hepatitis B in your community? Your community is together with that the health center that you serve. What are the biggest challenge for people living with hepatitis B in your community

Respondent: The biggest challenges to people that are living with hepatitis B in this community is emm, the finance, the money to buy drugs, the place to treat them.

2. Interviewer: What are the biggest needs to address hepatitis B in your community?

Respondent: The first of all is to prevent it, to immunized, against hepatitis B, then to train, give information about the causes. Make people know about the causes of hepatitis B so that they would not get it. The next one is to when the the disease comes to assist them by treating them and giving them other things that they need.

3. Interviewer: Is there a strong need for the hepatitis B birth dose? (Why or why not?)

Respondents: I don't understand that one.

Interviewer; Do people need hepatitis B birth dose?

Respondent: Yes, there is a need for it in the community.

Interviewer: why do you think that there is need for Hepatitis B birth dose?

Respondents: There is need for hepatitis B birth dose so that you prevent the disease from coming.

3b. Interviewer: Apart from you, do others see a need for the hepatitis B birth dose?

Respondents: yes. Others see a need because we do tell them the need..

4. Interviewer: Can you tell me about the current programs or practices for the hepatitis B birth dose in your clinic?

Respondents: Immediately a woman gives birth, even if it is not on immunization days. I tell them, give because we have hepatitis B in the solar. So it is always here, so they do give immediately they give birth.

4a. Interviewer: Is the hepatitis B birth dose easy to get within 24 hours of birth? (Why or why not?)

Respondents: Yes

Interviewer: why is it easy to get?

Respondent: We have it here in the clinic and we have solar where we keep them.

4b. Interviewer: Who can administer the hepatitis B birth dose?

Respondents: Everybody.

Interviewer: I want you to list their designation

Respondents: laughs, hmm starting from everybody that is here. Coming to health, nurses can administer it. Doctor can administer it, junior communitant worker can administer it. Senior workers can administer it. Even the volunteer health workers that are here, they do administer hepatitis B.

4c. Interviewer: What are challenges/barriers to getting the hep B birth dose vaccine? Some people are not able to get them

Respondents: Challenges is that maybe their facility is far from the health local Govt where they do get the vaccine. Maybe they do not have equipment to keep the vaccine

Interviewer: This two now is a different question entirely. Most care givers dont bring their new born babies to get the vaccine. What are the challenges? They cant access the facilities

Respondents: Ignorance. Ignorance that is the major challenge. If they know but now this present day, people are aware and they do come for it.

5. Interviewer: Have you gotten information from participants regarding their experiences with the hepatitis B birth dose? Care givers, mothers have they given you their experiences about Hepatitis B birth dose vaccine?

Respondents: I have not gotten any experiences

Interviewer: Just like maybe women now bringing their child after taking the vaccine have they given you feedbacks after taken the vaccine?

Respondents: Some said that their children child started running fever. Some said that the place is red. Some said that their child cried throughout the night. But I tell them maybe it is because of the pain.

5a. Interviewer: What is the community understanding of the hepatitis B birth dose? You have been in this community for a long while. What do the understand? What is their understanding?

Respondents: they know that it is needful. There is need for them to take it. They know that hepatitis B is deadly disease and that they need to be protected so beacause of that they do come to take it often.

5b. Interviewer: Are people interested in getting the hepatitis B birth dose?

Respondents: Yes, even the adults do come here sometimes, to ask us to give them

5c. Interviewer: Can you describe what kind of specific information/messaging you have heard?

Respondents: I do not understand that question

Interviewer: We ask what is the community understanding? what do you know? Can you describe what kind of specific information that you have?

Respondent: I don't understand

Interviewer: Recasts question

Respondents: I know that hepatitis b is a deadly disease. I know that hepatitis B is a deadly disease. One needs to be immunized against it and try as much as possible to prevent getting the disease.

6. Interviewer: Do you have any ideas or suggestions for improving hepatitis B birth dose vaccination rates?

Respondents: I will suggest that people should be trained. The information should be given, especially to the traditional birth attendant. People do go there. Some women go there to deliver and stay for more than three weeks before they come here to take the zero dose. Even some private hospitals, they should be given… they should know about it.

6ai Interviewer: This question you will tell us at the community level. What are the suggestion?

Respondents: Making people to be aware of the disease and telling them the importance of taking the vaccination at the birth immediately.

6aii. Interviewer: Health care system level

Respondents: It is what we are doing here. Immediately you give birth, give that vaccine. Do not wait until immunization day because anything can happen.

6aiii. Interviewer: Provider level

Respondents: Is it for us or is it for new born babies?

Interviewer: What kind of ideas or suggestion you give for hepatitis B birth dose vaccination rate among the health workers?

Respondents; I have already said that, the suggestion is to give the child the vaccination immediately the child comes out when you give birth

Interviewer: so what do you think we can do to health care workers after we have carried out this to improve the vaccination rate?

Respondents: to give them the information and make sure that they carry it out.

6b. Interviewer: Are there specific strategies for education or awareness that might be most effective?

Respondents. Is it in this facility?

Interviewer: it might be within this facility and within this community?

Respondents: mobilization. call people to tell them talk about hepatitis B and the birth dose.

7. Interviewer: Can you tell me about your experience with the maternal tetanus vaccination?

Respondents: is it in the health facility or personal experiences

Interviewer: the experience that you have heard whether in the health facility or personal ?

Respondents: I know that people are aware of the importance of tetanus toxin, even those people that are not coming for ante natal, they go for other places but they come here. They know that we have tetanus toxide. Women are aware of it now. Even those that are not pregnant they do come her for tetanus toxide.

7a. Interviewer: What barriers or challenges have you experienced related to the maternal tetanus vaccination?

Respondents: There hasn't been so much barriers.

Interviewer: That will make the vaccination rate to be low?

Respondents: if there is out of stock

Interviewer: You have experienced that?

Respondents: Yes. but not in recent times

7b. Interviewer: What successes have you experienced, that is what is working?

Respondents. Generally?

Interviewer: yes

Respondent. Is it generally concerning other vaccines?

Interviewer: everything in health

Respondents; everything in health. Generally, the everything is working very well. It is only that we are not been paid well. Before we do, before now, we do go to the health local Govt to bring vaccines everyday we have immunization. But now they have provided this thing solar,

Interviewer: okay

Respondent: so because the solar is here, at the beginning of the month, we do bring all the vaccines we need for the month. So most often we do not run out of stock. And another thing is that people are aware of all the vaccines. They know the importance. And there was a time we used to go for home visit telling people about these things but people now comes here so there is a lot of improvement. Even sometimes, we run out of cards. We use sheet, ordinary sheets to be writing for mothers. But these days we have cards immunization cards

8. Interviewer: So what types of health interventions have been the most successful in your community and why have they been so successful? We have two questions here

Respondents: I don't understand the activities you are talking here

Interviewer: you know. We implement activities in lower level. Most of the activities planned in the higher level is implemented in the lower level. Which of these activities is successful?

Respondents: that is successful

Interviewer: and why is it successful?

Respondents. Is there anything that is successful in this community? I will say immunization. NIPDS.

Interviewer: NIPDS? So why was it successful?

Respondents: I will say. People are aware of it and they corporate and it is successful because Polio has been eradicated.

Interviewer: Okay

Respondent: hmm

9. Interviewer: Is there anything else you think we should know about hepatitis B, sharing information about hepatitis B, or the hepatitis B birth dose?

Respondents: emmm is it to improve?

Interviewer: anything at all

Respondent: okay to improve hepatitis B birth dose…. There should be training and re training of health workers and other… is it only in this community?

Interviewer: in your community. Anything you know that you want to tell us

Respondent: hmmm. Like I said before, like I said that most women go to deliver in the traditional birth attendants, some women go there to deliver and those women they do not know about hepatitis B so they should be trained. Even if you retrain them

Interviewer: Thank you so much for granting us audience and everything you do in this facility. Thank you very much.

Note taking template for Pregnant women KII guide State 2, LGA 3, Facility 4

Demographic information of interviewee.

Age:27

Questions

1. Interviewer: What do you know about hepatitis B?

Respondent: it is an injection that is been injected to a baby with 24 hours of birth.

Interviewer: The first one I asked is about the virus, what do you know about hepatitis B infection?

Respondent: it is an infection gotten though human contact and through heat

2. Interviewer: What do you know about the hepatitis B birth dose?

Respondent: It is given to a baby or child at birth

2a. Interviewer: How do you feel about the hepatitis B birth dose?

Respondent: Nothing

2b. Interviewer: Have you ever asked for the hepatitis B birth dose before for other children?

Respondents: No

3. Interviewer: Do you plan to get the hepatitis B birth dose for your baby?

Respondent: Yes

3a. Interviewer: Why or why not?

Respondents: I heard it is good for the baby.

3b. Interviewer: Do you have any feelings of concern, stress, excitement? Why?

Respondents: No feelings

Interviewer: So why don't you have feelings?

Respondents: I don't know much about it sha.

4. Interviewer: Do you think the hepatitis B birth dose will work to prevent hepatitis B? (Why or why not?)

Respondents: Since they said it is good for the baby I think it is good

5. Interviewer: Do you think you could easily get the hepatitis B birth dose vaccine if you asked for it?

Respondents: I don’t know

5a. Interviewer: Will you ask for it?

Respondents: Yes

5b. Interviewer: Why do you say that?

Respondent: Because I heard it is good

5c. Interviewer: Is there anything that might come in the way or challenges you might face related to accessing the birth dose?

Respondent: Any problem like?

Interviewer: Maybe you want to bring your baby from coming to take the vaccine and the problem will stop you to give your child the vaccine?

Respondent: No

6. Interviewer: Do you feel the hepatitis B birth dose is important for you to get for your child? (Why or why not?)

Respondent: Yes

7. Interviewer: In general, do you think pregnant women in your region get the hepatitis B birth dose for their babies?

Respondents: I don’t know if they get it because I am not with them.

7a. Interviewer: What gives you that level of confidence (or lack of confidence)?

Respondent: I don’t really know much about it so I am not so saying I am 100% confident about it

8. interviewer: Do you have any suggestions for us if we want to educate people in your community about the hepatitis B birth dose?

Respondents: For me I will say that if you people will come to this health center during ante natal. When the women will be around because if you say they should come out in the market square some might not have the time but they will have the time to come for ante natal. I think that during that time that it will be okay.

Interviewer: but we will want everybody whether you are pregnant or not. So that some people may not be pregnant now so we wont see them at ante natal, some people don’t come to the

facility. We want women and their husband to hear. What advice will you give to us within the community?

Respondent: You tell the people so that people will go to a hall where everybody will come and tell them.

8a. Interviewer: Would you prefer to learn about hepatitis B? (Through stories, videos, flyers, other methods)

Respondents: For me any of them is good

Interviewer: which one is better?

Respondents: fliers

Interviewers: Apart from fliers is there any other one that is good?

Respondents: telling the stories

8b. Interviewer: How do you prefer to learn about health issues?

Respondents: Video

9. Interviewer: Is there anything else you think we should know about hepatitis B, sharing information about hepatitis B, or the hepatitis B birth dose?

Respondents: No

Note taking template for pregnant women KII guide: Igbo translation State 2, LGA 3, Facility 7

Ozi gbasara igwe mmadu nke onye a n’agba ajuju onu.; New Heaven health center, Enugu North.

Afo: Afo 30

Ajuju

1.Oju ajuju: Kedu ihe ị maara gbasara oria imeju a n’akpo ịba ọcha n’anya?

Osa ajuju: Eeee. Anurum ya. Anu tu lam ya.

Oju ajuju: Inu tu la ya mbu?

Osa ajuju: yes sir?

2.Oju ajuju: Kedu ihe ị maara gbasara ọgwụ mgbochi ịba ọcha n'anya

Osa ajuju: Ihe na adi nma na ya like ogwu mgbochi, I gba nwa gi ogwu mgboch ofuma, onweghi ike inwe nghari ma oburu na igbaa ya ogwu mgbochi out okwesiri. Oya ahu oma survive. Mana I treat ya ofuma onweghi anything o ga eme ya.

2a. Oju ajuju: Olee otú obi dị gị maka ọgwụ mgbochi ịba ọcha n'anya?

Osa ajuju: Obi dim uto. Obi dim uto because umuakam nile mu na agabaya and onweghi onye ha na aya odika oyaaa ahu

Oju ajuju: so umu gi nile I na amu I gbara ha ogwu mgbochi?

Osa ajuju: Eeee. Agazurumm hacha.

2b. Oju ajuju: O nwetụla mgbe ị rịọrọ ka agbaa ụmụ gị ndị ọzọ ọgwụ ịba ọcha n'anya?

Osa ajuju: Mbaa

3.Oju ajuju: i nwere atụmatụ ọ bụla iji nweta ọgwụ mgbochi ịba ọcha n'anya maka nwa gị?

Osa ajuju: Eeeeeee. Ihem ga eji gbaya ogwu mgbochi bu ka oria ocha na anya hapu I gbuya

3a. Oju ajuju: Gini mere ma obu gini kpatara ya?

Osa ajuju: Ihem ga eji gbaya ogwu mgbochi bu ka o gbochie ya nria nria a ka o ghara ime ya.

3b. Oju ajuju: i nwere mmetụta ọ bụla gbasara nchegbu, nrụgide, obi ụtọ? N'ihi?

Osa ajuju:

4. Oju ajuju: I chere na i gba ogwu mgbochi oria ịba ọcha n'anya ga-arụ ọrụ ma o buru na agba ya nwa a muru ohuru? (Gini mere ma obu gini kpatara ya)?

Osa ajuju: Eeee. Echem na o ga aru oru oma

Oju ajuju: Gini mere I ji che na o ga aru oru oma?

Osa ajuju: Ihe mu ji chee na oga aru oru oma bu na ogwu mgbochi obula nwere ihe o na aru na ahu umutakiri. O na egbochi oria di iche iche like aruobara, afo osisa onyu nyu na ogbogbo, mana ihe ona egochi kariri

Oju ajuju: o nwere ike ime umutakiri aria oria ma owu ike na agwu ha?

Osa ajuju: ihe mere bu naa I gba umuntakiri ogwu mgboch ahu na eshi ha ike mana umutakiri na agbaghi ogwu mgbochi nay a aya ahu too much. Mana I gba ha ogwu mgboch ahu ahu na eshi ha ike too much.

5. Oju ajuju: Ichere na ị ga - enweta ọgwụ mgbochi ịba ọcha n'anya B n'ụzọ dị mfe ma ọ bụrụ na ị chọọ ya?

Osa ajuju: Eeeee. Yes. Yes sir.

5a. Oju ajuju: Iga-arịọ maka ya?

Osa ajuju: Mbaa. Hana asi ka agba nwa gi

5b. Oju ajuju: Gịnị mere i ji kwuo otu a?

Osa ajuju: Mu na asi ka ha gba ya nihi ihem ji si ka ha gba ya bu na almost na agbahuya agaghi agba ya ozo.

5c. Oju ajuju: Onwere ihe obula nke puru ibia n'uzo ma obu nsogbu ndi ozo nwere ike igbochi gi i nweta ogwu mgbochi iba ocha n'anya nke a n'agba nwa a muru ohuru?

Osa ajuju: Mbanu. Onweghi ihe obula ga egbochim ya.

Oju ajuju: O di gi ka igba nwa gi ogwu mgbochi iba ocha n’anya B di mkpa? Gini kpatara I ji kwuo otua?

Osa ajuju: Yes sir. Ihem ji che na odi nma bu na o na egbochi oria ocha na anya oma mee nwa ahu oburu godi na o mewere ya o ma rue zuo ole ahu o kwesiri iru.

6.Oju ajuju: Na mkpokọta, ị chere na ụmụ nwanyị dị ime nọ na mpaghara gị ga-enweta ọgwụ mgbochi ịba ọcha n'anya B maka ụmụ ha?

Osa ajuju: ihe mere bu naa, echem na ha na agba ya.

7a. Oju ajuju: Kedu ihe na-enye gị obi ike ahụ (ma ọ bụ enweghị ntụkwasị obi)?

Osa ajuju: Neigbourm sim a ana agba nwa ha ogwu mgbochiaa

8.Oju ajuju: inwere aro ọ bụla i enye anyị ma ọ bụrụ na anyị chọrọ ịkụziri ndị mmadụ n'obodo gị gbasara ọgwụ mgochi ịba ọcha n'anya nke a n’agba nwa a muru ohuru

Osa ajuju: o like ndumodu otu aga esi mee umuakaa, igba ha ogwu mgbochia out okwesiri.

8a. Oju ajuju: Ọ ga-amasị gị ịmụ gbasara ịba ọcha n'anya (Site na akụkọ, vidiyo, akwụkwọ ọkwa, ụzọ ndị ọzọ)?

Osa ajuju: O ka nma ka etinye ya na radio. Mana video di kwa nmam oo. Ihe video ji ka nma bun a onye obula ga na afu ya.

Oju ajuju: gini mere I ji si a video ka nma

Osa ajuju: Ihe o ji ka nma na radio bun a anyi bun di igbo like ihe ana afu na anya

8b. Oju ajuju: Kedu otu kachasị amasị gị ịmụ gbasara nsogbu ahụike?

Osa ajuju: Oka mma igbasa ya na radio. Ije one by one na egbu oge. Mana I na ekwu ya na radio na eje oge

Oju ajuju: o digi ka obu mmadu nile na ege radio. I nwere ike ikwu ya na ututu oughi mmadu nile na anu ya.

Osa ajuju: ihe I nwere ike ime ya bu oburu na o buru na church, o nwere ihe o na abu na village anyi, chrch now ndi health na aga na church na agbasa ozi . ihe na eme na church buru na I no ghi ya, oburu na nwa gi no ya, o ga agwa gi ya. Ma obu teacher na school nwere ike ino nuta ya.

9. Oju ajuju: Ọ nwere ihe ọzọ ị chere na anyị kwesịrị ịma gbasara ịba ọcha n'anya, ịkekọrịta ozi gbasara ịba ọcha n'anya , ma ọ bụ usoro ịgba nwa a muru ohuru ogwu mgbochi ịba ọcha n'anya ?

Osa ajuju: O nweghi ihe odo

Note taking template for Health care worker KII guide State 2, LGA 3, Facility 7

Demographic information of interviewee

Age: 43

Sex: Female

Designation: RI Provider

Questions

1.Interviewer: What are the biggest challenges for people living with hepatitis B in your community?

Respondent: is it vaccine or infection?

Interviewer: Infection

Respondent: death now. The biggest challenge is it can lead to death. Isolation

Interviewer: Other challenges?

Respondent: No

2. Interviewer: What are the biggest needs to address hepatitis B in your community?

Respondent: Vaccination

Interviewer: you mean vaccination is the biggest need?

Respondent: Yes

3a. Interviewer: Is there a strong need for the hepatitis B birth dose? (Why or why not?)

Respondent: Yes

Interviewer: why do you think there is a strong need?

Respondents: To prevent the hepatitis B infection

3b. Interviewer: Do others see a need for the hepatitis B birth dose?

Respondents: Yes

4. Interviewer: Can you tell me about the current programs or practices for the hepatitis B birth dose in your clinic?

Respondents: hmm, how we are doing it?

Interviewer: just tell us current programs or practices in your clinic?

Respondents: We are practicing in all the days of immunization even self at birth, if a woman gave birth, immediately the woman gave birth then we give the hepatitis B immediately.

Interviewer: So any other practice or program?

Respondents: if the woman birth in another facility, maybe the facility that is not providing RI then if the woman comes to our clinic, we give the hepatitis B if it is within the twenty-four hours of the to a week interval.

Interviewer: So what you are trying to tell us is irrespective of where the woman delivers whether within your facility or outside your facility that you administer the vaccine?

Respondents: yes

4a. Interviewer: Is the hepatitis B birth dose easy to get within 24 hours of birth? (Why or why not?)

Respondents:

4b. interviewer: Who can administer the hepatitis B birth dose?

Respondents: heath workers

Interviewer: what categorizes of health workers?

Respondents; Jchew, Chews and cho and nurses

4c. What are challenges/barriers to getting the hep B birth dose vaccine?

Respondents: we are having vaccines all the times so we can say that we don't have any challenge for now

Interviewer: It may not be all about you now. We cant assume that every woman bring their kids for vaccination so what do you think are their challenges?

Respondents: if the person give birth in another facility that is not giving the RI maybe before they will come it will be above the time of the vaccine

5. Have you gotten information from participants regarding their experiences with the hepatitis B birth dose?

Respondents: Yes I don't get the question

Interviewer: Regarding their experiences. If I access the vaccine now for my child as a mother, so have I related my experiences to you?

Respondent: maybe AFI no.

5a. Interviewer: What is the community understanding of the hepatitis B birth dose?

Respondents: It is very very important for to prevent the hepatitis B health infections

5b. Interviewer: Are people interested in getting the hepatitis B birth dose?

Respondents: Yes

5c. interviewer: Can you describe what kind of specific information/messaging you have heard?

Respondents: we have town announcers and theee town announcer or community mobiliser. Then once in a while they will go and do announcement for the vaccine.

Interviewer: what do they say about the vaccine?

Respondents: They say that they have heard about the vaccine that we are introducing and they are coming to take it.

6. Interviewer: Do you have any ideas or suggestions for improving hepatitis B birth dose vaccination rates?

Respondents: Community involvement. Through town announcer

6ai. Interviewer: At the community level?

Respondents: community involvement. They will now involve the community about the vaccine then through town announcer and other means.

Interviewer: Do you have anything again at this community level

Respondent: we go home visit

Interviewer: home visit to vaccinate?

Respondent: no.

Interviewer? What do you mean by home visit?

Respondents: home visit. We just invite them then go to home to home visit then to tell them that come and take the vaccine so so and so and all what it. We are given the health talk to the pregnant mother when they come for ante natal. Then we give health talk

6aii. Interviewer: Health care system level?

Respondents: To employ workers

Interviewer: what kind of workers?

Respondents: Community health workers. more health workers to employ more workers so that and materials or equipment or Vit a. that ones is already around for so we don't have any problem

6aiii. Interviewer: Provider level

Respondent: We give health talk, we give health about Hepatitis B vaccine

Interviewer: what else again?

Respondents: any other way you can do it

6b. Interviewer: Are there specific strategies for education or awareness that might be most effective?

Respondents: we can use role play

Interviewer: okay. what else again

Respondents: fliers, posters and I think it will help

7. interviewer: Can you tell me about your experience with the maternal tetanus vaccination?

Respondents: experience how?

Interviewer: experience you have had with the maternal tetanus vaccination especially with women coming to access it?

Respondents: they are taking it very well.

7a. Interviewers: What barriers or challenges have you experienced related to the maternal tetanus vaccination?

Respondents: some women are just afraid of injections that is the only barrier but if we explain to them and tell them the reason why they are to supposed to take the vaccine and then they accepted it

Interviewer: Apart from fear, is there any other reason?

Respondent : No

Interviewer: so fear is the major barrier?

Respondents. yes

Interviewer: so if that fear is off, everybody will come and take it?

Respondents: Yes because they know the importance because we have explained it to them

Interviewer: I am imagining if fear is the major challenge is there any other challenge?

Respondents: sometimes, the women forgot. there will forgot. The date that we gave them some people will not remember it.

7b. Interviewer: What successes have you experienced, what is working?

Respondents: I don't know how to answer that one

Interviewer: recasts question

Respondents: health talk. We have given them health talk. Some people will say, hey that it will pain me and I will show them this person, this neo natal tetanus injection pictures of people immunized. That is the reason that we want to give you injection.

8. Interviewer: What types of health interventions have been the most successful in your community and why have they been so successful?

Respondents: BCG immunization

Interviewer: what did you do in that BCG? why was it so successful?

Respondents: even if the mother delivers in another place, they must come to take that BCG

Interviewer: why do you think that they come to take it?

Respondents: To prevent that infection they are afraid of it more than every other thing

9. interviewer: Is there anything else you think we should know about hepatitis B, sharing information about hepatitis B, or the hepatitis B birth dose?

Respondent: I don't have any other thing

mNote taking template for Health care worker KII guide State 2, LGA 3, Facility 9

Demographic information of interviewee

Age: 54

Sex: Female

Designation: HF Officer in Charge

Questions

1. What are the biggest challenges for people living with hepatitis B in your community?

Respondent: Hep B is a viral Infection. Mosquito can transfer it from person to person and if you look to our environment, it is very bad. It is very easy for transfer because mosquito is here around us breeding.

Interviewer: So, what are the challenges people are having? The barriers, the obstacles, the gaps the difficulties, people living with hep B in your community. Like now, if somebody is infected with Hep B virus, what are the problems they face in this community?

Respondent: The community will face ermmm a very big problem because it is transferred from person to person by an infected mosquito and our environment is bushy so they can even breed fast

2. What are the biggest needs to address hepatitis B in your community?

Respondent: The biggest needs to do what?

Interviewer: the biggest needs in addressing, if we want to solve Hep B virus in your community, what is the biggest, the solution, the biggest need?

Respondent: The solution is to help us to clear this environment. Our environment is very bad. It is very very bad.

3a. Is there a strong need for the hepatitis B birth dose? (Why or why not?)

Respondent: Yes.

Interviewer: so why is there a strong need? Why do you think so?

Respondent: Because to protect the ne born baby from Hep B virus

3b. Do others see a need for the hepatitis B birth dose?

Respondent: they see it

4.Can you tell me about the current programs or practices for the hepatitis B birth dose in your clinic?

Respondent: The practice is to tell the mothers the importance of giving their children Hep B at birth during the antenatal clinic. It is very very necessary.

4a. Is the hepatitis B birth dose easy to get within 24 hours of birth? (Why or why not?)

Respondent: Yes

Interviewer: Why is it easy to get? We want to find out if it is easy to get that is within 24 hours if anybody put to bed, they can get it easily. You said it is easy.

Respondent: to get the vaccine?

Interviewer: Yes, to be vaccinated with the vaccine

Respondent: Yes naa

Interviewer: so why is it easy

Respondent: It is very easy because they told us we can give the child Hep B vaccine before 24 ours

Interviewer: any other reason?

Respondent: and we have the solar where we kept it

Interviewer: Any other reason?

4b. Who can administer the hepatitis B birth dose?

Respondent: I am am am am the one that administers it

Interviewer: as the OIC?

Respondent: Yes

Interviewer: Who else administers?

Respondent: even my assistant OIC

Interviewer: you as the OIC, the assistant OIC, who else administers?

Respondent: My volunteers can do it if I am not around

4c. What are challenges/barriers to getting the hep B birth dose vaccine?

Respondent: Some people like?

Interviewer: Some babies, some newborn babies, what are the barriers stopping them from getting it?

Respondent: The problem is that when the parents did not know the importance of that vaccine, they cannot come to the health center and ask us about the vaccine

Interviewer: So, what other challenges do you think?

Respondent: Another challenge is that we do not have enough staff so that we will be going to outreach to far place from this health center and tell them the importance of that Hep B.

Interviewer: So, you have talked about what you mean is that you don't have enough staff to conduct outreaches

Respondent: we cannot close this health center and go for outreach

Interviewer: any other reason again?

5. Have you gotten information from participants regarding their experiences with the hepatitis B birth dose?

Respondent: You mean the complain about the side effect or what

Interviewer: not really, both side effect like a feedback, the experiences from the caregivers, the mothers, or the fathers. They will give you feedback either the good or the bad experiences.

Respondent: they do

5a. What is the community understanding of the hepatitis B birth dose?

Respondent: I do not know about the community

5b. Are people interested in getting the hepatitis B birth dose?

Respondent: they are interested because we are telling them about the importance of it during the antenatal

5c. Can you describe what kind of specific information/messaging you have heard?

Respondent: the specific messages is that if you have already got the virus, you will not take the Hep B vaccine again because you have already immune to it

Interviewer: So, what other one?

6. Do you have any ideas or suggestions for improving hepatitis B birth dose vaccination rates?

Respondent: I do not have any idea or suggestion for increasing it

Interviewer: Yes, so that people will come and take, so that people will be bringing their newborn babies to come and collect the vaccine to come and access the vaccine so that the vaccination rate for Hep B birth dose in your facility will increase

Respondent: It is going out for outreach now or visiting

6ai. At the community level

Interviewer: So, at the community level, carrying outreach?

Respondent: Yes, we do but due to lack of staff, we go only once a month

Interviewer: So, what you are saying is that we should increase outreaches at the community level?

Respondent: Yes

6aii. Health care system level

Respondent: It's telling the community about Hep B birth dose

6aiii. Provider level

Respondent: within the health, my co-workers?

Interviewer: yes, within co-workers, you people are the providers, you people are the one that provide the services

Respondent: what we are going to do is tell them during the antenatal or immunization sessions the importance of Hep B virus at birth

6b. Are there specific strategies for education or awareness that might be most effective?

Respondent: we will look for town announcers that will tell them that we are coming

Interviewer: okay so what other strategies?

Respondent: jingles on the radio, in the church announcements

Interviewer: Any other strategies?

Respondent: telling the Igwe of the community

Interviewer: Involving the traditional rulers

7. Can you tell me about your experience with the maternal tetanus vaccination?

Respondent: my experience is that during the antenatal, the first dose will be given after 4 months of pregnancy, then at an interval of 4 months, you give another one. Then after the birth you give the third one. Then if you want to go for live, we will give you 5 times.

Interviewer: okay, this is beautiful. You have given us the schedule for TD vaccination. Those vaccinations that you have done, what are the experiences that you have had?

Respondent: the experience is that during the labor, there has not been any fits or plagues around here because of the tetanus toxoid they have already completed. And after delivery they will take another dose which is the third dose

7a. What barriers or challenges have you experienced related to the maternal tetanus vaccination?

Respondent: The experience is that sometimes they will not come at the date you told them to come and take the second dose. Some of them will not come.

Interviewer: okay, so what other challenges?

Interviewer: Okay

7b. What successes have you experienced, what is working?

Respondent: the success is that it prevents them from getting fits during their delivery

Interviewer: So, what successes have you experienced with the mothers that you can talk about during this vaccination against maternal tetanus. You have mentioned on that they do not get fits.

Respondent: you mean in the pregnant mother or other non-pregnant

Interviewer: both as regards ermmm maternal tetanus vaccination

Respondent: okay, I do not know any other thing again

8. What types of health interventions have been the most successful in your community and why have they been so successful?

Respondent: Health education in the community

Interviewer: on what?

Respondent: ermmmm prevention diseases like HIV, Covid-19 virus

Interviewer: why do you think it was successful?

Respondent: It was successful because we do not have any cases since I have been working in this place

Interviewer: that is number of cases have reduced?

Respondent: yes

9. Is there anything else you think we should know about hepatitis B, sharing information about hepatitis B, or the hepatitis B birth dose?

Respondent: concerning Hep B?

Interviewer: concerning Hep B either the virus or the birth dose vaccination

Respondent: I do not know oo

Interviewer: Okay. So, we thank you very much for your time. Everything here will be confidential. We thank you very much for the good work you are doing at the facility.

Note taking template for Pregnant women KII guide State 2, LGA 3, Facility 9

Demographic information of interviewee

Age: 40

Questions

1. What do you know about hepatitis B?

Respondent: No

2. What do you know about the hepatitis B birth dose?

Respondent: No

2a. How do you feel about the hepatitis B birth dose?

Respondent: NA

2b. Have you ever asked for the hepatitis B birth dose before for other children?

Respondent: I have not asked

3.Do you plan to get the hepatitis B birth dose for your baby?

Interviewer: The Hep B birth dose is that vaccine they give to a baby immediately within 24 hours after delivery and it is compulsory

Respondent: okay. So, in that case, my first child has it

Interviewer: so ermmm, okay let me go back to the previous question.

Have you ever asked for the Hep B birth dose, did you ask for it before they gave you or the nurses gave you?

Respondent: No, they just gave it to her

Interviewer:

Respondent:

3a. Why or why not?

Respondent: to avoid the child having hepatitis. To prevent the child from having hepatitis

3b. Do you have any feelings of concern, stress, excitement? Why?

Respondent: I don't understand

Interviewer: Do you feel concerned about the Hep B?

Respondent: as in giving the vaccine?

Interviewer: yes

Respondent: to prevent the child from having it

Interviewer: from having what exactly?

Respondent: the hepatitis

4. Do you think the hepatitis B birth dose will work to prevent hepatitis B? (Why or why not?)

Respondent: Yes

Interviewer: why do you think so?

Respondent: it will, I think I, I know prevention is better than cure

5. Do you think you could easily get the hepatitis B birth dose vaccine if you asked for it?

Respondent: Yes

Interviewer: if you request for it

Respondent: Yes

5a. Will you ask for it?

Respondent: no that I have known I will ask for it

5b. Why do you say that?

Respondent: because I have known the importance

5c. Is there anything that might come in the way or challenges you might face related to accessing the birth dose?

Respondent: it is only the health worker that will say it is not necessary or it is necessary for me either to go ahead or not

Interviewer: Apart from the health worker, is there anything that will distract you?

Respondent: I don't think so

6. Do you feel the hepatitis B birth dose is important for you to get for your child? (Why or why not?)

Respondent: Yes

Interviewer: why?

Respondent: to prevent them from contacting the disease

7. In general, do you think pregnant women in your region get the hepatitis B birth dose for their babies?

Respondent: I do not

7a. What gives you that level of confidence (or lack of confidence)?

Respondent: I don't understand this question

Interviewer: you see when you are sure of Hep B vaccine that you will take it so it will prevent your children from having hepatitis, so do you have confidence on the vaccine?

Respondent: Yes

Interviewer: So, what give you that level of confidence? Why are you confident about it?

Respondent: I have confidence about it because doctors approve it, health worker approve it

8. Do you have any suggestions for us if we want to educate people in your community about the hepatitis B birth dose?

Respondent: okay, my suggestion is that if you people want to come nd educate people, you can give them prior information that you people are coming so they can be around. Then you kind of organize a seminar

Interviewer: so apart from organizing a seminar, which other aspect again?

Respondent: then through, you can still educate them through fliers and posters

8a. Would you prefer to learn about hepatitis B? (Through stories, videos, flyers, other methods)

Interviewer: would you want to learn more about Hep B?

Respondent: Yes

Interviewer: how do you want us to give you more information on Hep B?

Respondent: For me I will prefer through phone. I think you said through phone?

Interviewer: I talked about like stories, video, movie, through fliers, banners, radio

Respondent: for me I prefer through video

Interviewer: what other method again will you prefer apart from video?

Respondent: Fliers

Interviewer: is there any other method again?

Respondent: No

8b. How do you prefer to learn about health issues?

Respondent: I can learn through fliers or posters

9. Is there anything else you think we should know about hepatitis B, sharing information about hepatitis B, or the hepatitis B birth dose?

Respondent: okay, before now I don’t know about it before, so I don’t have anything to tell you

Interviewer: Okay, thank you very much. We are so grateful you have done marvelously well. so thank you for the privilege to carry out this interview. Once again, have a lovely day.

TRANSCRIBED KII FOR DOCTOR State 2, LGA 4, Facility 1

Interviewer:

Okay, good afternoon, ladies and gentlemen. We are here lives at [facility name]. With the [name], the clinician in charge of this facility, and then Okay Then also the I'm here with my team. Also, I'm here with [name]. And then Madame ad from the state healthcare agency, and I'm your humble [name] So we will be having this key informant interview with [name] will get to know his feelings and his opinions about issues bothering on Hepatitis B first dose ,So we'll start by introductions, sir, May we know you sir?

Interviewee:

Am [name] I'm 57 years old. Okay.

Interviewer:

So how long have you been in this facility?

Interviewee:

So, I've been around the world. Well, around 13years 2008 till now

Interviewer:

Okay, so before we proceed, I would like to get your consent officially or record that you are okay with the asking you questions bothering on Hepatitis B first dose as the physician in charge of this facility, So do we have your consent?

Interviewee:

You see, just as I said, I'm only work in there. I'm not in charge of that facility .i only see out patients. That is all I do there.

Interviewer:

Thank you, So do we have consent to ask your questions?

Interviewee:

You have my consent, if requested to be restricted to what I've just told you is my circle of focus, if it goes beyond what is my duty, I may not assign it correctly.

Interviewer:

Thank you very much sir. So we'll try to restrict ourselves. Thank you, sir. So first and foremost, in your opinion, what are the biggest challenges for people living with hepatitis B, within a community? When I mean community, I mean, the catchment area of this health facility.

Interviewee:

The challenge is exactly the challenges every other person has Because the greatest problem is access to that healthcare, People don't have access to health care, either because of ignorance, or because they don't have the awareness of some other disease, that’s the only challenges

Interviewer:

Now that you’ve actually pinpointed some challenges, What do you actually think are the biggest needs to address these challenges?

Interviewee:

The best thing to do is that the government should organized Health Education and Health Awareness outreach, So that people will know exactly what are the services being render in health facility, because I believe so many people don't even know. And if they don't know, they won't access that health facility for such interventions.

Interviewer:

So, is there a strong need for hepatitis B first dose in your opinion?

Interviewee:

There should be

Interviewer:

So why would you say so

Interviewee:

Prevalent to Hepatitis B is very high, is obvious because the awareness is not there they don't access those interventions given to them at the health facilities.

Interviewer:

Do you think others actually see this need? Just as you're seeing it? Do you think others are seeing the need to have hepatitis B first dose?

Interviewee:

I wouldn’t talk for others, I'm only giving my own candid opinion as a medical practitioner, I'm not saying based on any other information else where

Interviewer:

So can you tell me about the current programs and practices for hepatitis B first dose within this particular health facilities that you are aware of

Interviewee

Just as I said earlier, that there is a schedule of duty handed down to me when I came here, irrespective of all my efforts that has been thwarted, so I've restricted myself to the schedule of the duties, seeing our patients. Because ideally, are to work as a team, everybody will be involved, if there is training or whatever everybody should be involved, so that everyone should be able to give his own candid advice or suggestions. But if you are skewed out, what do you do? You restrict yourself to what you are told to be doing.

Interviewer:

For which, in his opinion in this context is seen as the answer Yes. Okay. So you don't have any connection with deliveries, deliveries,

Interviewee:

Deliveries, if there is if there is a somebody in level, okay, and the workers felt the need for a doctor to intervene, and assist and I called upon and I'll do it.

Interviewer:

Have you had any such occurrences?

Interviewee:

Why not? Why not? Everything pertaining to immunization you understand? Yes. I don't know anything about it. I'm not involved, As the as the primary health facility, I'm not involved at the local government level,I don't know anything about it.

Interviewer:

So, that's okay. So is the hepatitis first dose easy to get within 24 hours?

Interviewee:

If you're , if you're asking me that? You're trying to lose my legs? Because I told you everything about immunization. I don't even know the stock they have, You understand is when you know the stock in the in the clinic, I don't even know if they have this dose of hepatitisB whatever, I don't knowUnknown

Interviewer:

That's okay, so, so, so sorry, sorry, for that, I'm just trying to assimilate what you said. So, so Okay. However, when your opinion as a professional ,in your opinion, you think can administer Hepatitis B first dose?

Interviewee:

I believe the workers are doing fantastic job, I believe so.

Interviewer:

So when you say the workers, what did u mean?

Interviewee:

The workers at the different categories, just choose their and choose, and the judges that populated the facility, I believe that just because they receive regular training at the local government so I believe they are doing a fantastic job.

Interviewer:

So we need out of grace to ask you what are the challenges or barriers to getting Hepatitis B first dose vaccine?

Interviewee:

Baby, if I were to answer is based on literature, okay, not based on experience, So availability should be one of the challenges.

Interviewer:

Okay, since have you in any way, come across anyone who have I mean, in any will come across anyone who have gotten or have accessed HepatitisB first does?

Interviewee:

No

Interviewer:

And any feedback?

Interviewee:

Have not

Interviewer:

Okay, so in your opinions sir, what do you think is the community's opinion, community's perspective about this? I mean general opinion, the community in your own coming from your own experience, what do you think I just

Interviewee:

As I told you earlier, I'm not into that intervention. I don't interact with the general public on it

Interviewer:

So people don't come to you like as a medical practitioner to discuss issues

Interviewee:

No

Interviewer:

Okay. Okay. So so then can you also well, could you say that people are interested in things that concerns hepatitis first dose?

Interviewee:

How can I tell you that when I told you that I don't interface with them

Interviewer:

Okay, so Okay, now looking at health generally. How would you getting people best assessed access health information? What do you think is the best way for people to access health information?

Interviewee:

Health outreach interventions, organizing health education, meeting people in the churches in all these faith organizations, okay, schools talking to them, that is how they will assess it And then announce on radio or television

Interviewer:

Okay, so generally now, do you have any suggestions? What are your suggestions for improvement? If what to improve,Probably with the rate, they improve the rate of immunization or to improve generally speaking, of personal point of view, what do you think we can do to improve vaccination rates, generally? Awareness, anything what can we do,

Interviewee:

I believe is running an inclusive organization has let every hand be on deck, so that everybody will give his/her own suggestions. So, apart from that, then health information services should improved, so that people will now get awareness of what is available in health facility? And how and how they will assess them.

Interviewer:

Okay, so, could you somehow, if we were to break it down into bits and pieces, can we just take it Okay, at the community level? What improvement measures do you think we can do? Again, at the health or the health care system level? What do you think we can do? And then at the provider level in the facility, what do you think we can do to improve hepatitisB vaccination?

Interviewee:

So if we start at the community level, awareness creation, so that the people will know what services can be rendered at the health facility. Then at the health facility level, the workers should be up and doing So that if they if the people have the information and come, they won't be disappointed if they don't get those services

Interviewer:

Ok at the health care system level which administer, I actually mentioned health care healthcare system level and then provide

Interviewee:

At the healthcare system level they should provide those vaccines.

Interviewer:

Well, I wouldn’t want to draw you into pure vaccination, since you say you're not fully. However, I would have wanted to ask you, so, are there specific strategies for education or awareness that might be most effective? In your opinion?

Interviewee:

I wouldn't be able to tell you that, because I'm not involved in immunization.

Interviewer:

Yeah, I know. But since you talked about awareness, are there strategies you think we can actually bring up to strengthen Or to boost awareness? If awareness is your opinion, what strategies?

Interviewee:

Okay, so many strategies? Right. Okay. I don't know exactly what you are pointing at

Interviewer:

Okay, well, like generally, then I say, okay, probably we'll talk about health education and health awareness. We'll actually looking at people going to school, this was it. Oh, so I just tried to get your own perspective, being someone in the grassroots, What strategy do you think we can employ?

Interviewee:

I think, for me, the best strategy is meeting them, meeting the community and meeting people at the community level and giving them proper information about the important of immunization. Telling them the need to present their children for immunizations immediately after birth and telling them what immunization is all about. And what it's the benefits they will benefit to get from it.

Interviewer:

Okay, yes. Okay. So So, let me take you off a little, in as much as you say you're not fully involved in immunization. But then I want to believe you're married, right? Yes. You have a wife? Yes. You have a family?, kids?, Yes. So probably, you must have had one or two interactions, even if not outside. Even

within your own family as a home. So can you tell me about your own experience with maternal tetanus vaccination? Tetanus now

Interviewee:

Okay. My experience, I don't have any negative experience because when my wife was pregnant, I know get got maternal tetanus vaccination

Interviewer:

Okay. So the way she gave birth, Do you know if she got those hepatitisB first dose, where does she give birth, Was it within? I'm just I'm just been curious.

Interviewee:

At the health facility, that time I was at the health facility

Interviewer:

Okay, Okay, so what barriers or challenges have you experienced? Now? Probably from looking at it generally Now, have you experienced related to maternal tetanus vaccination?

Interviewee:

Have not

Interviewer”:

Okay, you've not had any challenges. Okay, so, do you have success stories about vaccination hepatitis tetanus vaccination?

Interviewee:

Essentially the success story. Yeah. Is that that is a very remarkable decline.

Interviewer:

So what do you think is actually working?

Interviewee:

Because of the vaccination

Interviewer:

Okay. Okay. So well now that you're also into health? What types of health interventions have been the most successful? In your opinion? What health interventions? Generally speaking, you think have been the most successful in your community? Or why do you think that?

Interviewee:

Intervention?

Interviewer:

Yes

Interviewee:

That's immunization Yeah. It has been very successful,

Interviewer:

Why would you choose immunization over other health intervention?

Or what interventions are actually ? what types of health interventions so you say vaccination, so why?

Interviewee:

There are health interventions are many, okay, you understand, but I thought you are still talking at the level of immunization, at the level of hepatitis B immunization, that is what I was talking about. The establishment ahead facility is an intervention now. Because if you do, and there are so many of them around and there are workers posted to work there. These are the interventions

Interviewer:

So is there anything else you want us to know? Probably centered around Hepatitis B, or information dissemination? Or hepatitis first dose generally any other things you Want to add at this point? Or probably vaccination generally?

Interviewee:

The only thing I will say is that ,I will say that the creating awareness for people to know about immunization is very important. And all hands on deck to make sure that at least all the nooks and crannies of the community.

Interviewer:

Wow, it's been a wonderful moment having you for this interview. So I want to thank you one more time to make enough time from your very busy schedule to give us audience responses. You've given us a shot. I'm very sure is going to go a long way. He knows know making informed decisions as far as like hepetitisB and maternal tetanus vaccination is concerned. Thank you one more time for your time over and out. Thank you.

Interviewee:

Thank you, you welcome

TRANSCRIBED KII FOR PREGNANT WOMAN State 2, LGA 4, Facility 1

Interviewer:

Ndewo Nne, Ndewo. Kedu ka I mere? Ahu o di kwa? Umuaka kwanu?

Interviewee:

O di nma. Umuaka m di cha nma

Interviewer:

Anyi bun di oru ahu ike. O nwere obere ajuju ndi anyi choro I jug a si gi. Anyi lere anya na I ga enye anyi ohere ka anyi new ike juo gi ajuju ndi a? I ga a' sa anyi anyi e tu I si ghota. O nwe kwa nke na e do gi anya, I juo ajuju. Ndi mu na ha so bun di oru ahu ike. Aham bu [name], Onye otu m bu [name] Yan a Nne anyi ukwu bu madam Ede siri nah state primary health care. Ndi ozo no ebe a bun di oru ahu ike. Nke a putara na anyi abugi ndi oso chi egbu, anyi bun di oru. Ajuju ndi choro I ju gi abugi ndi ga ebute nsogbu nihi nke a anyi ga cho ka e were obi umeala saa anyi ajuju ndi a. I siri anyi juwa gi?

Interviewee:

Nsogbu a dighi. Juo Ajuju gi

Interviewer:

Kedi aha gi? E di afa ole? E bu onye Ebe? E ji umu ole? E di Ime? Ime Onwa Ole?

Interviewee:

Aham bu [name]. A dim 33 years. A bum onye Imo state mana a'na lum na Amaboukwu Aku. E jim umu Ise. [name].

Interviewer:

E nu la maka Oria eba ocha nánya?

Interviewee:

Eh a nu lam maka oria eba ocha nánya.

Interviewer:

Kedu Ihe ndi e nuru maka oria na ogwu eba ocha nánya?

Interviewee:

O mebeghim mbu, ma na a'nam a nu maka oria na ogwu eba ocha n'anya. Onwe kwa mgbe anyi na bia ebe a kányi gba ogwu eba ocha n'anya. O mebeghim mbu kama a nam a nu maka ya.

Interviewer:

E muola umu ole núlo ogwu a mbu?

Interviewee:

A muolam umu abuo ná ulo ogwu a. Nkea bu nke ato mana ebe a kam ga a mu kwa ya.

Interviewer:

Mgbe i muru nwa n'ulo ogwu a, Ha gbara nwa gi ogwu ngbochi oria eba ocha nánya nke mbu?

Interviewee:

Eh mgbe mmuru nwa izizi mu ebea, ha buru uzo gba ya BCG, Mgbe otu onwa ga siri, me kute kwa ya. Ha gbara ya ogwu ndi ozo ná ukwu mana aka. Ha gbakwara ya ogwu ruo 9 months.

Interviewer:

Ozugbo ahu e muru nwa, ha gbara ya ogwu eba ocha nánya nke mbu?

Interviewee:

Eh ha gbara ya ogwu eba ocha nánya

Interviewer:

Ele otu ogwu mgbochi eba ocha nánya ha gbara nwa gi di gin obi? O di gi nma ka o di gi njo?

Interviewee:

O dim nma di kwa nwa m nma nah u maka na onwe gi ihe omere ya na hu.

Interviewer:

Kedu kwa nu maka umu ndi ozo? O nwere mgbe e biara juo ajuju ka ha gba umu gi nah umu ndi ozo ogwu eba ocha nánya?

Interviewee:

Ha na ezi anyi ozi. Anyi na no nulo ha zie anyi ozi k'anyi bia gba ogwu eba ocha nánya. Ha na emeta anyi ofuma.

Interviewer:

Kedu ka obi di gi maka ogwu eba ocha n'anya nke mbu ha na gba nulo ogwu a?

Interviewee:

Obi dim uto maka na gburu gburu ebe obibi anyi, onwe bo gi onye ma obu nwatakiri ha siri na eba ocha nánya gburu.

Interviewer:

Onwere atuma tu e nwere maka I gba nwa nke a e di ime ya ogwu ngbochi oria eba ocha nánya nke mbu?

Interviewee:

Eh a jugom ha ma ha ga agba ya ogwu, ha siri eh

Interviewer:

Kedi ihe siri gi obi ike e ji nátu atumatu e gba nwa gi ogwu eba ocha nánya nke mbu?

Interviewee:

O bum maka na kamgbe m bidoro gbawa umuakam ogwu núlo ogwu a, o nweboghi onye ria ra ya

Interviewer:

Onwere nsogbu obula e nwere banyere umuaka gin a umuaka ndi ozo gbara ya bu ogwu?

Interviewee:

O nwegi nsogbu obula

Interviewer:

Núche gi, I chere na ogwu eba ocha nánya nke mbu ha na gba umuaka na ru oru?

Interviewee:

Eh e kwetaram na o na ru oru nihi na onwebeghi onye obu la m huru nime obodo anyi n aria oria eba ocha nánya.

Interviewer:

Ná uche nke gi, I chere na o di nfe I nwete ogu oria eba ocha nánya nu logu nile?

Interviewee:

A maram nah o dighi nfe nwete ya mere m ji na ekele unu bu government e tu unu si gbalia e me ka ogwua na di núlo ogwu nke anyi a.

Interviewer:

I ga aju ajuju maka ogwu eba ocha nánya nke mbu maka nwa gi?

Interviewee:

Eh agam a ju maka ya.

Interviewer:

Kedu ihe I ga eji eme nke a?

Interviewee:

O bu maka na ogwu a di nma nke ukwu ni gbochi oria eba ocha nánya

Interviewer:

O were Ihe obula ga egbochi nke a nu che gi?

Interviewee:

Mba O nwegi ihe obu la ga egbochi ya

Interviewer:

I chere na umu nwanyi ndi no nime obodo na agba umuaka ha ogwu mgbochi oria eba ocha nanya nke mbu?

Interviewee:

Eh ha na gba umuaka ha ogwu a

Interviewer:

Kedi ihe I kwere che otu a?

Interviewee:

Ihe kwere che out a bun a ndi oru ahu ike n'abanye nime obodo na gba umuaka ogwu mgbochi di iche iche.

Interviewer:

Kedi ozi oma ndi I were ike gwa umu nwanyi ndi no nime obodo banyere ogwu mbochi oria eba ocha nánya nke mbu?

Interviewee:

N'úchem, unu ga ejisi ike gwa ha ka ha mara udi ulogwu ha n'aga ka ha nwee ike igbazu umuaka ha ogwu tumadi ogwu iba ocha n'anya.

Interviewer:

I ga–acho imata kwu ihe banyere oria na ogwu iba ocha n'anya?

Interviewee:

Eee, a choro'm imata kwu ihe banyere ya ka'm nwee ike iga ndi ozo.

Interviewer:

kedu uzo I chere ga akacha nma ha ga esi kuziere gi banyere oria na ogwu iba ocha n'anya?

Interviewee:

E cherem na unu ga na agwa umu nwanyi okwu kwa mgbe na ulogwu ma obu unu edee ya na-akwukwo mado chaa ya pole n'uzo n'ile no n'ime obodo ga akacha mma iji kuziere umu nwanyi banyere oria na ogwu iba ocha n'anya.

Interviewer:

O nwere ihe ozo I ga acho igwa anyi?

Interviewee:

ihe ozo m ga-acho ikwu bu ka unu gbalia na ebutere ha ogwu ebe a ka ha n'agba umuaka anyi ogwu iba ocha n'anya maka na odi nma buru kwa ihe ziri ezi.

Interviewer:

Ndewo maka oge iweputara saa ajuju ndi a.

Interviewee:

Ndewo...

TRANSCRIBED KII FOR PREGNANT WOMAN State 2, LGA 4, Facility 2

Interviewer

Good afternoon, Madam. Okay, please. My name is [name]. And then with me is my colleague, [name], and madam [name]. Good afternoon all. So we will be conducting a KI, a key informant interviews on you. And then we'll also first and foremost require your permission, then your consent to actually go ahead. So I don't know if you have to have your consent to go ahead and ask you these questions.

Yes.

Okay. Ma

So, ma I want you to start by way of introduction. Just tell us Who you are?

Interviewee:

My name is [name] and I'm 28 years old.

Interviewer: Okay. [name]. Good afternoon. [name], please. What do you know about Hepatitis B?

Interviewee

Okay. I don't know much but I know little. I know It's an immunization given to kids. Okay. It's for health purpose. Okay. It's, that's the little I know about it.

Interviewer:

Do you know anything about Hepatitis B? Birth dose? You know, When I say birth dose I mean given to children at birth, are you aware about it?

Interviewee: Yes, at child birth.

Interviewer: Okay, so what level do you know about it? About giving hepatitis B vaccine at birth? What and What do you know about it? What knowledge do you have about it?

Interviewee: I don't really know, but I believe it's given to kids at the age of day old till a month or two months

Interviewer:

Is okay. You know, sorry. I actually didn't ask, are you pregnant? For the sake of those listening to us, whats the duration of pregnancy?

Interviewee: I'm three months pregnant.

Interviewer:

Three months pregnant. Okay. So, so how do you feel about giving children Hepatitis B at birth? That is, birth dose. How do you feel about hepatitis B birth dose.

Interviewee:

I believe it helped them now. It's helped them grow and prevents them from disease and infections, sickness from contacting from maybe the environment, weather and all that, so.

Interviewer:

Okay, so have you ever have you ever asked for the hepatitis B birthdose before for other children or your children? Have you asked them you requested for it?

Interviewee: Yes, yes. Yes.

Interviewer: When was that?

Interviewee: That was six years ago when I gave birth to my daughter. My first daughter?

Interviewer: oh you have a daughter?

Interviewee: Yes.

Interviewer: You requested for it

Interviewee :

Yes.

Interviewer :

Was it given? was that request granted?

Interviewee :

Yes, it was granted

Interviewer :

Okay, so now you're pregnant. Right? Do you plan to get Hepatitis B birth dose for your baby?

Interviewee :

Yes, I do.

Interviewer:

Why?

Interviewee : I believe it's helped their health. Yes, it helped them. So it's good for kids for children, it's very important for children.

Interviewer: So you plan to get it for your child?

Interviewer:. Okay. So what I wan..do you have any feelings or concerns or something that makes you somehow happy about Hepatitis B birth dose, that is, giving children Hepatitis B at birth, Do you have any concerns? Do you have anything you want us to know about it? Or is there anything very appealing to you like,

Interviewee: it's nice, at least for the for government to or for you people under health to bring up the topic of Hepatitis B, I believe it's good for them ,for kids. That's why they brought it up. Because if it's not good, they won't bring it. So it's very important. That's why it's existing. So I it's very important. I urge parents to always give to their kids, because I believe it's helped them and it's going to help them.

Interviewer: Okay, so do you think, in your experience, do you think Hepatitis B birth dose would walk you know, to prevent Hepatitis B? Do you think it actually works even though given at birth?

Interviewee:Yes.

Interviewer:Why? Why do you think so?

Interviewee: I if, if it doesn't work, it will not come to existence. Yes. And children have taken it. I believe it's prevent them from a contacting the Hepatitis B disease or whatever. So I believe it works. That's why it's there. And that's why they advise parents to give to their children.

Interviewer: For you, are you actually speaking from experience, did it work for you?

Interviewee: yes, it works for me, and I believe it works for my friends that have given to their kids.

Interviewer: Okay, yes. Okay. So the, but, in your own experience Also, do you think you could do think that getting Hepatitis B birth dose? You know, Hepatitis vaccine at birth dose, do you think it's easy to get? Do you think it's something you can easily get? Or is it? Is it? Is it difficult to get these children vaccinated with hepatitis B at birth? Do you think it's easy? Easily accessible? Or do you think it is difficult to get?

Interviewee: It's it's quite easy. If you request for it, they will give it to you.

Interviewer: Oh really? If you ask for it? So will you ask for it?

Interviewee. Yeah, I'd ask for it. I'd ask because it's important, it's better they take it on time early than later on, they'd start looking for it. Or maybe at that time, they will not be able to get it when they start having the sickness. So it's better to take it from birth So as to prevent it for future purpose.

Interviewer: So when you say early,I really want you to break down early, you see because early can be anytime. So, you know, are you really talking,how early is early?

Interviewee: Atleast from that very first day of your childbirth to like atleast three, four or five months is better you get to give to them. So that it will help them to prevent the sickness.

Interviewer:Okay, so judging from experience right now, you have a child, he or she,?

Interviewee: She

Interviewer: She was vaccinated at death, right?

Interviewee: Yes.

Interviewer: You also have friends that have been vaccinated at birth. Okay. So so from your experience, is there anything that might come in the way you know, like, or like a challenge you might face related to accessing Hepatitis B first dose? Is ther anything you feel like a challenge people might face in their quest to access?

Interviewee: Me, We never face any challenges? Yes, it was very easy. But I don't know to others. But I believe it was very easy for us to get for my kids, my daughter to be vaccinated. So it was easy.

Interviewer: Okay. So do you feel like this B birth dose is important for you to get for your child?

Interviewee: Yes, it’s very important.

Interviewer: Why? Why do you feel so ? you know?

Interviewee: it's, it's important in the sense that, you know, we parents, it's, sometimes we can just go we can just get away with other house chores and family issues and we forget about this thing. So it's better we give them on time before it's break them down or before the sickness starts occurring. And from there we’d start running up and down and and we’d start doing test and at the end of the day, we'll find out that same thing we were supposed to get when the baby was small. That's the sickness the baby is having now. So we start looking for it, at that time, it might be very difficult to get, ehen, because we don't request on time, because of the condition. So it's better just give to them when it's necessary. And when our mind come down, to it. so

Interviewer: It’s good to know that. So So in general, in general, your general perspective, do you think pregnant women just like you, in your region, when I mean Region, like within this region, maybe in South East, in this state, in this area? Do you really think in general that they get Hepatitis B first dose for their children?

Interviewee:Yes.

Interviewer: you think they do that?

Interviewee Yes.

Interviewer: Why would you think so?

Interviewee: Because I actually gave my daughter her own in this place. So they get it.

Interviewer: Okay,

Interviewee: yes.

Interviewer: So there's no form of scarcity or something? And it's not difficult.

Interviewee: no, I'm not sure, no.

Interviewer: Okay. So what gives you that level of confidence? What makes you feel that all the women within this region , within this confines actually get this?

Interviewee: I've been seeing them coming, I've been seeing women coming down my floor, my friends, living around,they like I want to give my daughter , my child, I'm coming to the health center to give them the vaccine and all that. So I believe if it's not, if it's not available, or they're not getting here they won't come.

Interviewer: do you have any suggestions for us? If we want to educate people in your community about Hepatitis B first dose, what what suggestions what ways do you think the better for us to actually be able to access the people and give them information

Interviewee: What I want to advise is that I'll urge I'll beg, the government's or where it's coming from, to make sure it's always available. It should be on ground because every day, ladies, women give birth. So it's, I'd advise that immediately, they just give back to the child. Like from that very day of given birth, to like a month or two months. It's better they give to, they immunize the child with the vaccine. So I urge you guys to try as much as possible to make sure it's always available. It's always you should always be on ground, because some parents can forget about. But if it's on ground and they introduce it most of most of those people are not aware of it, if they come , like they give birth, and they're like this thing exist and you enlighten them they'd be like , Okay, give to my child. And em, because some people are not that educated to this extent that for them to know that something like this exist, but when it's available, the matron or the nurse on duty will be like, something like this exist o. The parents ,the the mother will be like, Okay, I will need it for my child. So if it's not available, the nurse or the matron will not say anything about because even, if she says anything about it, it's not be available to give to the child. So I beg you guys to please try as much as possible to always make it to be available.

Interviewer: So you as a person personally, would you like to learn more about Hepatitis B?

Interviewee: Yes.

Interviewer: So which, which kind of medium Do you think we can use best to teach you which kind of medium can you learn fast from? You know, what way do you think we can actually reach out to you to teach you more about hepatitis?

Interviewee: I advise that you put it on social media? Yes, like on Facebook, and then Instagram. You should make sure they always advertise. , mostly mostly all these a radio station, television station for them to…., for people, because, not everybody that go online or have phone to browse,to go on Facebook, and all that but when…….atleast, most people even in these rural area, they'd have television, and when they start seeing this hepatitis B vaccine and all that , they be like ah, this thing exist and so they will, they'll have the knowledge in this town. So I advise you guys to go on radio stations, television stations, to do more advertisements on this particular vaccine.

Interviewer: So how do you prefer to learn? How do you prefer to learn about health issues? What would you prefer? to learn? How best? Can one reach out to you on issues on health? Generally? Okay,

would you prefer? What would you would you really prefer someone to talk to you about health issues generally

Interviewee: Pregnant women like us, we come to health centers like this. So if we have the opportunity for people like you to come and educate us, enlighten us it will be very good. And it will be very nice, at least, most of us that don't have time for television and phone and all that. If we come to this place, we'll be able to communicate ourselves and get more education. Yeah, on this health issue. To me, I believe if something like that exists , I'll learn more, than me seeing it on television, more than seeing it on social media and all that,

Interviewer: That's wonderful. So finally, in conclusion, is there anything else you think we should know about hepatitis B? Or sharing information about hepatitis B or probably hepatitis Birth dose for you? What other things would you want us to know? with government? Anybody? What what else you have to say that I've actually not asked you? What other general issues?

Interviewee: Nothing much, nothing more, I've said all I know about it and say what is important to know and what is important to do?

Interviewer: Thank you very much for your time. I really appreciate you for making time out of your busy schedule to really give us this audience it will really go a long way to help us understand what we're dealing with. Thank you one more time.

Interviewee: Thank you my

Interviewer: So please, for the audience out there. We are still in [facility name], that is where we are right now. And we just ended this wonderful interview with Madam [name], thank you one more time.

Interviewee: Thank you sir.

TRANSCRIBED KII FOR RI SERVICE PROVIDER State 2, LGA 4, Facility 2

Interviewer:

Hello, good afternoon ladies and gentlemen. So we are at [facility name] for the KII for RI provider. I have here with me, [name], Can you say hi to the people?

Interviewee: Good afternoon.

Interviewer: I also have my colleagues, [name] and then madam [name] my humble self is [name] And together we'll be conducting this our KII interview. So madam [name] can you please introduce yourself to us and tell us your age?

Interviewee : Good afternoon, my name is [name]. 30years old

Interviewer: what is your designation?

Interviewee: CHEW.

Interviewer: you're a CHEW? And what is your work here?

Interviewee: I am an RI service provider

Interviewer: RI provider, Okay, thank you very much. So, before we start, I would like to get your consent, you know, to be able to actually these questions, I don't know, do you give your consent to actually these questions?

Interviewee: Yeah go on.

Interviewer: Okay, thank you very much. So first, what are the biggest challenges for people living with hepatitis B in your community?

Interviewee: The challenges that we are having is that they don't want to come to antenatal care when they are pregnant, because of not taking the TT dose, taking malaria, malaria tablets, and they suppose when they are pregnant, when they give birth to their children, their children will start having Hepatitis B.

Interviewer: Okay. Okay, so So, these are your biggest challenges for you? So what are the biggest needs to address Hepatitis B in the community? Now, you've told us the challenges, what are the biggest needs, you know, to address hepatitis B.

Interviewee: for us to get them, we have to go to the villages ,the rural areas for outreach and tell them the needs of taking Hepatitis B, when they give child to their, birth to their children, then after then we will advise them will tell them the need and the prevention, when you take that Hepatitis B injection,

Interviewer: maybe I'm not trying to preempt you, I'm just trying to guide you a little, you know, needs in the sense that, you know, now you've stated your challenges, right?

Interviewee: Yeah

Interviewer: So, what are the basic needs, the things you actually think you require, that will help you in addressing those challenges? You just mentioned earlier, what are the things you require something like the requirements,

Interviewee: okay, what we require from you people is for you to help us to advertise it through social media, through television station, and radio stations. To tell them what they have to do. The needs of taking those things. So that they will come they will have the confidence to come and register for the antenatal and give the to give their children the injection Hepatitis B injection vaccine.

Interviewer: Okay, so So is there a strong need from your experience? Is there a strong need for hepatitis B birth dose? No, within this community? Do think there is actually a strong need for people to to be given Hepatitis B first dose.

Interviewee: Yeah, Hepatitis B vaccine help children to provide hepatitis.

Interviewer: Well, I'm actually looking at you know, hepatitis B vaccine is one thing, you know, there is also the issue of birth dose, you know, giving it a birth and giving it out all the time, so I'm just asking in your experience do you think there's need to give it at birth

Interviewee: Yes, it's good so that it will boost their immunity that is the help of that first dose. it boosts their immunity even when the disease, maybe they have someone nearby having that disease, because that hepatitis B is a transmitted disease. It will prevent them not to get it.

Interviewer: Okay, okay. So now this is your own perspective.

Interviewee: yeah,

Interviewer: So but do you think , do you see the need for hepatitis B birth dose for others, do you think for others? Do you think they actually see the need to have this Hepatitis B birth dose? Well, from your experience,

Interviewee: many of them value it but many ignore and say that there is nothing that they can do about it. Maybe others will complain their husband doesn't allow them to go to immunization. But we do tell them that prevention is better than cure? When you think that first dose, it will prevent your child not to contact that disease?

Interviewer:

So can you tell me about the current programs or practices for the hepatitis B birth dose in your clinic. This your clinic here. Can you tell us more about current programs ongoing now or practices that you do here for hepatitis B birth dose?

Interviewee:

Well, when they come for immunization on Wednesdays, we advise them, we tell them everything about all the immunization not only the hepatitis B.

Interviewer:

You tell them on Wednesdays?

Interviewee: Yeah.

Interviewer:

So there's no special program ongoing? So the Hepatitis B birth dose is easy to get within 24 hours of bed?

Interviewee:

Yes

Interviewer:

Very easily. Why?

Interviewee:

We have it here and whenever we requested for it, or maybe we are lacking, we just go to the Ogbede Central store, we get the vaccines.

Interviewer:

Okay. So who can administer the hepatitis B birth dose?

Interviewee:

The CHEW working here can administer it.

Interviewer:

Only the CHEW?

Interviewee:

Even our OIC, the RI, anyone.

Interviewer:

Okay. So what are the challenges and barriers to getting hepatitis B birth dose vaccines? Are there any challenges or barriers to getting the vaccine that you know of?

Interviewee:

No

Interviewer:

No challenges. Okay. So have you gotten information from participants regarding their experiences with hepatitis B, birth dose?

Interviewee:

No.

Interviewer:

You have not gotten any information from people that have gotten it?

Interviewee:

No. Because when they come, they tell us that their child is having one sickness or the other, or maybe it is jaundice. We will now give them the HBD.

Interviewer:

Okay, so what is the community's understanding of Hepatitis B birth dose?

Interviewee:

Well. Some would tell you that it is not the vaccines that will prevent it. Even the one that -the person that has it- when the person, maybe did not take the malaria tablets, maybe the child may get the disease after birth, but we make them to understand that when you are pregnant, you think the malaria drugs, at it suppose, that it will not allow your child to contact the disease.

Interviewer:

Okay, thank you very much. So if you are listening, I've actually been laying emphasis on birth dose for some reasons, right? Hepatitis B. In as much as I know that Hepatitis B will actually be taking some other time, but I've actually been laying emphasis on the birth dose. So do you think people are actually interested in getting Hepatitis B birth dose? Do you think they're interested?

Interviewee:

Yes, they're interested.

Interviewer:

Why would you say that?

Interviewee:

Many of them are literates. And more, especially, those people that are literate, they will like to immunize their children more especially on that first dose. But the illiterate ones will ignore it.

Interviewer:

Okay, so as far as Hepatitis B birth dose is concerned, can you describe what kind of specific information or messaging you have heard about Hepatitis B?

Interviewee:

Hepatitis B first dose help children to get more immunity to fight against that disease.

Interviewer:

So do you have any idea or suggestions for improving Hepatitis B birth dose vaccination rate from where we are currently? Do you have any ideas or suggestions you want to give us to help, you know- would you think would help -to improve the vaccination rates of children with birth dose?

Interviewee:

What we are begging you people is for you to go to social media, advice for it, do advertisements for it. Tell them what first dose Hepatitis B can do for a child, so they will get to know about it. And they'll be taking the children to the health centers to take the immunization.

Interviewer:

Okay, so what about at the community level here? At the committee level, what suggestions can we use to improve vaccination rates for hepatitis B birth dose?

Interviewee:

You go there, maybe we send some people that will go to outreach, or you send the health workers to go to the rural area, tell them the needs and tell them to take the children to the health centers to take the vaccines.

Interviewer:

Okay, so what about at health care system level as health workers, what suggestions do you have? If what you just said, the suggestion for reaching the community? What about in the program itself as health care workers generally? What suggestions do you have?

Interviewee:

What I suggest is that all the health workers will improve to go for the training to learn more about it. It will help them to spread the news to the communities.

Interviewer:

So what about at provider level, what suggestions you think we can use to improve reach of Hepatitis B birth dose?

Interviewee:

What you people will do for us? Well, give them more light on it. That’s the only thing. And provide more equipments.

Interviewer:

Okay. Can you be more specific?

Interviewee:

Maybe, others may say that there are not sleeping under nets, that makes them to get malaria because of a mosquito bites. You give them something that they will give to those children, because many of them will be coming here and telling you that they don't have mosquito nets since they have been immunizing their children, no gifts for them. So we need that gift that will attract the attention. Yes.

Interviewer:

So are there specific strategies for education or awareness that might be most effective in your opinion? What strategies can we use to educate or make people more aware?

Interviewee:

Okay. Maybe you may bring a picture of someone having Hepatitis and how you can treat it and how you can prevent your child from getting it. When they see the poster, you will teach them through that poster and they will understand.

Interviewer:

Is that all? Okay. So can you tell me about your experience with maternal tetanus vaccination. What is your experience?

Interviewee:

That tetanus help the pregnant woman not to have maybe convulsion through labour? You tell them the need, that someone that did not take the TT may actually have convulsion through the child birth.

Interviewer:

So what barriers or challenges have you experienced related to maternal tetanus vaccination? What are the challenges you've experienced related to the tetanus vaccination?

Interviewee:

I have not seen.

Interviewer:

Okay. You've not experienced any challenges or barriers. So in your opinion, it is okay to take the tetanus vaccination?

Interviewee:

The barriers of not getting it right?

Interviewer:

Something like that you know. Challenges, general challenges, bottlenecks, barriers as regards maternal tetanus vaccination.

Interviewee:

Okay. No for now.

Interviewer:

So what successes have you experienced? What is working for you? What have you actually, what innovations have you put in place that is actually working for you, as regards women assessing the tetanus vaccination and all other stuffs?

Interviewee:

For the ones that I saw, the people that are coming here for the anti-natal, we do give them advice, and they take the vaccines. When they are in labor, they will not have any problem. Then the delivery will go successfully. I have not experienced anything bad.

Interviewer:

Okay. So what type of health interventions have been the most successful in this community, you know, and why? Why have they been so successful? What health interventions have been very successful within this community? Do you understand what I'm trying to say? So I don't know. What type of health interventions have been the most successful in your community and why do you think they'll be successful?

Interviewee:

Well, whenever they come for anti-natal, we make them to be happy. We give them maybe- some times - we buy something for them. They will eat or we interact with them. Yes, after interacting with them, the way you communicate with them will make them to come for the anti-natal next time. This time, maybe, when they are coming, they will bring their sisters or their friends.

Interviewer:

Okay, so are there other interventions you have in mind?

Interviewee:

Well, through gifts, you can get them.

Interviewer:

So is there anything else you want us to know about Hepatitis B, sharing information about the Hepatitis B birth dose, is there any other thing you want to add?

Interviewee:

Hepatitis B first dose will help children to not contact the hepatitis B, it helps them to a boost their immunity, and it will make them to be strong, even when they see the person, they get in contact with the person having the disease, they will be able to combat it because they have received the first dose of diabetes.

Interviewer:

Okay. Thank you very much. Thank you for your time. So, like you rightly said, you don't have nothing to say you. You've concluded rightly. So want to thank you for your time. I will appreciate you so much for taking your time to really, really give us this insight into this study. Thank you very much. And from our end, we wish you well and we pray you continue that which you know how to do best. Thank you very much.

So for emphasis, we are still at [facility name]. So we've been talking with [name], the CHEW and the RI officer in this facility. Thank you very much.

TRANSCRIBED KII FOR COMMUNITY VOLUNTEER State 2, LGA 4, Facility 6

Interviewer:

Good afternoon. Ladies and gentlemen, we are live now at [facility name]. And we have with us a seasoned community volunteer, who have been in the service of directing people to assess healthcare from this facility, you know, from within the community. So as usual, I have with me in my team [name], and my humble [name]. Together, we will be administering this key informant interview. So, before we start, can we have your consent? Do we have your consent to continue with this interview?

Interviewee:

Yes Sir.

Interviewer:

Okay. Thank you very much. So can you please introduce yourself to our audience?

Interviewee:

Thank you, sir. I am [name], a committee volunteer in this community [facility name].

Interviewer:

Okay, so how old are you Ma?

Interviewee:

I am 35years old.

Interviewer:

So how long have you been in the business of a community volunteering?

Interviewee:

About five years now.

Interviewer:

Okay, five years. So Ma, having been in this business for like five years, I assume you have come in contact with so many persons; mothers and other categories? Ages alike. So what can you, What do you think are the biggest challenges for people living with hepatitis B in your community?

Interviewee:

Challenges they are having is that, atimes, you know in this our community, they don't normally like to visit hospitals. Because if you see any case like that, any person who is suffering from that hepatitis, they believe in this native doctors, even if you refer them to hospital do not go, they will tell you that in the hospital, they are not managing the case like that they prefer negative drugs to that hospital medicine

Interviewer:

So now, having noticed these challenges, and probably other challenges, what are the biggest needs? In your opinion? What do you think are the biggest needs to address Hepatitis B in the community?

Interviewee:

The is the need is that, you have to create awareness, through advocacy visits, and through this community mobilization, because atimes, they do hear the Igwe's. They call them for community mobilization they will come out in masses. And people like health care, all the healthcare providers should be there to tell them more about this hepatitis, the side effect, the signs and symptoms and so on. So they come out in mass to receive the vaccine.

Interviewer:

So is there a strong need for the Hepatitis B birth dose, have you heard of Hepatitis B birth dose before?

Interviewee:

Yes, sir.

Interviewer

Okay, so do you think there's a strong need for hepatitis B birth dose?

Interviewee:

Yes, Sir there is need for that.

Interviewer:

Why?

Interviewee:

Because, you know atimes, this Hepatitis B is transmissible, we can transfer from one person to another. And he can also transfer through semen of blood. So immediately a child is born, you have to give it to the child, the child to receive that vaccine to protect the child against that hepatitis B virus,

Interviewer:

Okay. So in your opinion, that is the reason why there is need for it?

Interviewee

Yes Sir.

Interviewer:

So do you think others see this need, you know, for hepatitis B, birth dose, the way you see it,

Interviewee:

No, its out ignorance, it's not their fault. But I believe as time goes on, people, we need it more.

Interviewer:

Okay. So in your opinion, you think they also see the need to have this?

Interviewee:

Yes Sir.

Interviewer

The same way you see it?

Interviewee:

Yes Sir.

Interviewer:

Okay. So can you tell me about current programs? Because your line of work as a committee, I believe you interface with the health facility?

Interviewee:

Yes Sir.

Interviewer:

Often?

Interviewee:

Yes Sir

Interviewer:

So can you tell me about current programs or practices for the hepatitis B birth dose, in your clinic, within this clinic that are in your attachment area?

Interviewee:

In terms of that program, they normally do it during immunization visit, because we normally have immunization on every Wednesday, that’s for fixed post ,other ones are outreach, on that Wednesday, people were taught on all these immunization diseases, both hepatitis and others, how it can be prevented , how, like to the days and types of reception, the time to receive it and when not to receive it. That’s all I know.

Interviewer:

Okay, So you know about immunization?

Interviewee:

Yes Sir.

Interviewer:

So is the Hepatitis B birth dose easy to get within 24 hours of birth?

Interviewee:

Yes Sir

Interviewer:

Why do you think so?

Interviewee:

Is easy because in Achara Ohodo we have solar, the vaccine is normally available all the time, is easy to get.

Interviewer:

So in your opinion now, who do you think and administer the hepatitis B birth dose?

Interviewee:

A health practitioner?

Interviewer:

Okay.

Interviewee:

A trained health practitioner, not everybody that knows the sight and limits of administration.

Interviewer:

Okay, so what are the challenges and barriers to getting Hepatitis B birth dose vaccines?

Interviewee:

The challenges? Like, it depends on the area, where the mother delivered the child, If the mother delivered the child in a health center, probably, the child must receive must that birth dose that time. But, when the mother deliver the child outside the health center, the mother will not come to the health center to receive the vaccine immediately. And also all this ignorance of ignorance, they may not have access to it.

Interviewer:

Okay. So is that the only challenge you know, about Hepatitis B birth dose vaccination?

Interviewee:

Unless if it is not available in the health center at that time, but once it is available?

Interviewer:

Ok so have you gotten information from participants? Because, your line of work, I believe you interface with people that come to the facility, Since you're a volunteer in this facility. So have you gotten information like feedback from participants regarding their experience with the hepatitis B birth dose?

Interviewee:

Yes, the information is that they normally like all these immunizable vaccine, especially this hepatitis, they will tell you that the child is not, the child is just too small to receive any vaccine'. And sometimes they will tell you that the thing causes fever. They don't want to give their child any vaccine at all now until the child grows. That's what they normally say about that Hepatitis B.

Interviewer:

So now in that slide coming from that angle now, could you shed more light on the community understanding of hepatitis B birth dose?

Interviewee:

Base on this, We normally do home visits, because at times, if you call them, they may not come to us they will tell you that they want to go to this market or this market. We schedule the for home visits, you go house to house to teach them on the importance of this hepatitis B birth dose,the signs and the causes of it. The benefits of it, the importance of it if they received the vaccine.

Interviewer:

So you go round to teach them?

Interviewee:

Yes, house to house during visit.

Interviewer:

So now, since you go around to teach them what to do you actually say is their understanding, do you How would you rate their own understanding of this hepatitis B birth dose?

Interviewee:

Some of them don't, there is, some of them don't understand it, like 50% of them don't understand, there are times after teaching them, they will understand that their mother-in-law will tell them later ''don't go oh, during their own time they didn't even receive any vaccine''. But, you know some of them are learned, they can even sneak to come to the health center and receive. Some of them do understand.

Interviewer:

So now also in that same angle. Do you think people are interested in getting the Hepatitis?

Interviewee:

They're interested.

Interviewer:

Okay. So, can you describe what kind of specific information or messaging you have heard about Hepatitis B birth dose? What information about the Hepatitis B vaccination or specifically birth dose? What kind of information or messaging Have you heard about it?

Interviewee:

About this hepatitis B? No, it is caused by Hepatitis B virus, it normally affect the liver. Ehen, once you receive that vaccine, it will produce antibody against the hepatitis B virus, thereby protecting the child from contacting that virus. And once you receive that vaccine it will protect you against that virus, and that virus, you know all these viral diseases they are deadly. And they cannot, anyway atimes they do say that there is no drug for viral diseases. You don't, we just treat them symptomatic, depending on the present symptoms but it's better, like one adage say prevention is better than cure. Its better you prevented disease done than to cure it.

Interviewer:

Ok, prevention is better than cure, I like that. So do you have any ideas or suggestions for improving Hepatitis B vaccination rates, you have any ideas you have suggestions you will have was on how we can best improve Hepatitis B birth dose vaccination rate, especially, maybe you would kind of want to say maybe break it down, maybe in the community, what suggestions would you have for us in the community, by giving, Because since you're close to the community, then maybe in the health system level, you also want to let us know what suggestions you have for improving Hepatitis B birth dose vaccination rate, and then maybe Finally, we can now look at the provider, like here a facility, we are this vaccination is being given? Do you have any suggestions for us on how best to improve vaccination rate?

Interviewee:

Yes Sir, the suggestion I have it can be improved through campaign, through community mobilization, through that awareness, creating awareness first, that's the first thing, and you have to create awareness for people to know the thing, the Hepatitis B

Interviewer:

It is community, or the health system or, or the provider,

Interviewee:

Community, both provider and the community. Because you the investigators, of this thing, you have to teach the providers the importance, and they will learn more. Because as you're teaching them, they are learning you're imparting that knowledge on them. And they will push that thing because we the providers are at the grassroots, we can assess them easily.

Interviewer:

Okay, so, in essence, you are talking about creating awareness, right?

Interviewee:

Yes Sir

Interviewer:

And then other stuffs just like you mentioned, okay, so are there specific strategies for education, or awareness that might be most effective. In your opinion, are there specific strategies you think that if you employ them in health education, or increasing awareness, that might be very effective?

Interviewee:

Yeah, There are some Strategies like little health talk during that immunization session, Antenatal session, that home visits, even that committee mobilization. Because you know, in terms of committee mobilization, you have to meet the Igwe. And the all these committee members, do you hear the cry of Igwe, if igwe called them for a meeting, for anything. Ehen, they will come out in mass to hear about that, and the thing can be achieved through that health talk.

Interviewer:

So, just for emphasis sake, you've mentioned this word twice ''Igwe'', who is Igwe?

Interviewee:

We have the Igwe, as in Igwe, like the leader, the leader of the community. Yeah, I don't know what again.

Interviewer:

Okay, the leader of the community, okay.

Interviewee:

Yes.

Interviewer:

Okay, Okay, So, can you tell me more about your own experience now, with maternal tetanus, vaccination?

Interviewee:

Maternal tetanus vaccination? Is important, ah this thing, anyway is not only for pregnant women, both women from childbearing age. That is from 15 to 45 years, because it will preventing the mother against tetanus. And once the mother receives that tetanus vaccine, there is no probability of risk that is happening that consider Tec nine that normally affect the mothers. And once you have the immunity against that tetanus, it will not transfer to your child, if you are pregnant. You know, atimes in our own local area, they don't normally like women of childbearing age, don’t normally receive that this thing, unless if they are pregnant, and if they are pregnant, you receive the first dose, let’s assume they

received the first dose today, after 28days they come back for second dose, after six months they come back for third dose, if they reach, but if you want to prevent it, if you want to receive it for life, you have to receive the third one after six months, after one year, you receive the fourth one, after one year again you will receive the fifth one and this one is for life.

Interviewer:

Wow, that's good to know. So now having said all that, so what do you think are the barriers or challenges, you know, you have experienced in addition to maternal tetanus vaccination? Are there challenges you have experienced?

Interviewee:

You know atimes they normally forget, that's their problem, even if they call for this Antenatal visit after teaching them, they will say, okay, they will come back and later you not see them. And if you ask them, they say they have forgotten, and they don't want to receive again because they're afraid Okay, they will not receive again. They have phobia of receiving rejection, most of them have phobia for that.

Interviewer:

Having stated all of these, do you have any success? What successes have you experienced? You know? And what is really working for you in this facility? And what are the things you're putting in place that is actually working on a new patient? What's your success story, in terms of vaccination, generally?

Interviewee:

These vaccination, more of them are coming. They do patronize this health center in terms of vaccination, many of them are receiving, and I have not seen all this cases of AFI, I have not seen that case, because, you know atimes if a mother comes to your clinic to receive vaccination, or any type of injection and we see access or any other thing, they will not come back again. But I thank God I have not seen that case since I came here.

Interviewer:

Okay. So what types of health interventions have been the most successful in your community? And why have they been so successful? Of course, you know, we have different health interventions. So what types have been very successful in your community? In your opinion? And why do you think they've been so successful?

Interviewee:

This immunization, they are successful, because you know atimes if they come here for immunization, franking speaking, we do chat, play, we do weighing of a thing, give them health talk based on family planning, and other things because we offering family planning services here. And even if they don't want to receive modern one, we can even teach them natural. This immunization is ok, family planning.

Interviewer:

So, is there anything else you think we should know about Hepatitis B, or sharing information about it or the Hepatitis B birth dose in particular, is there anything else you want to share or anything you think can help more information you want to share that?

Interviewee:

You know, this hepatitis of a thing, you know we have different types of hepatitis, you have A to E, we have oral and semen, that is, there is a one that your contact from figure oral and other one is blood or semen and the one that is prevented is the one that have the vaccine is hepatitis B which is bloody this thing.

Interviewer:

Okay, is that all?

Interviewee:

Solution this thing, based on the hepatitis B, normally affect the liver and that is the liver is the major organ inside our body that helps in detoxification of those things that we take and once we taking and the organ that is that normally detoxifies is affected it will affect the whole organ. And another thing based on this hepatitis is the signs and symptoms of it, we have yellowish of the eyes, yellowish of the urine, if you are urinating, your urine will be yellow. And also weakness, weakness yes, another aspect of it is that hepatitis B is high, is deadly, is deadly. But once you receive the vaccine, you can have immunity against it, even the adults we have adults one and have these children one. And another thing is that If you are a pregnant woman that come to our clinic for this thing you have to check your Hepatitis B status, both HIV and hepatitis B status for us to know that they have the virus.

Interviewer:

Okay. Thank you very much. So is that all? Is there any other thing you want to add? In terms of, is there any other thing you want to say?

Interviewee:

That's all I can say for now.

Interviewer:

Okay, thank you very much, Ma. It has been a wonderful moment sharing and gaining more insights from you, madam. So On behalf of the team, first we what to appreciate you for the timeout you know for giving us time, granting us audience to administer this interview to you. So be rest assured that your responses they won't be taking for granted they will actually have been reshaping, you know, and making informed decisions. Thank you one more time. So on behalf of the team and my colleague, [name]. We are over and out.

TRANSCRIBED KII FOR PREGNANT WOMAN State 2, LGA 4, Facility 6

Interviewer:

Good morning everybody, good morning ladies and gentlemen

Interviewee:

Good morning sir

Interviewer:

We are here live at [facility name] in [LGA name], here we have with us a pregnant woman, who are accessing her health Care by health facility which by name [name] and also as usual am here with my colleagues [name] and I my humble self, [name] we will be conducting this key informant interview of [name], before we start [name], pls,do we have your phone consent to administer this key informant interview to you?

Interviewee:

Yes sir

Interviewer:

For the sake of urgency, can you please tell us more about yourself? By way of introduction.

Interviewee:

Okay, Am [name], am from this [facility name] in [LGA name], in [state name]

Interviewer: So, how old are you ma?

Interviewee: Am 28years old

Interviewer:

Do you have kids?

Interviewer:

Yes

Interviewer:

Are you pregnant?

Interviewee:

Yes sir

Interviewer:

How old is your pregnancy now?

Interviewee:

Is now 3months

Interviewer:

Okay, Do you normally access health care in this facility?

Interviewee:

Yes

Interviewer:

Okay, we will be asking you some questions that centre on the service you access in this health facility, Birth and foremost ma, what did you know about hepatitisB ?

Interviewee:

All I know about hepatitisB Birth dose, the Birth time I saw it is on [state], I saw it when a particular child eye is yellowish in colour,I now ask the woman, what's happening to the child?The woman told me that time that the woman is having hepatitisB, I now asked the woman what causes this? She responded I don't know, till now I don't know what causes it, but I know hepatitisB B, the person that her child have it, his/her eyes will be yellowish in colour, that's all I know about hepatitisB, I don't know other things about it

Interviewer:

Have you heard about hepatitisB Birth dose before?

Interviewee:

Yes

Interviewer:

So, what did you know about hepatitisB Birth dose?

Interviewee;

hepatitisB Birth dose is it how they give the drugs?

hepatitisB Birth dose should be taken within 24hours of giving birth or two weeks after your birth

Interviewer:

So that's all you know about hepatitisB Birth dose?

Interviewee:

Yes

Interviewer:

And when the vaccine is given?

Interviewee:

Yes

Interviewer:

So how do you feel, about hepatitisB Birth dose? How does it make you feel?

The knowledge of hepatitisB Birth dose, how do you feel about it?

Interviewee:

How I feel is this, maybe is for prevention, may be the hepatitisB Birth dose is like a prevention so that the child will not contact the disease

Interviewer:

Have you ever ask for hepatitisB Birth dose before? either for your child or any other child, have you requested for it?

Interviewee:

No

Interviewer: was your child given hepatitisB Birth dose?

You said you have one child

Interviewee: yes, yes, is two because that time I have birth, my mum came down and ask me whether I gave my child hepatitisB Birth dose I told her NO and my child is abit weak that time and she take me to this place

Interviewer:

Okay, Now you are pregnant, do you plan to get hepatitisB Birth dose for your baby when you deliver

Interviewee:

Yes

Interviewer:

Why? Why do you plan to?

Interviewee:

Because, whole I plan is , I noticed that that time that is very necessary to get that hepatitisB Birth dose for child to take it

Interviewer:

So do you have any feeling? Now that you've planned it and you know the reason why you planned for it, do you have any feeling or concerns like excitement, how does it makes you feel? That you are planning that you child will be taking hepatitisB Birth dose when you deliver? Do you have any felling about it?

Interviewee:

Yes, I have

Interviewer:

Like what, what , be precise

Interviewee: What concerns about the hepatitisB well, I don't have anything concerning the Birth dose maybe the hepatitisB, I don't have anything now to say

Interviewer:

So you don't have any concern, So ma do you think hepatitisB Birth dose giving to children, do you think it works to prevent hepatitisB?

Interviewee: In my own understanding, I Believed that it helps

Interviewer:

Why?

Interviewee:

Because, I tell you that if I give my child the drug for hepatitisB it will prevent

Interviewer:

So do you think you could easily get, now that you have a reason for it and you believe it works do you think you can easily get hepatitisB Birth dose vaccine if you ask for it?

Interviewee:

Yes

Interviewer:

Will you ask for it?

Interviewee:

Yes

Interviewer:

Why, why will you ask for it

Interviewee:

Firth of all, I will ask when I need it, maybe if I want to inject my child for hepatitisB, I will come here believing that you will have it.

Interviewer:

Is that the reason why you will ask for it?

I want to know why, why do you really want to ask for your child hepatitisB Birth dose?

Interviewee:

To prevent it, so i will ask for it

Interviewer:

Is there anything that might come In the way like a challenge you might face related to accessing hepatitisB Birth dose?

Interviewee:

No

Interviewer:

No challenge?

Interviewee:

Yes

Interviewer:

Do you feel hepatitisB Birth dose is important for you to get for your child

Interviewee:

Yes

Interviewer:

Why do you feel so?

Interviewee:

Is very important, why is very important is, I will repeat it again, why is very important is that as have said before, if you give a child hepatitisB Birth dose am believing that the child will not suffer from that disease again that is the reason...

Interviewer:

So okay, looking at the in general perspective now, generally, do you think pregnant woman in your region, I mean this regional area, may be within this community or the state do you believe or think pregnant woman are taking hepatitisB Birth dose for there babies?

Interviewee:

Yes, I believe they will get it in there various places

Interviewer:

Do you think they are getting it?

Interviewee:

Yes

Interviewer:

So, what give you that confidence?

Interviewee: as far as health Care is concerned I believed they will have it there

Interviewer:

So you believe it will be available?

So do you have any suggestions? Do you have any suggestions for us, that may help us in educate people in the society about hepatitisB Birth dose, is there any suggestions you want to give us? On how best to educate people in the community about hepatitisB Birth dose

Interviewee: well, I don't have any suggestions about it as of now

Interviewer:

Is there any way you can suggest, things you think, the way you think we can go about it to educate woman like you in the community?

Interviewee:

I don't have but the only thing that I will say is that you people have to help the health centres when they are going for interview, you people will help us, like I am in this village now, you people will help us like the vaccine they are giving to us, maybe a mother that have pregnancy you will provide needed things, because people in the village they don't know those things, even as for me I don't know it before, but when I, like the former baby I give birth to when am having the experience, I don't know those things before, so am begging you people to help us having all those things here.

Interviewer:

So, we've gotten you, but we also want to know did you now prefer to learn about hepatitisB? How do you prefer to learn about hepatitisB? In what way do you think you can learn more about hepatitisB?

Interviewee:

Hmmm, like when am coming here, they will start teaching us about it, I believe they will help us, because is they are, they are, they are line and then help teaching us about hepatitisB and the rest of them and how your baby, they will ask you what a d what are you taking? They will ask you all those things, about what you are eating, are you eating frequently or not? I believed when they start coming here they will teach us those things.

Interviewer;

Are there other medium in which you think you can easily get information, espe I here within the community whether mediums or techniques or method or strategy that you thing pregnant women like you within the community can also easily access information about vaccination like hepatitisB

Interviewee:

Yes

Interviewer:

Can you share those mediums with us?

Interviewee:

Like Church, sometimes they will come here maybe church, maybe hospital, hmmmm

Interviewer:

Okay, thank you very much, so, how do you prefer to learn about health issues generally? For what success do you get, for emphasis sake, how do you prefer to learn more about hepatitisB or health issues generally? How do you think is a best way, you prefer to learn

Interviewee:

Hmmm, maybe as am coming here and learn more about hepatitisB that's number one, secondly if I can go to this Nsuka general hospital, like go there and register maybe through there I will learn more or maybe I will learn from my fellow woman, maybe some of them that knows about the hepatitisB.

Interviewer:

Okay, yea is that all?

Interviewee:

Yes

Interviewer:

Okay, is there any other things? So ehn, is there anything you thing we should know about hepatitisB, sharing information about hepatitisB or hepatitisB Birth dose anything you think we should know

Interviewee:

Like the question you ask me before, is something that I now remind through fliers , you can ask other questions

Interviewer:

So we can learn through fliers? So is there any other things you think we should know? Any other things you want to share with us, probably we don't ask about hepatitisB Birth dose or how we can be able to reach to women like you in the society, anything other things you want to add please

Interviewee:

Nothing else

Interviewer:

Thank you very much Madam [name], it has being a wonderful moment with you, we appreciate you for giving out your time to grant us this interview, we rest assure that the responses you've given now, we help us in making some decision and to chart our way forward we say thank you

Interviewee:

Thank you sirs

TRANSCRIBED KII FOR PREGNANT WOMAN State 2, LGA 4, Facility 6

Interviewer:

Good morning everybody, good morning ladies and gentlemen

Interviewee:

Good morning sir

Interviewer:

We are here live at [facility name] in [LGA name], here we have with us a pregnant woman, who are accessing her health Care by health facility which by name [name] and also as usual am here with my colleagues [name] and I my humble self, [name] we will be conducting this key informant interview of [name], before we start [name] pls,do we have your phone consent to administer this key informant interview to you?

Interviewee:

Yes sir

Interviewer:

For the sake of urgency, can you please tell us more about yourself? By way of introduction.

Interviewee:

Okay, Am [name], am from this [facility name] in [LGA name], in [state name]

Interviewer: So, how old are you ma?

Interviewee: Am 28years old

Interviewer:

Do you have kids?

Interviewer:

Yes

Interviewer:

Are you pregnant?

Interviewee:

Yes sir

Interviewer:

How old is your pregnancy now?

Interviewee:

Is now 3months

Interviewer:

Okay, Do you normally access health care in this facility?

Interviewee:

Yes

Interviewer:

Okay, we will be asking you some questions that centre on the service you access in this health facility, Birth and foremost ma, what did you know about hepatitisB ?

Interviewee:

All I know about hepatitisB Birth dose, the Birth time I saw it is on [state], I saw it when a particular child eye is yellowish in colour,I now ask the woman, what's happening to the child?The woman told me that time that the woman is having hepatitisB, I now asked the woman what causes this? She responded I don't know, till now I don't know what causes it, but I know hepatitisB B, the person that her child have it, his/her eyes will be yellowish in colour, that's all I know about hepatitisB, I don't know other things about it

Interviewer:

Have you heard about hepatitisB Birth dose before?

Interviewee:

Yes

Interviewer:

So, what did you know about hepatitisB Birth dose?

Interviewee;

hepatitisB Birth dose is it how they give the drugs?

hepatitisB Birth dose should be taken within 24hours of giving birth or two weeks after your birth

Interviewer:

So that's all you know about hepatitisB Birth dose?

Interviewee:

Yes

Interviewer:

And when the vaccine is given?

Interviewee:

Yes

Interviewer:

So how do you feel, about hepatitisB Birth dose? How does it make you feel?

The knowledge of hepatitisB Birth dose, how do you feel about it?

Interviewee:

How I feel is this, maybe is for prevention, may be the hepatitisB Birth dose is like a prevention so that the child will not contact the disease

Interviewer:

Have you ever ask for hepatitisB Birth dose before? either for your child or any other child, have you requested for it?

Interviewee:

No

Interviewer: was your child given hepatitisB Birth dose?

You said you have one child

Interviewee: yes, yes, is two because that time I have birth, my mum came down and ask me whether I gave my child hepatitisB Birth dose I told her NO and my child is abit weak that time and she take me to this place

Interviewer:

Okay, Now you are pregnant, do you plan to get hepatitisB Birth dose for your baby when you deliver

Interviewee:

Yes

Interviewer:

Why? Why do you plan to?

Interviewee:

Because, whole I plan is , I noticed that that time that is very necessary to get that hepatitisB Birth dose for child to take it

Interviewer:

So do you have any feeling? Now that you've planned it and you know the reason why you planned for it, do you have any feeling or concerns like excitement, how does it makes you feel? That you are planning that you child will be taking hepatitisB Birth dose when you deliver? Do you have any felling about it?

Interviewee:

Yes, I have

Interviewer:

Like what, what , be precise

Interviewee: What concerns about the hepatitisB well, I don't have anything concerning the Birth dose maybe the hepatitisB, I don't have anything now to say

Interviewer:

So you don't have any concern, So ma do you think hepatitisB Birth dose giving to children, do you think it works to prevent hepatitisB?

Interviewee: In my own understanding, I Believed that it helps

Interviewer:

Why?

Interviewee:

Because, I tell you that if I give my child the drug for hepatitisB it will prevent

Interviewer:

So do you think you could easily get, now that you have a reason for it and you believe it works do you think you can easily get hepatitisB Birth dose vaccine if you ask for it?

Interviewee:

Yes

Interviewer:

Will you ask for it?

Interviewee:

Yes

Interviewer:

Why, why will you ask for it

Interviewee:

Firth of all, I will ask when I need it, maybe if I want to inject my child for hepatitisB, I will come here believing that you will have it.

Interviewer:

Is that the reason why you will ask for it?

I want to know why, why do you really want to ask for your child hepatitisB Birth dose?

Interviewee:

To prevent it, so i will ask for it

Interviewer:

Is there anything that might come In the way like a challenge you might face related to accessing hepatitisB Birth dose?

Interviewee:

No

Interviewer:

No challenge?

Interviewee:

Yes

Interviewer:

Do you feel hepatitisB Birth dose is important for you to get for your child

Interviewee:

Yes

Interviewer:

Why do you feel so?

Interviewee:

Is very important, why is very important is, I will repeat it again, why is very important is that as have said before, if you give a child hepatitisB Birth dose am believing that the child will not suffer from that disease again that is the reason...

Interviewer:

So okay, looking at the in general perspective now, generally, do you think pregnant woman in your region, I mean this regional area, may be within this community or the state do you believe or think pregnant woman are taking hepatitisB Birth dose for there babies?

Interviewee:

Yes, I believe they will get it in there various places

Interviewer:

Do you think they are getting it?

Interviewee:

Yes

Interviewer:

So, what give you that confidence?

Interviewee: as far as health Care is concerned I believed they will have it there

Interviewer:

So you believe it will be available?

So do you have any suggestions? Do you have any suggestions for us, that may help us in educate people in the society about hepatitisB Birth dose, is there any suggestions you want to give us? On how best to educate people in the community about hepatitisB Birth dose

Interviewee: well, I don't have any suggestions about it as of now

Interviewer:

Is there any way you can suggest, things you think, the way you think we can go about it to educate woman like you in the community?

Interviewee:

I don't have but the only thing that I will say is that you people have to help the health centres when they are going for interview, you people will help us, like I am in this village now, you people will help us like the vaccine they are giving to us, maybe a mother that have pregnancy you will provide needed things, because people in the village they don't know those things, even as for me I don't know it before, but when I, like the former baby I give birth to when am having the experience, I don't know those things before, so am begging you people to help us having all those things here.

Interviewer:

So, we've gotten you, but we also want to know did you now prefer to learn about hepatitisB? How do you prefer to learn about hepatitisB? In what way do you think you can learn more about hepatitisB?

Interviewee:

Hmmm, like when am coming here, they will start teaching us about it, I believe they will help us, because is they are, they are, they are line and then help teaching us about hepatitisB and the rest of them and how your baby, they will ask you what a d what are you taking? They will ask you all those things, about what you are eating, are you eating frequently or not? I believed when they start coming here they will teach us those things.

Interviewer;

Are there other medium in which you think you can easily get information, espe I here within the community whether mediums or techniques or method or strategy that you thing pregnant women like you within the community can also easily access information about vaccination like hepatitisB

Interviewee:

Yes

Interviewer:

Can you share those mediums with us?

Interviewee:

Like Church, sometimes they will come here maybe church, maybe hospital, hmmmm

Interviewer:

Okay, thank you very much, so, how do you prefer to learn about health issues generally? For what success do you get, for emphasis sake, how do you prefer to learn more about hepatitisB or health issues generally? How do you think is a best way, you prefer to learn

Interviewee:

Hmmm, maybe as am coming here and learn more about hepatitisB that's number one, secondly if I can go to this Nsuka general hospital, like go there and register maybe through there I will learn more or maybe I will learn from my fellow woman, maybe some of them that knows about the hepatitisB.

Interviewer:

Okay, yea is that all?

Interviewee:

Yes

Interviewer:

Okay, is there any other things? So ehn, is there anything you thing we should know about hepatitisB, sharing information about hepatitisB or hepatitisB Birth dose anything you think we should know

Interviewee:

Like the question you ask me before, is something that I now remind through fliers , you can ask other questions

Interviewer:

So we can learn through fliers? So is there any other things you think we should know? Any other things you want to share with us, probably we don't ask about hepatitisB Birth dose or how we can be able to reach to women like you in the society, anything other things you want to add please

Interviewee:

Nothing else

Interviewer:

Thank you very much Madam [name], it has being a wonderful moment with you, we appreciate you for giving out your time to grant us this interview, we rest assure that the responses you've given now, we help us in making some decision and to chart our way forward we say thank you

Interviewee:

Thank you sirs

TRANSCRIBED KII FOR HF OFFICER IN CHARGE
State 2, LGA 4, Facility 8

Interviwer:

Good afternoon, ladies and gentlemen we are here live at [facility name], where we will be interfacing and conducting a key informant interview about hepatitisB vaccination on[name] the OIC of [facility name], with me here I have my colleague doctor [name] and I my humble self [name], together we'll be conducting this key informant interview. So madam [name], before we start I will like to get your consent if we should go ahead with this interview on record.

Interviewee:

Yeah, you are free

Interviewer:

Okay, please I would like you to speak up a little so we can get you.

Interviewee:

Ok, you are free

Interviewer:

Okay, Thank you very much, So can you tell us a little about yourself?

Interviewee:

My name is [name]. N. am aged 45 years

Interviewer:

Thank you very much, I will also please ask one more time that you speak up so that we can really get your feedback on the recorder. So Madam [name] you are the OIC of this facility, So for how long have you been the OIC of this facility?

Interviewee:

For three years now

Interviewer:

So maa in this period that you have been an OIC what would you say are the biggest challenges for people living with hepatitisB within the community? The biggest challenges they face?

Interviewee:

Well, for me I can say that the biggest challenge are

1. Lack of knowledge. At times there is suffering that they don't know what is happening to them and at times you be telling them, this is a problem, This is the problem. They will not understand it. They would prefer going to a chemist to get their drug, instead of going to the health facility and do test.

Interviewer:

So having noticed these challenges, like you rightly say, what are the biggest needs now you've seen, the biggest challenges, so what are the biggest needs in your opinion to address hepatitisB challenges in your community. What do you think you need to address these challenges?

Interviewee:

I believe that they need health education through their town announcers and their community leaders

Interviewer:

Okay. Well, health education. So, so is there a strong need for the hepatitisB Birth dose?

Interviewee:

Yes, because it is a very good preventive measure at the best, like Igbo people, you know, if you give birth to a baby and someone came to see the baby and say, no, no, don't put these baby, They will feel that the person didn't recognize the person rejected the person. So you would like to touch the baby and you don't know who has the infection, because this hepatitisB is an infectious disease, We don't know the person that have the infection, And if you didn't vaccinate the baby against the hepatitis B disease, if the person that have the disease touch the baby or even the baby inhales it from the carrier, the baby may contact the disease.   So that it is, so you have to protect the baby before going to the village

Interviewer:

Okay. So I got, I've got that. So do you think others actually see the same need for hepatitis B Birth dose too? Just as you're seeing it, Do you think others are seeing the same need?

Interviewee:

Yes I believe so. Because, many at times when we went to workshop at our local government they always hammering it even on radio and other places, they always hammering about immunization , So I believe you should know that is very important.

Interviewer:

So when they hammer on it, do they actually hammer on the Birth dose?

Interviewee:

Yes,

Interviewer:

Okay. So can you tell me about current programs or practices for hepatitisB Birth dose within your clinic here within your health facility?

Interviewee:

In the current programs, this is it, any person or any mother that delivers in this case facility? Our first thing is to immunize the baby, with hepatitis vaccine before the baby went back to the village to give the person a maximum protection. Then anyone that came from the village we have to give the vaccine to the person are the first contacts, And we always announce at the churches, and using the town announcer to announce

Interviewer:

Okay. So, so those are practices. So what about programs? Are there ongoing programs that also support what you do that support vaccination of hapetitisB birth dose programs, health programs that you all conduct within this facility?

Interviewee:

We carried it out using the town announcer and conducting Immunizations every week

interviewer:

Conducting immunization every weeks. Okay. So, is hapetitisB dose easy to get within 24 hours of birth?

Interviewee:

Yes

Interviewer:

Why?

Interviewee:

We have our functional solar and he first is dead witty. When the thing is about, so let's say we have only one bottle or two bottles, then we will go to local government and request for another one

Interviewer:

So who can administer hepatitis B first dose

Interviewee:

Here, we all the health workers can administer it

Interviewer:

So you can do it

Interviewee:

All of us are trained to do that

Interviewer:

So what are challenges or barriers to getting hepatitis B first dose vaccine? Are there challenges? Are there barriers to getting the vaccine?

Interviewee:

No

Interviewer:

there is no?

Interviewee:

I don’t think so

Interviewer:

Okay. So, so have you gotten information from participants in this in this regard, I mean, people that would be vaccinated, right. Or people whose children have been vaccinated with hepatitis B first dose, Have you gotten any feedback from them or any information from them regarding their experience with hepatitis B first dose?

Interviewee:

No, they are happy experience, whenever they came for immunization, for my humble self, So when they came in for immunization, we do ask questions how do you see these? what information do you know about this thing? Do you know about this? No, they are always complaining that they are okay about it

Interviewer:

So, so what is the community understanding in your perspective? What is the understanding of the community? As regards appetite B first dose? what is your understanding of the community

Interviewee:

There believe is they understand it has the first injection giving to child after birth, to give the child protection against hepatitis diseases

Interviewer:

Okay. That's the general understanding of the community, so as people interested in getting the first dose?

Interviewee:

They are very interested

Interviewer:

Can you describe what kind of specific information or messaging that you have heard as the healthcare worker? What kind of specific information or messaging about hepatitis B first dose that you heard?

Interviewee:

Wow. The information I've heard about hepatitis B first dose is that is immunization given to a child immediately after birth to protect the child against hepatitis infection

Interviewer:

That's all you've heard about. That's all the messaging you've heard

Interviewee:

and it's very good essentially, because it protect the child against hepatitis diseases

interviewer:

Okay. So do you have any ideas or suggestions for improvements, you know if we are to improve hepatitis B vaccination rates from where it is currently to probably somewhere higher. Right. So, what would you suggest to us? What kind of suggestions or ideas would you sell to us on how to go out of it?

Interviewee:

Eh, can say that we need more sensitization to the villagers and even to the health workers, at times, health workers need training, they need training to train more people not only hepatitis but all immunization also the villagers they need sensitization they need to know more about this hepatitisB through there town announcer, traditional rulers, churches and opinion data.

Interviewer:

Okay. Well, so so now if we're to, you've made some selling points here now, but however, if we're to break it down into different levels, right? If you have to suggest on way to improve hepatitisB first dose at different levels. How would you suggest to us that we can do that at the community level Because I want to get your, feeling about what to do at the community level, at the health system level, and then at the service provider level, because, you know, we have of course, you know what I mean, by the health system level, within the health structure, And then now at the provider level here that you are providing this care, what suggestions would you give on how to improve hepatitis B first dose vaccination

Interviewee:

On Provider level, my own suggestion is that the hepatitis vaccine supposed to be available at all moments, it ought to be available at all moment, to enhance the giving of the vaccination ,Because when the vaccination is not there, we cannot give what is not there So I believe if the vaccine is there. It will helped the health workers whenever clients come to the facility,

Interviewer:

At Health care system level?

Interviewee:

At health care system level, is almost the same things, we make sure all the key equipment like core chain equipment and treatments are available because if they are not available the things affect the system, just although this is a failure because when it is not available, when there is no functionable fridge for solar the thing affect the vaccination program. So I believe there must be an adequate solar or a refrigerator Or plants, Because when there is no solar, you are using the refrigerator, if there is no electricity the thing will also affect the core chain system. So at the community level, we are to het the information, telling the community member this is the importance of this vaccine, this is the importance

of this vaccine, tell them the importance of the vaccine, so that they will come to the health facility So tell them that the vaccine is available at all moment because it will be an annoying things when they come for the vaccine and the vaccine is not available, tomorrow if you are calling them, they may not be there.

Interviewer:

So are there specific strategies for education or awareness that might be most effective? In your opinion you mentioned strategy of education for awareness creation

Interviewee:

The strategy is this if it is possible, eh, if it is possible, let the vaccine be available. And also using the, the leaders to tell the members of the community about the importance of the vaccine.

Interviewer:

By leaders, what do you mean? Could you be a bit specific?

Interviewee:

What i mean by the leaders is traditional leaders, if the traditional leaders talk to the community, they will listen to them, So if I believe if the traditional rulers or opinion rulers talk to the people of the community about the important of particular program, they will understand it and follow his step, And when the program is always available, I think the people will always appreciate it.

Interviewer:

Okay. Thank you very much. So can you tell me about your own experience now? There've been a little, can you tell me about your experience with maternal tetanus vaccination?

Interviewee:

Maternal tetanus vaccination is a vaccination given to a pregnancy mother or any child bearing mother from the age of 15 to 45 years. If the mother is pregnant once you give is only two times during the pregnancy, but if the mother is not pregnant, if somebody wants to take it at lifetime it will be five times, whether the person is pregnant or not , should be 5 times provided that the person is always following the steps

Interviewer:

So what is your experience in this health center? What has been your experience with maternal tetanus vaccination?

Interviewee:

Yes. In this health center, it is always available. We always give it to a pregnant mothers then those that are not pregnant for those that want to take the lifetime, they'll always be there. And the pregnant mother always taking it.

Interviewer:

Okay. So what barriers or challenges have you experienced related to maternal tetanus vaccination? What are the challenges you've been experiencing or barriers as regards to maternal vaccination?

Interviewee:

The only challenge have ever witness is there is Sometimes we get out of stock of the vaccine, So when the mother came and we told them there is no vaccine, they will not be happy after that, you cant see another challenges,

Interviewer:

So have you mentioned some challenges that you have, so what are your successes? What successes have you experienced? What is really working for you? What are you doing differently that you think is working for you as regards to maternal tetanus vaccination?

Interviewee:

There is nothing specials that I did that enhance the success but well, I always have my drugs, the drugs are there. So whenever they came, they would just take the drug, the availability of the drugs and the equipment for the drugs, like needles, syringes. So

Interviewer:

What types of health interventions have been the most? Of course you understand what I mean by health intervention, Right? so what types of these interventions have been the most successful in your community? And why have they been so successful?

Interviewee:

Well, like immunization, not only for this tetanus or hepatitis or immunization organizations, it's very, very successful in this community. The reason is that we, the health workers, we are trying our best, we are going for advocacy, community mobilization and community diagnosis too. Then we always make sure that the drugs are available because when you tell them to come to the health center and receive drugs and they come but there is no drugs, there is another problem, so we make sure the drugs are available at all moments. And thank God we have functionable solar so we make sure that the solar is there and is drug is in good condition.

Interviewer:

Oh, okay. Well, is there other things you want to add, is there anything you want to you think we should know about hepatitis B and information you want to give us, Probably anything you have heard or about hepatitis B that you want to tell us, but that maybe I may have missed there's something else you want to chip in that you think is very of important. Do you think he's going to be a importance?

Interviewee:

I don't think there is anyone or anything we missed

Interviewer:

There's nothing you want to add, suggestions or anything?

Interviewee:

My suggestion is government has to improve in immunization like provision of core chain system, provision of refrigerators, provision of generators to hospitals to enhance good core chain system, then also this announcements on radios and television ,They have to give proper announcements, So people so that people will learn and also training of health workers, I believe it will work well.

Interviewer:

Well, thank you very much, madam Nnadi Fidelia You've been of immense help this afternoon on behalf of my colleague. I want to say thank you for making our time to grant us audience for this interview and be rest assured that all your inputs and all your opinions and the responses are been well noted. And I believe it's going to guide, help guide decision-making for further programs and activities. Thank you once more for me and my colleague say, thank you all, bye.

Interviewee: thank you very much

TRANSCRIBED KII FOR EZI UKEHE PHC PREGNANT WOMAN

INTERVIEWER

Good afternoon, ladies and gentlemen. So we are at Eziukehe Health Center. We are we are about to interview a pregnant mother that assesses health care in this facility by name, [name]. I am here with my colleague [name] and my humble self [name]. So together we will be administering this key informant interview. So before we start Madam odogbo Deborah, do we have your consent to conduct this interview on the record?

INTERVIEWEE

Yes.

INTERVIEWER

Thank you very much ma. So please Madam, can you just introduce yourself to us.

INTERVIEWEE

My name is [name], I'm from Eziukehe.

INTERVIEWER

Okay, so [name] how old are you please?

INTERVIEWEE

Am 32 years old.

INTERVIEWER

Do you have kids?

INTERVIEWEE

I have three kids?

INTERVIEWER

Okay, so how old is the youngest?

INTERVIEWEE

The youngest is 11 years.

INTERVIEWER

The youngest of your kids is how old?

INTERVIEWEE

The youngest?

INTERVIEWER

The smallest?

INTERVIEWEE

Five years, Five years.

INTERVIEWER

Okay. Thank you very much. So Madam Odogbo what do you know about hepatitis B?

INTERVIEWEE

Hepatitis B is discoloration of the eyes.

INTERVIEWER

Any other thing you know about it, what else do you know generally about the disease?

INTERVIEWEE

It is a disease that is effecting the liver.

INTERVIEWER

Okay, thank you very much. So what do you know about Hepatitis B birth dose.

INTERVIEWEE

We were given hepatitis B at birth as zero dose?

INTERVIEWER

Okay, so how do you feel, how do you feel about the hepatitis B birth dose? How do you feel about the hepatitis B birth dose? How do you feel?

INTERVIEWEE

It is normal, to give the children at birth.

INTERVIEWER

Okay, so have you ever asked for hepatitis B birth dose before for your children? Have you ever asked for hepatitis B birth dose to be given to your children?

INTERVIEWEE

Yes.

INTERVIEWER

Okay. Where was that? When and where did you ask for it?

INTERVIEWEE

At birth

INTERVIEWER

Okay

INTERVIEWEE

Because when I deliver the children at birth I will like to give to children because it's very important but the government know the important of it, to give children at that birth dose. Whenever i deliver, i will like to give it because it is important. That is why government said that we should give the child hepatitis B at birth dose.

INTERVIEWER

Okay. So have you, do you have any plan to get, you are pregnant now right? So do you have any plan to get Hepatitis B birth dose for your baby?

INTERVIEWEE

Yes.

INTERVIEWER

Why

INTERVIEWEE

I would like when I deliver, I will give my child at birth because it is very important.

INTERVIEWER

So do you have any concerns or stress or excitement, do you have any concern about giving children Hepatitis B birth dose at birth. Do you have any concern? Is they any reason or anything you have or stress about it?

INTERVIEWEE

Is not much stress because it's good. When you have a child, you give her at the birth. It will work, it will help the child growth.

INTERVIEWER

So do you think the hepatitis B birth dose that is given to children works? Do you think it works and prevent hepatitis?

INTERVIEWEE

Yes it works because if it does not work government will not allow us to give it to our children, and many children that have taken it, i didn't see any reaction in them. Whenever I deliver my child I will carry my baby to health center and give her because it is good.

INTERVIEWER

Okay. So do you think you could easily, do you think it is easy to get Hepatitis B birth dose vaccine if you ask for it? Whenever you ask for it, do you think it's easy?

INTERVIEWEE

Yes.

INTERVIEWER

Okay. So will you ask for it?

INTERVIEWEE

I would ask

INTERVIEWER

Okay, why will you ask for it?

INTERVIEWEE

Because it's very important.

INTERVIEWER

Okay, so you ask for it because it's important.

Mrs. Odogbo Deborah

Yes.

INTERVIEWER

Okay. So is there anything that might come in the way you know, like a challenge you might face related to assessing Hepatitis B? Is there any challenge in your own opinion? Is there anything you feel that can act as a hindrance or a challenge or a stumbling block to assessing Hepatitis B birth dose?

INTERVIEWEE

Anything that will make, if it is the child is not healthy i will not come and give her the dose.

INTERVIEWER

Okay, so for you the health of the child is actually is predominant. Okay, so do you feel the hepatitis B birth dose is important for you to get for your child?

INTERVIEWEE

Yes, it is very important.

INTERVIEWER

Why do you feel so?

INTERVIEWEE

It is important because when you come to health center, they say it is in health center. If it is not important then all this health center will not have it. And whenever you come the fridge is there, it is in

the fridge. I will like to come and give it because it is good. If it is not good, they will not put it in that fridge.

INTERVIEWER

Okay, so if it is not good they will not put it in the fridge. Okay, so in general now looking at General perspective, you are from this community right?

INTERVIEWEE

Yes

INTERVIEWER

So in general, do you feel, do you think other pregnant women in this region, in this area? Do you think there also feel the same way about hepatitis, giving their children Hepatitis B birth dose the same way you feel?

INTERVIEWEE

Yes, because when we are coming to ante-natal, the Nurses is telling them the importance of immunization. And everybody will say that if you come to ante-natal, you hear what the nurses are telling you. It is good that the nurses are telling you to the reality.

INTERVIEWER

Good. So what gives you that level of confidence? What makes you, what gives you confidence about hepatitis B birth dose or other women within the community are actually seeing it, valuing it the way you are valuing it? What gives you that confidence?

INTERVIEWEE

It is the Nurses that are working, because they know what they are doing. If they are not working well you will not see pregnant women, but they are working well. Whenever we come, they teach us and we are happy to go and next time if am coming i tell neighbors so we follow and receive the thing.

INTERVIEWER

So now based on what you've said so far, do you have any suggestions for us? If we want to educate the people in your community? Maybe we want to educate people in this community about Hepatitis B. birth dose. What would you suggest? How do you think we can go about it? What is the best way in your opinion that we can go about educating people, women like you within the community?

INTERVIEWEE

You know that a pregnant woman, you will educate them but you know sometime if you give them something, anything you give to them, they will like it. But whenever she know that if you come they will give you something they will like to come.

INTERVIEWER

Can you break down something, can you break down what you mean by giving something for our audience to understand it better?

INTERVIEWEE

Some will give bag, some will give tissue, and some will give soap. If you give anything that will, even those that will collect bag. Whenever they hear that they will give them something, if you collect bag you will want to come out.

INTERVIEWER

So would you prefer to learn about Hepatitis B? How will you prefer to learn about a hepatitis B? What is the best way you think we can actually reach out to people like you, or probably others that are pregnant within the community about information about hepatitis B?

INTERVIEWEE

Is through nurses and town announcer.

INTERVIEWER

Okay. So how do you prefer to learn about, what about general health issues, issues concerning your health, what is the best way you could prefer that we can reach you better with information about it.

INTERVIEWEE

In the village roads is good but we do not have radio. Through the Nurses and the town announcer, because you may be cooking at your kitchen. You will hear the town announcer announcing. If you are cooking you may stop. If the children is making noise, you tell them stop, you just listen at the town announcer. What the man is saying. It will help you to know because radio is good but we do not have radio.

INTERVIEWER

So ma, it has been a wonder moment but before we end this interview i will like to know, is there anything else you think we should know about like the hepatitis B? And in terms of information sharing within the community about hepatitis B, and then probably the administration of hepatitis B, is there anything that you want us to know? Is there any other thing, insights you want to share? Or you want to pick for the audience listening to you right now? Is there nothing you want them to know about hepatitis b?

INTERVIEWEE

Is good, but you know that, Some people we come but when you have time to come to village square and make the village come out, that some people are coming to address you people on hepatitis B. some will come, maybe man or woman or most children will come but if it is health center we say it is pregnant women and nursing mothers will come to health center. But if it's the village square everybody will come and hear what you are saying. You know that most men like to beer, where that they are drinking wine, if the man hear in the village square they may go there and discuss it, those that are not they will learn and it will help to go on.

INTERVIEWER

Thank you very much, Madam Odogbo Deborah. It has been a wonderful time with you and we appreciate you so much for making time to answer all these questions, all the answers you have given have been taking note of and I believe that it will help us to make more informed decision. So and we say thank you very much. God bless you, ma. So from the team, thank you. Over and out.

Interviewee:

Thank you

TRANSCRIBED KII FOR PREGNANT WOMAN State 2, LGA 4, Facility 10

Interviewer:

Ehm, Good morning everybody, so We're here at [facility name] to conduct a key informat interview for one of the pregnant mother that access health Care within this facility, Eh, this afternoon I'm here with my colleague [name] and my humble self [name], so we will be interviewing [name], One of the pregnant women that access care in this facility. So madam [name] before we start, we will like to get your Consent to go ahead with this interview, Do you give, or have your consent to go ahead and ask you this questions on records?

Interviewee Response: Yes.

Interviewer:

OK, thank you. So can we get to know you, can you introduce yourself to us?

Interviewee Response:

Your welcome, my name is eh [name], I am a 35 years old woman with four (4) kids

Interviewer:

OK, are you currently pregnant

Interviewee Response: Yes

Interviewer:

So How old is your pregnancy

Interviewee Response:

I am five (5) months pregnant

Interviewer:

Have you been accessing health Care here in this facility

Interviewee Response: yes

Interviewer:

OK thank you very much. So without waisting much of your time madam [name], I will like to know, what do you know about Hepatitis B

Interviewee Response

Ehm ehm, the little I know about Hepatitis B is the first immunization given to a child immediately after birth to prevent the child from contacting Hepatitis B.

Interviewer:

OK, so now your talking about the vaccine, so what about the disease itself, what do you know about the Disease

Interviewee Response

Hepatitis B is an infection that normally infect a child in uterus after birth.

Interviewer:

OK, thank you very much, so what do you know now about hepatitis B birth dose, what is your knowledge about Hepatitis B birth dose

Interviewee Response

Hepatitis B birth dose is the injection given to a child immediately after birth.

Interviewer:

OK, OK immediately after birth, so how do you feel about hepatitis B birth dose, how do you feel about it

Interviewee Response

Eh**,** what I feel about it, the injection is very very important immunization because of the work it has on the child

Interviewer:

OK, OK so have you ever asked for hepatitis B birth dose before for your children or other children

Interviewee Response:

Yes

Interviewer:

When was that

Interviewee Response:

I ask of it, when ever I deliver, i normally ask the nurse when I'm to come for it

Interviewer:

OK, so do you plan to get hepatitis B birth dose for tho baby, the one you are carrying now

Interviewee Response:

Yes

Interviewer:

OK, so why will you want to do that

Interviewee Response:

Ehm is because I want, I want my children to be prevented against this Hepatitis B

Interviewer:

OK, so haven't had this plan now, do you have any concern, any thing that is a concern that you wish to share with us about hepatitis B, both the vaccination, the birth dose or is there any excitement about it, any feeling or is there any stress you have around hepatitis B birth dose

Interviewee Response:

Eh**,** the excitement I have of it because of the preventive method it has on my children

Interviewer:

OK, so you're excited because it going to prevent you're children

Interviewee Response:

Yes

Interviewer:

Ok, so do you think the hepatitis B birth dose works to prevent hepatitis B. Do you think it works

Interviewee Response:

Yes, its works

Interviewer:

OK, so why do you think so

Interviewee Response

Yes because the rate children dies this days is not as before, because of this hepatitis B. The government has given us. Its prevent death of children, rapid death rate.

Interviewer:

OK, OK so do you think you could easily get the hepatitis B birth dose vaccine if you ask for it

Interviewee Response:

Yes

Interviewer:

OK, so will you ask for it

Interviewee Response:

Yes I will ask for it

Interviewer:

Why will you ask for it

Interviewee Response:

Yes, because of the important of it in my children

Interviewer:

OK, so is there anything that might come in the way or post a challenge that you may face related to accessing birth dose for hepatitis B, is there any challenge or problem you may face

Interviewee Response:

The challenge is maybe if I come to a clinic and I I'm not able to meet any of the health worker, it will be a challenge because I'm not able to get it at that moment, I will feel sad.

Interviewer:

OK, so do you feel that hepatitis B birth dose is important to get for you're child

Interviewee Response:

Yes

Interviewer:

Why, why do you feel so

Interviewee Response:

Because of it importance on the children

Interviewer:

Because of the importance to your children, wow so speaking generally now, do you feel pregnant women in you're region, I mean within this region of yours and you feel the get hepatitis B birth dose to their child

Interviewee Response:

I can't say for any other person, though since I've been coming here for ante-Nata in this health center, the nurse I meet here, they're working hard, very very hard to make sure that immunization is given to children

OK, including hepatitis B

Interviewee Response:

Yes

Interviewer:

OK, so what gives you that level of confidence that this people you know are actually hard working, and that their hard work is equally going to help to immunized the children, what gives you that confidence

Interviewee Response:

What gives me that confidence is because immunization is perform almost weekly, on weekly basis here in the center I visit

Interviewer:

Ok, so now with this confidence that you have, do you have any suggestions for us, we will like you to suggest for us like what we can do, like what we can educate your people in your community about hepatitis B birth dose, what do you suggest

Interviewee Response:

I suggest that the government should try to give all information on hepatitis B on radio and television, even on, maybe in churches and schools, we announce to them about it

Interviewer

OK, in televisions, schools and churhes, OK so will you prefer to learn, I what way now will you prefer to learn about hepatitis B, like in what strategies you as a person, how will you prefer to learn about hepatitis B, in what way will you prefer to be taught, ah don't know if you get my question, there are so many ways one can learn about it, so many ways, so what ways will you prefer

Interviewee Response:

by giving health talk, by giving more health talks on it, I will learn better

Interviewer

OK, so what about general speaking about health issue's generally now, what ways and avenues do you used to learn more about health generally

Interviewee Response:

OK, we use television, radio or televisions and radio in shot.

Interviewer

OK television and radio you can used to learn more about hepatitis B. So is there anything else you will like us to know, you think we should know about hepatitis B,. Seeing that this program is been cantered in hepatitis B, is there anything else, and key information you think you can give us, any insight you can give us about hepatitis B expercially as it relate to dissemination of hepatitis B information within the community and also administration of birth dose of hepatitis B, is there any you wi love to share with us

Interviewee Response:

Not really..

Interviewer

OK you have exhausted everything you think we should know

Interviewee Response:

Yes

Interviewer

OK, we appreciate you so much for finding time in granting us this audience, so be rest assured that your responses have been well noted and will be put into good used and will also help shape and make inform decisions. Thank you very much once more, thank you I'm out.

TRANSCRIBED KII FOR MIDWIFE State 2, LGA 4, Facility 10

Interviewer

Good afternoon, ladies and gentlemen. We are here at [facility name]. In ward three, we are actually going to be having a key Informat interview with the nurse midwife in charge of the facility by [name]. I'm here with my team, [name] and [name] of the state Primary Health Care Development Agency. My name is [name]. So we'll be putting together this interview. Good afternoon once more madam. For emphasis sake can you just introduce yourself one more time

Interviewee

Okay, my name is [name], Nurse mid-wife heading [facility].

Interviewer

So how old are you madam

Interviewee

I am 34 years

Interviewer

So how long have you been doing this job

Interviewee

This is 12 years

Interviewer

Okay, so.madam, Before we proceed on, we want to get your consent, Before I start this interview, we actually have some questions that revolve around Hepatitis B with its application and its management. And I believe you've heard about the hepatitis B birth dose, birth dose of hepatitis B, I want to get your consent to go ahead with this interview, may I have your consent to answer this question.

Interviewee

yes

Interviewer

okay, thank you very much. Okay So for starters ma, what are the biggest challenges for people living with hepatitis B, in your community

Interviewee

challenges

Interviewer

Yes the biggest challenges you ever encounter for people living with hepatitis B, I guess, you know people living with hepatitis B as a disease itself, So what are the biggest challenges

Interviewee

The biggest challenges is that we don't have a big hospital that will be managing those patients and at times referers places is too far from this our locality. So that is the problem we're having And we don't have doctors in our facilities that will be managing those cases.

Interviewer

Okay. So having noticed your challenges, so what would you say are the biggest needs to address these challenges

Interviewee

Our need is to a build cottage hospital or otherwise General Hospital and I apply doctors and qualified nurses, even CHO's an senior CHEWS or something like that. So that we can have proper management, because delay in management can cause death.

Interviewer

Okay. So, are there strong needs for hepatitis B birth dose, Do you think there is a strong need to have hepatitis B birth dose in the community

Interviewee

Exactly

Interviewer

Why, why would you say as to why it is important,

Interviewee

It is important, like zero Dose, it has no function, but after that zero dose and Hepatitis B one, then the the, the immunity will now activate and work very well.

Interviewer

I think it's you actually know about hepatitis B vaccination, and you know, somehow not to be empty anyway, but you know that it's either you give it a birth, or then if you miss it at birth yiu could give it some other time, say two weeks, six weeks, okay. So at this emphasis we are actually looking at giving it at birth, that why I mean birth dose so most of the questions I will actually be asking you will revolve around giving hepatitis B at birth.

Interviewee

yes, it is very, very important to give it at birth or before or after two weeks, immediately the baby is after two weeks born, we should wait till penta one because it will not complete the the time.

Interviewer

Okay, so, is there a strong need from your perspective, do you see a strong need for hepatitis B birth dose to be given, you think that is?

Interviewee

Very, very important for the prevention, Yes.

Interviewer

Okay.do do others, in your opinion. You think others actually see the same need that you you know,you know that there is need for it, do you think others actually still see the same need of giving Hepatitis B at birth dose to be given

Interviewee

Exactly

Interviewer

why, why would you say that

Interviewee

we are giving a health talk to mothers,

Interviewer

okay

Interviewee

Yes, we give health talk to them all the time of RI or even when we are doing home visits on mobilization So Some mothers knows that the importance of giving Hepatitis B birth dose

Interviewer

Okay, so, can you tell me more about current programs, you know, current programs or practices for the hepatitis B birth dose in your clinic, in this your facility, can you tell me more about programs that are ongoing programs that you do in this facility or probably practices things you do, that actually tends to promote Hepatitis B first dose vaccination.

Interviewee

Yes, you give health talk about Hepatitis B, how to prevent it, and how to take care of the patients if the person contracted it and we give strong a health talks about this hepatitis B. During Pregnancy ANC and during RI for mothers that came for that.

Interviewer

Okay, so is the hepatitis B birth dose easy to get within 24 hours of it, is it easy to assess

Interviewee

is is easy for those that have solar fridge or or other freezers

Interviewer

Do you have solar fridge

Interviewee

Exactly

Interviewer

Okay, so is it easy to give

Interviewee

Exactly

Interviewer

Is it easy to get it within 24 hours

Interviewee

If we notice the baby has no joidice we give immediately.

Interviewer

Okay, So, okay, for you if the baby don't have joidice you won't give

Interviewee

Yes.

Interviewer

Okay, So you see Javing jondice as a contra indications

Interviewee

Yeah, the definition of joidice is the same thing like hepatitis yellowish coloration of the skin and the yellowish of the conjunctivitis of the eyes. When you give the vaccine it will wash the case.

Interviewer

Okay, So who can administer Hepatitis B, in your opinion, who can administer

Interviewee

health workers that is in their facility

Interviewer

That's all of them or are there categories of persons you think are suppose to administer it,

Interviewee

like health workers can do

Interviewer

If you say health workers it Like a big umbrella

Interviewee

Like nurses, Chew and Jchews

Interviewer

okay. So what are the challenges or barriers to getting the hepatitis B birth dose vaccine are there any challenges?

Interviewee

they are challenges for those that don't have sola fridge, but as for me i don't have any challenges of giving this because I have solar fridge.

Interviewer

so have you gotten any information from participant, That what i mean by participant I mean, those that have gotten a privacy vaccination, have you ever gotten any information from them? regarding their experience with the vaccines, hepatitis B birth dose, Have you gotten any feedback from people that have been vaccinated or whose children have been vaccinated with hepatitis B birth dose, have they given you any report, good or bad reports, we just need your feedback.

Interviewee

Like for our area, mothers are rejoicing that in the olden days, that many children's is dying, because of this communicable disease or something like that but when the case came and immediately like, if you have administered that vaccine, if the infection came, it will not be worsten than than those that have not given and know the importance of immunization as of now. Yes.

Interviewer

So what is the community understanding of hepatitis B in your opinion, What is the community understanding of hepatitis B birth dose. Community or the community? How do You think they actually understand hepatitis B birth dose, what is it all about in your opinion

Interviewee

Yes, in my own opinion, they understand it, because they say that in the olden days that this disease is killing their children. So, from now, we are now in a computer age and the health center and health post are now available in everywhere, the now receive information about the importance, advantages and disadvantages of that virus.

Interviewer

So So, are people interested in getting the hepatitis B birth dose

Interviewee

Exactly

Interviewer

So can you describe what kind of specific information or messaging you have heard about the hepatitis B birth dose, Can you describe what kind of information that you have? or specific messaging that you have gotten about hepatitis B birth dose

Interviewee

There is one training we had here, the taught us that any child, you diagnose or you suspected that the baby is having joidice we should not give. So that is one thing announce of hepatitis B

Interviewer

So any other thing, any other information Annouce

Interviewee

No

Interviewer

Ok, That is the major key method. Okay, so do you have any idea or suggestion? for improvement? How do you think we can actually improve Hepatitis B birth dose rates from what it is now to something higher

Interviewee

OK to my own opinion, like any health post or health centers, they should provide cold box or fridge so that they will encrypt or install the vaccines to maintain the potency of the vaccine so that any child born immediately should receive.

Interviewer

So, I also want to guide in some way we like at the community, what suggestions do you have that could help us improve the vaccinations

Interviewee

To continue steady mobilization. like during this last august meeting, we can do talk to mothers to know the importance of immunization and otherwise, okay, for me, I will explain more for them. I am the supervisor for family planning, and otherwise, I do give them health talks on family planning, I am the service provider

Interviewer

Okay, What about the health? You know, you've just mentioned that for the health community for the community level, What about the health care system level that is, at the management level, What suggestions you think you have to help things that can help us improve on vaccination of hepatitis B

Interviewee

Yes, to make sure that the data tools is available, Because anything you do without documentation is zero

Interviewer

okay. So then now, thank God you said you are a service provider. So at the provider level, I'm in your facility, what can we do at the facility level that can help us improve vaccination rate of hepatitis B.

Interviewee

To make sure that there is no lack of a vaccine, They will bring the vaccine to our various facilities. The strange is from 000 0.05 and 0.5. And even mixing rages is available at all time, and make sure that our data is complete on the path.

Interviewer

okay. Thank you very much for that feedback. So, going forward, I guess Pacific strategies for education or awareness that might be very effective. In your opinion, strategies you'd like us to implement that you think if we do so, it will help improve health education, and awareness for the people.

Interviewee

We need more training about this hepatitis because, it is a deadly disease.

Interviewer

So can you tell me about your experience experience with maternal tetanus? I'm looking at it. Can you tell me your experience with maternal tetanus vaccination? What are your experiences

Interviewee

for those that don't receive the TT one TT two to otherwise, Unfortunately, I have witnesses those that goes to TBAs, Yeah, am in my facility and they carry jus neonate of two days. And the baby is having that lock jaw or something like that, I asked questions to the mother, just TBA i know, the person does not go to health centers to receive TT one, TT two and otherwise. So I advised them that each pregnancy mothers is supposed to receive at least first those or second dose before delivery, if she can take it for life after delivery come by six months, one year for that six months and one year after. That's one life whatever you need subsequent, like 10 minutes in each subsequent pregnancy.

Interviewer

Okay. So what barriers or challenges have you experienced? related to the maternal tetanus vaccination? What challenges have you experienced related to tetanus vaccination? What are the challenges or barriers.

Interviewee

I don't see any barriers because I have solar and the vaccine is there, so any person that cime for booking will receive after four weeks, will come and receive the second dose. I don't have any barrier because I have solar. And well equipped vaccine.

Interviewer

Ok, so You dont have barriers or challenges, so what are your success stories? What are your success? What what success Have you experienced or what is working for you.

Interviewee

It's working because Since i headed that place, I have not gotten any case of neonatal death or Maternal neonatal death.

Interviewer

Ok, so what Are you doing differently? What do you think you're doing differently than the other two

Interviewee

I am doing what applies to my best method, using sterilized procedure in my delivery, or circumcision or ear piercing, I have to maintain sterile procedure to avoid contamination of any dust that we brought about this tetanus.

Interviewer

So what type of health interventions have been the most successful? right in your community? And why have they been so successful? Of all the intervention so far? which one has actually been fairly successful within your community catchment area?

Interviewee

is ANC even this RI, because many people asked about taking beta one, beta two, beta three, they will ask me that they should come with us to hear that health talks given to them that they are nervous in the field. And I do give them health talks about ANC, family planning and otherwise, and even the importance of immunization.

Interviewer

So, so Wow, that's wonderful to hear from you. So is there anything else you think we should know about Hepatitis B, or probably about informations related to hepatitis B, or something specific to hepatitis B that actually i haven't asked you, or any other information or thing you'd like to add? Or you want us to know about?

Interviewee

I don't have any more.

So you've been able to digest all you have, wow It has been a honor hearing from you ma, thank you very much for the work you've been doing. Thank you for the time for giving this audience a long way to help us understand how best to approach issues concerning Hepatitis B. Once again, we what to say thank you, it has been a wonderful moment with madam, the nurse in charge of Amanefi Health Center, Madam Ibuka Ann. thank you very much once more for having you on board. Thank you.

TRANSCRIBED KII FOR PREGNANT WOMAN State 2, LGA 4, Facility 10

Interviewer:

Ehm, Good morning everybody, so We're here at [facility name] to conduct a key informat interview for one of the pregnant mother that access health Care within this facility, Eh, this afternoon I'm here with my colleague [name] and my humble self [name], so we will be interviewing [name], One of the pregnant women that access care in this facility. So madam "[name]" before we start, we will like to get your Consent to go ahead with this interview, Do you give, or have your consent to go ahead and ask you this questions on records?

Interviewee Response: Yes.

Interviewer:

OK, thank you. So can we get to know you, can you introduce yourself to us?

Interviewee Response:

Your welcome, my name is eh [name], I am a 35 years old woman with four (4) kids

Interviewer:

OK, are you currently pregnant

Interviewee Response: Yes

Interviewer:

So How old is your pregnancy

Interviewee Response:

I am five (5) months pregnant

Interviewer:

Have you been accessing health Care here in this facility

Interviewee Response: yes

Interviewer:

OK thank you very much. So without waisting much of your time madam [name], I will like to know, what do you know about Hepatitis B

Interviewee Response

Ehm ehm, the little I know about Hepatitis B is the first immunization given to a child immediately after birth to prevent the child from contacting Hepatitis B.

Interviewer:

OK, so now your talking about the vaccine, so what about the disease itself, what do you know about the Disease

Interviewee Response

Hepatitis B is an infection that normally infect a child in uterus after birth.

Interviewer:

OK, thank you very much, so what do you know now about hepatitis B birth dose, what is your knowledge about Hepatitis B birth dose

Interviewee Response

Hepatitis B birth dose is the injection given to a child immediately after birth.

Interviewer:

OK, OK immediately after birth, so how do you feel about hepatitis B birth dose, how do you feel about it

Interviewee Response

Eh**,** what I feel about it, the injection is very very important immunization because of the work it has on the child

Interviewer:

OK, OK so have you ever asked for hepatitis B birth dose before for your children or other children

Interviewee Response:

Yes

Interviewer:

When was that

Interviewee Response:

I ask of it, when ever I deliver, i normally ask the nurse when I'm to come for it

Interviewer:

OK, so do you plan to get hepatitis B birth dose for tho baby, the one you are carrying now

Interviewee Response:

Yes

Interviewer:

OK, so why will you want to do that

Interviewee Response:

Ehm is because I want, I want my children to be prevented against this Hepatitis B

Interviewer:

OK, so haven't had this plan now, do you have any concern, any thing that is a concern that you wish to share with us about hepatitis B, both the vaccination, the birth dose or is there any excitement about it, any feeling or is there any stress you have around hepatitis B birth dose

Interviewee Response:

Eh**,** the excitement I have of it because of the preventive method it has on my children

Interviewer:

OK, so you're excited because it going to prevent you're children

Interviewee Response:

Yes

Interviewer:

Ok, so do you think the hepatitis B birth dose works to prevent hepatitis B. Do you think it works

Interviewee Response:

Yes, its works

Interviewer:

OK, so why do you think so

Interviewee Response

Yes because the rate children dies this days is not as before, because of this hepatitis B. The government has given us. Its prevent death of children, rapid death rate.

Interviewer:

OK, OK so do you think you could easily get the hepatitis B birth dose vaccine if you ask for it

Interviewee Response:

Yes

Interviewer:

OK, so will you ask for it

Interviewee Response:

Yes I will ask for it

Interviewer:

Why will you ask for it

Interviewee Response:

Yes, because of the important of it in my children

Interviewer:

OK, so is there anything that might come in the way or post a challenge that you may face related to accessing birth dose for hepatitis B, is there any challenge or problem you may face

Interviewee Response:

The challenge is maybe if I come to a clinic and I I'm not able to meet any of the health worker, it will be a challenge because I'm not able to get it at that moment, I will feel sad.

Interviewer:

OK, so do you feel that hepatitis B birth dose is important to get for you're child

Interviewee Response:

Yes

Interviewer:

Why, why do you feel so

Interviewee Response:

Because of it importance on the children

Interviewer:

Because of the importance to your children, wow so speaking generally now, do you feel pregnant women in you're region, I mean within this region of yours and you feel the get hepatitis B birth dose to their child

Interviewee Response:

I can't say for any other person, though since I've been coming here for ante-Nata in this health center, the nurse I meet here, they're working hard, very very hard to make sure that immunization is given to children

OK, including hepatitis B

Interviewee Response:

Yes

Interviewer:

OK, so what gives you that level of confidence that this people you know are actually hard working, and that their hard work is equally going to help to immunized the children, what gives you that confidence

Interviewee Response:

What gives me that confidence is because immunization is perform almost weekly, on weekly basis here in the center I visit

Interviewer:

Ok, so now with this confidence that you have, do you have any suggestions for us, we will like you to suggest for us like what we can do, like what we can educate your people in your community about hepatitis B birth dose, what do you suggest

Interviewee Response:

I suggest that the government should try to give all information on hepatitis B on radio and television, even on, maybe in churches and schools, we announce to them about it

Interviewer

OK, in televisions, schools and churhes, OK so will you prefer to learn, I what way now will you prefer to learn about hepatitis B, like in what strategies you as a person, how will you prefer to learn about hepatitis B, in what way will you prefer to be taught, ah don't know if you get my question, there are so many ways one can learn about it, so many ways, so what ways will you prefer

Interviewee Response:

by giving health talk, by giving more health talks on it, I will learn better

Interviewer

OK, so what about general speaking about health issue's generally now, what ways and avenues do you used to learn more about health generally

Interviewee Response:

OK, we use television, radio or televisions and radio in shot.

Interviewer

OK television and radio you can used to learn more about hepatitis B. So is there anything else you will like us to know, you think we should know about hepatitis B,. Seeing that this program is been cantered in hepatitis B, is there anything else, and key information you think you can give us, any insight you can give us about hepatitis B expercially as it relate to dissemination of hepatitis B information within the community and also administration of birth dose of hepatitis B, is there any you wi love to share with us

Interviewee Response:

Not really..

Interviewer

OK you have exhausted everything you think we should know

Interviewee Response:

Yes

Interviewer

OK, we appreciate you so much for finding time in granting us this audience, so be rest assured that your responses have been well noted and will be put into good used and will also help shape and make inform decisions. Thank you very much once more, thank you I'm out.
